# e-Bitter: Bitterant Prediction by the Consensus Voting From the Machine-Learning Methods

**DOI:** 10.3389/fchem.2018.00082

**Published:** 2018-03-29

**Authors:** Suqing Zheng, Mengying Jiang, Chengwei Zhao, Rui Zhu, Zhicheng Hu, Yong Xu, Fu Lin

**Affiliations:** ^1^School of Pharmaceutical Sciences, Wenzhou Medical University, Wenzhou, China; ^2^Chemical Biology Research Center, Wenzhou Medical University, Wenzhou, China; ^3^Center of Chemical Biology, Guangzhou Institutes of Biomedicine and Health, Chinese Academy of Sciences, Guangzhou, China

**Keywords:** QSAR, bitter taste, bitterant prediction, classification, machine learning, taste prediction

## Abstract

*In-silico* bitterant prediction received the considerable attention due to the expensive and laborious experimental-screening of the bitterant. In this work, we collect the fully experimental dataset containing 707 bitterants and 592 non-bitterants, which is distinct from the fully or partially hypothetical non-bitterant dataset used in the previous works. Based on this experimental dataset, we harness the consensus votes from the multiple machine-learning methods (e.g., deep learning etc.) combined with the molecular fingerprint to build the bitter/bitterless classification models with five-fold cross-validation, which are further inspected by the Y-randomization test and applicability domain analysis. One of the best consensus models affords the accuracy, precision, specificity, sensitivity, F1-score, and Matthews correlation coefficient (MCC) of 0.929, 0.918, 0.898, 0.954, 0.936, and 0.856 respectively on our test set. For the automatic prediction of bitterant, a graphic program “e-Bitter” is developed for the convenience of users via the simple mouse click. To our best knowledge, it is for the first time to adopt the consensus model for the bitterant prediction and develop the first free stand-alone software for the experimental food scientist.

## Introduction

Bitter taste is one of the basic taste modalities including the bitterness, sweetness, umaminess, saltiness, acidness, and fatness (Besnard et al., 2016; Roper and Chaudhari, 2017). Evolutionarily, bitter taste is pivotal to the survival by protecting organisms from the consumption of potentially poisonous substances, which often taste bitter. Perception of the bitter taste is mainly mediated by the taste receptors type 2 (Tas2Rs) family of G-protein coupled receptors (GPCRs) on the apical membrane of the taste receptor cells located in the taste buds (Jaggupilli et al., 2016; Roper and Chaudhari, 2017). Intriguingly, Tas2Rs are also expressed in the extra-oral tissues, e.g., the gastrointestinal tract and respiratory system, etc., indicating that they are also intricately involved in the other crucial biological processes (Clark et al., 2012; Shaik et al., 2016).

In humans, 25 different *h*Tas2Rs are evolved to bind the bitterants with diverse chemical structures (Behrens and Meyerhof, 2013; Jaggupilli et al., 2016). Some Tas2Rs such as Tas2R10, Tas2R14, and Tas2R46 broadly accommodate the various bitterants, while some Tas2Rs such as Tas2R50 exquisitely select the specific bitterants (Meyerhof et al., 2010; Brockhoff et al., 2011; Ji et al., 2014). At the same time, the promiscuous bitterant can interact with multiple Tas2Rs, while the selective bitterant can only activate one or few specific Tas2Rs (Di Pizio and Niv, 2015). Therefore, the compound that can stimulate at least one Tas2R can be treated as a bitterant, however, only the compound that cannot activate any of 25 *h*Tas2Rs can be defined as a non-bitterant, since a compound cannot stimulate one specific Tas2R, which could still elicit the bitterness via targeting another Tas2R.

Bitterness is often perceived as an unpleasant taste, albeit it is considered desirable in some products such as tea, coffee, and beer etc. In most cases, bitterness influences the palatability of the functional beverage and food containing the bitter ingredients, and also poses a major problem for the patient acceptability and compliance of the bitter-taste drugs, especially for the pediatric formulations (Drewnowski and Gomez-Carneros, 2000; Mennella et al., 2013). Therefore, the bitter-tasting assessment is imperative in the functional food/beverage development, and could be considered in advance during the drug discovery process.

Bitter-tasting assessment is often an arduous and tedious task. Basically there are two types of experimental taste evaluations: *in-vivo* and *in-vitro* approaches, which are systematically reviewed by Anand et al. (2007). One of the most direct methods is called “human taste panel studies,” which evaluates the taste of standard and test stimuli in the healthy human volunteers with the well-designed protocols (Anand et al., 2007). However, this experimental method has its major disadvantage due to the higher probability of toxicity for the bitter compounds, which will cause the safety and ethical issues and consequently limit its application in the high-throughput screening of the bitterants. In contrast to the experimental methods, *in-silico* method provides a cheap and rapid alternative to identify the most likely bitterants from the small-molecule database (Bahia et al., 2018). Thus, the computational prediction of the bitterant becomes more and more important prior to the laborious and time-consuming experimental taste assessment.

Current commonly-used computational methods for the bitterant prediction are categorized by Bahia et al. which are listed as follows: (Bahia et al., 2018) structure-based method (Floriano et al., 2006; Brockhoff et al., 2010; Singh et al., 2011; Tan et al., 2011; Marchiori et al., 2013; Sandal et al., 2015; Acevedo et al., 2016; Karaman et al., 2016; Suku et al., 2017), ligand-based method (Roland et al., 2013, 2015; Levit et al., 2014) and machine-learning based method (Rodgers et al., 2006; Huang et al., 2016; Dagan-Wiener et al., 2017). Structure-based method requires the 3D structures of Tas2Rs, whose crystal structures still remain unresolved. In contrast, ligand-based method approach such as the 3D-pharmacophore method still works even in the absence of 3D structures of Tas2Rs. Both methods work well for the particular bitter-taste receptor, Nevertheless, a compound that cannot activate one specific Tas2R could still trigger the bitter taste via stimulating the other 24 *h*Tas2Rs. Thus, both methods are not suitable for the general classification of bitterant/non-bitterant. Machine-learning based approach can effectively circumvent the aforementioned problems and can directly predict the bitter or bitterless compounds (Rodgers et al., 2005, 2006; Huang et al., 2016; Dagan-Wiener et al., 2017; Bahia et al., 2018). In this emerging method, certain experimental dataset including both the bitter and bitterless compounds is employed to establish the prediction model, while the target information of the bitter compound is not necessary, which confers the unique advantage on this method.

There are three typical studies about the general bitterant prediction with the machine-learning approach based on the relatively large dataset (Rodgers et al., 2006; Huang et al., 2016; Dagan-Wiener et al., 2017), although there are several studies about the congeneric systems with the comparatively small dataset (Takahashi et al., 1982; Spillane et al., 2002; Cravotto et al., 2005; Scotti et al., 2007). In addition, all these studies focus on the prediction of small-molecule bitterant, which is our current main research interest, thus the prediction of bitter peptide explored in the other studies (Ney, 1979; Soltani et al., 2013) will not be reviewed here.

Rodgers et al. employ the Naive Bayes algorithm and circular fingerprint (MOLPRINT 2D, Willett et al., 1998) to classify the bitter/bitterless compounds (Rodgers et al., 2006). The dataset consists of 649 bitterants and 13,530 hypothetical non-bitterants. All the bitterants are from their proprietary database, while 13,530 hypothetical non-bitterants are randomly selected from the MDL Drug Data Repository (MDDR). The prediction model gives the best accuracy, precision, specificity, and sensitivity of 88, 24, 89, and 72% respectively in the five-fold cross-validation. It's the first bitterant prediction model trained with the large dataset. Nevertheless, the bitterless compounds in their study are not experimentally confirmed, and their work didn't provide a practical prediction tool for the users to have a test on their model.

Huang et al. developed the first online prediction tool called “BitterX,” which combines Support Vector Machine (SVM) approach (Vapnik, 1995) with the physicochemical descriptors (Huang et al., 2016). In their study, the dataset is composed of 539 bitterants and 539 non-bitterants. Five hundred thirty-nine bitterrants are gathered from the literature and the publicly available BitterDB (Wiener et al., 2012). For 539 non-bitterants, 20 non-bitterants are from their in-house bitterless compounds validated by the experiments, and 519 non-bitterants are the representative structures clustered from the compounds without the tag of “bitter” in the Available Chemicals Directory (ACD) database (http://accelrys.com). Their bitterant prediction model offers the impressive accuracy (91~92%), precision (91~92%), specificity (91~92%), and sensitivity (91~94%) on the test set. However, 519 compounds assumed as the non-bitterants are still not confirmed by the experiments. Thus, the limited number of experimental non-bitterants are the bottleneck for the machine-learning based approach.

Recently Wiener et al. published a prediction tool named “BitterPredict,” which adopts 12 basic physiochemical descriptors and 47 Schrödinger QikProp descriptors (Dagan-Wiener et al., 2017). In this work, the classification method is the adaptive ensemble machine-learning method “Adaptive Boosting” (AdaBoost), whose advantage is that this method is simple, fast, less susceptible to the overfitting. Meanwhile, the dataset is larger than the counterpart in Huang et al. and comprises 691 bitterants and 1,917 non-bitterants. The bitterants are mainly from their BitterDB (Wiener et al., 2012), and the work of Rojas et al. (2016) The non-bitterants are composed of 1,360 non-bitter flavors, 336 sweeteners, 186 tasteless compounds, and 35 non-bitter molecules (Eric Walters, 1996; Arnoldi et al., 1997; Ley et al., 2006; Rojas et al., 2016). The last four sets of compounds are experimentally confirmed, whereas 1,360 non-bitter flavors are gathered from the Fenroli's Handbook of Flavor Ingredients (Burdock, 2004), and are hypothetically defined as the non-bitterants if the word “bitter” is not explicitly mentioned in the description section of each compound in the book (Burdock, 2004). Their prediction model gives the accuracy (83%), precision (66%), specificity (86%), and sensitivity (77%) on the test set. Nevertheless, majorities of non-bitterants (1,360 non-bitter flavors) are still hypothetical. In addition, BitterPredict works in the environment of commercial MATLAB package and requires the commercial Schrödinger software to generate molecular descriptors, which will hamper the extensive test by the users. So far, users can only send the data to authors for the prediction.

In short, all three works adopt Naive Bayes, SVM or Adaboost as the classification method. The recently popular machine-learning methods such as deep neuron network (DNN) (LeCun et al., 2015), random forest (RF) (Breiman, 2001), and gradient boosting machine (GBM) (Friedman, 2002), frequently manifest the promising performance in the kaggle competition (www.kaggle.com/competitions), but were not used in the bitterant prediction before. In addition, the simple K-nearest neighbors (KNN) method (Itskowitz and Tropsha, 2005), which is generally used as the baseline for the comparison of machine-learning methods, was never applied in the bitterant prediction as well. Moreover, the consensus voting strategy based on the multiple machine-learning methods also was not employed to build the bitterant classification model in the past. Secondly, the previous works make use of the fully or partially hypothetical non-bittterant dataset. Therefore, there is a pressing need to make use of the fully experimental dataset with the relatively large size to develop the consensus model for the bitterant prediction that can be utilized by the food scientists in an easy-to-use and free software.

In this work, we collect only the experimentally confirmed bitterants and non-bitterants. Based on this fully experimental dataset, we adopt the popular Extended-connectivity Fingerprint (ECFP) (Rogers and Hahn, 2010) as the molecular descriptors and propose the consensus voting from the current mainstream machine-learning methods such as KNN, SVM, RF, GBM, and DNN to build the bitterant/non-bitterant classification models. All the models are carefully inspected by the Y-randomization test to ensure their reliability, and some promising models are subsequently selected to construct nine consensus models that are integrated in our program for the bitterant prediction. To aid the food scientists to automatically predict whether the compound of interest is bitter or not, we present a convenient graphic program called “e-Bitter,” which natively implements ECFPs for the automatic generation of the molecular descriptors. More importantly, e-Bitter can intuitively visualize the inter-connected 3D structural feature, feature importance and feature partial derivative for any specific bit “1” in ECFP. At last, the performance and functions of our program compared with other bitterant prediction tools are discussed.

## Materials and methods

### Data collection and preprocess

An appropriate experimental dataset including both the bitterants and non-bitterants are critical to properly build the reasonable prediction model. Three criteria are defined for our data curation. (1) All the disconnected structures such as salts are not considered. (2) Only the compounds with the common elements C, H, O, N, S, P, Si, F, Cl, Br, or I are collected. (3) The same compound labeled with the different taste qualities will be excluded. (4) The duplicated compounds from the different sources will be removed. Based on these criteria, all the compounds are curated as the Tripos mol2 format.

In our work, majorities of bitterants are downloaded from the publicly available BitterDB (Wiener et al., 2012), and the others are retrieved from the literature (Rodgers et al., 2006; Rojas et al., 2016). The total number of the bitterants is 707. However, the data source of the non-bitterants raises a tough issue, since most of the published works often did not report the non-bitterants due to the less scientific significance. Hence only 132 tasteless and 17 non-bitter compounds retrieved from the literature are treated as the non-bitterants (Huang et al., 2016; Rojas et al., 2016). In order to further extend the size of the bitterless dataset, we tentatively propose to use the sweet molecules that can be generally assumed as the non-bitterants. The sweet compounds are downloaded from the SuperSweet (Ahmed et al., 2011) and SweetenersDB (Chéron et al., 2017). Database and additionally gathered from the literature (Zhong et al., 2013; Cristian et al., 2016; Rojas et al., 2016), which results in 443 compounds. The whole dataset, containing 707 bitterants and 592 non-bitterants, is publicly available in our e-Bitter program, with which users can handily view the 3D structure of each compound and its corresponding label (Y: bitterant or N: non-bitterant).

**Figure 1:**
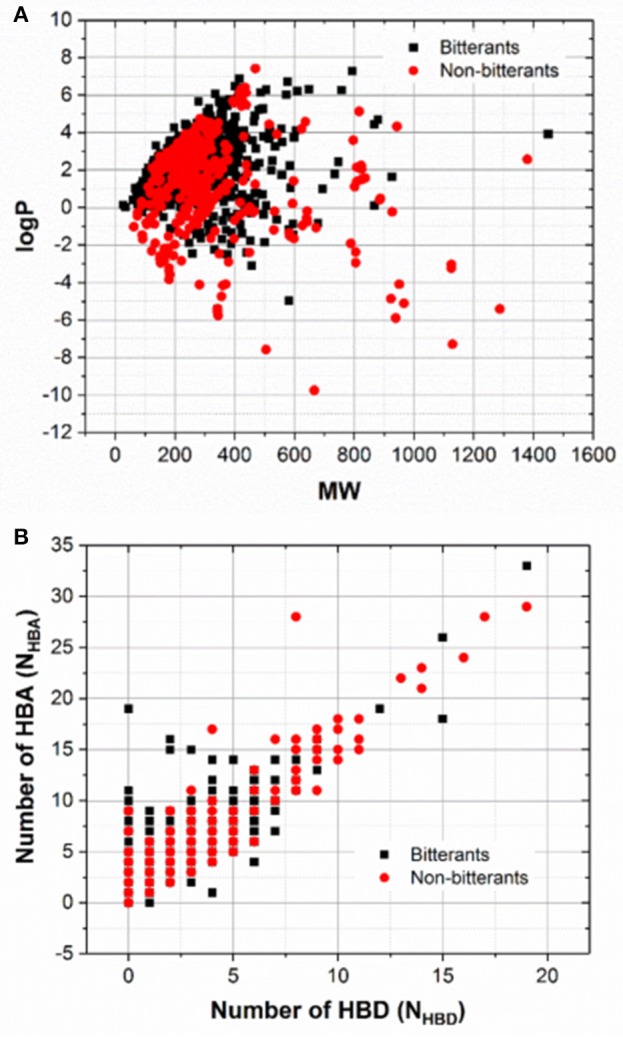
The scatter plots of MW vs. logP **(A)** and N_HBD_ vs. N_HBA_ **(B)**. N_HBD_ and N_HBA_ refer to the numbers of hydrogen-bond donors and acceptors respectively.

**Figure 2:**
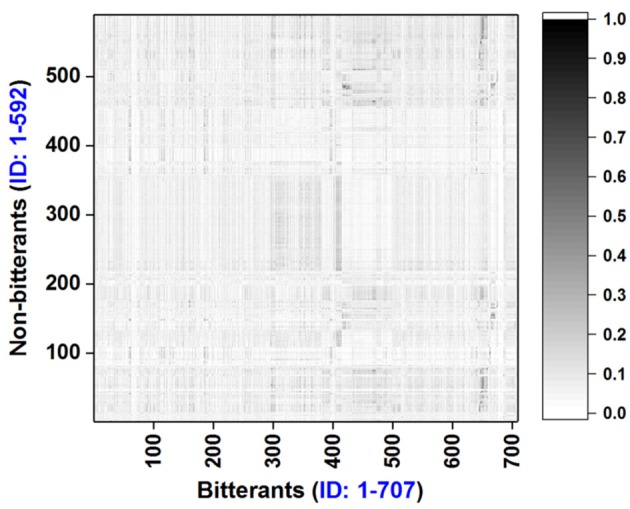
Tanimoto similarity matrix for the bitterants vs. non-bitterants. Similarity is calculated based on the 2048bit-ECFP6 fingerprint with our e-Bitter program.

To explore the chemical space of our dataset, molecular weight (MW), logP, and the numbers of hydrogen-bond donor and acceptor (N_HBD_ and N_HBA_) for all the bitterants and non-bitterants are calculated with Openbabel v2.4 (O'Boyle et al., 2011). The histograms of logP, MW, N_HBD_, and N_HBA_ are plotted in Figures S1–S4 and the scatter plots of logP vs. MW and N_HBA_ vs. N_HBD_ are shown in Figures 1A,B respectively. Furthermore, the Tanimoto similarity matrix (Figure 2) between bitterants and non-bitterants is calculated based on the 2048bit-ECFP6 due to its more features and less bit collisions.

In order to derive and validate the bitter/bitterless classification model, the whole dataset is randomly split into two chunks: the dataset for the cross-validation and the test set for the independent validation. The detailed data-splitting scheme is given as follows: 20% of the bitterants and 20% of the non-bitterants (141 bitterants and 118 non-bitterants) randomly selected from the whole dataset are treated as the test set (**Dataset-Test**), while the rest of them (566 bitter and 474 bitterless compounds) are adopted to train the model with the cross-validation (CV), which is denoted as **Dataset-CV**. In the five-fold cross-validation, **Dataset-CV** is randomly split into five chunks. One chunk is employed as the internal validation set (**Dataset-Internal-Validation**), and the remaining four chunks are combined to form the training set (**Dataset-Training**). This procedure will be repeated for five times, which is prepared for the five-fold cross-validation. Finally, to reduce the bias from the data-splitting scheme, the whole data-splitting procedure will be repeated for nineteen or three times depending on the different machine-learning methods. More specifically, 19 data-splitting schemes are adopted for the model-training with KNN, SVM, GBM, and RF, while only three data-splitting schemes are used for the model-training with DNN2 and DNN3 that are very computationally expensive.

### Molecular descriptors for the machine-learning algorithms

In this work, Extended-connectivity Fingerprint (ECFP) (Rogers and Hahn, 2010) is adopted as the molecular descriptor. Thus, ECFP is implemented natively in our e-Bitter program due to the following three factors. (1) ECFP, one typical class of topological fingerprints, was manifested to be powerful in the classification (Ekins et al., 2010; Rogers and Hahn, 2010; Chen et al., 2011; Hu et al., 2012; Braga et al., 2015, 2017; Koutsoukas et al., 2016; Rodríguez-Pérez et al., 2017; Varsou et al., 2017; Wang et al., 2017; Yang et al., 2017). However, ECFP has not been applied to the general classification of diverse bitter/bitterless compounds in the literature. (2) The existing softwares with the ECFP function such as Pipeline Pilot (http://accelrys.com), JCHEM (https://www.chemaxon.com), and RDKit (http://www.rdkit.org) etc. cannot provide a facile and intuitive mean to highlight the fingerprint bit “1” in the context of the 3D structure and also cannot inform us the importance of each bit. But it is worth mentioning that Bioalerts program (Cortes-Ciriano, 2016), which is developed based on the RDKit, can offer a very useful function to generate the 2D structure image highlighting with one ECFP bit. Nevertheless, Bioalerts doesn't have a 3D graphic frontend to support the interactive visualization. (3) The native integration of ECFP in our e-Bitter program will decrease the software dependency on any other packages and will be convenient for the users to deploy this program on their own computers. The implementation of ECFP is given as follows.

The generation procedure of ECFP can be divided into the following steps (Rogers and Hahn, 2010). (1) Initial assignment of the atom identifiers. The initial integer identifiers, which are assigned to all the non-hydrogen atoms of the given molecule, encode the local information about the corresponding atom such as Sybyl atom type, atomic number, and connection count etc. (2) Iterative update of the atom identifiers. Each atom identifier is recursively updated to reflect the identifiers of each atom's neighbors till a specified diameter is reached. The commonly defined diameter is 4 or 6. (3) Duplication elimination of the atom identifiers. The multiple identifiers representing the equivalent atom-environment are removed. (4) Folding operation of the atom identifiers. All the identifiers are mapped into the bit string with the fix-sized length, in spite of the occasional bit collision. The frequently-used length of the fingerprint is 1,024 or 2,048 bits. (5) Record of the structural features. Finally all the fingerprint bits “1,” original identifiers and their corresponding structural features are recorded for the subsequent visualization. This step is purposely designed to couple with our 3D visualization platform. In this study, 1024bit-ECFP4, 2048bit-ECFP4, 1024bit-ECFP6, and 2048bit-ECFP6 will be harnessed in the following model-training, since more bits will reduce the chance of bit collision during the folding operation, while the larger diameters will provide more structural features.

### Feature selection based on the feature importance

In this work, both full features without the feature selection and feature subset with the feature selection are attempted to examine whether the feature selection is beneficial to our bitterrant/non-bitterant classification. Herein, feature selection is conducted based on the feature importance (Teixeira et al., 2013) derived from the model-training with the random forest (RF) method, which will be elaborated in the following section. Thus, the full features without the feature selection, and the feature subset after the feature selection are adopted as the molecular descriptors to systematically evaluate the performance of bitter/bitterless classification.

### Model training without the prior feature selection

In this work, five algorithms (KNN, SVM, RF, GBM, and DNN) will be utilized to train the models via the Scikit-learn, Keras and TensorFlow python libraries, which are fully integrated in the Windows version of python package (Winpython 3.5.4.0) with the download site (https://winpython.github.io/). For the sake of the fine tuning of hyper-parameters, the five-fold cross-validation is conducted to explore the corresponding optimal parameters for each machine-learning method, which will be succinctly introduced as follows.

K-nearest neighbors (KNN) algorithm is a non-parametric method used for the classification, which is based on the closest training instances in the feature space (Itskowitz and Tropsha, 2005). The number of nearest neighbors (K) and the weighting methods will affect the performance of KNN model. In this study, K (1, 3, 5, 7, 9, 11, 13, and 15) and two weighting schemes (uniform weight or distance-dependent weight) are explored during the cross-validation.

Support Vector Machine (SVM) is a popular machine-learning technique that performs the classification by constructing the hyper-planes in the multi-dimensional space that separates the different classes (Vapnik, 1995). The radial basis function (RBF) is used as the kernel, and the grid search is harnessed to optimize the penalty parameter C (1,000, 5,000, 10,000, 50,000, and 100,000) and the kernel parameter gamma (0.0001, 0.0005, 0.001, 0.005, 0.01, and 0.1).

Random forest (RF) is an ensemble learning method by generating the multiple decision trees via the bootstrap sampling of training set and random selection of feature subset from the total descriptors (Breiman, 2001). Finally, RF predicts the class based on the consensus votes from these multiple decision trees. In addition, RF can provide the importance of each feature, which is very useful for the intuitive interpretation of the prediction model and is the key criterion for our feature selection in the following section. The number of decision trees (10, 50, 100, 200, 300, 400, 500, 600, 700, 800, 900, and 1,000) will be probed during the cross-validation.

Gradient boosting machine (GBM) is also an ensemble machine learning technique to construct the multiple decision trees in a step-wise manner. Each decision tree is not randomly generated as in the random forest, but is consecutively built to give a better estimate of the response variable. More specifically, GBM is to stepwisely construct a new decision tree as a weak learner with the maximum correlation to the negative gradient of the loss function (Friedman, 2002). The number of decision trees (10, 50, 100, 200, 300, 400, 500, 600, 700, 800, 900, and 1,000), and the learning rate (0.1, 0.2, 0.3, 0.4, 0.5, 0.6, 0.7, 0.8, and 0.9) will be tried during the cross-validation.

Deep neuron network (DNN) is a neural network with more than one hidden layer between the input and output layers. In DNN, thousands of neurons in each layer can be extensively applied to the dataset with thousands of features, and more advanced regularization technique such as the dropout can be used to prevent the overfitting problem (LeCun et al., 2015). Nevertheless, DNN requires the users to adjust a variety of parameters. The number of epochs, the size of mini-batches and the dropout rate are the most important parameters. The number of epochs refers to the number of times that the model is exposed to the training dataset. The size of mini-batches defines the number of training samples exposed to the model before updating of the weight. The dropout rate is the percentage of neurons that are randomly-selected and ignored during the training. In this study, the number of epochs (100, 200, 300, 400, 500, and 600), the size of mini-batches (60, 80, 100, 120, 140, and 160), and the dropout rate (0.1, 0.2, 0.3, 0.4, and 0.5) will be probed in the cross-validation. The dropout technique is exerted only after each hidden layer. Moreover, four configurations of deep neuron network with the different numbers of hidden layers (2 or 3 layers) and neurons per layer (1,024 or 2,048) are explored, which are defined in detail as follows: **DNN2** (Figure S5) contains two hidden layers [input layer: X (1,024 or 2,048) neurons; hidden layer1: X neurons; hidden layer2: X neurons; output layer: 2 neurons], and **DNN3** (Figure S6) includes three hidden layers [input layer: X (1,024 or 2,048) neurons; hidden layer1: X neurons; hidden layer2: X neurons; hidden layer3: X neurons; output layer: 2 neurons]. Additionally, the rectified linear unit function (ReLU) is used as the activation function. adam algorithm is adopted as the optimizer and “binary crossentropy” is employed for the loss function.

Upon completion of model-training with the five-fold cross-validation, the optimal parameters and the corresponding best models are achieved based on highest F1-score in Equation (1). Thus, the combination of four ECFP fingerprints, different random splits of the dataset, and different machine-learning methods (KNN, SVM, RF, GBM, DNN2, and DNN3) will totally offer 328 trained models with the optimal parameters in Table S1. Subsequently, all those models are evaluated on the test set with the following metrics: accuracy, precision, specificity, sensitivity, Matthews correlation coefficient (MCC) and F1-score (Equations 1–6), which are also listed in Table S1.

(1) $$\begin{array}{r} \text{F}1\text{-score}=2\times \text{TP }/\;\left(2\times \text{TP}+\text{FP}+\text{FN}\right) \end{array}$$

(2) $$\begin{array}{r} \text{Accuracy}=\left(\text{TP}+\text{TN}\right)\;/\;\left(\text{TP}+\text{TN}+\text{FP}+\text{FN}\right) \end{array}$$

(3) $$\begin{array}{r} \text{Precision}=\text{TP }/\;\left(\text{TP}+\text{FP}\right) \end{array}$$

(4) $$\begin{array}{r} \text{Specificity}=\text{TN }/\;\left(\text{TN}+\text{FP}\right) \end{array}$$

(5) $$\begin{array}{r} \text{Sensitivity}=\text{TP }/\;\left(\text{TP}+\text{FN}\right) \end{array}$$

(6) $$\begin{array}{r} MCC=\frac{\left(\text{TP}\times \text{TN}-\text{FP}\times \text{FN}\right)}{\sqrt{\left(\text{TP}+\text{FP})\;(\text{TP}+\text{FN})\;(\text{TN}+\text{FP})\;(\text{TN}+\text{FN}\right)}} \end{array}$$

(7) $$\begin{array}{l} \Delta \text{F}1-\text{score}=\;|\text{F}1-\text{score }(\text{cross}-\text{validation})\\ \;-\text{F}1-\text{score }(\text{testset})| \end{array}$$

Where TP, TN, FP, and FN refer to the true bitterant, true non-bitterant, false bitterant, and false non-bitterant respectively. F1-score and Matthews correlation coefficient (MCC) are commonly used to measure the quality of binary classifications. F1-score (cross-validation) denotes that F1-score is evaluated on the internal validation dataset during the cross-validation, and F1-score (test test) marks that F1-score is assessed on the test set. ΔF1-score is the absolute value of the difference between F1-score (cross-validation) and F1-score (test set). ΔF1-score is calculated to monitor the potential overfitting or underfitting. If ΔF1-score is small, it means that the model performances are similar on the internal-validation dataset and test set. For the sake of the conciseness, F1-score (test set) is reduced to F1, hence the symbol “F1” specifically means that F1-score is evaluated on the test set by default if there is no additional statement in this work.

### Model training with the prior feature selection

Feature selection is commonly adopted to eliminate the redundant features in the machine-learning study. In order to demonstrate whether there is any improvement for our bitterant/non-bitterant classification, feature selection is performed based on the feature importance derived from the random forest (RF) method.

More specifically, as described in the previous section about the model-training without the feature selection, 76 runs of random forest are conducted considering the combination of four ECFP fingerprints and different random splits of the dataset, which will lead to 76 models and the attendant 76 sets of feature importance. Then the feature importance for all the bits in the ECFP fingerprint is sorted descendingly and plotted in Figures S7–S10. Thus, the top 512, 256, and 128 important features (Figures S7–S10) are selected respectively as the typical feature subsets for the following model-training, since the exhaustive and systematic scan of feature number ranging from 1 to fingerprint length is really time-consuming especially for the training of deep neuron networks such as DNN2 and DNN3.

Subsequently, each set of important features are combined with the machine-learning algorithms (KNN, SVM, GBM, RF, DNN2, and DNN3) to train the models respectively. The training process is nearly identical to the aforementioned model-training without the feature selection. The only difference is existed in the configuration of DNN: DNN2 with two hidden layers (input layer: X (512, 256, or 128) neurons; hidden layer1: X neurons; hidden layer2: X neurons; output layer: 2 neurons) and DNN3 with three hidden layers (input layer: X (512, 256, or 128) neurons; hidden layer1: X neurons; hidden layer2: X neurons; hidden layer3: X neurons; output layer: 2 neurons). Thus, the combination of three sets of important features and six machine-learning methods (KNN, SVM, RF, GBM, DNN2, and DNN3), different random data-splitting schemes (three splits for DNN2/DNN3 and nineteen splits for the others) and four ECFPs will lead to 984 models.

After the five-fold cross-validation, the best models are harvested according to the highest F1-scores, and then all the best 984 models are assessed on the test set, which are appended to Table S1. Hence 1,312 models including 984 models with feature selection and 328 models without feature selection are obtained. To reduce the bias from the random data splitting, 96 average models (AM) are derived from 1,312 individual models by averaging over the different data splitting schemes and are tabulated in Table S2.

### Y-randomization test

Y-randomization test (Rücker et al., 2007) is conducted to inspect the reliability of all the 1,312 models. In this test, the experimentally observed labels (bitter or bitterless) for **Dataset-CV** are randomly shuffled without changing the total number of bitterants and non-bitterants (Table S3). Worthy of notice is that some labels are still correct due to this random operation. Thus, the newly generated **Dataset-CV** still contains some true samples but with lot of noise, and its detail is described in Table S3. Subsequently, the five-fold cross-validation on this new dataset is performed with exactly the same molecular descriptors and protocols mentioned in the previous section about the model-training. The best models are determined based on the highest F1-scores assessed on the internal validation dataset during the cross-validation, and further evaluated on the test set (**Dataset-Test**) without any random shuffling. All the evaluation metrics are collected in Table S4.

### Applicability domain analysis

Generally speaking, compounds that are highly dissimilar from all the compounds used in the model-training cannot be predicted reliably (Tropsha, 2010), thus the applicability domain of our models should be defined in accordance with the guideline of Organization for Economic Cooperation and Development (OECD). In this work, each compound in the test set (**Dataset-Test**) is compared with every compound in the cross-validation dataset (**Dataset-CV**) according to the Tanimoto similarity based on 2048bit-ECFP6 fingerprint due to its more structural features and less bit collisions. Subsequently, five most similar compounds from **Dataset-CV** are retrieved and treated as five nearest neighbors for the given compound in **Dataset-Test**, and the average of five similarities is defined as the “average-similarity” between this given compound and these five nearest neighbors. It should be noted that five nearest neighbors are selected here, because the optimal nearest neighbors is five for the best KNN model with full 2048bit-ECFP6 (M0255 in Table S1) based on the highest F1-score (0.927).

Following the definition above, each compound in **Dataset-Test** finds its own five nearest neighbors in **Dataset-CV** and compute its corresponding average-similarity. Similarly, each compound in **Dataset-CV** also retrieves its own five nearest neighbors in **Dataset-CV** and calculates its corresponding average-similarity. Finally the histograms of the average-similarity for **Dataset-Test** and **Dataset-CV** are given in Figure S11 to address the applicability domain of our models. This average-similarity is used to reflect the closeness between the given compound and its neighboring compounds in the cross-validation dataset (**Dataset-CV**). If the average-similarity is close to 1, it means that the given compound can find very similar compounds in the training set, and the prediction for the given compound based on our models is not extrapolated and can be considered as a reliable inference. Nevertheless, in reality it is often very difficult for us to expect that the compound of user's interest can always find very similar neighboring compounds in our dataset. Thus, an appropriate threshold for the average-similarity should be defined based on Figure S11.

### Consensus voting strategy used for the bitterant prediction

In this work, 1,312 individual models (M0001-M1312 in Table S1) and 96 average models (AM01-AM96 in Table S2) are achieved. Although all the models are public available and can be used for the bitterant prediction through the flexible function of “customized model” in our e-Bitter program. However, it would be confusing for the users without any recommendation.

Thus, nine consensus models are proposed based on the balance among the accuracy, speed and diversity of machine-learning methods, and are implicitly integrated in our e-Bitter program. Consensus model 1 (**CM01**) selects 19 best individual models (Table S5) from Table S1 purely based on the highest F1-scores in each data-splitting scheme. Consensus model 2 (**CM02**) chooses the average models (AM32, AM28, AM31, AM11, and AM69 in Table S6) considering each machine-learning method with the highest F1-scores to balance the diversity and performance of machine-learning methods. Consensus model 3 (**CM03**) considers the top average models (AM32, AM26, AM28, AM62, and AM52 in Table S7) with the highest F1-scores. Consensus model 4 (**CM04**) selects the top five average models (AM31, AM49, AM55, AM67, and AM43 in Table S8) trained with KNN. Consensus model 5 (**CM05**) comprises the top five average models (AM32, AM26, AM62, AM50, and AM56 in Table S9) trained with SVM. Consensus model 6 (**CM06**) includes the top five average models (AM69, AM63, AM51, AM33, and AM57 in Table S10) trained with GBM. Consensus model 7 (**CM07**) combines the top five average models (AM28, AM52, AM10, AM46, and AM70 in Table S11) trained with RF. Consensus model 8 (**CM08**) consists of the top average models (AM11, AM23, AM35, AM05, and AM29 in Table S12) trained with DNN2. Consensus model 9 (**CM09**) contains the top average models (AM06, AM36, AM12, AM18, and AM30 in Table S13) trained with DNN3. All the evaluation metrics for each consensus model (Table 1) are obtained by averaging over all the constituent models.

**Table 1:** The comparison of models evaluated on their own test sets reported in the original works.

**Program**	**Model**	**Accuracy (test set)**	**Precision (test set)**	**Specificity (test set)**	**Sensitivity (test set)**	**F1-score (test set)**	**MCC (test set)**
e-Bitter	CM01	0.929(0.012)	0.918(0.014)	0.898(0.020)	0.954(0.017)	0.936(0.011)	0.856(0.025)
	CM02	0.910(0.007)	0.904(0.009)	0.881(0.012)	0.933(0.008)	0.919(0.006)	0.819(0.013)
	CM03	0.914(0.002)	0.905(0.005)	0.880(0.007)	0.942(0.003)	0.923(0.001)	0.828(0.003)
	CM04	0.910(0.003)	0.902(0.007)	0.877(0.011)	0.936(0.004)	0.919(0.002)	0.819(0.006)
	CM05	0.914(0.002)	0.909(0.002)	0.886(0.003)	0.937(0.003)	0.922(0.001)	0.828(0.003)
	CM06	0.897(0.001)	0.892(0.003)	0.865(0.004)	0.925(0.003)	0.908(0.000)	0.794(0.002)
	CM07	0.910(0.002)	0.897(0.005)	0.869(0.008)	0.946(0.004)	0.920(0.001)	0.821(0.003)
	CM08	0.899(0.005)	0.904(0.007)	0.884(0.010)	0.912(0.009)	0.908(0.005)	0.796(0.010)
	CM09	0.896(0.006)	0.899(0.011)	0.877(0.017)	0.913(0.013)	0.906(0.005)	0.791(0.012)
BitterX	SVM	0.918(0.009)	0.917(0.008)	0.917(0.008)	0.920(0.012)	0.918(0.009)	0.836(0.017)
BitterPredict	Adaboost	0.832	0.657	0.855	0.768	0.708	0.595

*(1) No standard deviation can be given for BitterPredict, since only one random data-splitting scheme is adopted in their work. (2) Three random data-splitting schemes are used for BitterX, thus the evaluation metrics for BitterX are shown here via averaging over three different data-splitting schemes*.

### Model comparison among BitterX, BitterPredict, and e-Bitter

Models from the BitterX, BitterPredict and e-Bitter will be compared in two manners: (1) the direct comparison of F1-score (test set) and MCC (test set), which are derived from their own works and (2) the more objective comparison on three external test sets from the recent work of Wiener et al.

**Figure 3:**
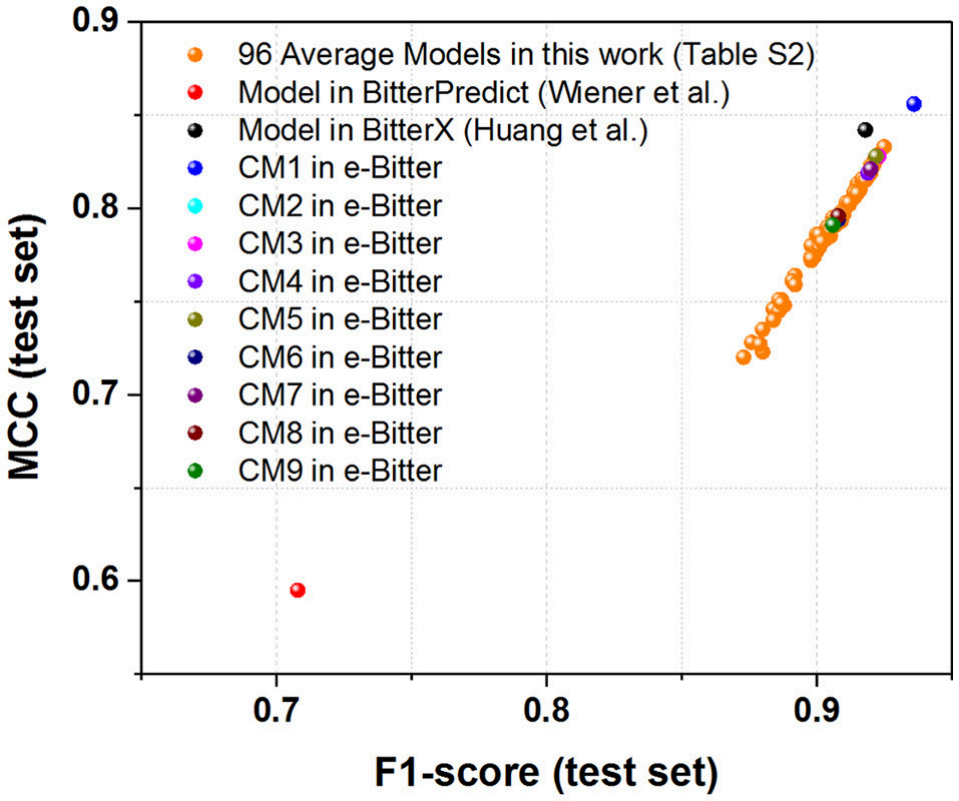
The scatter plot of MCC (test set) vs. F1-score (test set) for our models (9 consensus models and 96 average models) and the models from BitterPredict and BitterX. The MCC (test set) and F1-score (test set) for the models from BitterPredict and BitterX are calculated based on the data reported in the original works.

For the first direct comparison, two performance indicators F1-score (test set) and MCC (test set) should be given for each model. However, both evaluation metrics are not directly reported in the works of Wiener et al. and Huang et al. thus F1-score (test set) and MCC (test set) are indirectly derived from their works. To vividly demonstrate the performance of each model, the scatter plot of MCC (test set) vs. F1-score (test set) is plotted in Figure 3 based on Table 1 and Table S2.

**Figure 4:**
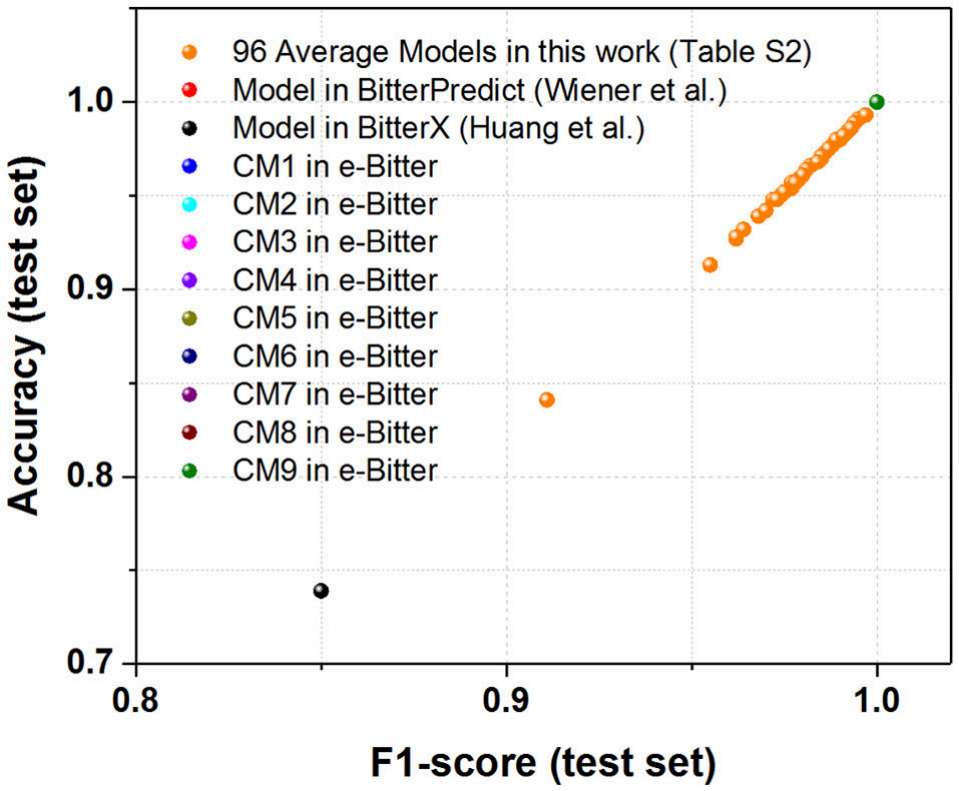
The scatter plot of accuracy (test set) vs. F1-score (test set) for all the models (9 consensus models and 96 average models) evaluated on the “Bitter New” dataset with 23 bitterants. Accuracy instead of MCC is used as Y axis, since MCC cannot be calculated due to the zero number of TF and FP in this dataset without the non-bitterants.

**Figure 5:**
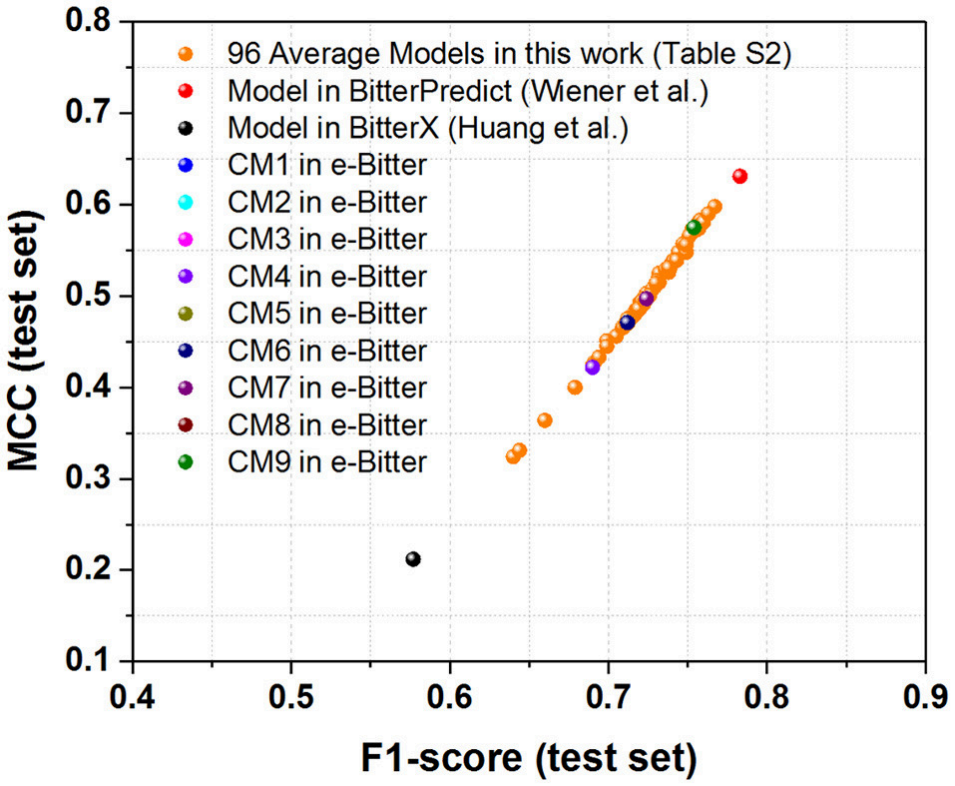
The scatter plot of MCC (test set) vs. F1-score (test set) for all the models (9 consensus models and 96 average models) evaluated on the “UNIMI set” dataset (23 bitterants and 33 non-bitterants).

**Figure 6:**
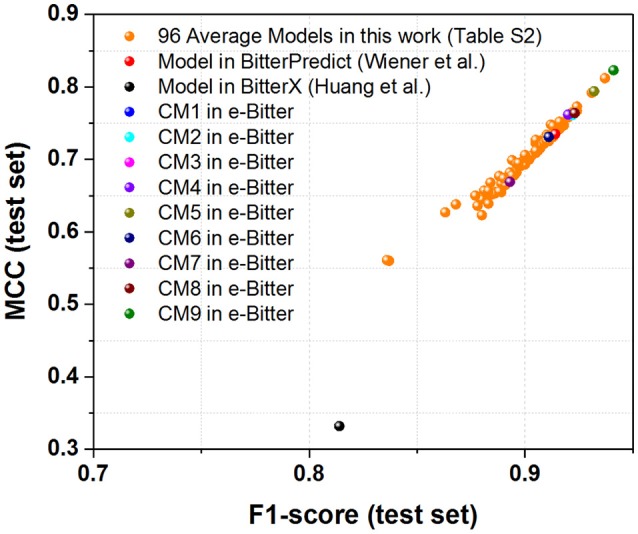
The scatter plot of MCC (test set) vs. F1-score (test set) for all the models (9 consensus models and 96 average models) evaluated on the “Phytochemical Dictionary” dataset (49 bitterants and 26 non-bitterants).

For the further fair comparison, three external independent test sets from the recent work of Dagan-Wiener et al. (2017) are used for the independent assessment and are given as follows: “Bitter New” dataset (23 bitterants), “UNIMI set” dataset (23 bitterants and 33 non-bitterants) and “Phytochemical Dictionary” dataset (49 bitterants and 26 non-bitterants). The prediction results by BitterPredict for these three test sets are reported in the work of Dagan-Wiener et al. (2017) and compiled in Tables 2–4 for the convenience of comparison. BitterX prediction is conducted by the manual uploading of each molecule to the web server one by one, which gives results in Tables 2–4. The prediction by e-Bitter is performed in batch for these three datasets and offers the results in Tables 2–4. In addition, scatter plot of MCC (test set) vs. F1-score (test set) or accuracy (test set) vs. F1-score (test set) for all the models are plotted in Figures 4–6.

**Table 2:** The comparison of models evaluated on the “Bitter New” dataset with 23 bitterants.

**Program**	**Model**	**Failed**	**TP**	**TN**	**FP**	**FN**	**Accuracy (test set)**	**Precision (test set)**	**Specificity (test set)**	**Sensitivity (test set)**	**F1-score (test set)**	**MCC (test set)**
e-Bitter	CM01	0	23	0	0	0	1.000	1.000	–	1.000	1.000	–
	CM02	0	23	0	0	0	1.000	1.000	–	1.000	1.000	–
	CM03	0	23	0	0	0	1.000	1.000	–	1.000	1.000	–
	CM04	0	23	0	0	0	1.000	1.000	–	1.000	1.000	–
	CM05	0	23	0	0	0	1.000	1.000	–	1.000	1.000	–
	CM06	0	23	0	0	0	1.000	1.000	–	1.000	1.000	–
	CM07	0	23	0	0	0	1.000	1.000	–	1.000	1.000	–
	CM08	0	23	0	0	0	1.000	1.000	–	1.000	1.000	–
	CM09	0	23	0	0	0	1.000	1.000	–	1.000	1.000	–
BitterX	SVM	0	17	0	0	6	0.739	1.000	–	0.739	0.850	–
BitterPredict	Adaboost	0	17	0	0	6	0.739	1.000	–	0.739	0.850	–

*(1) No standard deviation can be given in this table, since only one unambiguous predicted label should be provided by our e-Bitter and this predicted label is obtained from the predicted probability averaging over all their respective constituent models. (2) Specificity (test set) and MCC (test set) cannot be given due to the zero number of TN and FP*.

**Table 3:** The comparison of models evaluated on the “UNIMI set” dataset (23 bitterants and 33 non-bitterants).

**Program**	**Model**	**Failed**	**TP**	**TN**	**FP**	**FN**	**Accuracy (test set)**	**Precision (test set)**	**Specificity (test set)**	**Sensitivity (test set)**	**F1-score (test set)**	**MCC (test set)**
e-Bitter	CM01	0	21	18	15	2	0.696	0.583	0.545	0.913	0.712	0.471
	CM02	0	21	18	15	2	0.696	0.583	0.545	0.913	0.712	0.471
	CM03	0	21	19	14	2	0.714	0.600	0.576	0.913	0.724	0.497
	CM04	0	20	18	15	3	0.679	0.571	0.545	0.870	0.690	0.422
	CM05	0	21	19	14	2	0.714	0.600	0.576	0.913	0.724	0.497
	CM06	0	21	18	15	2	0.696	0.583	0.545	0.913	0.712	0.471
	CM07	0	21	19	14	2	0.714	0.600	0.576	0.913	0.724	0.497
	CM08	0	23	18	15	0	0.732	0.605	0.545	1.000	0.754	0.575
	CM09	0	23	18	15	0	0.732	0.605	0.545	1.000	0.754	0.575
BitterX	SVM	1	15	18	14	8	0.600	0.517	0.562	0.652	0.577	0.212
BitterPredict	Adaboost	0	18	28	5	5	0.821	0.783	0.848	0.783	0.783	0.631

*(1) No standard deviation can be given in this table, since only one unambiguous predicted label should be provided by our e-Bitter and this predicted label is obtained from the predicted probability averaging over all their respective constituent models*.

**Table 4:** The comparison of models evaluated on the “Phytochemical Dictionary” dataset (49 bitterants and 26 non-bitterants).

**Program**	**Model**	**TP**	**TN**	**FP**	**FN**	**Accuracy (test set)**	**Precision (test set)**	**Specificity (test set)**	**Sensitivity (test set)**	**F1-score (test set)**	**MCC (test set)**
e-Bitter	CM01	48	20	6	1	0.907	0.889	0.769	0.980	0.932	0.794
	CM02	47	20	6	2	0.893	0.887	0.769	0.959	0.922	0.761
	CM03	48	20	6	1	0.907	0.889	0.769	0.980	0.932	0.794
	CM04	46	21	5	3	0.893	0.902	0.808	0.939	0.920	0.762
	CM05	48	20	6	1	0.907	0.889	0.769	0.980	0.932	0.794
	CM06	46	20	6	3	0.880	0.885	0.769	0.939	0.911	0.731
	CM07	46	18	8	3	0.853	0.852	0.692	0.939	0.893	0.669
	CM08	48	19	7	1	0.893	0.873	0.731	0.980	0.923	0.764
	CM09	48	21	5	1	0.920	0.906	0.808	0.980	0.941	0.823
BitterX	SVM	46	8	18	3	0.720	0.719	0.308	0.939	0.814	0.332
BitterPredict	Adaboost	48	18	8	1	0.880	0.857	0.692	0.980	0.914	0.735

*(1) No standard deviation can be given in this table, since only one unambiguous predicted label should be provided by our e-Bitter and this predicted label is obtained from the predicted probability averaging over all their respective constituent models*.

### Derivation of feature importance and feature partial derivative of ECFP bit

The feature importance of ECFP bit is also of particular interest for us. In this work, feature importance of each ECFP bit is derived from the random forest (RF) after the five-fold cross-validation as mentioned above, and feature importance will be automatically linked to the corresponding fingerprint bit “1” and structural feature in our e-Bitter program. However, the feature importance from RF can only tell us whether these features are vital to the bitter/bitterless classification, but cannot inform us whether each ECFP bit “1” in a compound positively or negatively influences the bitterness, which can be described by the concept “feature partial derivative.” (Hasegawa et al., 2010; Marcou et al., 2012; Polishchuk, 2017).

(8) $$\begin{array}{r} w_{i}=\partial_{i}f\left(x\right)=\frac{\partial f}{\partial x}\left(x_{1},x_{2},\ldots ,x_{k}\right) \end{array}$$

Where *k* is the length of ECFP fingerprint; *i* means the *ith* fingerprint bit of interest, which is ranging from 1 to *k*; *x* is the value of fingerprint bit (1 or 0); *w*_*i*_ is the feature partial derivative, which actually belongs to the sensitivity analysis.

The feature partial derivative, exactly defined by Equation (8) and Hasegawa et al. (2010) is firstly proposed in the work of Byvatov and Schneider (2004) and is systematically reviewed in the work of Polishchuk (2017). To derive the feature partial derivative of each ECFP bit, the backward finite difference approach is adopted and briefly described as follows (Hasegawa et al., 2010). Firstly, the fingerprint bit nullification is simply done by the replacement of the bit “1” with zero, and then the difference between the predicted probabilities, from the original prediction and the new prediction after the bit nullification, is defined as the feature partial derivative for this fingerprint bit. If the feature partial derivative for one bit is positive, it means that this bit “1” is important to the bitterness of this compound in the positive manner, otherwise, this bit negatively affect the bitterness of this compound. This procedure is repeated for each bit “1” in a compound, thus the feature partial derivative of each ECFP bit in the compound can be derived, which can be done automatically in the e-Bitter program.

### Implementation of e-Bitter program

In order to automate the whole process, e-Bitter is developed for the convenience of users. In the current implementation, there are two basic parts. One is the generation and visualization of ECFP fingerprint, which is natively implemented in the e-Bitter program; the other is the underlying model-prediction with the diverse machine-learning approaches via evoking the external Scikit-learn, Keras, and TensorFlow python libraries natively integrated in the Winpython v3.5.4.0. For the sake of seamless fusion between these two parts, various python scripts have been implemented and integrated in the e-Bitter program.

Currently, there are three main functions in this program. (1) Predict the bitterant after loading the molecule into e-Bitter program. Batch function is also developed to screen the bitter compounds against the small-molecule database. (2) Visualize the fingerprint bit in the context of 3D structure, view the feature importance of fingerprint bit contributing to the bitterant/non-bitterant classification and look over the feature partial derivative of fingerprint bit reflecting the negative or positive influence on the bitterness. (3) Check whether the compound of users' interests is located within the applicability domain of our models.

## Results and discussion

### Fully experimental dataset used in this work

Our dataset contains the experimentally confirmed 707 bitterants and 592 non-bitterants. The bitterless dataset is composed of the experimentally validated 132 tasteless compounds, 17 non-bitter compounds and 443 sweeteners, which is different from the fully hypothetical non-bitterants employed in the works of Rodgers et al. (2006) and is also distinct from the partially hypothetical non-bitterants used in the work of Dagan-Wiener et al. (2017) and Huang et al. (2016) More specifically, Huang et al. adopt 20 experimental non-bitterants and 519 hypothetical non-bitterants, and Wiener et al. treat the 1,360 hypothetically non-bitter flavors and the experimentally validated tasteless, non-bitter and sweet compounds (557 compounds) as the non-bitterants (1,917 compounds), while our work only conservatively considers the experimentally confirmed tasteless, sweet and non-bitter molecules as the non-bitterants (592 compounds).

In the work of Wiener et al. 1,360 hypothetically non-bitter flavor compounds are assumed as the non-bitterants for the bitter/bitterless classification (Dagan-Wiener et al., 2017). However, some of hypothetically non-bitter flavors are small and volatile odorous molecules, which are modulated by about 400 olfactory receptors to give rise to the sense of smell (Hauser et al., 2017). As we know, the olfactory receptors, belonging to the class A family of GPCRs, possess the binding pocket in the TMD domain (Sayako et al., 2008), meanwhile, 25 *h*Tas1Rs, arguably categorized as class A family of GPCRs, have the binding site in the TMD domain as well (Di Pizio et al., 2016). Hence some of these small odorants may still have chances to promiscuously activate *h*Tas2Rs to elicit the bitterness. Hence, caution should be taken in the use of hypothetically non-bitter flavors.

Moreover, the preliminary bitter/bitterless classification model in the work of Wiener et al., which is trained on the dataset including the non-bitter flavors and 2,000 diverse molecules selected from ChEMBL database (Gaulton et al., 2012) as the hypothetical non-bitterants, is probably not very promising, since there is no detailed performance evaluation about this preliminary model mentioned in their work, and actually these 2,000 compounds from ChEMBL database are excluded in the model-training to achieve their final best classification model (Dagan-Wiener et al., 2017). Therefore, our work does not take account of the hypothetical non-bitterants in our dataset.

### The chemical space of our bitterants/non-bitterants dataset

To examine whether the chemical space differs obviously between the bitterants and non-bitterants, the histograms of the molecular weight (MW), logP, the number of hydrogen-bond donor (N_HBD_) and acceptor (N_HBA_) for this dataset are shown in Figures S1–S4 and analyzed as follows. For logP and MW (Figures S1, S2), the overall distributions for the bitterants and non-bitterants are very similar, except the height of peak. In the histogram of logP, the peak for the bitterants is a bit sharper, which indicates that more bitterants tends to be hydrophobic, while in the histogram of MW, the peak for bitterants is a little flat. Moreover, the scatter plot of logP vs. MW (Figure 1A) also illustrate that there is no apparent separation between the bitterants and non-bitterants from this perspective. For the bitterants, the scatter plot of logP vs. MW is also very close to the counterpart in the work of Wiener et al., where they proposed the bitter domain defined by the relations as follows: −3 = < logP ≤ 7 and MW ≤ 700 (Dagan-Wiener et al., 2017).

Moreover, N_HBD_ for the bitterants is predominantly ranging from 0 to 2 (Figure S3), and N_HBA_ for the non-bitterants mainly varies from 1 to 3 (Figure S4). Similarly, the distributions of N_HBA_ for the bitterants and non-bitterants have peaks at 2 and 4 respectively. Thus, the comparisons of N_HBD_ or N_HBA_ imply that the bitterants are slightly less hydrophilic than the non-bitterants. In addition, the scatter plots of N_HBA_ vs. N_HBD_ show that the distributions for the bitterants and non-bitterants cannot be easily distinguished from this view (Figure 1B). Therefore, it seems that logP, MW, N_HBD_, and N_HBA_ are not good to intuitively discriminate the bitterants and non-bitterants.

Furthermore, ECFP based similarity matrix (Figure 2) clearly shows the overall Tanimoto similarities between the bitterants and non-bitterants are quite low with the average value (0.0694) over the whole matrix. This provide an important clue that ECFP fingerprint may be a good molecular descriptor for our bitterants and non-bitterants classification. Thus in our work, various ECFP fingerprints are implemented and explored.

### Comparison of models trained with the different ECFPs and without the feature selection

ECFP fingerprints are extensively adopted as the molecular descriptors for the machine-learning based QSAR or QSPR study (Ekins et al., 2010; Hu et al., 2012; Braga et al., 2015, 2017; Koutsoukas et al., 2016; Varsou et al., 2017; Wang et al., 2017; Yang et al., 2017). However, it has not been adopted to predict the bitterant in the existing literature. In most of the previous works about the classification, 1024bit-ECFP4 or 2048bit-ECFP6 are often chosen by default. In this work, 1024bit-ECFP4, 2048bit-ECFP4, 1024bit-ECFP6, and 2048bit-ECFP6 are systematically explored, since ECFP6 possesses more structural features than ECFP4, and 2,048 bits can accommodate more structural features than 1024bits to alleviate the bit collision. Thus in this section, the trained models without the prior feature selection are used for the comparison.

In order to statistically compare the overall performance of those four ECFP fingerprints in the context of each machine-learning method, two-sample *T*-test is conducted based on F1-scores from two sets of nineteen random data-splitting schemes, and is systematically performed for each pair (Table 5 and Tables S14–S16) except the model-training with DNN, since only three random data-splitting are done for DNN due to its demanding computational time and are not enough to perform *T*-test for the limited sample size. Thus in this section, only KNN, SVM, GBM, and RF are statistically compared with the *p*-value from two-sample *T*-test. For KNN, Table 5 shows that the average F1-scores for 1024bit-ECFP4 (AM01), 2048bit-ECFP4 (AM07), 1024bit-ECFP6 (AM13), and 2048bit-ECFP6 (AM19) are 0.898, 0.900, 0.898, and 0.898 respectively, and there are no significant differences among two ECFPs according to the criterion (*p*-value < 0.0001). Thus, different ECFPs won't statistically influence the F1-score for KNN with full features. Based on the Tables S14–S16, this conclusion is also hold for SVM, GBM, and RF from the statistical perspective.

**Table 5:** Comparisons of average models (AM) with the different ECFPs, but with the same KNN method and full features.

**Name of average model**	**1024bit-ECFP4 [AM01, F1 = 0.898(0.018)]**	**2048bit-ECFP4 [AM07, F1 = 0.900(0.014)]**	**1024bit-ECFP6 [AM13, F1 = 0.898(0.016)]**	**2048bit-ECFP6 [AM19, F1 = 0.898(0.017)]**
1024bit-ECFP4 [AM01, F1 = 0.898(0.018)]	–	3.3221497380e-01(N)	9.7217732030e-01(N)	9.6728090414e-01(N)
2048bit-ECFP4 [AM07, F1 = 0.900(0.014)]	3.3221497380e-01(N)	–	2.1534925183e-01(N)	4.9376183004e-01(N)
1024bit-ECFP6 [AM13, F1 = 0.898(0.016)]	9.7217732030e-01(N)	2.1534925183e-01(N)	–	9.8674211132e-01(N)
2048bit-ECFP6 [AM19, F1 = 0.898(0.017)]	9.6728090414e-01(N)	4.9376183004e-01(N)	9.8674211132e-01(N)	–

*(1) “N” means that there is no significant difference between two models according to the criterion (p-value < 0.0001); (2) the number in the element of matrix is the p-value after two-sample T-test for every two average models. (3) “–” indicates that two-sample T-test between the same average models will be ignored*.

In short, different ECFPs won't statistically affect the performance of our bitterant/non-bitterant classification in the context of the same machine-learning method with the full features. From this view, the default choice of 1024bit-ECFP4 or 2048bit-ECFP6 is generally acceptable in the bitterant/non-bitterant classification. In our case, four ECFPs are still adopted, since their combination with the feature selection, multiple machine-learning methods and consensus strategy may offer a better solution, which will be discussed as follows.

### Comparison of models trained with and without the prior feature selection

Feature selection is widely employed to choose a subset of features for the model training in the machine-learning studies. In this work, the top 512, 256, and 128 important bits of ECFPs are selected to build the models for the comparison with their counterparts, which are trained with the full features.

The impact of feature selection on the model performance will be assessed in the context of specific combination of ECFPs and machine-learning methods. To compare the model performance in the statistical manner, two-sample *T*-test is also conducted based on the F1-scores from two sets of nineteen random data-splitting schemes and all the *T*-test results including the *p*-value will be reported in Table 6 and Tables S17–S31.

**Table 6:** Comparisons of average models (AM) with the different feature numbers, but with the same KNN method and 1024bit-ECFP4.

**Name of average model**	**Full features [AM01, F1 = 0.898(0.018)]**	**512 features [AM25, F1 = 0.911(0.012)]**	**256 features [AM49, F1 = 0.920(0.016)]**	**128 features [AM73, F1 = 0.910(0.013)]**
Full features [AM01, F1 = 0.898(0.018)]	–	4.9645296142e-05(Y)	3.6331197284e-05(Y)	3.1292342028e-03(N)
512 features [AM25, F1 = 0.911(0.012)]	4.9645296142e-05(Y)	–	2.1671323186e-02(N)	7.8500884020e-01(N)
256 features [AM49, F1 = 0.920(0.016)]	3.6331197284e-05(Y)	2.1671323186e-02(N)	–	7.9667376708e-03(N)
128 features [AM73, F1 = 0.910(0.013)]	3.1292342028e-03(N)	7.8500884020e-01(N)	7.9667376708e-03(N)	–

*(1) “N” means that there is no significant difference between two models according to the criterion (p-value < 0.0001), while “Y” suggests that there is significant difference between two models according to the criterion (p-value < 0.0001); (2) the number in the element of matrix is the p-value after two-sample T-test for every two average models. (3) “–” indicates that two-sample T-test between the same average models will be ignored*.

Feature selection consistently affects the performance of model trained with KNN and four ECFPs (Table 6 and Tables S17–S19). Here KNN method with 1024bit-ECFP4 is taken as an example (Table 6). The models trained with 512 features (AM25, F1 = 0.911) or 256 features (AM49, F1 = 0.920) manifest the significantly different performance compared to its counterpart trained with full features (AM01, F1 = 0.898) according to the criterion (*p*-value < 0.0001). Based on the average F1-score, in this case feature number 512 or 256 after feature selection is better than the full features. However, it is not always true that feature selection will be helpful to the KNN with 1024bit-ECFP4. The models trained with 128 features (AM73, F1 = 0.910) and full features (AM01, F1 = 0.898) have the similar performance, since two-sample *T*-test illustrates that there are no significant difference of performance between these two models, since the *p*-value is larger than 0.0001. Thus, feature selection will improve the performance of KNN method depending on the specific feature number. KNN method with the other ECFPs (Tables S17–S19) gives the same conclusion.

However, for the models trained the SVM method and ECFPs (Tables S20–S24), feature selection won't influence the performance relative to the full features, since there is no significant difference among these models according to the criterion (*p*-value < 0.0001). However, it is worth mentioning that the model trained with 128 features (AM92, F1 = 0.905) has significant difference of F1-score compared with the counterpart trained with 512 features (AM44, F1 = 0.921) based on the criterion (*p*-value < 0.0001).

Similarly, feature selection has no effect on the RF and GBM methods combined with any ECFP (Tables S25–S31), since no significant differences of F1-score are observed based on the criterion (*p*-value < 0.0001). This is expected for RF and GBM methods, since they have their own intrinsic capabilities to select the important features, hence RF and GBM methods are not very sensitive to the prior feature selection, which is consistent with the finding in the work of Zang et al. (2017).

Therefore, relative to the full features, 256 or 512 features will be good for the KNN method with ECFPs, while feature selection has no impact on SVM, GBM and RF methods in our cases from the statistical analysis. It should be noted that DNN2 and DNN3 are excluded in our two-sample *T*-test due to very limited samples, thus the effect of the feature selection on DNN2 and DNN3 is not discussed.

### Overall performance of all the individual models and average models

In this work, 1,312 models have been harvested considering the combination of different ECFPs, machine-learning methods, feature selection, and random data-splitting schemes. To reduce the bias from different random data-splitting schemes, 96 average models (**AM**) are obtained from 1,312 models by averaging over different data-splitting schemes.

**Figure 7:**
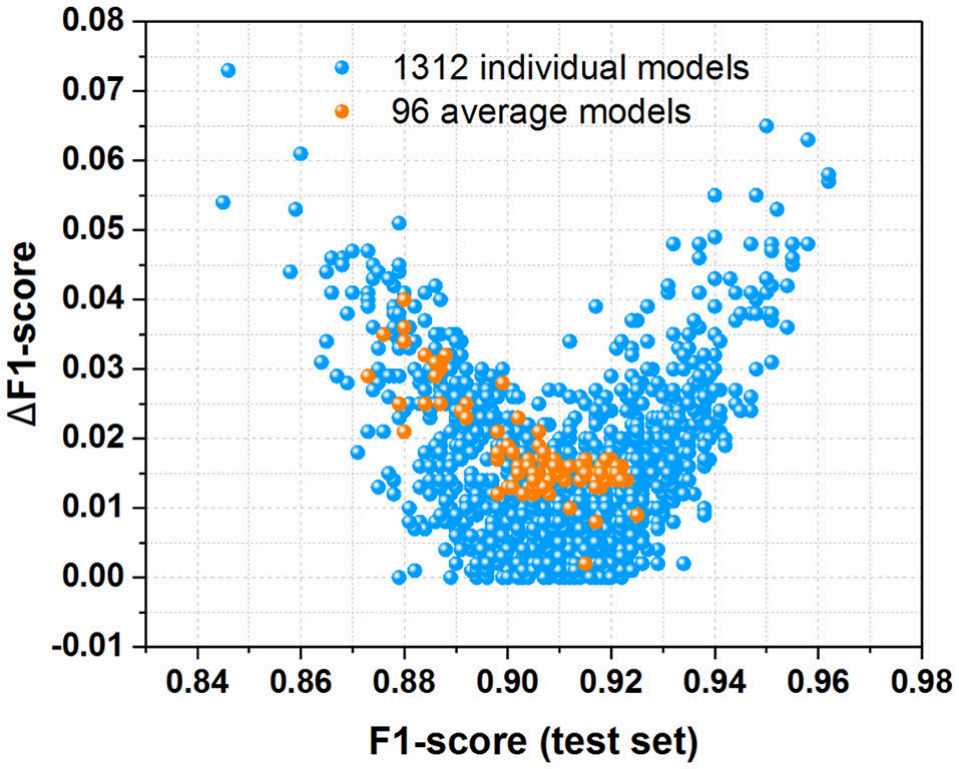
The scatter plot of ΔF1-score vs. F1-score (test set) for all the 1,312 individual models and 96 average models derived from this work.

To evaluate the performance of all 1,312 models and 96 average models, both F1-score and ΔF1-score are calculated from Tables S1, S2, which are displayed in Figure 7. F1-score is the key criterion used to select the best model during the modeling training with cross-validation, while ΔF1-score used to inspect the possible overfitting or underfitting of model is obtained with Equation (7). The scatter plot of ΔF1-score vs. F1-score (test set) demonstrates that all the average models and most of individual models have the ΔF1-score lower than 0.04, indicating that the performance on the test set and in the cross-validation is quite similar. Hence all these average models and most of individual models are probably robust without the apparent overfitting or underfitting from this perspective.

In addition, majorities of the average models and individual models are located in the bottom-right corner of the scatter plot (Figure 7), suggesting that most of them have high F1-score larger than 0.90. Moreover, MCC (test set) vs. F1-score (test set) for all the 1,312 individual models and 96 average models (Figure S12) indicates that most of these models are quite good from the perspective of MCC and F1-score. Therefore, most of our average models and individual models exhibit the promising performance.

### Y-randomization test of our models

To examine the reliability of all the 1,312 models, Y-randomization test is conducted by the random shuffling of the experimentally-observed labels, which generates a noisy dataset (Table S3). Before the model-training, the accuracy, precision, specificity, sensitivity, F1-score, and MCC of this noisy dataset are 47.5~53.3%, 51.8~57.1%, 42.4~48.7%, 51.8~57.1% 0.518~0.571, and −0.058~0.058 respectively (Table S3), which indicates that almost half of samples are still correct in this noisy dataset. Taken the randomized **exp01** (Table S3) as an example, the respective numbers for the true bitterants (TP) and true non-bitterants (TN) are 315 and 223, while the respective numbers for the false bitterants (FP) and false non-bitterants (FN) are 251 and 251 due to the random shuffling (Table S3). However, the ratio between bitterants (TP and FP) and non-bitterants (TN and FN) is still 566:474, which is identical with its counterpart in the original **Dataset-CV** before the shuffling.

After training on these noisy datasets via the cross-validation, the models with the best performance (highest F1-score) on the internal validation dataset are also assessed on the test set, which is not transformed by any random shuffling for the labels. According to Figure S13, the F1-score and MCC of the models (blue spheres) in the Y-randomization test is strikingly decreased relative to the original models (red spheres), which suggests that our original models are quite reliable. Meanwhile, the MCC of all these models in the Y-randomization test are close to 0, indicating that these models in the Y-randomization test have no better than the random prediction. Hence the Y-randomization test substantiates the reliability of the original models.

### Applicability domain assessment of our models

Applicability domain of models are defined quantitatively by the average-similarity between the given test compound and its five nearest neighbors in **Dataset-CV**. If the given test compound is close to its neighbors with a larger average-similarity, it means that the chemical space of this given compound is covered by **Dataset-CV**, thus the prediction is probably interpolated from **Dataset-CV**, which gives more reliable estimation. According to the average-similarity histograms (Figure S11) of **Dataset-CV** and **Dataset-Test**, the compounds in the test set (**Dataset-Test**) are fully covered by the dataset for the cross-validation (**Dataset-CV**), thus the given compound with average-similarity higher than 0.1 is assumed to be within the applicability domain of our models according to Figure S11. If the compound is not located within the applicability domain of our models, the prediction for this compound is probably extrapolated from **Dataset-CV** and consequently is not confident.

### Performance comparison among BitterX, BitterPredict, and e-Bitter

BitterX and BitterPredict adopt SVM and Adaboost methods respectively, while e-Bitter implicitly integrates nine consensus models (CM01-CM09) and optionally includes 96 average models (AM01-AM96). To evaluate the performance of all the models, two types of comparisons are conducted. One is the direct comparison on the test set reported in the original works. The other is the more fair comparison on the three external test sets in the recent work of Wiener et al.

For the direct comparison, F1-score (test set) and MCC (test set) of the model from BitterX are 0.918 and 0.842 respectively (Table 1), and the counterparts of the model from BitterPredict are 0.708 and 0.595 respectively (Table 1). It worth mentioning that F1-score and MCC are all calculated based on the data reported in their works. From this perspective, BitterX is much better than BitterPredict. Additionally, the model from BitterX and our models including consensus models and average models are markedly better than the counterpart from BitterPredict (Red sphere in Figure 3). Moreover, one of our consensus models (**CM1**, Blue sphere in Figure 3) is also a little better than the model from bitterX (Black sphere in Figure 3), the consensus models (**CM03**, **CM05**, and **CM07**) are comparable to the model from BitterX and the consensus models (**CM02**, **CM04**, **CM06**, **CM08**, and **CM09**) are slightly inferior to the model from BitterX. It should be noted that the model from BitterPredict is derived from only one random data-splitting scheme, and the model from BitterX is averaged over three random data-splitting schemes. Our consensus models are built by averaging over all the constituent models (19 individual models for **CM01** and 5 average models for **CM02-CM09**), while our average models (AM) in Table S2 are derived by averaging over 19 random data-splitting schemes for KNN, SVM, GBM, and RF or three random data-splitting schemes for DNN2 and DNN3. Therefore, our models in the e-Bitter may offer more robust results in this respect.

To seek the further objective evaluation, three external test sets in the recent work of Dagan-Wiener et al. (2017) are employed accordingly. For the “Bitter New” dataset with 23 bitterants, the prediction accuracies are 74, 74, and 100% for bitterX, BitterPredict, and e-Bitter respectively, while F1-scores are 0.85, 0.85, and 1.00 for bitterX, BitterPredict, and e-Bitter respectively (Table 2). Thus, all the consensus models and average models in the e-Bitter afford the best performance for this dataset (Figure 4 and Table 2).

As concerning the “UNIMI set” dataset (23 bitterants and 33 non-bitterants), bitterX obtains the accuracy, precision, specificity, sensitivity, F1-score and MCC of 60, 52, 56, 65%, 0.58 and 0.21 respectively (Table 3), which are lower than their counterparts reported in the work of Huang et al. (2016) BitterPredict offers the accuracy, precision, specificity, sensitivity, F1-score and MCC of 82, 78, 85, 78%, 0.78 and 0.63 respectively reported in the work of Dagan-Wiener et al. (2017) Meanwhile, e-Bitter gives the accuracy, precision, specificity, sensitivity, F1-score and MCC of 68~73%, 57~61%, 55~58%, 87~100%, 0.69~0.75, and 0.47~0.58 respectively considering the different consensus models (Table 3), which are also lower than their counterparts evaluated on our test set (**Dataset-Test**) in Table S2. Obviously, BitterPredict and e-Bitter (9 consensus models and 96 average models) unanimously outperform BitterX (Figure 5), while BitterPredict exhibits the slightly better performance than e-Bitter in light of F1-score and MCC (Figure 5).

Regarding to the “Phytochemical Dictionary” dataset (49 bitterants and 26 non-bitterants), BitterPredict provides the accuracy, precision, specificity, sensitivity, F1-score and MCC of 88, 86, 69, 98%, 0.91 and 0.735 respectively (Table 4, Dagan-Wiener et al., 2017) while e-Bitter predicts that the accuracy, precision, specificity, sensitivity, F1-score and MCC are 85~92%, 85~91%, 69~81%, 94~98%, 0.89~0.94, and 0.67~0.82, which is comparable with the counterparts evaluated on our test set (**Dataset-Test**) in Table S2. BitterX achieves the accuracy, precision, specificity, sensitivity, F1-score and MCC of 72, 72, 31, 94%, 0.814 and 0.332 respectively (Table 4). Thus for this dataset, all 9 consensus models and 96 average models in the e-Bitter are consistently better than BitterX (Figure 6), while most of the consensus models (**CM01**, **CM02**, **CM03**, **CM04**, **CM05**, **CM08**, and **CM09**) and some average models show more promising results than BitterPredict based on the F1-score and MCC (Figure 6).

Therefore, in light of the performance indicators F1-score and MCC, e-Bitter obtains the best performance for the “Bitter New” dataset and “Phytochemical Dictionary” dataset. e-Bitter consistently outperforms bitterX for all these three test sets. BitterPredict achieves the slightly better result than e-Bitter for the “UNIMI set” dataset. It is worth noting that in this section the performance of e-Bitter specifically refers to the performance of the consensus models, albeit the performance of 96 average models are optionally available in the e-Bitter and also discussed above.

### Interactive visualization of ECFP fingerprint bit, 3D structural feature, feature importance, and feature partial derivative

**Figure 8:**
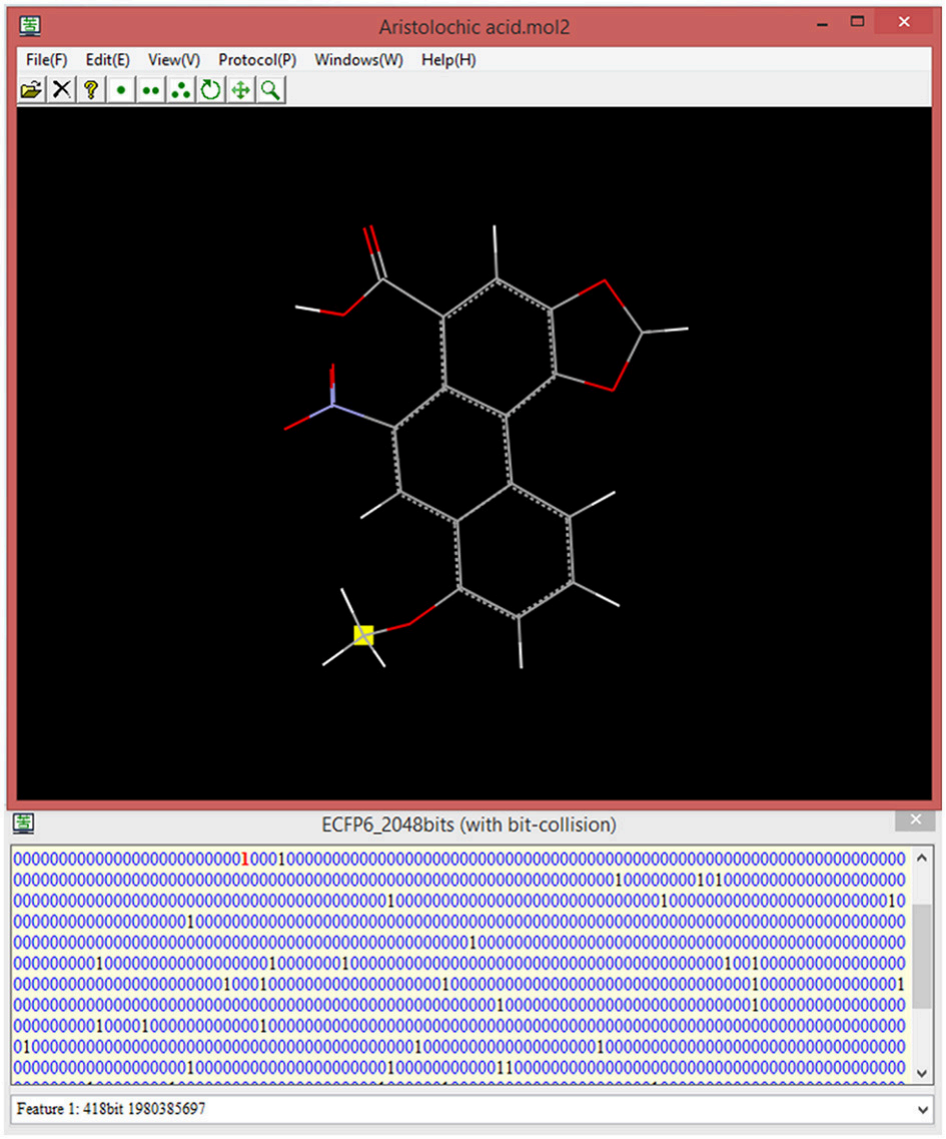
The interactive visualization of the fingerprint bit and its corresponding structural feature in the 3D viewer. The title of fingerprint window shows the type of ECFP and also displays whether there is bit-collision occurring in the fingerprint. All the structural features can be visualized by browsing the list in the combo box (bottom) even if there is bit-collision.

ECFP fingerprint is prevalently used in the chemoinformatics, however, this “0/1” bit string is quite obscure for the food scientist to correlate the specific bit “1” to its corresponding structural feature in the context of 3D structure. For this purpose, our implementation is designed to record all the structural information including atoms and bonds for each bit during the ECFP generation, and is intended to highlight the structural feature in the context of the whole 3D structure via clicking the bit “1” of interest. This unique feature of our implementation will provide an appealing advantage. In our current version, ECFP diameter and bit length can be customized by the users, albeit the commonly-used ECFP diameter is 4 or 6, and frequently-used ECFP bit length is 1,024 or 2,048. Once setup the diameter and bit length, e-Bitter program can generate the ECFP fingerprint in the fingerprint windows via simply clicking on the menu, and can automatically associate all the bit “1” with the corresponding structural features in the 3D viewer, and is ready for the users to select the bit of interest (Figure 8).

Besides the structural visualization for the fingerprint bit, the associated feature importance and feature partial derivative are also very useful information. The feature importance emphasizes the importance of each bit contributing to the bitter/bitterless classification, and the feature partial derivative of each bit stresses the positive or negative influence of each bit on the bitterness of the compound. Hence, the fingerprint bit “1,” its corresponding structural feature, feature importance, and feature partial derivative are closely interconnected and can be interactively visualized in our e-Bitter program. This interactive and synchronization function can be well depicted by Figure S14.

### Demonstration of e-Bitter program

**Figure 9:**
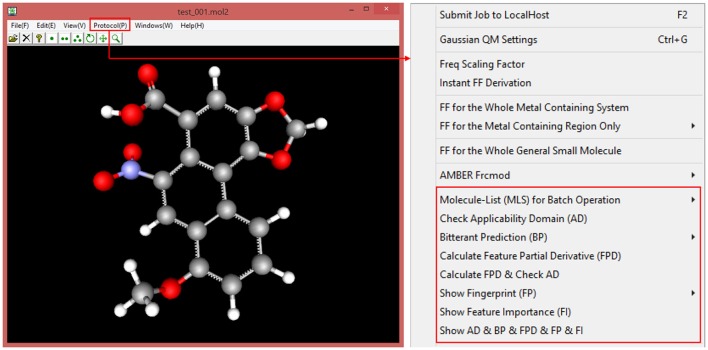
The basic functions in the e-Bitter program, which is highlighted by the red rectangle.

e-Bitter is a stand-alone package, which can be freely downloaded from Dropbox shared folder (https://www.dropbox.com/sh/3sebvza3qzmazda/AADgpCRXJtHAJzS8DK_P-q0ka?dl=0). This program is well tested on the 64bit windows operating system such as Win7, Win8, and Win10, while the external Scikit-learn, Keras and Tensorflow python libraries can be easily set up via simply installing the Winpython-64bit v3.5.4.0 that integrates the complete python environment and rich python libraries on the windows operating systems. In this sense, the whole installation process on user's computer is quite handy. Once installed and configured, e-Bitter can predict whether the molecule loaded in the 3D viewer is bitter or not, or can perform an automated virtual screening against a small-molecule database to obtain the possible bitterants candidates. Additionally, e-Bitter program also implements the job management system (Figure S15), since it would be time-consuming to conduct the prediction with the consensus models, particularly for those models including the deep neuron network methods. Figure 9 briefly recapitulates all the functions in the e-Bitter program.

Besides this graphic mode, e-Bitter program can also work in the console mode, since some users prefer to use the command line to predict the bitterants. The only input for e-Bitter program is the molecular file with the Tripos Mol2 format. The detailed example and usage are described in the tutorial and manual, which are integrated in the e-Bitter program and can also be accessed from the Dropbox shared folder.

Furthermore, all the consensus models and their constituent models integrated in our program can be easily upgraded. As we know, the dataset about the bitterants and non-bitterants will grow more and more rapidly, thus we will continue to spend the effort to constantly upgrade our models with the larger experimental dataset, and upload the new models to the folder called “model” in the Dropbox shared folder. Users can download the latest models to replace the previous ones for their prediction.

### Overall function comparison among BitterX, BitterPredict, and e-Bitter

Relative to the online tool bitterX (Huang et al., 2016), e-Bitter works on the local machines, which ensures that users can well exploit their own computational resource to test their propriety compounds without turning to the external web server. Moreover, e-Bitter can screen the small-molecule database in batch. More importantly, e-Bitter program adopts the consensus voting strategy based on the multiple machine-learning algorithms to enhance the reliability of prediction result.

Compared to the MATLAB tool BitterPredict (Dagan-Wiener et al., 2017), e-Bitter adopts the ECFP fingerprint as the molecular descriptors, which is natively implemented in our program. Thus, e-Bitter does not depend on any other commercial softwares such as Schrödinger package required in BitterPredict. In addition, this free e-Bitter program employs the versatile machine-learning algorithms and works compatibly with the free python environment, while BitterPredict is developed and runs in the commercial MATLAB environment, which will restrict its extensive tests or applications for most of the users.

### Limitation and future prospect

Admittedly, our work has some limitations. (1) From the perspective of curated data, the experimentally confirmed dataset probably still has some intrinsic noise, since in the experimental taste assessment, the trained panelists have some objective factors such as the individual gene-polymorphism of Tas2Rs, and also have some subjective factors such as the mixed tastes, which are unlikely to be clearly discerned. (2) As for the parameter optimization in the model training process, it is a daunting task to explore the complete parameters combinations, since the parameter spaces for machine-learning methods such as DNN are very large. Thus, in this work only the key parameters are tuned with the grid method. (3) Regarding to the feature selection, feature importance from the random forest method is used as the criterion to select the important features. In this work, only full features, 512, 256, and 128 features are attempted, while the other feature numbers and feature selection methods, which may provide more promising results, are not tried because of the tremendous combination of options and parameters. (4) From the view of consensus models, this strategy will introduce some extra computational-burden. First, different types of ECFP fingerprints should be generated for each compound, and then each constituent model coupled with its corresponding fingerprint affords the prediction result also for each compound, finally the results from all the constituent models will be averaged to make the final prediction. Therefore, this procedure is not extremely fast, especially for the consensus model containing the deep-neuron network. (5) From the function of e-Bitter, this program is only focused on the bitterant classification for the small molecule due to our current research priority.

In the future, we will devote to further collect the high-quality dataset and constantly update the models. Moreover, we will pay more attention to the other feature selection methods and parameter optimization schemes and will implement in the future version of e-Bitter program. Furthermore, e-Bitter program will be extended to qualitatively classify the bitter/bitterless peptide, quantitatively predict the bitterness of the bitterants, and explore the possible target information of the bitterants.

## Conclusions

In this work, it is the first time that the fully experimental bitterants/non-bitterants dataset, consensus voting based on the multiple machine-learning algorithms, and ECFP fingerprints are adopted to build the bitter/bitterless classification models. Through the exhaustive parameter exploration with the five-fold cross-validation, all the models are carefully scrutinized by the Y-randomization test to ensure their reliability, and subsequently nine consensus models are constructed based on the individual or average models, which differ in term of accuracy, speed and diversity of models. Evaluation on the three external test set from Wiener et al. demonstrates that e-Bitter outperforms bitterX on these three test sets; e-Bitter harvests the better results than BitterPredict for the “Bitter New” and “Phytochemical Dictionary” dataset; BitterPredict demonstrates slightly better performance than e-Bitter for the “UNIMI set” dataset. To automate the whole process, we develop a graphic e-Bitter program for the bitterant prediction or screening against the small-molecule database in batch. Additionally, e-Bitter program natively implements ECFP fingerprint, and more importantly, e-Bitter can vividly visualize the structural feature, feature importance, and feature partial derivative for any specific bit “1” in the ECFP fingerprint. We hope our work can provide a useful tool for the experimental scientist to rationally design and screen the bitterants.

## Author contributions

SZ wrote the manuscript. SZ and MJ performed the calculations. MJ, CZ, RZ, and ZH curated the dataset and test the program. SZ and MJ are contributed equally. YX and FL revised the manuscript. SZ and FL conceived the workflow and developed the program.

### Conflict of interest statement

The authors declare that the research was conducted in the absence of any commercial or financial relationships that could be construed as a potential conflict of interest.
