# Supplementary material for: e-Bitter: Bitterant Prediction by the Consensus Voting From the Machine-Learning Methods
Source: Front Chem. 2018 Mar 29;6:82. doi: 10.3389/fchem.2018.00082 (PMC5885771; doi:10.3389/fchem.2018.00082)
Supplement: Supplementary file 1 [file Table1.docx]

**e-Bitter: Bitterant Prediction by the Consensus Voting From the Machine-learning Methods**

**Suqing Zheng**^1,2*#^**, Mengying Jiang**^1#^**, Chengwei Zhao**^1^**, Rui Zhu**^1^**, Zhicheng Hu**^1^**, Yong Xu**^3^**, and Fu Lin**^1^*

*^1^: School of Pharmaceutical Sciences, Wenzhou Medical University, Wenzhou, Zhejiang, P. R. China, 325035*

*^2^: Chemical Biology Research Center, Wenzhou Medical University, Wenzhou, Zhejiang, P. R. China, 325035*

*^3^: Center of Chemical Biology, Guangzhou Institutes of Biomedicine and Health, Chinese Academy of Sciences, Guangzhou, Guangdong, P. R. China, 510530*

Correspondence to: Fu Lin (E-mail: [*lin1449@126.com*](mailto:lin1449@126.com)); Suqing Zheng (E-mail: [zsq_2016@126.com](mailto:zsq_2016@126.com))

| **** |
| --- |
| **Figure S1.** The histogram of logP. |

| **** |
| --- |
| **Figure S2.** The histogram of MW (molecular weight). |

| **** |
| --- |
| **Figure S3.** The histogram of N_HBD_ (the number of hydrogen-bond donor) |

| **** |
| --- |
| **Figure S4.** The histogram of N_HBA_ (the number of hydrogen-bond acceptor) |

| **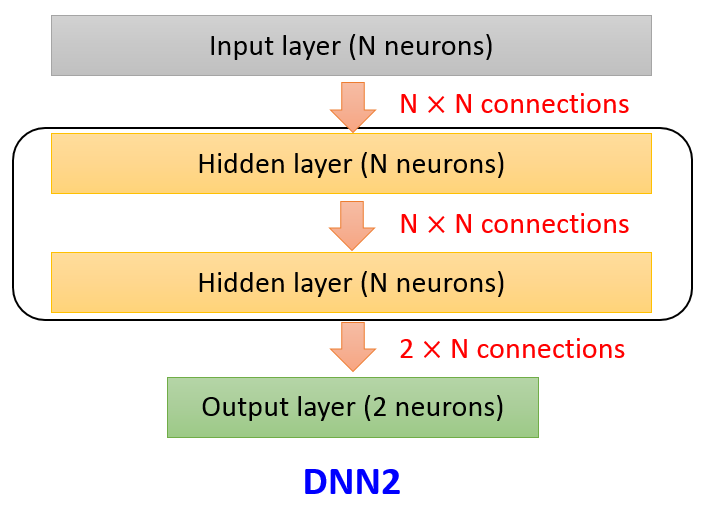** |
| --- |
| **Figure S5.** The configuration of DNN2 with two hidden layers. N in the figure refers to the number neurons and is set to the feature number in this work. |

| **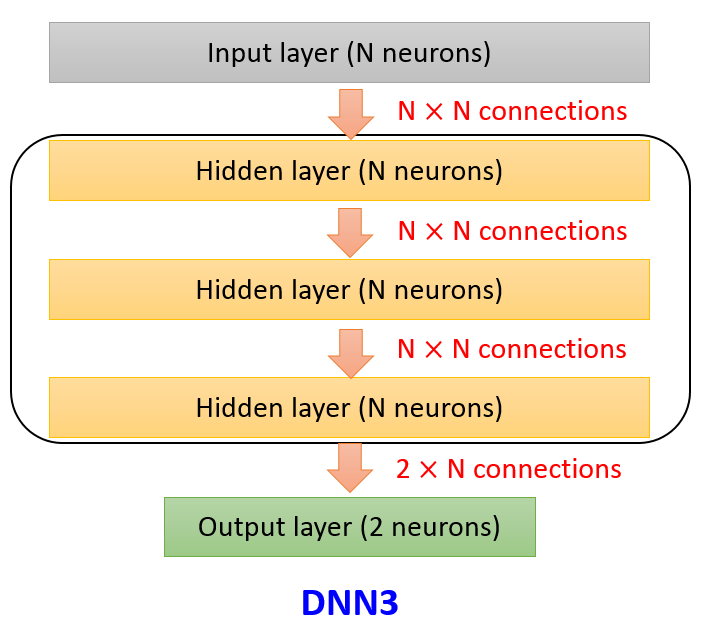** |
| --- |
| **Figure S6.** The configuration of DNN3 with two hidden layers. N in the figure refers to the number neurons and is set to the feature number in this work. |

| **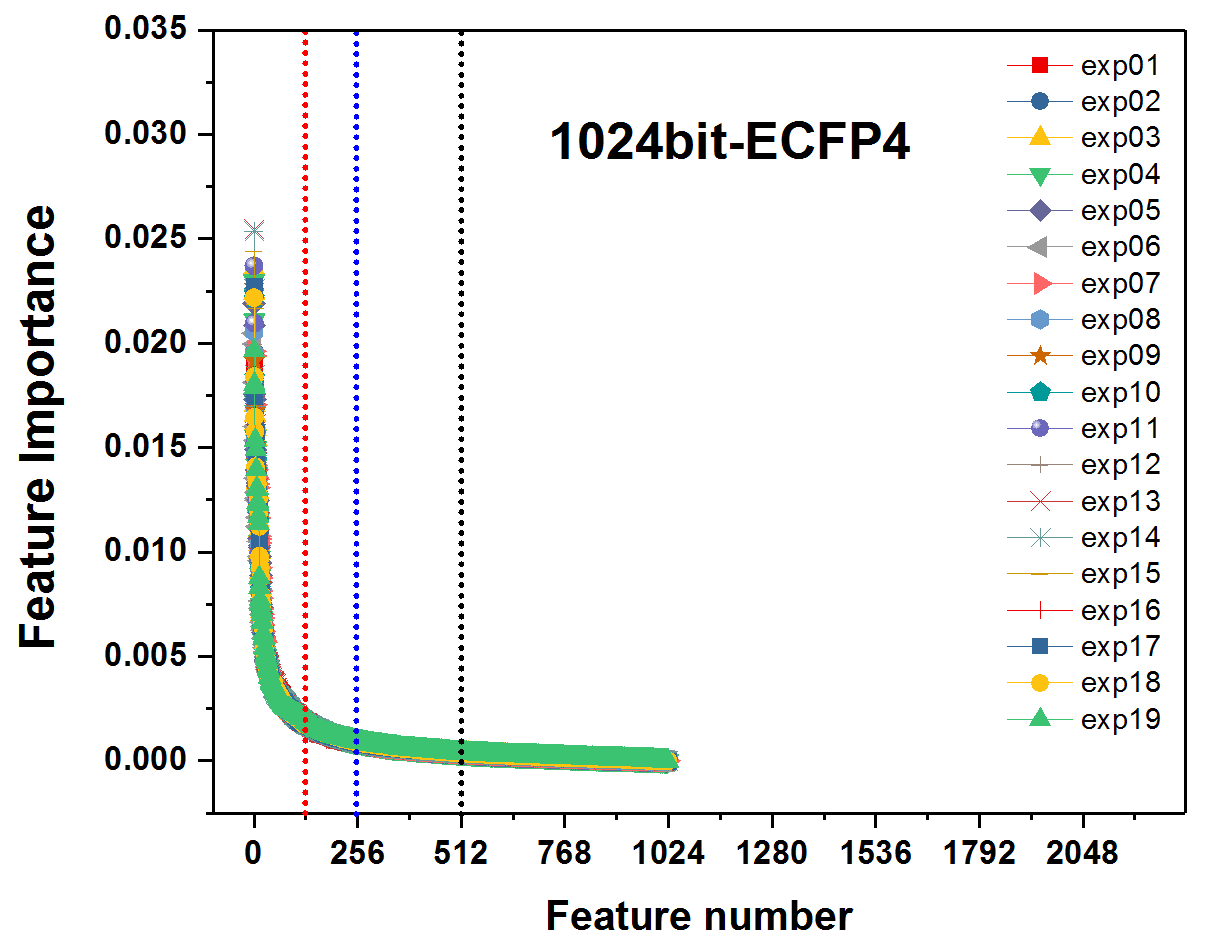** |
| --- |
| **Figure S7.** Nineteen sets of the feature importance derived from the random forest method with 1024bit-ECFP4. |

| **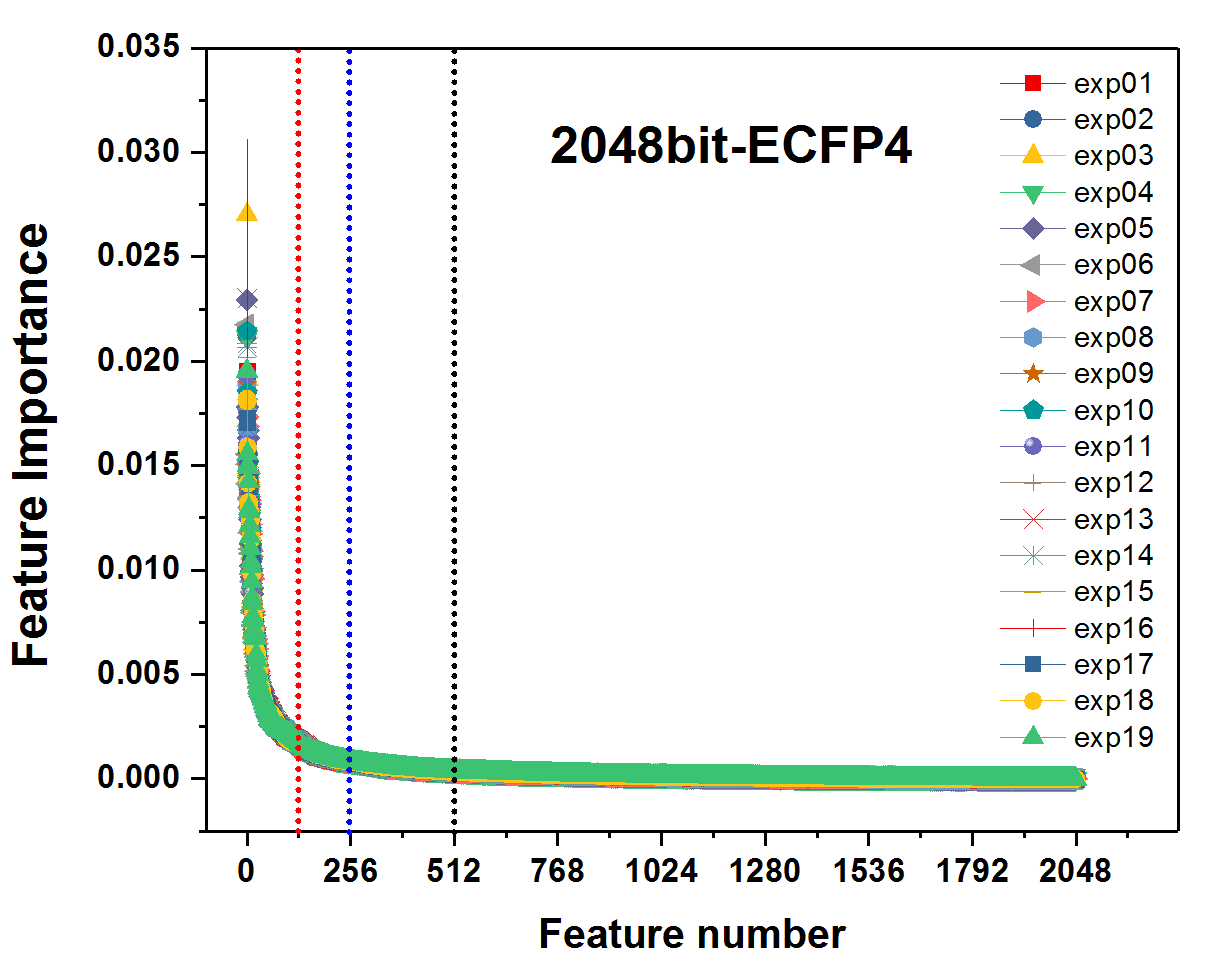** |
| --- |
| **Figure S8.** Nineteen sets of the feature importance derived from the random forest method with 2048bit-ECFP4. |

| **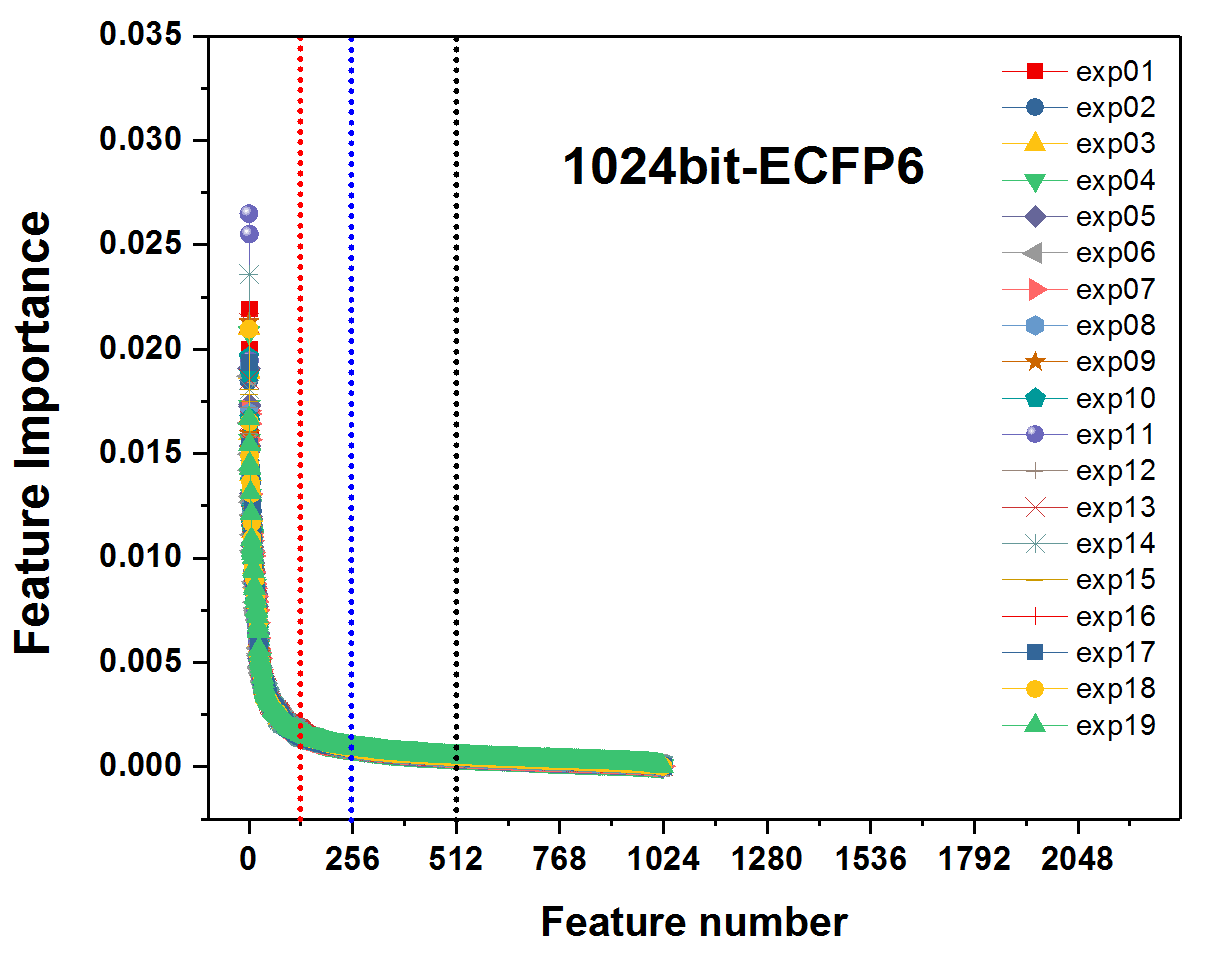** |
| --- |
| **Figure S9.** Nineteen sets of the feature importance derived from the random forest method with 1024bit-ECFP6. |

| **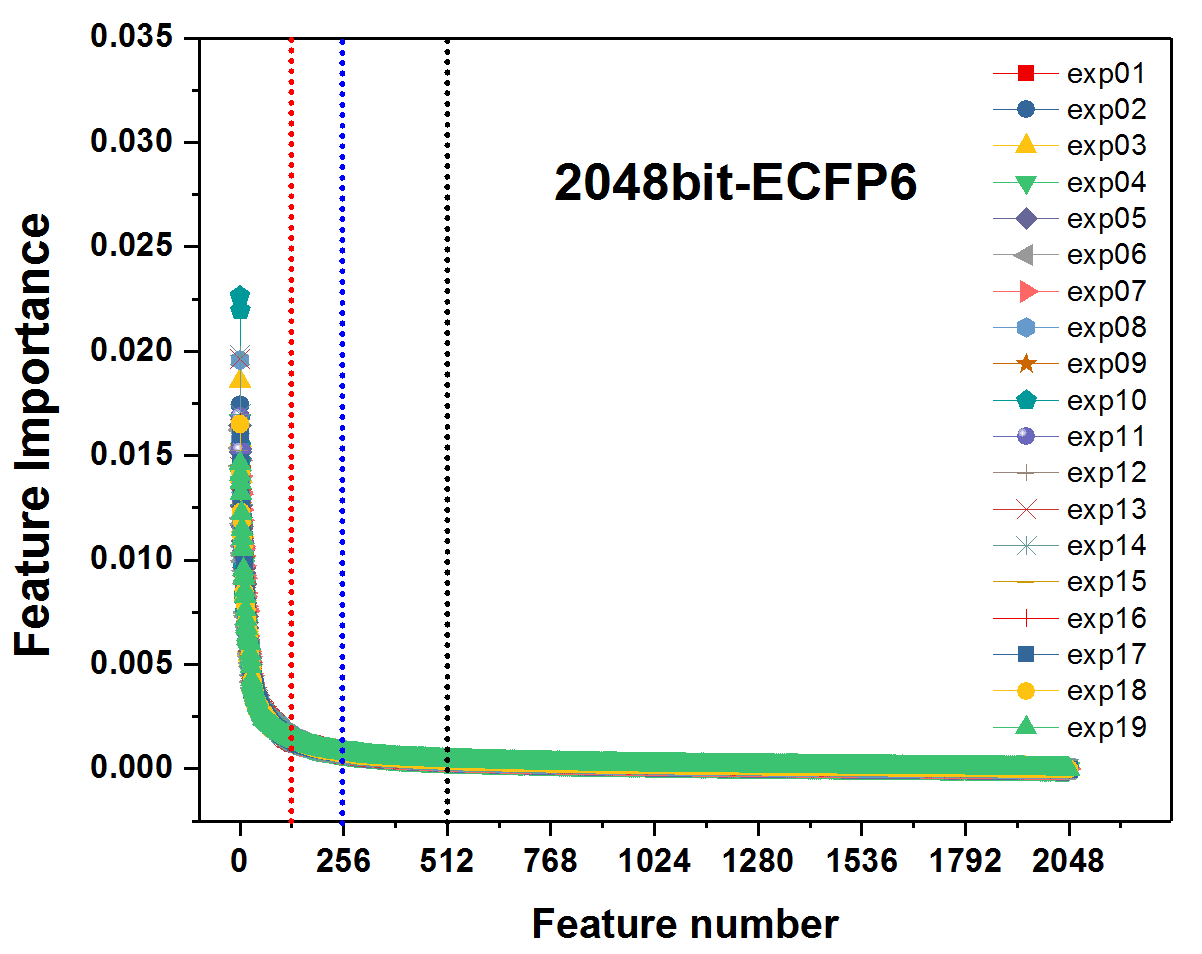** |
| --- |
| **Figure S10.** Nineteen sets of the feature importance derived from the random forest method with 2048bit-ECFP6. |

| 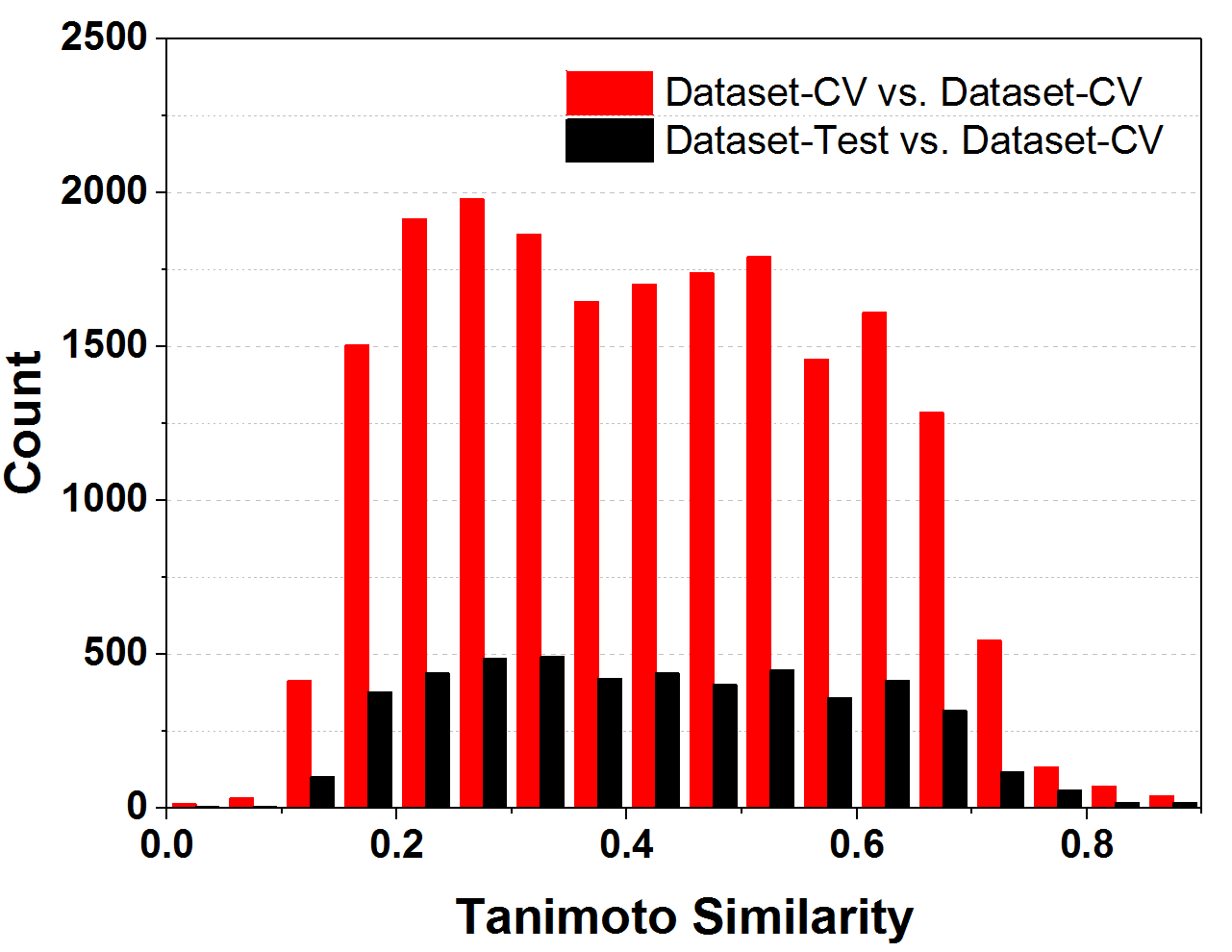 |
| --- |
| **Figure S11.** The histograms of average-similarity for **Dataset-Test** and **Dataset-CV** considering nineteen different random data-splitting schemes. |

| 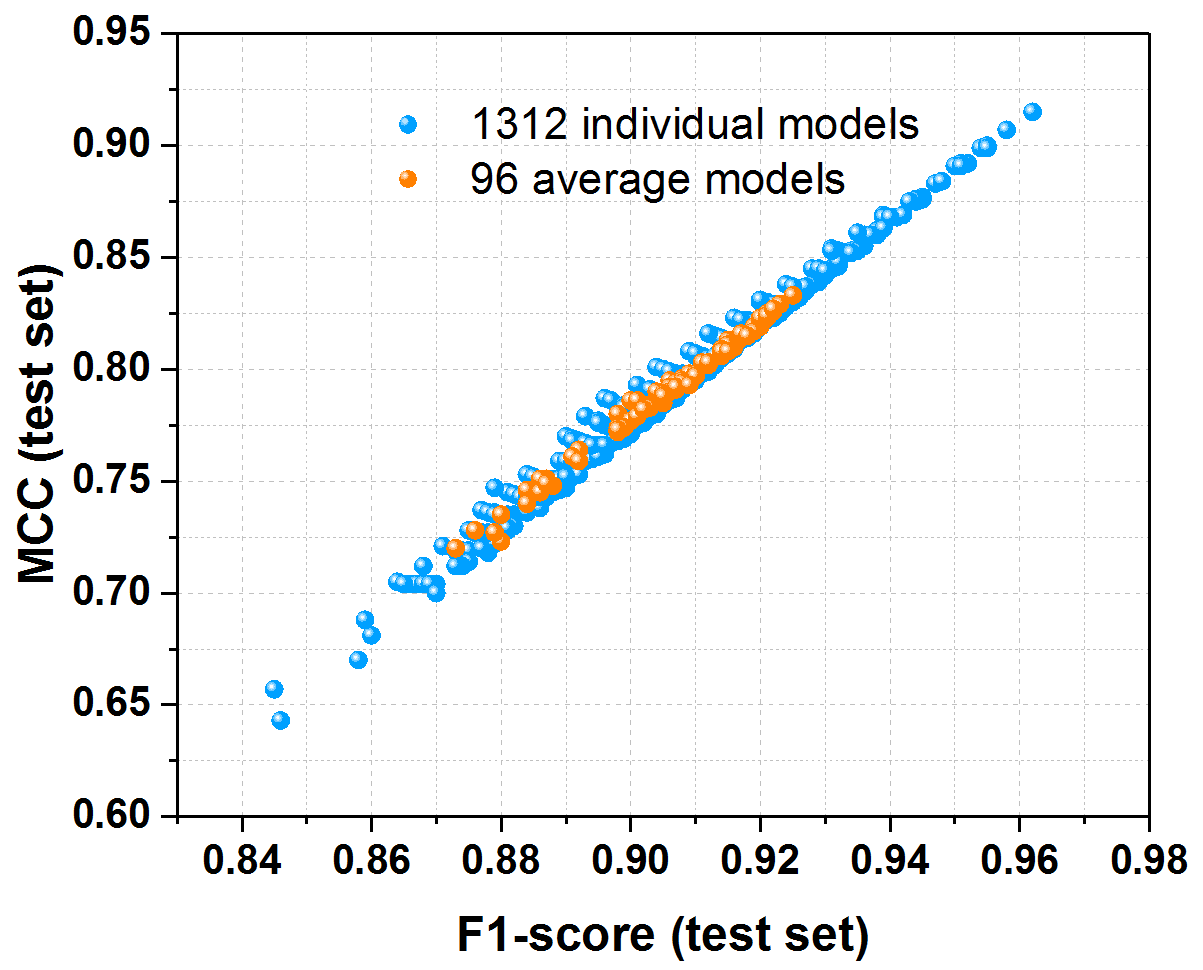 |
| --- |
| **Figure S12.** The scatter plot of MCC (test set) vs. F1-score (test set) for all the 1312 individual models and 96 average models derived from this work. |

| 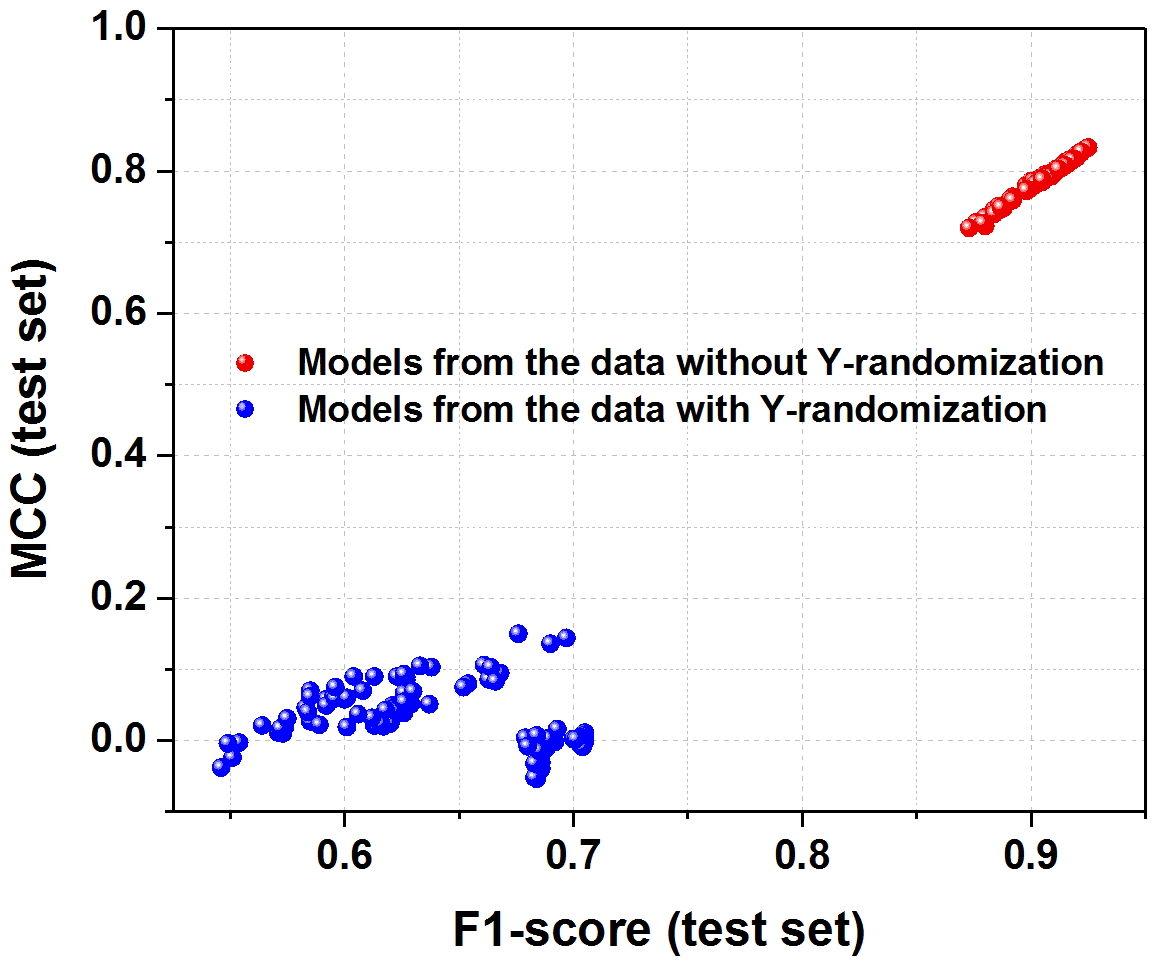 |
| --- |
| **Figure S13.** The scatter plot of MCC (test set) vs. F1-score (test set) for all the 96 average models derived from the data with and without Y-randomization. |

| 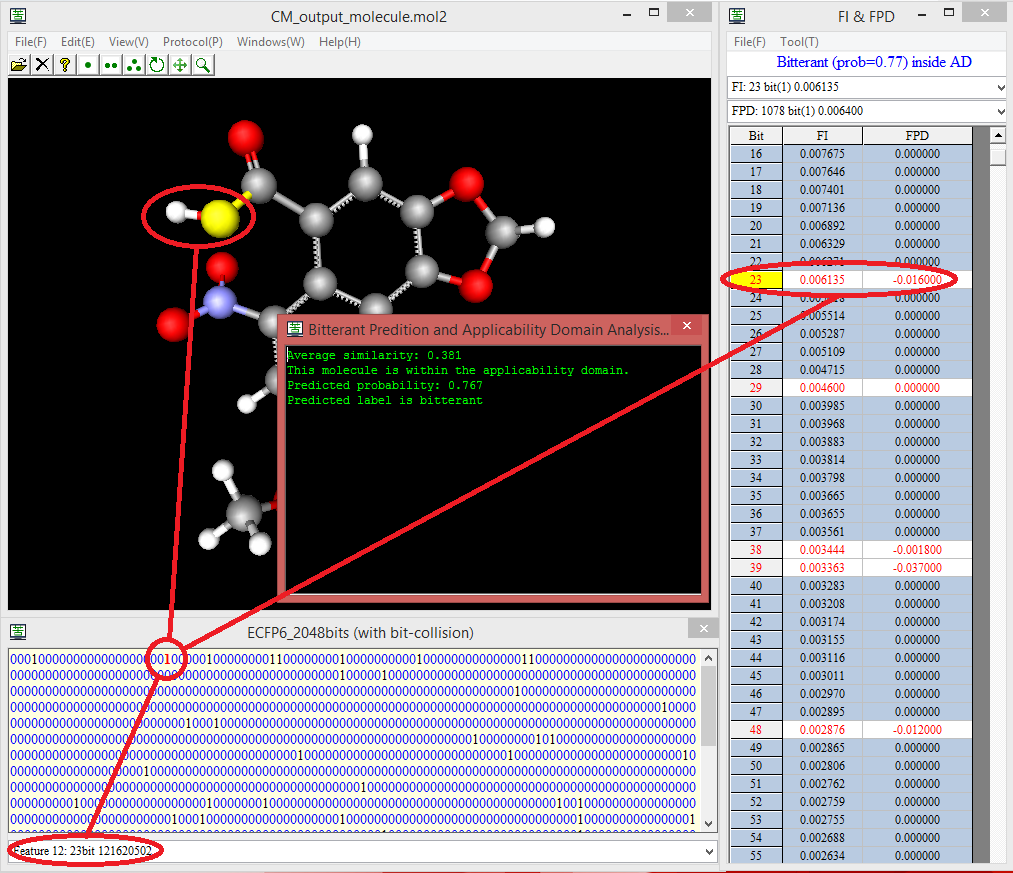 |
| --- |
| **Figure S14.** The interactive visualization of fingerprint bit “1”, its corresponding structural feature, feature importance, and feature partial derivative in our e-Bitter program. |

| 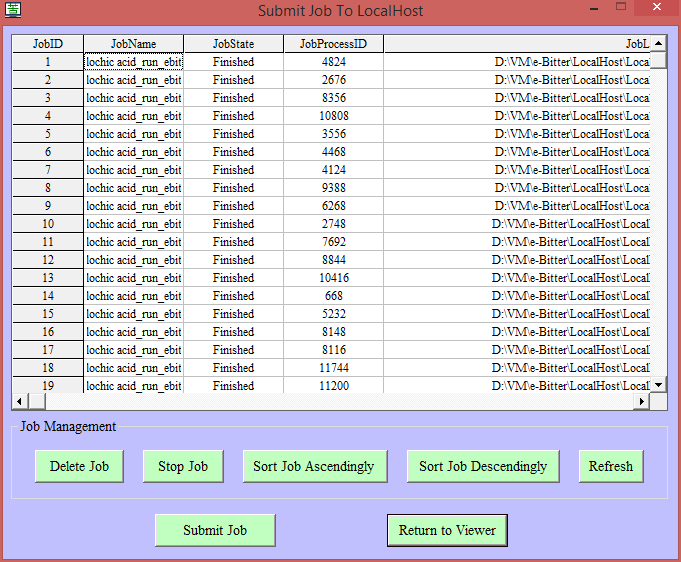 |
| --- |
| **Figure S15.** Job management system implemented in our e-Bitter program. |

**Table S1.** All the individual models for the bitterant/non-bitterant classification in this work.

| **Model** | **FS** | **EXP** | **FP** | **Method** | **Accuracy(test)** | **Precision (test)** | **Specificity(test)** | **Sensitivity(test)** | **F1-score**  **(test)** | **MCC**  **(test)** | **F1-score**  **(CV)** | **Parameters from CV*** |
| --- | --- | --- | --- | --- | --- | --- | --- | --- | --- | --- | --- | --- |
| M0001 | full | exp01 | 1024bit-ECFP4 | KNN | 0.853 | 0.871 | 0.847 | 0.858 | 0.864 | 0.705 | 0.895 | 9 distance |
| M0002 | full | exp02 | 1024bit-ECFP4 | KNN | 0.884 | 0.917 | 0.907 | 0.865 | 0.891 | 0.769 | 0.897 | 7 distance |
| M0003 | full | exp03 | 1024bit-ECFP4 | KNN | 0.873 | 0.886 | 0.864 | 0.879 | 0.883 | 0.743 | 0.891 | 7 distance |
| M0004 | full | exp04 | 1024bit-ECFP4 | KNN | 0.869 | 0.891 | 0.873 | 0.865 | 0.878 | 0.736 | 0.890 | 7 distance |
| M0005 | full | exp05 | 1024bit-ECFP4 | KNN | 0.931 | 0.924 | 0.907 | 0.950 | 0.937 | 0.860 | 0.891 | 7 distance |
| M0006 | full | exp06 | 1024bit-ECFP4 | KNN | 0.884 | 0.917 | 0.907 | 0.865 | 0.891 | 0.769 | 0.886 | 7 distance |
| M0007 | full | exp07 | 1024bit-ECFP4 | KNN | 0.911 | 0.928 | 0.915 | 0.908 | 0.918 | 0.822 | 0.892 | 7 distance |
| M0008 | full | exp08 | 1024bit-ECFP4 | KNN | 0.911 | 0.934 | 0.924 | 0.901 | 0.917 | 0.822 | 0.878 | 3 distance |
| M0009 | full | exp09 | 1024bit-ECFP4 | KNN | 0.915 | 0.934 | 0.924 | 0.908 | 0.921 | 0.830 | 0.888 | 5 uniform |
| M0010 | full | exp10 | 1024bit-ECFP4 | KNN | 0.876 | 0.876 | 0.847 | 0.901 | 0.888 | 0.751 | 0.884 | 9 distance |
| M0011 | full | exp11 | 1024bit-ECFP4 | KNN | 0.900 | 0.908 | 0.890 | 0.908 | 0.908 | 0.798 | 0.900 | 5 distance |
| M0012 | full | exp12 | 1024bit-ECFP4 | KNN | 0.873 | 0.909 | 0.898 | 0.851 | 0.879 | 0.747 | 0.904 | 5 distance |
| M0013 | full | exp13 | 1024bit-ECFP4 | KNN | 0.865 | 0.879 | 0.856 | 0.872 | 0.875 | 0.728 | 0.903 | 3 distance |
| M0014 | full | exp14 | 1024bit-ECFP4 | KNN | 0.884 | 0.894 | 0.873 | 0.894 | 0.894 | 0.766 | 0.885 | 7 distance |
| M0015 | full | exp15 | 1024bit-ECFP4 | KNN | 0.900 | 0.926 | 0.915 | 0.887 | 0.906 | 0.799 | 0.881 | 5 distance |
| M0016 | full | exp16 | 1024bit-ECFP4 | KNN | 0.900 | 0.908 | 0.890 | 0.908 | 0.908 | 0.798 | 0.886 | 5 distance |
| M0017 | full | exp17 | 1024bit-ECFP4 | KNN | 0.892 | 0.938 | 0.932 | 0.858 | 0.896 | 0.787 | 0.897 | 3 distance |
| M0018 | full | exp18 | 1024bit-ECFP4 | KNN | 0.896 | 0.919 | 0.907 | 0.887 | 0.903 | 0.791 | 0.888 | 5 distance |
| M0019 | full | exp19 | 1024bit-ECFP4 | KNN | 0.896 | 0.932 | 0.924 | 0.872 | 0.901 | 0.793 | 0.892 | 3 uniform |
| M0020 | full | exp01 | 1024bit-ECFP4 | SVM | 0.915 | 0.905 | 0.881 | 0.943 | 0.924 | 0.829 | 0.915 | 5 0.1 |
| M0021 | full | exp02 | 1024bit-ECFP4 | SVM | 0.896 | 0.880 | 0.847 | 0.936 | 0.907 | 0.790 | 0.916 | 50 0.05 |
| M0022 | full | exp03 | 1024bit-ECFP4 | SVM | 0.876 | 0.861 | 0.822 | 0.922 | 0.890 | 0.752 | 0.921 | 10 0.1 |
| M0023 | full | exp04 | 1024bit-ECFP4 | SVM | 0.915 | 0.889 | 0.856 | 0.965 | 0.925 | 0.831 | 0.914 | 100000.0 0.05 |
| M0024 | full | exp05 | 1024bit-ECFP4 | SVM | 0.915 | 0.884 | 0.847 | 0.972 | 0.926 | 0.832 | 0.920 | 5 0.05 |
| M0025 | full | exp06 | 1024bit-ECFP4 | SVM | 0.915 | 0.934 | 0.924 | 0.908 | 0.921 | 0.830 | 0.907 | 5000.0 0.01 |
| M0026 | full | exp07 | 1024bit-ECFP4 | SVM | 0.888 | 0.889 | 0.864 | 0.908 | 0.898 | 0.774 | 0.915 | 10 0.01 |
| M0027 | full | exp08 | 1024bit-ECFP4 | SVM | 0.927 | 0.936 | 0.924 | 0.929 | 0.932 | 0.852 | 0.910 | 5 0.05 |
| M0028 | full | exp09 | 1024bit-ECFP4 | SVM | 0.950 | 0.944 | 0.932 | 0.965 | 0.954 | 0.899 | 0.912 | 100 0.05 |
| M0029 | full | exp10 | 1024bit-ECFP4 | SVM | 0.911 | 0.893 | 0.864 | 0.950 | 0.921 | 0.822 | 0.908 | 100000.0 0.05 |
| M0030 | full | exp11 | 1024bit-ECFP4 | SVM | 0.911 | 0.910 | 0.890 | 0.929 | 0.919 | 0.821 | 0.914 | 10 0.05 |
| M0031 | full | exp12 | 1024bit-ECFP4 | SVM | 0.892 | 0.884 | 0.856 | 0.922 | 0.903 | 0.782 | 0.918 | 1 0.05 |
| M0032 | full | exp13 | 1024bit-ECFP4 | SVM | 0.888 | 0.878 | 0.847 | 0.922 | 0.900 | 0.774 | 0.927 | 10 0.05 |
| M0033 | full | exp14 | 1024bit-ECFP4 | SVM | 0.892 | 0.874 | 0.839 | 0.936 | 0.904 | 0.783 | 0.921 | 10 0.05 |
| M0034 | full | exp15 | 1024bit-ECFP4 | SVM | 0.888 | 0.864 | 0.822 | 0.943 | 0.902 | 0.776 | 0.905 | 100000.0 0.05 |
| M0035 | full | exp16 | 1024bit-ECFP4 | SVM | 0.923 | 0.923 | 0.907 | 0.936 | 0.930 | 0.844 | 0.920 | 5 0.05 |
| M0036 | full | exp17 | 1024bit-ECFP4 | SVM | 0.919 | 0.923 | 0.907 | 0.929 | 0.926 | 0.836 | 0.918 | 10 0.05 |
| M0037 | full | exp18 | 1024bit-ECFP4 | SVM | 0.903 | 0.920 | 0.907 | 0.901 | 0.910 | 0.806 | 0.911 | 10 0.05 |
| M0038 | full | exp19 | 1024bit-ECFP4 | SVM | 0.923 | 0.912 | 0.890 | 0.950 | 0.931 | 0.845 | 0.918 | 10000.0 0.05 |
| M0039 | full | exp01 | 1024bit-ECFP4 | GBM | 0.865 | 0.873 | 0.847 | 0.879 | 0.876 | 0.727 | 0.897 | 600 0.4 |
| M0040 | full | exp02 | 1024bit-ECFP4 | GBM | 0.876 | 0.876 | 0.847 | 0.901 | 0.888 | 0.751 | 0.900 | 600 0.1 |
| M0041 | full | exp03 | 1024bit-ECFP4 | GBM | 0.880 | 0.893 | 0.873 | 0.887 | 0.890 | 0.759 | 0.913 | 1000 0.1 |
| M0042 | full | exp04 | 1024bit-ECFP4 | GBM | 0.873 | 0.880 | 0.856 | 0.887 | 0.883 | 0.743 | 0.899 | 800 0.2 |
| M0043 | full | exp05 | 1024bit-ECFP4 | GBM | 0.903 | 0.892 | 0.864 | 0.936 | 0.913 | 0.806 | 0.910 | 700 0.2 |
| M0044 | full | exp06 | 1024bit-ECFP4 | GBM | 0.907 | 0.915 | 0.898 | 0.915 | 0.915 | 0.813 | 0.896 | 400 0.7 |
| M0045 | full | exp07 | 1024bit-ECFP4 | GBM | 0.892 | 0.895 | 0.873 | 0.908 | 0.901 | 0.782 | 0.902 | 300 0.3 |
| M0046 | full | exp08 | 1024bit-ECFP4 | GBM | 0.907 | 0.887 | 0.856 | 0.950 | 0.918 | 0.814 | 0.903 | 200 0.2 |
| M0047 | full | exp09 | 1024bit-ECFP4 | GBM | 0.927 | 0.936 | 0.924 | 0.929 | 0.932 | 0.852 | 0.884 | 50 0.5 |
| M0048 | full | exp10 | 1024bit-ECFP4 | GBM | 0.880 | 0.872 | 0.839 | 0.915 | 0.893 | 0.759 | 0.897 | 400 0.2 |
| M0049 | full | exp11 | 1024bit-ECFP4 | GBM | 0.900 | 0.914 | 0.898 | 0.901 | 0.907 | 0.798 | 0.899 | 300 0.1 |
| M0050 | full | exp12 | 1024bit-ECFP4 | GBM | 0.876 | 0.887 | 0.864 | 0.887 | 0.887 | 0.751 | 0.900 | 400 0.1 |
| M0051 | full | exp13 | 1024bit-ECFP4 | GBM | 0.876 | 0.871 | 0.839 | 0.908 | 0.889 | 0.751 | 0.901 | 500 0.4 |
| M0052 | full | exp14 | 1024bit-ECFP4 | GBM | 0.888 | 0.884 | 0.856 | 0.915 | 0.899 | 0.774 | 0.908 | 200 0.7 |
| M0053 | full | exp15 | 1024bit-ECFP4 | GBM | 0.873 | 0.870 | 0.839 | 0.901 | 0.885 | 0.743 | 0.897 | 500 0.4 |
| M0054 | full | exp16 | 1024bit-ECFP4 | GBM | 0.907 | 0.909 | 0.890 | 0.922 | 0.915 | 0.813 | 0.895 | 400 0.3 |
| M0055 | full | exp17 | 1024bit-ECFP4 | GBM | 0.900 | 0.908 | 0.890 | 0.908 | 0.908 | 0.798 | 0.899 | 1000 0.2 |
| M0056 | full | exp18 | 1024bit-ECFP4 | GBM | 0.911 | 0.940 | 0.932 | 0.894 | 0.916 | 0.823 | 0.896 | 400 0.1 |
| M0057 | full | exp19 | 1024bit-ECFP4 | GBM | 0.923 | 0.942 | 0.932 | 0.915 | 0.928 | 0.845 | 0.898 | 200 0.3 |
| M0058 | full | exp01 | 1024bit-ECFP4 | RF | 0.880 | 0.877 | 0.847 | 0.908 | 0.892 | 0.758 | 0.917 | 600 |
| M0059 | full | exp02 | 1024bit-ECFP4 | RF | 0.888 | 0.873 | 0.839 | 0.929 | 0.900 | 0.775 | 0.918 | 800 |
| M0060 | full | exp03 | 1024bit-ECFP4 | RF | 0.900 | 0.881 | 0.847 | 0.943 | 0.911 | 0.799 | 0.914 | 700 |
| M0061 | full | exp04 | 1024bit-ECFP4 | RF | 0.900 | 0.891 | 0.864 | 0.929 | 0.910 | 0.798 | 0.915 | 900 |
| M0062 | full | exp05 | 1024bit-ECFP4 | RF | 0.915 | 0.889 | 0.856 | 0.965 | 0.925 | 0.831 | 0.915 | 900 |
| M0063 | full | exp06 | 1024bit-ECFP4 | RF | 0.927 | 0.924 | 0.907 | 0.943 | 0.933 | 0.852 | 0.920 | 200 |
| M0064 | full | exp07 | 1024bit-ECFP4 | RF | 0.915 | 0.899 | 0.873 | 0.950 | 0.924 | 0.829 | 0.904 | 900 |
| M0065 | full | exp08 | 1024bit-ECFP4 | RF | 0.919 | 0.911 | 0.890 | 0.943 | 0.927 | 0.837 | 0.911 | 700 |
| M0066 | full | exp09 | 1024bit-ECFP4 | RF | 0.946 | 0.938 | 0.924 | 0.965 | 0.951 | 0.891 | 0.904 | 700 |
| M0067 | full | exp10 | 1024bit-ECFP4 | RF | 0.900 | 0.862 | 0.814 | 0.972 | 0.913 | 0.803 | 0.916 | 900 |
| M0068 | full | exp11 | 1024bit-ECFP4 | RF | 0.923 | 0.917 | 0.898 | 0.943 | 0.930 | 0.844 | 0.911 | 300 |
| M0069 | full | exp12 | 1024bit-ECFP4 | RF | 0.892 | 0.895 | 0.873 | 0.908 | 0.901 | 0.782 | 0.916 | 1000 |
| M0070 | full | exp13 | 1024bit-ECFP4 | RF | 0.903 | 0.897 | 0.873 | 0.929 | 0.913 | 0.805 | 0.919 | 300 |
| M0071 | full | exp14 | 1024bit-ECFP4 | RF | 0.900 | 0.871 | 0.831 | 0.957 | 0.912 | 0.801 | 0.917 | 1000 |
| M0072 | full | exp15 | 1024bit-ECFP4 | RF | 0.876 | 0.861 | 0.822 | 0.922 | 0.890 | 0.752 | 0.907 | 50 |
| M0073 | full | exp16 | 1024bit-ECFP4 | RF | 0.927 | 0.918 | 0.898 | 0.950 | 0.934 | 0.852 | 0.912 | 1000 |
| M0074 | full | exp17 | 1024bit-ECFP4 | RF | 0.907 | 0.915 | 0.898 | 0.915 | 0.915 | 0.813 | 0.918 | 800 |
| M0075 | full | exp18 | 1024bit-ECFP4 | RF | 0.911 | 0.928 | 0.915 | 0.908 | 0.918 | 0.822 | 0.904 | 600 |
| M0076 | full | exp19 | 1024bit-ECFP4 | RF | 0.934 | 0.925 | 0.907 | 0.957 | 0.941 | 0.868 | 0.920 | 200 |
| M0077 | full | exp01 | 1024bit-ECFP4 | DNN2 | 0.903 | 0.914 | 0.898 | 0.908 | 0.911 | 0.806 | 0.911 | 600 140 0.4 |
| M0078 | full | exp02 | 1024bit-ECFP4 | DNN2 | 0.888 | 0.894 | 0.873 | 0.901 | 0.898 | 0.774 | 0.921 | 100 80 0.1 |
| M0079 | full | exp03 | 1024bit-ECFP4 | DNN2 | 0.896 | 0.901 | 0.881 | 0.908 | 0.905 | 0.790 | 0.922 | 600 140 0.3 |
| M0080 | full | exp01 | 1024bit-ECFP4 | DNN3 | 0.907 | 0.903 | 0.881 | 0.929 | 0.916 | 0.813 | 0.916 | 400 60 0.2 |
| M0081 | full | exp02 | 1024bit-ECFP4 | DNN3 | 0.911 | 0.921 | 0.907 | 0.915 | 0.918 | 0.821 | 0.919 | 400 60 0.1 |
| M0082 | full | exp03 | 1024bit-ECFP4 | DNN3 | 0.903 | 0.903 | 0.881 | 0.922 | 0.912 | 0.805 | 0.916 | 300 160 0.4 |
| M0083 | full | exp01 | 2048bit-ECFP4 | KNN | 0.876 | 0.892 | 0.873 | 0.879 | 0.886 | 0.751 | 0.904 | 5 distance |
| M0084 | full | exp02 | 2048bit-ECFP4 | KNN | 0.884 | 0.924 | 0.915 | 0.858 | 0.890 | 0.770 | 0.903 | 7 distance |
| M0085 | full | exp03 | 2048bit-ECFP4 | KNN | 0.892 | 0.906 | 0.890 | 0.894 | 0.900 | 0.782 | 0.898 | 3 distance |
| M0086 | full | exp04 | 2048bit-ECFP4 | KNN | 0.884 | 0.894 | 0.873 | 0.894 | 0.894 | 0.766 | 0.894 | 5 distance |
| M0087 | full | exp05 | 2048bit-ECFP4 | KNN | 0.934 | 0.931 | 0.915 | 0.950 | 0.940 | 0.868 | 0.897 | 5 distance |
| M0088 | full | exp06 | 2048bit-ECFP4 | KNN | 0.888 | 0.931 | 0.924 | 0.858 | 0.893 | 0.779 | 0.892 | 5 distance |
| M0089 | full | exp07 | 2048bit-ECFP4 | KNN | 0.911 | 0.928 | 0.915 | 0.908 | 0.918 | 0.822 | 0.895 | 5 distance |
| M0090 | full | exp08 | 2048bit-ECFP4 | KNN | 0.884 | 0.917 | 0.907 | 0.865 | 0.891 | 0.769 | 0.884 | 7 distance |
| M0091 | full | exp09 | 2048bit-ECFP4 | KNN | 0.919 | 0.929 | 0.915 | 0.922 | 0.925 | 0.837 | 0.888 | 7 distance |
| M0092 | full | exp10 | 2048bit-ECFP4 | KNN | 0.876 | 0.892 | 0.873 | 0.879 | 0.886 | 0.751 | 0.894 | 3 distance |
| M0093 | full | exp11 | 2048bit-ECFP4 | KNN | 0.907 | 0.921 | 0.907 | 0.908 | 0.914 | 0.814 | 0.897 | 5 distance |
| M0094 | full | exp12 | 2048bit-ECFP4 | KNN | 0.884 | 0.905 | 0.890 | 0.879 | 0.892 | 0.768 | 0.902 | 5 distance |
| M0095 | full | exp13 | 2048bit-ECFP4 | KNN | 0.876 | 0.887 | 0.864 | 0.887 | 0.887 | 0.751 | 0.906 | 3 distance |
| M0096 | full | exp14 | 2048bit-ECFP4 | KNN | 0.880 | 0.899 | 0.881 | 0.879 | 0.889 | 0.759 | 0.889 | 5 distance |
| M0097 | full | exp15 | 2048bit-ECFP4 | KNN | 0.903 | 0.926 | 0.915 | 0.894 | 0.910 | 0.807 | 0.883 | 5 distance |
| M0098 | full | exp16 | 2048bit-ECFP4 | KNN | 0.888 | 0.906 | 0.890 | 0.887 | 0.896 | 0.775 | 0.891 | 9 distance |
| M0099 | full | exp17 | 2048bit-ECFP4 | KNN | 0.900 | 0.939 | 0.932 | 0.872 | 0.904 | 0.801 | 0.899 | 3 uniform |
| M0100 | full | exp18 | 2048bit-ECFP4 | KNN | 0.888 | 0.918 | 0.907 | 0.872 | 0.895 | 0.777 | 0.889 | 5 distance |
| M0101 | full | exp19 | 2048bit-ECFP4 | KNN | 0.892 | 0.931 | 0.924 | 0.865 | 0.897 | 0.786 | 0.889 | 3 uniform |
| M0102 | full | exp01 | 2048bit-ECFP4 | SVM | 0.892 | 0.879 | 0.847 | 0.929 | 0.903 | 0.782 | 0.921 | 1 0.05 |
| M0103 | full | exp02 | 2048bit-ECFP4 | SVM | 0.884 | 0.872 | 0.839 | 0.922 | 0.897 | 0.767 | 0.920 | 1000.0 0.05 |
| M0104 | full | exp03 | 2048bit-ECFP4 | SVM | 0.892 | 0.901 | 0.881 | 0.901 | 0.901 | 0.782 | 0.928 | 5 0.01 |
| M0105 | full | exp04 | 2048bit-ECFP4 | SVM | 0.915 | 0.894 | 0.864 | 0.957 | 0.925 | 0.830 | 0.919 | 5 0.05 |
| M0106 | full | exp05 | 2048bit-ECFP4 | SVM | 0.911 | 0.873 | 0.831 | 0.979 | 0.923 | 0.826 | 0.925 | 100000.0 0.05 |
| M0107 | full | exp06 | 2048bit-ECFP4 | SVM | 0.931 | 0.942 | 0.932 | 0.929 | 0.936 | 0.860 | 0.914 | 5 0.01 |
| M0108 | full | exp07 | 2048bit-ECFP4 | SVM | 0.927 | 0.924 | 0.907 | 0.943 | 0.933 | 0.852 | 0.914 | 5 0.01 |
| M0109 | full | exp08 | 2048bit-ECFP4 | SVM | 0.927 | 0.936 | 0.924 | 0.929 | 0.932 | 0.852 | 0.912 | 100 0.05 |
| M0110 | full | exp09 | 2048bit-ECFP4 | SVM | 0.946 | 0.944 | 0.932 | 0.957 | 0.951 | 0.891 | 0.914 | 5000.0 0.05 |
| M0111 | full | exp10 | 2048bit-ECFP4 | SVM | 0.915 | 0.889 | 0.856 | 0.965 | 0.925 | 0.831 | 0.913 | 500 0.05 |
| M0112 | full | exp11 | 2048bit-ECFP4 | SVM | 0.919 | 0.929 | 0.915 | 0.922 | 0.925 | 0.837 | 0.923 | 10 0.01 |
| M0113 | full | exp12 | 2048bit-ECFP4 | SVM | 0.884 | 0.883 | 0.856 | 0.908 | 0.895 | 0.766 | 0.924 | 500 0.05 |
| M0114 | full | exp13 | 2048bit-ECFP4 | SVM | 0.900 | 0.891 | 0.864 | 0.929 | 0.910 | 0.798 | 0.926 | 10 0.05 |
| M0115 | full | exp14 | 2048bit-ECFP4 | SVM | 0.903 | 0.882 | 0.847 | 0.950 | 0.915 | 0.807 | 0.920 | 5000.0 0.05 |
| M0116 | full | exp15 | 2048bit-ECFP4 | SVM | 0.888 | 0.864 | 0.822 | 0.943 | 0.902 | 0.776 | 0.910 | 10 0.05 |
| M0117 | full | exp16 | 2048bit-ECFP4 | SVM | 0.931 | 0.918 | 0.898 | 0.957 | 0.938 | 0.860 | 0.921 | 500 0.05 |
| M0118 | full | exp17 | 2048bit-ECFP4 | SVM | 0.911 | 0.910 | 0.890 | 0.929 | 0.919 | 0.821 | 0.922 | 100 0.05 |
| M0119 | full | exp18 | 2048bit-ECFP4 | SVM | 0.900 | 0.914 | 0.898 | 0.901 | 0.907 | 0.798 | 0.919 | 100 0.01 |
| M0120 | full | exp19 | 2048bit-ECFP4 | SVM | 0.934 | 0.919 | 0.898 | 0.965 | 0.941 | 0.868 | 0.918 | 10 0.05 |
| M0121 | full | exp01 | 2048bit-ECFP4 | GBM | 0.903 | 0.903 | 0.881 | 0.922 | 0.912 | 0.805 | 0.892 | 200 0.3 |
| M0122 | full | exp02 | 2048bit-ECFP4 | GBM | 0.876 | 0.871 | 0.839 | 0.908 | 0.889 | 0.751 | 0.898 | 400 0.3 |
| M0123 | full | exp03 | 2048bit-ECFP4 | GBM | 0.876 | 0.881 | 0.856 | 0.894 | 0.887 | 0.751 | 0.907 | 100 0.4 |
| M0124 | full | exp04 | 2048bit-ECFP4 | GBM | 0.907 | 0.893 | 0.864 | 0.943 | 0.917 | 0.814 | 0.890 | 500 0.1 |
| M0125 | full | exp05 | 2048bit-ECFP4 | GBM | 0.903 | 0.887 | 0.856 | 0.943 | 0.914 | 0.806 | 0.898 | 200 0.8 |
| M0126 | full | exp06 | 2048bit-ECFP4 | GBM | 0.907 | 0.933 | 0.924 | 0.894 | 0.913 | 0.815 | 0.890 | 700 0.1 |
| M0127 | full | exp07 | 2048bit-ECFP4 | GBM | 0.900 | 0.886 | 0.856 | 0.936 | 0.910 | 0.798 | 0.894 | 200 0.3 |
| M0128 | full | exp08 | 2048bit-ECFP4 | GBM | 0.900 | 0.891 | 0.864 | 0.929 | 0.910 | 0.798 | 0.890 | 500 0.1 |
| M0129 | full | exp09 | 2048bit-ECFP4 | GBM | 0.946 | 0.950 | 0.941 | 0.950 | 0.950 | 0.891 | 0.885 | 200 0.4 |
| M0130 | full | exp10 | 2048bit-ECFP4 | GBM | 0.876 | 0.866 | 0.831 | 0.915 | 0.890 | 0.751 | 0.897 | 600 0.1 |
| M0131 | full | exp11 | 2048bit-ECFP4 | GBM | 0.896 | 0.913 | 0.898 | 0.894 | 0.903 | 0.790 | 0.905 | 800 0.1 |
| M0132 | full | exp12 | 2048bit-ECFP4 | GBM | 0.884 | 0.894 | 0.873 | 0.894 | 0.894 | 0.766 | 0.899 | 600 0.1 |
| M0133 | full | exp13 | 2048bit-ECFP4 | GBM | 0.880 | 0.872 | 0.839 | 0.915 | 0.893 | 0.759 | 0.908 | 900 0.2 |
| M0134 | full | exp14 | 2048bit-ECFP4 | GBM | 0.876 | 0.881 | 0.856 | 0.894 | 0.887 | 0.751 | 0.903 | 600 0.2 |
| M0135 | full | exp15 | 2048bit-ECFP4 | GBM | 0.876 | 0.861 | 0.822 | 0.922 | 0.890 | 0.752 | 0.888 | 200 0.2 |
| M0136 | full | exp16 | 2048bit-ECFP4 | GBM | 0.900 | 0.897 | 0.873 | 0.922 | 0.909 | 0.797 | 0.901 | 500 0.1 |
| M0137 | full | exp17 | 2048bit-ECFP4 | GBM | 0.880 | 0.893 | 0.873 | 0.887 | 0.890 | 0.759 | 0.903 | 900 0.1 |
| M0138 | full | exp18 | 2048bit-ECFP4 | GBM | 0.888 | 0.900 | 0.881 | 0.894 | 0.897 | 0.774 | 0.891 | 200 0.5 |
| M0139 | full | exp19 | 2048bit-ECFP4 | GBM | 0.915 | 0.905 | 0.881 | 0.943 | 0.924 | 0.829 | 0.891 | 100 0.2 |
| M0140 | full | exp01 | 2048bit-ECFP4 | RF | 0.892 | 0.890 | 0.864 | 0.915 | 0.902 | 0.782 | 0.921 | 900 |
| M0141 | full | exp02 | 2048bit-ECFP4 | RF | 0.888 | 0.878 | 0.847 | 0.922 | 0.900 | 0.774 | 0.921 | 400 |
| M0142 | full | exp03 | 2048bit-ECFP4 | RF | 0.896 | 0.875 | 0.839 | 0.943 | 0.908 | 0.791 | 0.920 | 100 |
| M0143 | full | exp04 | 2048bit-ECFP4 | RF | 0.896 | 0.885 | 0.856 | 0.929 | 0.907 | 0.790 | 0.911 | 400 |
| M0144 | full | exp05 | 2048bit-ECFP4 | RF | 0.927 | 0.901 | 0.873 | 0.972 | 0.935 | 0.854 | 0.916 | 50 |
| M0145 | full | exp06 | 2048bit-ECFP4 | RF | 0.919 | 0.929 | 0.915 | 0.922 | 0.925 | 0.837 | 0.915 | 100 |
| M0146 | full | exp07 | 2048bit-ECFP4 | RF | 0.919 | 0.900 | 0.873 | 0.957 | 0.928 | 0.838 | 0.906 | 1000 |
| M0147 | full | exp08 | 2048bit-ECFP4 | RF | 0.919 | 0.917 | 0.898 | 0.936 | 0.926 | 0.836 | 0.912 | 900 |
| M0148 | full | exp09 | 2048bit-ECFP4 | RF | 0.946 | 0.938 | 0.924 | 0.965 | 0.951 | 0.891 | 0.903 | 700 |
| M0149 | full | exp10 | 2048bit-ECFP4 | RF | 0.900 | 0.862 | 0.814 | 0.972 | 0.913 | 0.803 | 0.918 | 100 |
| M0150 | full | exp11 | 2048bit-ECFP4 | RF | 0.927 | 0.918 | 0.898 | 0.950 | 0.934 | 0.852 | 0.915 | 200 |
| M0151 | full | exp12 | 2048bit-ECFP4 | RF | 0.900 | 0.908 | 0.890 | 0.908 | 0.908 | 0.798 | 0.916 | 800 |
| M0152 | full | exp13 | 2048bit-ECFP4 | RF | 0.896 | 0.885 | 0.856 | 0.929 | 0.907 | 0.790 | 0.922 | 600 |
| M0153 | full | exp14 | 2048bit-ECFP4 | RF | 0.903 | 0.882 | 0.847 | 0.950 | 0.915 | 0.807 | 0.921 | 1000 |
| M0154 | full | exp15 | 2048bit-ECFP4 | RF | 0.903 | 0.897 | 0.873 | 0.929 | 0.913 | 0.805 | 0.911 | 50 |
| M0155 | full | exp16 | 2048bit-ECFP4 | RF | 0.923 | 0.912 | 0.890 | 0.950 | 0.931 | 0.845 | 0.913 | 50 |
| M0156 | full | exp17 | 2048bit-ECFP4 | RF | 0.907 | 0.915 | 0.898 | 0.915 | 0.915 | 0.813 | 0.923 | 700 |
| M0157 | full | exp18 | 2048bit-ECFP4 | RF | 0.915 | 0.934 | 0.924 | 0.908 | 0.921 | 0.830 | 0.909 | 600 |
| M0158 | full | exp19 | 2048bit-ECFP4 | RF | 0.934 | 0.919 | 0.898 | 0.965 | 0.941 | 0.868 | 0.920 | 400 |
| M0159 | full | exp01 | 2048bit-ECFP4 | DNN2 | 0.919 | 0.929 | 0.915 | 0.922 | 0.925 | 0.837 | 0.919 | 200 120 0.5 |
| M0160 | full | exp02 | 2048bit-ECFP4 | DNN2 | 0.900 | 0.897 | 0.873 | 0.922 | 0.909 | 0.797 | 0.919 | 100 120 0.4 |
| M0161 | full | exp03 | 2048bit-ECFP4 | DNN2 | 0.907 | 0.903 | 0.881 | 0.929 | 0.916 | 0.813 | 0.924 | 300 80 0.1 |
| M0162 | full | exp01 | 2048bit-ECFP4 | DNN3 | 0.888 | 0.864 | 0.822 | 0.943 | 0.902 | 0.776 | 0.895 | 200 60 0.5 |
| M0163 | full | exp02 | 2048bit-ECFP4 | DNN3 | 0.903 | 0.887 | 0.856 | 0.943 | 0.914 | 0.806 | 0.888 | 200 60 0.5 |
| M0164 | full | exp03 | 2048bit-ECFP4 | DNN3 | 0.888 | 0.894 | 0.873 | 0.901 | 0.898 | 0.774 | 0.896 | 100 60 0.5 |
| M0165 | full | exp01 | 1024bit-ECFP6 | KNN | 0.873 | 0.860 | 0.822 | 0.915 | 0.887 | 0.743 | 0.900 | 5 distance |
| M0166 | full | exp02 | 1024bit-ECFP6 | KNN | 0.884 | 0.894 | 0.873 | 0.894 | 0.894 | 0.766 | 0.892 | 7 distance |
| M0167 | full | exp03 | 1024bit-ECFP6 | KNN | 0.869 | 0.864 | 0.831 | 0.901 | 0.882 | 0.735 | 0.883 | 5 distance |
| M0168 | full | exp04 | 1024bit-ECFP6 | KNN | 0.888 | 0.868 | 0.831 | 0.936 | 0.901 | 0.775 | 0.887 | 5 distance |
| M0169 | full | exp05 | 1024bit-ECFP6 | KNN | 0.923 | 0.906 | 0.881 | 0.957 | 0.931 | 0.845 | 0.890 | 5 distance |
| M0170 | full | exp06 | 1024bit-ECFP6 | KNN | 0.873 | 0.897 | 0.881 | 0.865 | 0.881 | 0.745 | 0.891 | 3 distance |
| M0171 | full | exp07 | 1024bit-ECFP6 | KNN | 0.911 | 0.910 | 0.890 | 0.929 | 0.919 | 0.821 | 0.895 | 3 uniform |
| M0172 | full | exp08 | 1024bit-ECFP6 | KNN | 0.884 | 0.894 | 0.873 | 0.894 | 0.894 | 0.766 | 0.880 | 9 distance |
| M0173 | full | exp09 | 1024bit-ECFP6 | KNN | 0.915 | 0.899 | 0.873 | 0.950 | 0.924 | 0.829 | 0.887 | 7 distance |
| M0174 | full | exp10 | 1024bit-ECFP6 | KNN | 0.880 | 0.872 | 0.839 | 0.915 | 0.893 | 0.759 | 0.888 | 3 uniform |
| M0175 | full | exp11 | 1024bit-ECFP6 | KNN | 0.907 | 0.893 | 0.864 | 0.943 | 0.917 | 0.814 | 0.894 | 5 distance |
| M0176 | full | exp12 | 1024bit-ECFP6 | KNN | 0.869 | 0.891 | 0.873 | 0.865 | 0.878 | 0.736 | 0.892 | 3 distance |
| M0177 | full | exp13 | 1024bit-ECFP6 | KNN | 0.865 | 0.863 | 0.831 | 0.894 | 0.878 | 0.727 | 0.907 | 3 distance |
| M0178 | full | exp14 | 1024bit-ECFP6 | KNN | 0.876 | 0.856 | 0.814 | 0.929 | 0.891 | 0.752 | 0.887 | 11 distance |
| M0179 | full | exp15 | 1024bit-ECFP6 | KNN | 0.900 | 0.908 | 0.890 | 0.908 | 0.908 | 0.798 | 0.881 | 5 distance |
| M0180 | full | exp16 | 1024bit-ECFP6 | KNN | 0.896 | 0.901 | 0.881 | 0.908 | 0.905 | 0.790 | 0.888 | 5 uniform |
| M0181 | full | exp17 | 1024bit-ECFP6 | KNN | 0.900 | 0.920 | 0.907 | 0.894 | 0.906 | 0.799 | 0.895 | 3 distance |
| M0182 | full | exp18 | 1024bit-ECFP6 | KNN | 0.861 | 0.883 | 0.864 | 0.858 | 0.871 | 0.721 | 0.889 | 9 distance |
| M0183 | full | exp19 | 1024bit-ECFP6 | KNN | 0.888 | 0.906 | 0.890 | 0.887 | 0.896 | 0.775 | 0.886 | 3 uniform |
| M0184 | full | exp01 | 1024bit-ECFP6 | SVM | 0.903 | 0.892 | 0.864 | 0.936 | 0.913 | 0.806 | 0.905 | 50 0.05 |
| M0185 | full | exp02 | 1024bit-ECFP6 | SVM | 0.884 | 0.899 | 0.881 | 0.887 | 0.893 | 0.767 | 0.908 | 5 0.01 |
| M0186 | full | exp03 | 1024bit-ECFP6 | SVM | 0.865 | 0.863 | 0.831 | 0.894 | 0.878 | 0.727 | 0.916 | 100 0.01 |
| M0187 | full | exp04 | 1024bit-ECFP6 | SVM | 0.892 | 0.865 | 0.822 | 0.950 | 0.905 | 0.785 | 0.902 | 5000.0 0.05 |
| M0188 | full | exp05 | 1024bit-ECFP6 | SVM | 0.915 | 0.874 | 0.831 | 0.986 | 0.927 | 0.835 | 0.908 | 50 0.05 |
| M0189 | full | exp06 | 1024bit-ECFP6 | SVM | 0.903 | 0.920 | 0.907 | 0.901 | 0.910 | 0.806 | 0.914 | 5 0.01 |
| M0190 | full | exp07 | 1024bit-ECFP6 | SVM | 0.900 | 0.886 | 0.856 | 0.936 | 0.910 | 0.798 | 0.908 | 5 0.01 |
| M0191 | full | exp08 | 1024bit-ECFP6 | SVM | 0.907 | 0.903 | 0.881 | 0.929 | 0.916 | 0.813 | 0.899 | 1 0.05 |
| M0192 | full | exp09 | 1024bit-ECFP6 | SVM | 0.942 | 0.932 | 0.915 | 0.965 | 0.948 | 0.884 | 0.893 | 10 0.01 |
| M0193 | full | exp10 | 1024bit-ECFP6 | SVM | 0.915 | 0.884 | 0.847 | 0.972 | 0.926 | 0.832 | 0.901 | 100000.0 0.05 |
| M0194 | full | exp11 | 1024bit-ECFP6 | SVM | 0.915 | 0.910 | 0.890 | 0.936 | 0.923 | 0.829 | 0.905 | 5 0.01 |
| M0195 | full | exp12 | 1024bit-ECFP6 | SVM | 0.880 | 0.872 | 0.839 | 0.915 | 0.893 | 0.759 | 0.916 | 10000.0 0.05 |
| M0196 | full | exp13 | 1024bit-ECFP6 | SVM | 0.880 | 0.857 | 0.814 | 0.936 | 0.895 | 0.761 | 0.915 | 10000.0 0.05 |
| M0197 | full | exp14 | 1024bit-ECFP6 | SVM | 0.888 | 0.868 | 0.831 | 0.936 | 0.901 | 0.775 | 0.906 | 50 0.01 |
| M0198 | full | exp15 | 1024bit-ECFP6 | SVM | 0.884 | 0.854 | 0.805 | 0.950 | 0.899 | 0.770 | 0.902 | 5000.0 0.05 |
| M0199 | full | exp16 | 1024bit-ECFP6 | SVM | 0.903 | 0.887 | 0.856 | 0.943 | 0.914 | 0.806 | 0.909 | 10 0.01 |
| M0200 | full | exp17 | 1024bit-ECFP6 | SVM | 0.923 | 0.912 | 0.890 | 0.950 | 0.931 | 0.845 | 0.911 | 50000.0 0.05 |
| M0201 | full | exp18 | 1024bit-ECFP6 | SVM | 0.900 | 0.926 | 0.915 | 0.887 | 0.906 | 0.799 | 0.901 | 5 0.01 |
| M0202 | full | exp19 | 1024bit-ECFP6 | SVM | 0.923 | 0.906 | 0.881 | 0.957 | 0.931 | 0.845 | 0.907 | 50 0.05 |
| M0203 | full | exp01 | 1024bit-ECFP6 | GBM | 0.876 | 0.871 | 0.839 | 0.908 | 0.889 | 0.751 | 0.897 | 400 0.1 |
| M0204 | full | exp02 | 1024bit-ECFP6 | GBM | 0.884 | 0.894 | 0.873 | 0.894 | 0.894 | 0.766 | 0.895 | 300 0.4 |
| M0205 | full | exp03 | 1024bit-ECFP6 | GBM | 0.865 | 0.853 | 0.814 | 0.908 | 0.880 | 0.728 | 0.904 | 600 0.1 |
| M0206 | full | exp04 | 1024bit-ECFP6 | GBM | 0.896 | 0.875 | 0.839 | 0.943 | 0.908 | 0.791 | 0.901 | 400 0.1 |
| M0207 | full | exp05 | 1024bit-ECFP6 | GBM | 0.907 | 0.877 | 0.839 | 0.965 | 0.919 | 0.816 | 0.893 | 200 0.4 |
| M0208 | full | exp06 | 1024bit-ECFP6 | GBM | 0.911 | 0.921 | 0.907 | 0.915 | 0.918 | 0.821 | 0.909 | 500 0.3 |
| M0209 | full | exp07 | 1024bit-ECFP6 | GBM | 0.884 | 0.872 | 0.839 | 0.922 | 0.897 | 0.767 | 0.890 | 500 0.3 |
| M0210 | full | exp08 | 1024bit-ECFP6 | GBM | 0.903 | 0.892 | 0.864 | 0.936 | 0.913 | 0.806 | 0.898 | 500 0.1 |
| M0211 | full | exp09 | 1024bit-ECFP6 | GBM | 0.900 | 0.902 | 0.881 | 0.915 | 0.908 | 0.797 | 0.891 | 200 0.1 |
| M0212 | full | exp10 | 1024bit-ECFP6 | GBM | 0.892 | 0.865 | 0.822 | 0.950 | 0.905 | 0.785 | 0.899 | 100 0.7 |
| M0213 | full | exp11 | 1024bit-ECFP6 | GBM | 0.919 | 0.911 | 0.890 | 0.943 | 0.927 | 0.837 | 0.897 | 200 0.1 |
| M0214 | full | exp12 | 1024bit-ECFP6 | GBM | 0.884 | 0.905 | 0.890 | 0.879 | 0.892 | 0.768 | 0.902 | 300 0.4 |
| M0215 | full | exp13 | 1024bit-ECFP6 | GBM | 0.892 | 0.879 | 0.847 | 0.929 | 0.903 | 0.782 | 0.903 | 100 0.2 |
| M0216 | full | exp14 | 1024bit-ECFP6 | GBM | 0.884 | 0.872 | 0.839 | 0.922 | 0.897 | 0.767 | 0.899 | 400 0.4 |
| M0217 | full | exp15 | 1024bit-ECFP6 | GBM | 0.888 | 0.873 | 0.839 | 0.929 | 0.900 | 0.775 | 0.888 | 800 0.1 |
| M0218 | full | exp16 | 1024bit-ECFP6 | GBM | 0.900 | 0.897 | 0.873 | 0.922 | 0.909 | 0.797 | 0.901 | 200 0.7 |
| M0219 | full | exp17 | 1024bit-ECFP6 | GBM | 0.900 | 0.914 | 0.898 | 0.901 | 0.907 | 0.798 | 0.893 | 200 0.4 |
| M0220 | full | exp18 | 1024bit-ECFP6 | GBM | 0.896 | 0.919 | 0.907 | 0.887 | 0.903 | 0.791 | 0.890 | 100 0.3 |
| M0221 | full | exp19 | 1024bit-ECFP6 | GBM | 0.927 | 0.930 | 0.915 | 0.936 | 0.933 | 0.852 | 0.900 | 300 0.3 |
| M0222 | full | exp01 | 1024bit-ECFP6 | RF | 0.873 | 0.855 | 0.814 | 0.922 | 0.887 | 0.744 | 0.914 | 100 |
| M0223 | full | exp02 | 1024bit-ECFP6 | RF | 0.884 | 0.863 | 0.822 | 0.936 | 0.898 | 0.768 | 0.910 | 200 |
| M0224 | full | exp03 | 1024bit-ECFP6 | RF | 0.888 | 0.859 | 0.814 | 0.950 | 0.902 | 0.777 | 0.914 | 500 |
| M0225 | full | exp04 | 1024bit-ECFP6 | RF | 0.903 | 0.882 | 0.847 | 0.950 | 0.915 | 0.807 | 0.910 | 400 |
| M0226 | full | exp05 | 1024bit-ECFP6 | RF | 0.911 | 0.873 | 0.831 | 0.979 | 0.923 | 0.826 | 0.907 | 800 |
| M0227 | full | exp06 | 1024bit-ECFP6 | RF | 0.923 | 0.917 | 0.898 | 0.943 | 0.930 | 0.844 | 0.910 | 400 |
| M0228 | full | exp07 | 1024bit-ECFP6 | RF | 0.903 | 0.882 | 0.847 | 0.950 | 0.915 | 0.807 | 0.901 | 800 |
| M0229 | full | exp08 | 1024bit-ECFP6 | RF | 0.923 | 0.912 | 0.890 | 0.950 | 0.931 | 0.845 | 0.912 | 600 |
| M0230 | full | exp09 | 1024bit-ECFP6 | RF | 0.946 | 0.926 | 0.907 | 0.979 | 0.952 | 0.892 | 0.899 | 900 |
| M0231 | full | exp10 | 1024bit-ECFP6 | RF | 0.907 | 0.873 | 0.831 | 0.972 | 0.919 | 0.818 | 0.908 | 1000 |
| M0232 | full | exp11 | 1024bit-ECFP6 | RF | 0.911 | 0.883 | 0.847 | 0.965 | 0.922 | 0.824 | 0.914 | 50 |
| M0233 | full | exp12 | 1024bit-ECFP6 | RF | 0.900 | 0.897 | 0.873 | 0.922 | 0.909 | 0.797 | 0.912 | 800 |
| M0234 | full | exp13 | 1024bit-ECFP6 | RF | 0.888 | 0.878 | 0.847 | 0.922 | 0.900 | 0.774 | 0.915 | 700 |
| M0235 | full | exp14 | 1024bit-ECFP6 | RF | 0.900 | 0.866 | 0.822 | 0.965 | 0.913 | 0.802 | 0.918 | 200 |
| M0236 | full | exp15 | 1024bit-ECFP6 | RF | 0.892 | 0.879 | 0.847 | 0.929 | 0.903 | 0.782 | 0.905 | 1000 |
| M0237 | full | exp16 | 1024bit-ECFP6 | RF | 0.911 | 0.893 | 0.864 | 0.950 | 0.921 | 0.822 | 0.909 | 700 |
| M0238 | full | exp17 | 1024bit-ECFP6 | RF | 0.896 | 0.896 | 0.873 | 0.915 | 0.905 | 0.790 | 0.910 | 1000 |
| M0239 | full | exp18 | 1024bit-ECFP6 | RF | 0.907 | 0.915 | 0.898 | 0.915 | 0.915 | 0.813 | 0.902 | 600 |
| M0240 | full | exp19 | 1024bit-ECFP6 | RF | 0.931 | 0.918 | 0.898 | 0.957 | 0.938 | 0.860 | 0.914 | 400 |
| M0241 | full | exp01 | 1024bit-ECFP6 | DNN2 | 0.903 | 0.892 | 0.864 | 0.936 | 0.913 | 0.806 | 0.906 | 300 140 0.3 |
| M0242 | full | exp02 | 1024bit-ECFP6 | DNN2 | 0.903 | 0.908 | 0.890 | 0.915 | 0.912 | 0.805 | 0.905 | 400 100 0.5 |
| M0243 | full | exp03 | 1024bit-ECFP6 | DNN2 | 0.861 | 0.862 | 0.831 | 0.887 | 0.874 | 0.719 | 0.917 | 100 60 0.5 |
| M0244 | full | exp01 | 1024bit-ECFP6 | DNN3 | 0.903 | 0.903 | 0.881 | 0.922 | 0.912 | 0.805 | 0.902 | 100 160 0.3 |
| M0245 | full | exp02 | 1024bit-ECFP6 | DNN3 | 0.896 | 0.896 | 0.873 | 0.915 | 0.905 | 0.790 | 0.904 | 100 100 0.2 |
| M0246 | full | exp03 | 1024bit-ECFP6 | DNN3 | 0.873 | 0.870 | 0.839 | 0.901 | 0.885 | 0.743 | 0.914 | 500 80 0.5 |
| M0247 | full | exp01 | 2048bit-ECFP6 | KNN | 0.865 | 0.858 | 0.822 | 0.901 | 0.879 | 0.727 | 0.902 | 5 distance |
| M0248 | full | exp02 | 2048bit-ECFP6 | KNN | 0.876 | 0.904 | 0.890 | 0.865 | 0.884 | 0.753 | 0.891 | 5 uniform |
| M0249 | full | exp03 | 2048bit-ECFP6 | KNN | 0.869 | 0.864 | 0.831 | 0.901 | 0.882 | 0.735 | 0.889 | 5 distance |
| M0250 | full | exp04 | 2048bit-ECFP6 | KNN | 0.900 | 0.876 | 0.839 | 0.950 | 0.912 | 0.799 | 0.878 | 5 distance |
| M0251 | full | exp05 | 2048bit-ECFP6 | KNN | 0.915 | 0.922 | 0.907 | 0.922 | 0.922 | 0.829 | 0.888 | 3 uniform |
| M0252 | full | exp06 | 2048bit-ECFP6 | KNN | 0.869 | 0.896 | 0.881 | 0.858 | 0.877 | 0.737 | 0.892 | 3 distance |
| M0253 | full | exp07 | 2048bit-ECFP6 | KNN | 0.900 | 0.902 | 0.881 | 0.915 | 0.908 | 0.797 | 0.893 | 3 uniform |
| M0254 | full | exp08 | 2048bit-ECFP6 | KNN | 0.903 | 0.903 | 0.881 | 0.922 | 0.912 | 0.805 | 0.886 | 5 distance |
| M0255 | full | exp09 | 2048bit-ECFP6 | KNN | 0.919 | 0.911 | 0.890 | 0.943 | 0.927 | 0.837 | 0.888 | 5 uniform |
| M0256 | full | exp10 | 2048bit-ECFP6 | KNN | 0.892 | 0.869 | 0.831 | 0.943 | 0.905 | 0.784 | 0.887 | 5 distance |
| M0257 | full | exp11 | 2048bit-ECFP6 | KNN | 0.896 | 0.901 | 0.881 | 0.908 | 0.905 | 0.790 | 0.890 | 3 distance |
| M0258 | full | exp12 | 2048bit-ECFP6 | KNN | 0.869 | 0.869 | 0.839 | 0.894 | 0.881 | 0.735 | 0.889 | 5 distance |
| M0259 | full | exp13 | 2048bit-ECFP6 | KNN | 0.873 | 0.870 | 0.839 | 0.901 | 0.885 | 0.743 | 0.899 | 3 distance |
| M0260 | full | exp14 | 2048bit-ECFP6 | KNN | 0.915 | 0.905 | 0.881 | 0.943 | 0.924 | 0.829 | 0.892 | 3 distance |
| M0261 | full | exp15 | 2048bit-ECFP6 | KNN | 0.873 | 0.909 | 0.898 | 0.851 | 0.879 | 0.747 | 0.879 | 3 uniform |
| M0262 | full | exp16 | 2048bit-ECFP6 | KNN | 0.903 | 0.903 | 0.881 | 0.922 | 0.912 | 0.805 | 0.890 | 5 uniform |
| M0263 | full | exp17 | 2048bit-ECFP6 | KNN | 0.884 | 0.888 | 0.864 | 0.901 | 0.894 | 0.766 | 0.889 | 5 distance |
| M0264 | full | exp18 | 2048bit-ECFP6 | KNN | 0.865 | 0.879 | 0.856 | 0.872 | 0.875 | 0.728 | 0.888 | 9 distance |
| M0265 | full | exp19 | 2048bit-ECFP6 | KNN | 0.884 | 0.905 | 0.890 | 0.879 | 0.892 | 0.768 | 0.884 | 3 uniform |
| M0266 | full | exp01 | 2048bit-ECFP6 | SVM | 0.923 | 0.917 | 0.898 | 0.943 | 0.930 | 0.844 | 0.911 | 10 0.01 |
| M0267 | full | exp02 | 2048bit-ECFP6 | SVM | 0.892 | 0.884 | 0.856 | 0.922 | 0.903 | 0.782 | 0.918 | 5 0.01 |
| M0268 | full | exp03 | 2048bit-ECFP6 | SVM | 0.888 | 0.878 | 0.847 | 0.922 | 0.900 | 0.774 | 0.918 | 100 0.01 |
| M0269 | full | exp04 | 2048bit-ECFP6 | SVM | 0.903 | 0.892 | 0.864 | 0.936 | 0.913 | 0.806 | 0.910 | 10 0.01 |
| M0270 | full | exp05 | 2048bit-ECFP6 | SVM | 0.903 | 0.887 | 0.856 | 0.943 | 0.914 | 0.806 | 0.918 | 5 0.01 |
| M0271 | full | exp06 | 2048bit-ECFP6 | SVM | 0.919 | 0.935 | 0.924 | 0.915 | 0.925 | 0.837 | 0.915 | 5 0.01 |
| M0272 | full | exp07 | 2048bit-ECFP6 | SVM | 0.931 | 0.930 | 0.915 | 0.943 | 0.937 | 0.860 | 0.911 | 5 0.01 |
| M0273 | full | exp08 | 2048bit-ECFP6 | SVM | 0.923 | 0.923 | 0.907 | 0.936 | 0.930 | 0.844 | 0.910 | 10 0.01 |
| M0274 | full | exp09 | 2048bit-ECFP6 | SVM | 0.946 | 0.950 | 0.941 | 0.950 | 0.950 | 0.891 | 0.909 | 500 0.01 |
| M0275 | full | exp10 | 2048bit-ECFP6 | SVM | 0.911 | 0.888 | 0.856 | 0.957 | 0.922 | 0.823 | 0.912 | 10 0.01 |
| M0276 | full | exp11 | 2048bit-ECFP6 | SVM | 0.911 | 0.915 | 0.898 | 0.922 | 0.919 | 0.821 | 0.919 | 5 0.01 |
| M0277 | full | exp12 | 2048bit-ECFP6 | SVM | 0.865 | 0.853 | 0.814 | 0.908 | 0.880 | 0.728 | 0.921 | 1000.0 0.05 |
| M0278 | full | exp13 | 2048bit-ECFP6 | SVM | 0.892 | 0.890 | 0.864 | 0.915 | 0.902 | 0.782 | 0.915 | 5 0.01 |
| M0279 | full | exp14 | 2048bit-ECFP6 | SVM | 0.896 | 0.875 | 0.839 | 0.943 | 0.908 | 0.791 | 0.919 | 10 0.01 |
| M0280 | full | exp15 | 2048bit-ECFP6 | SVM | 0.876 | 0.852 | 0.805 | 0.936 | 0.892 | 0.753 | 0.903 | 5 0.05 |
| M0281 | full | exp16 | 2048bit-ECFP6 | SVM | 0.911 | 0.904 | 0.881 | 0.936 | 0.920 | 0.821 | 0.917 | 10 0.01 |
| M0282 | full | exp17 | 2048bit-ECFP6 | SVM | 0.923 | 0.912 | 0.890 | 0.950 | 0.931 | 0.845 | 0.919 | 1000.0 0.05 |
| M0283 | full | exp18 | 2048bit-ECFP6 | SVM | 0.903 | 0.914 | 0.898 | 0.908 | 0.911 | 0.806 | 0.917 | 5 0.01 |
| M0284 | full | exp19 | 2048bit-ECFP6 | SVM | 0.931 | 0.930 | 0.915 | 0.943 | 0.937 | 0.860 | 0.912 | 5 0.01 |
| M0285 | full | exp01 | 2048bit-ECFP6 | GBM | 0.884 | 0.863 | 0.822 | 0.936 | 0.898 | 0.768 | 0.890 | 100 0.1 |
| M0286 | full | exp02 | 2048bit-ECFP6 | GBM | 0.853 | 0.846 | 0.805 | 0.894 | 0.869 | 0.704 | 0.897 | 200 0.1 |
| M0287 | full | exp03 | 2048bit-ECFP6 | GBM | 0.888 | 0.873 | 0.839 | 0.929 | 0.900 | 0.775 | 0.899 | 100 0.5 |
| M0288 | full | exp04 | 2048bit-ECFP6 | GBM | 0.900 | 0.881 | 0.847 | 0.943 | 0.911 | 0.799 | 0.893 | 300 0.6 |
| M0289 | full | exp05 | 2048bit-ECFP6 | GBM | 0.892 | 0.874 | 0.839 | 0.936 | 0.904 | 0.783 | 0.892 | 400 0.3 |
| M0290 | full | exp06 | 2048bit-ECFP6 | GBM | 0.884 | 0.878 | 0.847 | 0.915 | 0.896 | 0.766 | 0.896 | 50 0.2 |
| M0291 | full | exp07 | 2048bit-ECFP6 | GBM | 0.911 | 0.904 | 0.881 | 0.936 | 0.920 | 0.821 | 0.891 | 50 0.4 |
| M0292 | full | exp08 | 2048bit-ECFP6 | GBM | 0.888 | 0.889 | 0.864 | 0.908 | 0.898 | 0.774 | 0.888 | 200 0.6 |
| M0293 | full | exp09 | 2048bit-ECFP6 | GBM | 0.934 | 0.931 | 0.915 | 0.950 | 0.940 | 0.868 | 0.885 | 400 0.3 |
| M0294 | full | exp10 | 2048bit-ECFP6 | GBM | 0.884 | 0.858 | 0.814 | 0.943 | 0.899 | 0.769 | 0.899 | 100 0.2 |
| M0295 | full | exp11 | 2048bit-ECFP6 | GBM | 0.896 | 0.907 | 0.890 | 0.901 | 0.904 | 0.790 | 0.904 | 100 0.2 |
| M0296 | full | exp12 | 2048bit-ECFP6 | GBM | 0.873 | 0.880 | 0.856 | 0.887 | 0.883 | 0.743 | 0.896 | 300 0.2 |
| M0297 | full | exp13 | 2048bit-ECFP6 | GBM | 0.869 | 0.869 | 0.839 | 0.894 | 0.881 | 0.735 | 0.906 | 700 0.1 |
| M0298 | full | exp14 | 2048bit-ECFP6 | GBM | 0.903 | 0.882 | 0.847 | 0.950 | 0.915 | 0.807 | 0.902 | 100 0.2 |
| M0299 | full | exp15 | 2048bit-ECFP6 | GBM | 0.888 | 0.878 | 0.847 | 0.922 | 0.900 | 0.774 | 0.884 | 600 0.2 |
| M0300 | full | exp16 | 2048bit-ECFP6 | GBM | 0.880 | 0.872 | 0.839 | 0.915 | 0.893 | 0.759 | 0.903 | 300 0.1 |
| M0301 | full | exp17 | 2048bit-ECFP6 | GBM | 0.896 | 0.913 | 0.898 | 0.894 | 0.903 | 0.790 | 0.894 | 200 0.3 |
| M0302 | full | exp18 | 2048bit-ECFP6 | GBM | 0.892 | 0.912 | 0.898 | 0.887 | 0.899 | 0.783 | 0.895 | 600 0.2 |
| M0303 | full | exp19 | 2048bit-ECFP6 | GBM | 0.923 | 0.912 | 0.890 | 0.950 | 0.931 | 0.845 | 0.889 | 200 0.3 |
| M0304 | full | exp01 | 2048bit-ECFP6 | RF | 0.880 | 0.872 | 0.839 | 0.915 | 0.893 | 0.759 | 0.914 | 500 |
| M0305 | full | exp02 | 2048bit-ECFP6 | RF | 0.888 | 0.868 | 0.831 | 0.936 | 0.901 | 0.775 | 0.911 | 600 |
| M0306 | full | exp03 | 2048bit-ECFP6 | RF | 0.903 | 0.882 | 0.847 | 0.950 | 0.915 | 0.807 | 0.918 | 900 |
| M0307 | full | exp04 | 2048bit-ECFP6 | RF | 0.900 | 0.881 | 0.847 | 0.943 | 0.911 | 0.799 | 0.908 | 200 |
| M0308 | full | exp05 | 2048bit-ECFP6 | RF | 0.919 | 0.895 | 0.864 | 0.965 | 0.928 | 0.838 | 0.910 | 900 |
| M0309 | full | exp06 | 2048bit-ECFP6 | RF | 0.903 | 0.903 | 0.881 | 0.922 | 0.912 | 0.805 | 0.915 | 600 |
| M0310 | full | exp07 | 2048bit-ECFP6 | RF | 0.915 | 0.889 | 0.856 | 0.965 | 0.925 | 0.831 | 0.902 | 500 |
| M0311 | full | exp08 | 2048bit-ECFP6 | RF | 0.915 | 0.899 | 0.873 | 0.950 | 0.924 | 0.829 | 0.908 | 100 |
| M0312 | full | exp09 | 2048bit-ECFP6 | RF | 0.954 | 0.939 | 0.924 | 0.979 | 0.958 | 0.907 | 0.895 | 1000 |
| M0313 | full | exp10 | 2048bit-ECFP6 | RF | 0.896 | 0.852 | 0.797 | 0.979 | 0.911 | 0.798 | 0.911 | 50 |
| M0314 | full | exp11 | 2048bit-ECFP6 | RF | 0.919 | 0.900 | 0.873 | 0.957 | 0.928 | 0.838 | 0.910 | 1000 |
| M0315 | full | exp12 | 2048bit-ECFP6 | RF | 0.903 | 0.892 | 0.864 | 0.936 | 0.913 | 0.806 | 0.912 | 600 |
| M0316 | full | exp13 | 2048bit-ECFP6 | RF | 0.896 | 0.880 | 0.847 | 0.936 | 0.907 | 0.790 | 0.918 | 400 |
| M0317 | full | exp14 | 2048bit-ECFP6 | RF | 0.900 | 0.871 | 0.831 | 0.957 | 0.912 | 0.801 | 0.920 | 800 |
| M0318 | full | exp15 | 2048bit-ECFP6 | RF | 0.896 | 0.880 | 0.847 | 0.936 | 0.907 | 0.790 | 0.906 | 300 |
| M0319 | full | exp16 | 2048bit-ECFP6 | RF | 0.911 | 0.888 | 0.856 | 0.957 | 0.922 | 0.823 | 0.909 | 50 |
| M0320 | full | exp17 | 2048bit-ECFP6 | RF | 0.903 | 0.908 | 0.890 | 0.915 | 0.912 | 0.805 | 0.913 | 600 |
| M0321 | full | exp18 | 2048bit-ECFP6 | RF | 0.915 | 0.922 | 0.907 | 0.922 | 0.922 | 0.829 | 0.903 | 200 |
| M0322 | full | exp19 | 2048bit-ECFP6 | RF | 0.923 | 0.901 | 0.873 | 0.965 | 0.932 | 0.846 | 0.916 | 400 |
| M0323 | full | exp01 | 2048bit-ECFP6 | DNN2 | 0.900 | 0.897 | 0.873 | 0.922 | 0.909 | 0.797 | 0.915 | 500 60 0.1 |
| M0324 | full | exp02 | 2048bit-ECFP6 | DNN2 | 0.907 | 0.909 | 0.890 | 0.922 | 0.915 | 0.813 | 0.920 | 100 120 0.5 |
| M0325 | full | exp03 | 2048bit-ECFP6 | DNN2 | 0.880 | 0.867 | 0.831 | 0.922 | 0.893 | 0.759 | 0.920 | 100 60 0.3 |
| M0326 | full | exp01 | 2048bit-ECFP6 | DNN3 | 0.880 | 0.857 | 0.814 | 0.936 | 0.895 | 0.761 | 0.883 | 100 60 0.5 |
| M0327 | full | exp02 | 2048bit-ECFP6 | DNN3 | 0.869 | 0.841 | 0.788 | 0.936 | 0.886 | 0.738 | 0.878 | 100 80 0.5 |
| M0328 | full | exp03 | 2048bit-ECFP6 | DNN3 | 0.822 | 0.760 | 0.627 | 0.986 | 0.858 | 0.670 | 0.814 | 100 60 0.5 |
| M0329 | 512 | exp01 | 1024bit-ECFP4 | KNN | 0.873 | 0.886 | 0.864 | 0.879 | 0.883 | 0.743 | 0.911 | 3 distance |
| M0330 | 512 | exp02 | 1024bit-ECFP4 | KNN | 0.900 | 0.932 | 0.924 | 0.879 | 0.905 | 0.800 | 0.910 | 3 uniform |
| M0331 | 512 | exp03 | 1024bit-ECFP4 | KNN | 0.880 | 0.882 | 0.856 | 0.901 | 0.891 | 0.758 | 0.911 | 7 distance |
| M0332 | 512 | exp04 | 1024bit-ECFP4 | KNN | 0.880 | 0.872 | 0.839 | 0.915 | 0.893 | 0.759 | 0.902 | 5 distance |
| M0333 | 512 | exp05 | 1024bit-ECFP4 | KNN | 0.915 | 0.905 | 0.881 | 0.943 | 0.924 | 0.829 | 0.902 | 3 uniform |
| M0334 | 512 | exp06 | 1024bit-ECFP4 | KNN | 0.900 | 0.914 | 0.898 | 0.901 | 0.907 | 0.798 | 0.908 | 3 uniform |
| M0335 | 512 | exp07 | 1024bit-ECFP4 | KNN | 0.911 | 0.899 | 0.873 | 0.943 | 0.920 | 0.821 | 0.900 | 3 uniform |
| M0336 | 512 | exp08 | 1024bit-ECFP4 | KNN | 0.907 | 0.921 | 0.907 | 0.908 | 0.914 | 0.814 | 0.906 | 5 distance |
| M0337 | 512 | exp09 | 1024bit-ECFP4 | KNN | 0.915 | 0.928 | 0.915 | 0.915 | 0.921 | 0.829 | 0.904 | 3 distance |
| M0338 | 512 | exp10 | 1024bit-ECFP4 | KNN | 0.888 | 0.878 | 0.847 | 0.922 | 0.900 | 0.774 | 0.903 | 5 distance |
| M0339 | 512 | exp11 | 1024bit-ECFP4 | KNN | 0.911 | 0.910 | 0.890 | 0.929 | 0.919 | 0.821 | 0.902 | 5 distance |
| M0340 | 512 | exp12 | 1024bit-ECFP4 | KNN | 0.900 | 0.914 | 0.898 | 0.901 | 0.907 | 0.798 | 0.916 | 5 distance |
| M0341 | 512 | exp13 | 1024bit-ECFP4 | KNN | 0.896 | 0.880 | 0.847 | 0.936 | 0.907 | 0.790 | 0.916 | 3 distance |
| M0342 | 512 | exp14 | 1024bit-ECFP4 | KNN | 0.900 | 0.891 | 0.864 | 0.929 | 0.910 | 0.798 | 0.904 | 5 distance |
| M0343 | 512 | exp15 | 1024bit-ECFP4 | KNN | 0.915 | 0.916 | 0.898 | 0.929 | 0.923 | 0.829 | 0.897 | 5 distance |
| M0344 | 512 | exp16 | 1024bit-ECFP4 | KNN | 0.919 | 0.917 | 0.898 | 0.936 | 0.926 | 0.836 | 0.901 | 3 distance |
| M0345 | 512 | exp17 | 1024bit-ECFP4 | KNN | 0.907 | 0.927 | 0.915 | 0.901 | 0.914 | 0.814 | 0.918 | 3 uniform |
| M0346 | 512 | exp18 | 1024bit-ECFP4 | KNN | 0.911 | 0.928 | 0.915 | 0.908 | 0.918 | 0.822 | 0.900 | 3 distance |
| M0347 | 512 | exp19 | 1024bit-ECFP4 | KNN | 0.911 | 0.921 | 0.907 | 0.915 | 0.918 | 0.821 | 0.906 | 3 uniform |
| M0348 | 512 | exp01 | 1024bit-ECFP4 | SVM | 0.911 | 0.910 | 0.890 | 0.929 | 0.919 | 0.821 | 0.920 | 5 0.1 |
| M0349 | 512 | exp02 | 1024bit-ECFP4 | SVM | 0.896 | 0.901 | 0.881 | 0.908 | 0.905 | 0.790 | 0.922 | 500 0.05 |
| M0350 | 512 | exp03 | 1024bit-ECFP4 | SVM | 0.911 | 0.915 | 0.898 | 0.922 | 0.919 | 0.821 | 0.929 | 10 0.05 |
| M0351 | 512 | exp04 | 1024bit-ECFP4 | SVM | 0.915 | 0.894 | 0.864 | 0.957 | 0.925 | 0.830 | 0.917 | 10 0.05 |
| M0352 | 512 | exp05 | 1024bit-ECFP4 | SVM | 0.923 | 0.901 | 0.873 | 0.965 | 0.932 | 0.846 | 0.921 | 500 0.05 |
| M0353 | 512 | exp06 | 1024bit-ECFP4 | SVM | 0.927 | 0.924 | 0.907 | 0.943 | 0.933 | 0.852 | 0.919 | 1 0.1 |
| M0354 | 512 | exp07 | 1024bit-ECFP4 | SVM | 0.915 | 0.879 | 0.839 | 0.979 | 0.926 | 0.833 | 0.913 | 5 0.1 |
| M0355 | 512 | exp08 | 1024bit-ECFP4 | SVM | 0.931 | 0.930 | 0.915 | 0.943 | 0.937 | 0.860 | 0.916 | 5 0.05 |
| M0356 | 512 | exp09 | 1024bit-ECFP4 | SVM | 0.942 | 0.950 | 0.941 | 0.943 | 0.947 | 0.883 | 0.909 | 1000.0 0.05 |
| M0357 | 512 | exp10 | 1024bit-ECFP4 | SVM | 0.903 | 0.872 | 0.831 | 0.965 | 0.916 | 0.809 | 0.910 | 1 0.05 |
| M0358 | 512 | exp11 | 1024bit-ECFP4 | SVM | 0.915 | 0.922 | 0.907 | 0.922 | 0.922 | 0.829 | 0.917 | 500 0.05 |
| M0359 | 512 | exp12 | 1024bit-ECFP4 | SVM | 0.903 | 0.903 | 0.881 | 0.922 | 0.912 | 0.805 | 0.922 | 10 0.1 |
| M0360 | 512 | exp13 | 1024bit-ECFP4 | SVM | 0.903 | 0.903 | 0.881 | 0.922 | 0.912 | 0.805 | 0.933 | 5 0.05 |
| M0361 | 512 | exp14 | 1024bit-ECFP4 | SVM | 0.888 | 0.884 | 0.856 | 0.915 | 0.899 | 0.774 | 0.929 | 5 0.05 |
| M0362 | 512 | exp15 | 1024bit-ECFP4 | SVM | 0.896 | 0.875 | 0.839 | 0.943 | 0.908 | 0.791 | 0.903 | 100000.0 0.05 |
| M0363 | 512 | exp16 | 1024bit-ECFP4 | SVM | 0.934 | 0.925 | 0.907 | 0.957 | 0.941 | 0.868 | 0.917 | 500 0.05 |
| M0364 | 512 | exp17 | 1024bit-ECFP4 | SVM | 0.915 | 0.916 | 0.898 | 0.929 | 0.923 | 0.829 | 0.921 | 10 0.1 |
| M0365 | 512 | exp18 | 1024bit-ECFP4 | SVM | 0.903 | 0.920 | 0.907 | 0.901 | 0.910 | 0.806 | 0.918 | 10000.0 0.05 |
| M0366 | 512 | exp19 | 1024bit-ECFP4 | SVM | 0.946 | 0.944 | 0.932 | 0.957 | 0.951 | 0.891 | 0.920 | 10 0.05 |
| M0367 | 512 | exp01 | 1024bit-ECFP4 | GBM | 0.876 | 0.881 | 0.856 | 0.894 | 0.887 | 0.751 | 0.901 | 200 0.3 |
| M0368 | 512 | exp02 | 1024bit-ECFP4 | GBM | 0.865 | 0.868 | 0.839 | 0.887 | 0.877 | 0.727 | 0.903 | 300 0.8 |
| M0369 | 512 | exp03 | 1024bit-ECFP4 | GBM | 0.888 | 0.889 | 0.864 | 0.908 | 0.898 | 0.774 | 0.916 | 900 0.2 |
| M0370 | 512 | exp04 | 1024bit-ECFP4 | GBM | 0.892 | 0.890 | 0.864 | 0.915 | 0.902 | 0.782 | 0.899 | 500 0.3 |
| M0371 | 512 | exp05 | 1024bit-ECFP4 | GBM | 0.919 | 0.900 | 0.873 | 0.957 | 0.928 | 0.838 | 0.914 | 300 0.3 |
| M0372 | 512 | exp06 | 1024bit-ECFP4 | GBM | 0.931 | 0.936 | 0.924 | 0.936 | 0.936 | 0.860 | 0.905 | 300 0.3 |
| M0373 | 512 | exp07 | 1024bit-ECFP4 | GBM | 0.876 | 0.871 | 0.839 | 0.908 | 0.889 | 0.751 | 0.906 | 900 0.1 |
| M0374 | 512 | exp08 | 1024bit-ECFP4 | GBM | 0.907 | 0.898 | 0.873 | 0.936 | 0.917 | 0.813 | 0.905 | 200 0.2 |
| M0375 | 512 | exp09 | 1024bit-ECFP4 | GBM | 0.911 | 0.921 | 0.907 | 0.915 | 0.918 | 0.821 | 0.893 | 300 0.1 |
| M0376 | 512 | exp10 | 1024bit-ECFP4 | GBM | 0.892 | 0.869 | 0.831 | 0.943 | 0.905 | 0.784 | 0.901 | 400 0.1 |
| M0377 | 512 | exp11 | 1024bit-ECFP4 | GBM | 0.907 | 0.915 | 0.898 | 0.915 | 0.915 | 0.813 | 0.906 | 200 0.2 |
| M0378 | 512 | exp12 | 1024bit-ECFP4 | GBM | 0.888 | 0.900 | 0.881 | 0.894 | 0.897 | 0.774 | 0.900 | 100 0.2 |
| M0379 | 512 | exp13 | 1024bit-ECFP4 | GBM | 0.888 | 0.884 | 0.856 | 0.915 | 0.899 | 0.774 | 0.908 | 600 0.1 |
| M0380 | 512 | exp14 | 1024bit-ECFP4 | GBM | 0.876 | 0.866 | 0.831 | 0.915 | 0.890 | 0.751 | 0.912 | 300 0.4 |
| M0381 | 512 | exp15 | 1024bit-ECFP4 | GBM | 0.880 | 0.877 | 0.847 | 0.908 | 0.892 | 0.758 | 0.902 | 500 0.3 |
| M0382 | 512 | exp16 | 1024bit-ECFP4 | GBM | 0.907 | 0.903 | 0.881 | 0.929 | 0.916 | 0.813 | 0.899 | 200 0.8 |
| M0383 | 512 | exp17 | 1024bit-ECFP4 | GBM | 0.911 | 0.915 | 0.898 | 0.922 | 0.919 | 0.821 | 0.902 | 300 0.5 |
| M0384 | 512 | exp18 | 1024bit-ECFP4 | GBM | 0.892 | 0.919 | 0.907 | 0.879 | 0.899 | 0.784 | 0.908 | 400 0.2 |
| M0385 | 512 | exp19 | 1024bit-ECFP4 | GBM | 0.927 | 0.942 | 0.932 | 0.922 | 0.932 | 0.853 | 0.902 | 500 0.1 |
| M0386 | 512 | exp01 | 1024bit-ECFP4 | RF | 0.888 | 0.878 | 0.847 | 0.922 | 0.900 | 0.774 | 0.921 | 600 |
| M0387 | 512 | exp02 | 1024bit-ECFP4 | RF | 0.892 | 0.874 | 0.839 | 0.936 | 0.904 | 0.783 | 0.920 | 500 |
| M0388 | 512 | exp03 | 1024bit-ECFP4 | RF | 0.888 | 0.868 | 0.831 | 0.936 | 0.901 | 0.775 | 0.917 | 1000 |
| M0389 | 512 | exp04 | 1024bit-ECFP4 | RF | 0.903 | 0.892 | 0.864 | 0.936 | 0.913 | 0.806 | 0.913 | 200 |
| M0390 | 512 | exp05 | 1024bit-ECFP4 | RF | 0.919 | 0.890 | 0.856 | 0.972 | 0.929 | 0.839 | 0.922 | 300 |
| M0391 | 512 | exp06 | 1024bit-ECFP4 | RF | 0.934 | 0.931 | 0.915 | 0.950 | 0.940 | 0.868 | 0.919 | 1000 |
| M0392 | 512 | exp07 | 1024bit-ECFP4 | RF | 0.915 | 0.894 | 0.864 | 0.957 | 0.925 | 0.830 | 0.906 | 200 |
| M0393 | 512 | exp08 | 1024bit-ECFP4 | RF | 0.934 | 0.925 | 0.907 | 0.957 | 0.941 | 0.868 | 0.914 | 1000 |
| M0394 | 512 | exp09 | 1024bit-ECFP4 | RF | 0.958 | 0.939 | 0.924 | 0.986 | 0.962 | 0.915 | 0.905 | 300 |
| M0395 | 512 | exp10 | 1024bit-ECFP4 | RF | 0.903 | 0.872 | 0.831 | 0.965 | 0.916 | 0.809 | 0.917 | 500 |
| M0396 | 512 | exp11 | 1024bit-ECFP4 | RF | 0.923 | 0.917 | 0.898 | 0.943 | 0.930 | 0.844 | 0.911 | 500 |
| M0397 | 512 | exp12 | 1024bit-ECFP4 | RF | 0.915 | 0.910 | 0.890 | 0.936 | 0.923 | 0.829 | 0.916 | 400 |
| M0398 | 512 | exp13 | 1024bit-ECFP4 | RF | 0.903 | 0.892 | 0.864 | 0.936 | 0.913 | 0.806 | 0.920 | 100 |
| M0399 | 512 | exp14 | 1024bit-ECFP4 | RF | 0.900 | 0.876 | 0.839 | 0.950 | 0.912 | 0.799 | 0.925 | 50 |
| M0400 | 512 | exp15 | 1024bit-ECFP4 | RF | 0.888 | 0.873 | 0.839 | 0.929 | 0.900 | 0.775 | 0.907 | 700 |
| M0401 | 512 | exp16 | 1024bit-ECFP4 | RF | 0.931 | 0.918 | 0.898 | 0.957 | 0.938 | 0.860 | 0.916 | 100 |
| M0402 | 512 | exp17 | 1024bit-ECFP4 | RF | 0.907 | 0.915 | 0.898 | 0.915 | 0.915 | 0.813 | 0.914 | 400 |
| M0403 | 512 | exp18 | 1024bit-ECFP4 | RF | 0.915 | 0.928 | 0.915 | 0.915 | 0.921 | 0.829 | 0.905 | 700 |
| M0404 | 512 | exp19 | 1024bit-ECFP4 | RF | 0.938 | 0.925 | 0.907 | 0.965 | 0.944 | 0.876 | 0.917 | 1000 |
| M0405 | 512 | exp01 | 1024bit-ECFP4 | DNN2 | 0.903 | 0.920 | 0.907 | 0.901 | 0.910 | 0.806 | 0.918 | 200 160 0.2 |
| M0406 | 512 | exp02 | 1024bit-ECFP4 | DNN2 | 0.896 | 0.901 | 0.881 | 0.908 | 0.905 | 0.790 | 0.917 | 400 120 0.4 |
| M0407 | 512 | exp03 | 1024bit-ECFP4 | DNN2 | 0.888 | 0.900 | 0.881 | 0.894 | 0.897 | 0.774 | 0.926 | 600 100 0.4 |
| M0408 | 512 | exp01 | 1024bit-ECFP4 | DNN3 | 0.903 | 0.914 | 0.898 | 0.908 | 0.911 | 0.806 | 0.917 | 100 100 0.1 |
| M0409 | 512 | exp02 | 1024bit-ECFP4 | DNN3 | 0.888 | 0.912 | 0.898 | 0.879 | 0.895 | 0.776 | 0.917 | 100 100 0.5 |
| M0410 | 512 | exp03 | 1024bit-ECFP4 | DNN3 | 0.888 | 0.906 | 0.890 | 0.887 | 0.896 | 0.775 | 0.923 | 400 140 0.4 |
| M0411 | 512 | exp01 | 2048bit-ECFP4 | KNN | 0.873 | 0.855 | 0.814 | 0.922 | 0.887 | 0.744 | 0.927 | 5 distance |
| M0412 | 512 | exp02 | 2048bit-ECFP4 | KNN | 0.884 | 0.905 | 0.890 | 0.879 | 0.892 | 0.768 | 0.911 | 3 uniform |
| M0413 | 512 | exp03 | 2048bit-ECFP4 | KNN | 0.911 | 0.904 | 0.881 | 0.936 | 0.920 | 0.821 | 0.915 | 3 distance |
| M0414 | 512 | exp04 | 2048bit-ECFP4 | KNN | 0.911 | 0.888 | 0.856 | 0.957 | 0.922 | 0.823 | 0.920 | 3 uniform |
| M0415 | 512 | exp05 | 2048bit-ECFP4 | KNN | 0.934 | 0.913 | 0.890 | 0.972 | 0.942 | 0.869 | 0.922 | 3 distance |
| M0416 | 512 | exp06 | 2048bit-ECFP4 | KNN | 0.915 | 0.934 | 0.924 | 0.908 | 0.921 | 0.830 | 0.916 | 3 uniform |
| M0417 | 512 | exp07 | 2048bit-ECFP4 | KNN | 0.915 | 0.905 | 0.881 | 0.943 | 0.924 | 0.829 | 0.907 | 5 distance |
| M0418 | 512 | exp08 | 2048bit-ECFP4 | KNN | 0.915 | 0.905 | 0.881 | 0.943 | 0.924 | 0.829 | 0.907 | 5 distance |
| M0419 | 512 | exp09 | 2048bit-ECFP4 | KNN | 0.938 | 0.937 | 0.924 | 0.950 | 0.944 | 0.875 | 0.907 | 3 uniform |
| M0420 | 512 | exp10 | 2048bit-ECFP4 | KNN | 0.900 | 0.886 | 0.856 | 0.936 | 0.910 | 0.798 | 0.908 | 5 distance |
| M0421 | 512 | exp11 | 2048bit-ECFP4 | KNN | 0.919 | 0.929 | 0.915 | 0.922 | 0.925 | 0.837 | 0.910 | 1 uniform |
| M0422 | 512 | exp12 | 2048bit-ECFP4 | KNN | 0.907 | 0.915 | 0.898 | 0.915 | 0.915 | 0.813 | 0.924 | 3 uniform |
| M0423 | 512 | exp13 | 2048bit-ECFP4 | KNN | 0.911 | 0.910 | 0.890 | 0.929 | 0.919 | 0.821 | 0.918 | 1 uniform |
| M0424 | 512 | exp14 | 2048bit-ECFP4 | KNN | 0.900 | 0.886 | 0.856 | 0.936 | 0.910 | 0.798 | 0.912 | 5 distance |
| M0425 | 512 | exp15 | 2048bit-ECFP4 | KNN | 0.896 | 0.896 | 0.873 | 0.915 | 0.905 | 0.790 | 0.907 | 5 distance |
| M0426 | 512 | exp16 | 2048bit-ECFP4 | KNN | 0.931 | 0.918 | 0.898 | 0.957 | 0.938 | 0.860 | 0.914 | 3 uniform |
| M0427 | 512 | exp17 | 2048bit-ECFP4 | KNN | 0.923 | 0.935 | 0.924 | 0.922 | 0.929 | 0.845 | 0.925 | 3 uniform |
| M0428 | 512 | exp18 | 2048bit-ECFP4 | KNN | 0.923 | 0.942 | 0.932 | 0.915 | 0.928 | 0.845 | 0.908 | 1 uniform |
| M0429 | 512 | exp19 | 2048bit-ECFP4 | KNN | 0.934 | 0.949 | 0.941 | 0.929 | 0.939 | 0.868 | 0.913 | 3 uniform |
| M0430 | 512 | exp01 | 2048bit-ECFP4 | SVM | 0.907 | 0.909 | 0.890 | 0.922 | 0.915 | 0.813 | 0.924 | 1 0.1 |
| M0431 | 512 | exp02 | 2048bit-ECFP4 | SVM | 0.903 | 0.903 | 0.881 | 0.922 | 0.912 | 0.805 | 0.924 | 5 0.1 |
| M0432 | 512 | exp03 | 2048bit-ECFP4 | SVM | 0.927 | 0.918 | 0.898 | 0.950 | 0.934 | 0.852 | 0.932 | 10000.0 0.1 |
| M0433 | 512 | exp04 | 2048bit-ECFP4 | SVM | 0.907 | 0.893 | 0.864 | 0.943 | 0.917 | 0.814 | 0.917 | 500 0.1 |
| M0434 | 512 | exp05 | 2048bit-ECFP4 | SVM | 0.911 | 0.888 | 0.856 | 0.957 | 0.922 | 0.823 | 0.923 | 10000.0 0.05 |
| M0435 | 512 | exp06 | 2048bit-ECFP4 | SVM | 0.927 | 0.936 | 0.924 | 0.929 | 0.932 | 0.852 | 0.924 | 1 0.1 |
| M0436 | 512 | exp07 | 2048bit-ECFP4 | SVM | 0.923 | 0.895 | 0.864 | 0.972 | 0.932 | 0.847 | 0.909 | 10 0.1 |
| M0437 | 512 | exp08 | 2048bit-ECFP4 | SVM | 0.931 | 0.942 | 0.932 | 0.929 | 0.936 | 0.860 | 0.914 | 500 0.1 |
| M0438 | 512 | exp09 | 2048bit-ECFP4 | SVM | 0.946 | 0.950 | 0.941 | 0.950 | 0.950 | 0.891 | 0.912 | 1000.0 0.1 |
| M0439 | 512 | exp10 | 2048bit-ECFP4 | SVM | 0.907 | 0.877 | 0.839 | 0.965 | 0.919 | 0.816 | 0.920 | 10 0.1 |
| M0440 | 512 | exp11 | 2048bit-ECFP4 | SVM | 0.911 | 0.910 | 0.890 | 0.929 | 0.919 | 0.821 | 0.919 | 50 0.01 |
| M0441 | 512 | exp12 | 2048bit-ECFP4 | SVM | 0.911 | 0.921 | 0.907 | 0.915 | 0.918 | 0.821 | 0.931 | 1 0.1 |
| M0442 | 512 | exp13 | 2048bit-ECFP4 | SVM | 0.907 | 0.898 | 0.873 | 0.936 | 0.917 | 0.813 | 0.931 | 1 0.1 |
| M0443 | 512 | exp14 | 2048bit-ECFP4 | SVM | 0.907 | 0.882 | 0.847 | 0.957 | 0.918 | 0.815 | 0.929 | 500 0.05 |
| M0444 | 512 | exp15 | 2048bit-ECFP4 | SVM | 0.903 | 0.892 | 0.864 | 0.936 | 0.913 | 0.806 | 0.911 | 1 0.1 |
| M0445 | 512 | exp16 | 2048bit-ECFP4 | SVM | 0.931 | 0.918 | 0.898 | 0.957 | 0.938 | 0.860 | 0.928 | 10 0.1 |
| M0446 | 512 | exp17 | 2048bit-ECFP4 | SVM | 0.919 | 0.935 | 0.924 | 0.915 | 0.925 | 0.837 | 0.927 | 100 0.1 |
| M0447 | 512 | exp18 | 2048bit-ECFP4 | SVM | 0.915 | 0.922 | 0.907 | 0.922 | 0.922 | 0.829 | 0.922 | 1000.0 0.05 |
| M0448 | 512 | exp19 | 2048bit-ECFP4 | SVM | 0.923 | 0.917 | 0.898 | 0.943 | 0.930 | 0.844 | 0.921 | 1 0.05 |
| M0449 | 512 | exp01 | 2048bit-ECFP4 | GBM | 0.900 | 0.897 | 0.873 | 0.922 | 0.909 | 0.797 | 0.907 | 200 0.9 |
| M0450 | 512 | exp02 | 2048bit-ECFP4 | GBM | 0.880 | 0.857 | 0.814 | 0.936 | 0.895 | 0.761 | 0.907 | 400 0.2 |
| M0451 | 512 | exp03 | 2048bit-ECFP4 | GBM | 0.892 | 0.901 | 0.881 | 0.901 | 0.901 | 0.782 | 0.915 | 100 0.4 |
| M0452 | 512 | exp04 | 2048bit-ECFP4 | GBM | 0.903 | 0.882 | 0.847 | 0.950 | 0.915 | 0.807 | 0.900 | 300 0.5 |
| M0453 | 512 | exp05 | 2048bit-ECFP4 | GBM | 0.896 | 0.880 | 0.847 | 0.936 | 0.907 | 0.790 | 0.914 | 200 0.4 |
| M0454 | 512 | exp06 | 2048bit-ECFP4 | GBM | 0.903 | 0.926 | 0.915 | 0.894 | 0.910 | 0.807 | 0.902 | 300 0.6 |
| M0455 | 512 | exp07 | 2048bit-ECFP4 | GBM | 0.896 | 0.880 | 0.847 | 0.936 | 0.907 | 0.790 | 0.907 | 700 0.3 |
| M0456 | 512 | exp08 | 2048bit-ECFP4 | GBM | 0.911 | 0.899 | 0.873 | 0.943 | 0.920 | 0.821 | 0.896 | 400 0.1 |
| M0457 | 512 | exp09 | 2048bit-ECFP4 | GBM | 0.942 | 0.938 | 0.924 | 0.957 | 0.947 | 0.883 | 0.899 | 700 0.1 |
| M0458 | 512 | exp10 | 2048bit-ECFP4 | GBM | 0.892 | 0.865 | 0.822 | 0.950 | 0.905 | 0.785 | 0.904 | 200 0.2 |
| M0459 | 512 | exp11 | 2048bit-ECFP4 | GBM | 0.900 | 0.902 | 0.881 | 0.915 | 0.908 | 0.797 | 0.910 | 400 0.1 |
| M0460 | 512 | exp12 | 2048bit-ECFP4 | GBM | 0.884 | 0.888 | 0.864 | 0.901 | 0.894 | 0.766 | 0.908 | 100 0.4 |
| M0461 | 512 | exp13 | 2048bit-ECFP4 | GBM | 0.888 | 0.878 | 0.847 | 0.922 | 0.900 | 0.774 | 0.909 | 200 0.3 |
| M0462 | 512 | exp14 | 2048bit-ECFP4 | GBM | 0.880 | 0.867 | 0.831 | 0.922 | 0.893 | 0.759 | 0.906 | 400 0.4 |
| M0463 | 512 | exp15 | 2048bit-ECFP4 | GBM | 0.892 | 0.874 | 0.839 | 0.936 | 0.904 | 0.783 | 0.897 | 300 0.4 |
| M0464 | 512 | exp16 | 2048bit-ECFP4 | GBM | 0.903 | 0.908 | 0.890 | 0.915 | 0.912 | 0.805 | 0.908 | 200 0.2 |
| M0465 | 512 | exp17 | 2048bit-ECFP4 | GBM | 0.892 | 0.901 | 0.881 | 0.901 | 0.901 | 0.782 | 0.912 | 300 0.1 |
| M0466 | 512 | exp18 | 2048bit-ECFP4 | GBM | 0.896 | 0.907 | 0.890 | 0.901 | 0.904 | 0.790 | 0.911 | 200 0.3 |
| M0467 | 512 | exp19 | 2048bit-ECFP4 | GBM | 0.919 | 0.900 | 0.873 | 0.957 | 0.928 | 0.838 | 0.905 | 50 0.5 |
| M0468 | 512 | exp01 | 2048bit-ECFP4 | RF | 0.892 | 0.884 | 0.856 | 0.922 | 0.903 | 0.782 | 0.921 | 200 |
| M0469 | 512 | exp02 | 2048bit-ECFP4 | RF | 0.880 | 0.862 | 0.822 | 0.929 | 0.894 | 0.760 | 0.919 | 700 |
| M0470 | 512 | exp03 | 2048bit-ECFP4 | RF | 0.892 | 0.860 | 0.814 | 0.957 | 0.906 | 0.786 | 0.925 | 800 |
| M0471 | 512 | exp04 | 2048bit-ECFP4 | RF | 0.911 | 0.893 | 0.864 | 0.950 | 0.921 | 0.822 | 0.916 | 200 |
| M0472 | 512 | exp05 | 2048bit-ECFP4 | RF | 0.915 | 0.884 | 0.847 | 0.972 | 0.926 | 0.832 | 0.917 | 600 |
| M0473 | 512 | exp06 | 2048bit-ECFP4 | RF | 0.911 | 0.921 | 0.907 | 0.915 | 0.918 | 0.821 | 0.922 | 200 |
| M0474 | 512 | exp07 | 2048bit-ECFP4 | RF | 0.907 | 0.882 | 0.847 | 0.957 | 0.918 | 0.815 | 0.908 | 200 |
| M0475 | 512 | exp08 | 2048bit-ECFP4 | RF | 0.931 | 0.918 | 0.898 | 0.957 | 0.938 | 0.860 | 0.913 | 500 |
| M0476 | 512 | exp09 | 2048bit-ECFP4 | RF | 0.942 | 0.932 | 0.915 | 0.965 | 0.948 | 0.884 | 0.908 | 200 |
| M0477 | 512 | exp10 | 2048bit-ECFP4 | RF | 0.907 | 0.877 | 0.839 | 0.965 | 0.919 | 0.816 | 0.918 | 900 |
| M0478 | 512 | exp11 | 2048bit-ECFP4 | RF | 0.923 | 0.917 | 0.898 | 0.943 | 0.930 | 0.844 | 0.918 | 800 |
| M0479 | 512 | exp12 | 2048bit-ECFP4 | RF | 0.903 | 0.903 | 0.881 | 0.922 | 0.912 | 0.805 | 0.925 | 200 |
| M0480 | 512 | exp13 | 2048bit-ECFP4 | RF | 0.900 | 0.886 | 0.856 | 0.936 | 0.910 | 0.798 | 0.920 | 50 |
| M0481 | 512 | exp14 | 2048bit-ECFP4 | RF | 0.903 | 0.872 | 0.831 | 0.965 | 0.916 | 0.809 | 0.928 | 700 |
| M0482 | 512 | exp15 | 2048bit-ECFP4 | RF | 0.900 | 0.886 | 0.856 | 0.936 | 0.910 | 0.798 | 0.908 | 400 |
| M0483 | 512 | exp16 | 2048bit-ECFP4 | RF | 0.923 | 0.906 | 0.881 | 0.957 | 0.931 | 0.845 | 0.918 | 600 |
| M0484 | 512 | exp17 | 2048bit-ECFP4 | RF | 0.903 | 0.908 | 0.890 | 0.915 | 0.912 | 0.805 | 0.921 | 100 |
| M0485 | 512 | exp18 | 2048bit-ECFP4 | RF | 0.903 | 0.920 | 0.907 | 0.901 | 0.910 | 0.806 | 0.917 | 300 |
| M0486 | 512 | exp19 | 2048bit-ECFP4 | RF | 0.934 | 0.919 | 0.898 | 0.965 | 0.941 | 0.868 | 0.917 | 800 |
| M0487 | 512 | exp01 | 2048bit-ECFP4 | DNN2 | 0.903 | 0.920 | 0.907 | 0.901 | 0.910 | 0.806 | 0.929 | 100 160 0.5 |
| M0488 | 512 | exp02 | 2048bit-ECFP4 | DNN2 | 0.888 | 0.889 | 0.864 | 0.908 | 0.898 | 0.774 | 0.925 | 600 120 0.5 |
| M0489 | 512 | exp03 | 2048bit-ECFP4 | DNN2 | 0.903 | 0.914 | 0.898 | 0.908 | 0.911 | 0.806 | 0.928 | 100 140 0.5 |
| M0490 | 512 | exp01 | 2048bit-ECFP4 | DNN3 | 0.907 | 0.927 | 0.915 | 0.901 | 0.914 | 0.814 | 0.926 | 400 160 0.4 |
| M0491 | 512 | exp02 | 2048bit-ECFP4 | DNN3 | 0.880 | 0.882 | 0.856 | 0.901 | 0.891 | 0.758 | 0.923 | 500 140 0.3 |
| M0492 | 512 | exp03 | 2048bit-ECFP4 | DNN3 | 0.903 | 0.903 | 0.881 | 0.922 | 0.912 | 0.805 | 0.926 | 300 60 0.3 |
| M0493 | 512 | exp01 | 1024bit-ECFP6 | KNN | 0.861 | 0.867 | 0.839 | 0.879 | 0.873 | 0.719 | 0.914 | 3 uniform |
| M0494 | 512 | exp02 | 1024bit-ECFP6 | KNN | 0.876 | 0.871 | 0.839 | 0.908 | 0.889 | 0.751 | 0.906 | 7 uniform |
| M0495 | 512 | exp03 | 1024bit-ECFP6 | KNN | 0.892 | 0.879 | 0.847 | 0.929 | 0.903 | 0.782 | 0.914 | 5 distance |
| M0496 | 512 | exp04 | 1024bit-ECFP6 | KNN | 0.907 | 0.887 | 0.856 | 0.950 | 0.918 | 0.814 | 0.900 | 3 uniform |
| M0497 | 512 | exp05 | 1024bit-ECFP6 | KNN | 0.927 | 0.896 | 0.864 | 0.979 | 0.936 | 0.855 | 0.908 | 5 uniform |
| M0498 | 512 | exp06 | 1024bit-ECFP6 | KNN | 0.880 | 0.899 | 0.881 | 0.879 | 0.889 | 0.759 | 0.905 | 3 uniform |
| M0499 | 512 | exp07 | 1024bit-ECFP6 | KNN | 0.915 | 0.905 | 0.881 | 0.943 | 0.924 | 0.829 | 0.902 | 5 uniform |
| M0500 | 512 | exp08 | 1024bit-ECFP6 | KNN | 0.903 | 0.892 | 0.864 | 0.936 | 0.913 | 0.806 | 0.902 | 5 uniform |
| M0501 | 512 | exp09 | 1024bit-ECFP6 | KNN | 0.911 | 0.899 | 0.873 | 0.943 | 0.920 | 0.821 | 0.901 | 5 uniform |
| M0502 | 512 | exp10 | 1024bit-ECFP6 | KNN | 0.888 | 0.873 | 0.839 | 0.929 | 0.900 | 0.775 | 0.905 | 3 uniform |
| M0503 | 512 | exp11 | 1024bit-ECFP6 | KNN | 0.919 | 0.905 | 0.881 | 0.950 | 0.927 | 0.837 | 0.908 | 3 uniform |
| M0504 | 512 | exp12 | 1024bit-ECFP6 | KNN | 0.880 | 0.893 | 0.873 | 0.887 | 0.890 | 0.759 | 0.915 | 3 uniform |
| M0505 | 512 | exp13 | 1024bit-ECFP6 | KNN | 0.884 | 0.878 | 0.847 | 0.915 | 0.896 | 0.766 | 0.913 | 3 distance |
| M0506 | 512 | exp14 | 1024bit-ECFP6 | KNN | 0.900 | 0.881 | 0.847 | 0.943 | 0.911 | 0.799 | 0.908 | 5 distance |
| M0507 | 512 | exp15 | 1024bit-ECFP6 | KNN | 0.915 | 0.910 | 0.890 | 0.936 | 0.923 | 0.829 | 0.904 | 5 distance |
| M0508 | 512 | exp16 | 1024bit-ECFP6 | KNN | 0.919 | 0.917 | 0.898 | 0.936 | 0.926 | 0.836 | 0.898 | 3 uniform |
| M0509 | 512 | exp17 | 1024bit-ECFP6 | KNN | 0.907 | 0.915 | 0.898 | 0.915 | 0.915 | 0.813 | 0.909 | 3 uniform |
| M0510 | 512 | exp18 | 1024bit-ECFP6 | KNN | 0.888 | 0.889 | 0.864 | 0.908 | 0.898 | 0.774 | 0.902 | 5 distance |
| M0511 | 512 | exp19 | 1024bit-ECFP6 | KNN | 0.919 | 0.917 | 0.898 | 0.936 | 0.926 | 0.836 | 0.906 | 3 uniform |
| M0512 | 512 | exp01 | 1024bit-ECFP6 | SVM | 0.880 | 0.867 | 0.831 | 0.922 | 0.893 | 0.759 | 0.914 | 100000.0 0.1 |
| M0513 | 512 | exp02 | 1024bit-ECFP6 | SVM | 0.896 | 0.885 | 0.856 | 0.929 | 0.907 | 0.790 | 0.920 | 100 0.05 |
| M0514 | 512 | exp03 | 1024bit-ECFP6 | SVM | 0.907 | 0.887 | 0.856 | 0.950 | 0.918 | 0.814 | 0.936 | 10000.0 0.05 |
| M0515 | 512 | exp04 | 1024bit-ECFP6 | SVM | 0.896 | 0.880 | 0.847 | 0.936 | 0.907 | 0.790 | 0.915 | 5 0.05 |
| M0516 | 512 | exp05 | 1024bit-ECFP6 | SVM | 0.915 | 0.894 | 0.864 | 0.957 | 0.925 | 0.830 | 0.913 | 5 0.05 |
| M0517 | 512 | exp06 | 1024bit-ECFP6 | SVM | 0.934 | 0.943 | 0.932 | 0.936 | 0.940 | 0.868 | 0.913 | 5 0.05 |
| M0518 | 512 | exp07 | 1024bit-ECFP6 | SVM | 0.923 | 0.906 | 0.881 | 0.957 | 0.931 | 0.845 | 0.911 | 5 0.05 |
| M0519 | 512 | exp08 | 1024bit-ECFP6 | SVM | 0.934 | 0.931 | 0.915 | 0.950 | 0.940 | 0.868 | 0.910 | 5 0.05 |
| M0520 | 512 | exp09 | 1024bit-ECFP6 | SVM | 0.942 | 0.944 | 0.932 | 0.950 | 0.947 | 0.883 | 0.906 | 100 0.1 |
| M0521 | 512 | exp10 | 1024bit-ECFP6 | SVM | 0.915 | 0.889 | 0.856 | 0.965 | 0.925 | 0.831 | 0.907 | 100 0.05 |
| M0522 | 512 | exp11 | 1024bit-ECFP6 | SVM | 0.907 | 0.898 | 0.873 | 0.936 | 0.917 | 0.813 | 0.916 | 1 0.05 |
| M0523 | 512 | exp12 | 1024bit-ECFP6 | SVM | 0.896 | 0.907 | 0.890 | 0.901 | 0.904 | 0.790 | 0.921 | 10000.0 0.05 |
| M0524 | 512 | exp13 | 1024bit-ECFP6 | SVM | 0.888 | 0.873 | 0.839 | 0.929 | 0.900 | 0.775 | 0.920 | 50 0.1 |
| M0525 | 512 | exp14 | 1024bit-ECFP6 | SVM | 0.896 | 0.880 | 0.847 | 0.936 | 0.907 | 0.790 | 0.920 | 50000.0 0.05 |
| M0526 | 512 | exp15 | 1024bit-ECFP6 | SVM | 0.900 | 0.876 | 0.839 | 0.950 | 0.912 | 0.799 | 0.903 | 5000.0 0.05 |
| M0527 | 512 | exp16 | 1024bit-ECFP6 | SVM | 0.931 | 0.907 | 0.881 | 0.972 | 0.938 | 0.862 | 0.915 | 10 0.05 |
| M0528 | 512 | exp17 | 1024bit-ECFP6 | SVM | 0.900 | 0.908 | 0.890 | 0.908 | 0.908 | 0.798 | 0.922 | 1 0.05 |
| M0529 | 512 | exp18 | 1024bit-ECFP6 | SVM | 0.903 | 0.908 | 0.890 | 0.915 | 0.912 | 0.805 | 0.908 | 10 0.05 |
| M0530 | 512 | exp19 | 1024bit-ECFP6 | SVM | 0.938 | 0.937 | 0.924 | 0.950 | 0.944 | 0.875 | 0.919 | 5000.0 0.05 |
| M0531 | 512 | exp01 | 1024bit-ECFP6 | GBM | 0.876 | 0.871 | 0.839 | 0.908 | 0.889 | 0.751 | 0.899 | 200 0.2 |
| M0532 | 512 | exp02 | 1024bit-ECFP6 | GBM | 0.876 | 0.876 | 0.847 | 0.901 | 0.888 | 0.751 | 0.897 | 200 0.4 |
| M0533 | 512 | exp03 | 1024bit-ECFP6 | GBM | 0.865 | 0.863 | 0.831 | 0.894 | 0.878 | 0.727 | 0.920 | 500 0.3 |
| M0534 | 512 | exp04 | 1024bit-ECFP6 | GBM | 0.884 | 0.883 | 0.856 | 0.908 | 0.895 | 0.766 | 0.897 | 400 0.4 |
| M0535 | 512 | exp05 | 1024bit-ECFP6 | GBM | 0.892 | 0.865 | 0.822 | 0.950 | 0.905 | 0.785 | 0.906 | 700 0.1 |
| M0536 | 512 | exp06 | 1024bit-ECFP6 | GBM | 0.931 | 0.936 | 0.924 | 0.936 | 0.936 | 0.860 | 0.905 | 400 0.4 |
| M0537 | 512 | exp07 | 1024bit-ECFP6 | GBM | 0.903 | 0.882 | 0.847 | 0.950 | 0.915 | 0.807 | 0.890 | 500 0.4 |
| M0538 | 512 | exp08 | 1024bit-ECFP6 | GBM | 0.907 | 0.898 | 0.873 | 0.936 | 0.917 | 0.813 | 0.895 | 200 0.3 |
| M0539 | 512 | exp09 | 1024bit-ECFP6 | GBM | 0.934 | 0.931 | 0.915 | 0.950 | 0.940 | 0.868 | 0.891 | 300 0.7 |
| M0540 | 512 | exp10 | 1024bit-ECFP6 | GBM | 0.903 | 0.887 | 0.856 | 0.943 | 0.914 | 0.806 | 0.903 | 700 0.2 |
| M0541 | 512 | exp11 | 1024bit-ECFP6 | GBM | 0.915 | 0.899 | 0.873 | 0.950 | 0.924 | 0.829 | 0.898 | 200 0.1 |
| M0542 | 512 | exp12 | 1024bit-ECFP6 | GBM | 0.892 | 0.912 | 0.898 | 0.887 | 0.899 | 0.783 | 0.902 | 100 0.3 |
| M0543 | 512 | exp13 | 1024bit-ECFP6 | GBM | 0.892 | 0.890 | 0.864 | 0.915 | 0.902 | 0.782 | 0.911 | 300 0.8 |
| M0544 | 512 | exp14 | 1024bit-ECFP6 | GBM | 0.880 | 0.872 | 0.839 | 0.915 | 0.893 | 0.759 | 0.907 | 700 0.1 |
| M0545 | 512 | exp15 | 1024bit-ECFP6 | GBM | 0.876 | 0.861 | 0.822 | 0.922 | 0.890 | 0.752 | 0.896 | 600 0.1 |
| M0546 | 512 | exp16 | 1024bit-ECFP6 | GBM | 0.896 | 0.875 | 0.839 | 0.943 | 0.908 | 0.791 | 0.895 | 300 0.3 |
| M0547 | 512 | exp17 | 1024bit-ECFP6 | GBM | 0.903 | 0.903 | 0.881 | 0.922 | 0.912 | 0.805 | 0.898 | 200 0.4 |
| M0548 | 512 | exp18 | 1024bit-ECFP6 | GBM | 0.896 | 0.907 | 0.890 | 0.901 | 0.904 | 0.790 | 0.894 | 300 0.3 |
| M0549 | 512 | exp19 | 1024bit-ECFP6 | GBM | 0.927 | 0.936 | 0.924 | 0.929 | 0.932 | 0.852 | 0.909 | 300 0.5 |
| M0550 | 512 | exp01 | 1024bit-ECFP6 | RF | 0.873 | 0.855 | 0.814 | 0.922 | 0.887 | 0.744 | 0.918 | 500 |
| M0551 | 512 | exp02 | 1024bit-ECFP6 | RF | 0.888 | 0.868 | 0.831 | 0.936 | 0.901 | 0.775 | 0.915 | 200 |
| M0552 | 512 | exp03 | 1024bit-ECFP6 | RF | 0.896 | 0.865 | 0.822 | 0.957 | 0.909 | 0.793 | 0.919 | 300 |
| M0553 | 512 | exp04 | 1024bit-ECFP6 | RF | 0.915 | 0.884 | 0.847 | 0.972 | 0.926 | 0.832 | 0.914 | 900 |
| M0554 | 512 | exp05 | 1024bit-ECFP6 | RF | 0.911 | 0.869 | 0.822 | 0.986 | 0.924 | 0.828 | 0.912 | 200 |
| M0555 | 512 | exp06 | 1024bit-ECFP6 | RF | 0.915 | 0.905 | 0.881 | 0.943 | 0.924 | 0.829 | 0.918 | 200 |
| M0556 | 512 | exp07 | 1024bit-ECFP6 | RF | 0.919 | 0.885 | 0.847 | 0.979 | 0.929 | 0.841 | 0.901 | 500 |
| M0557 | 512 | exp08 | 1024bit-ECFP6 | RF | 0.923 | 0.906 | 0.881 | 0.957 | 0.931 | 0.845 | 0.912 | 700 |
| M0558 | 512 | exp09 | 1024bit-ECFP6 | RF | 0.958 | 0.939 | 0.924 | 0.986 | 0.962 | 0.915 | 0.904 | 1000 |
| M0559 | 512 | exp10 | 1024bit-ECFP6 | RF | 0.907 | 0.868 | 0.822 | 0.979 | 0.920 | 0.819 | 0.911 | 600 |
| M0560 | 512 | exp11 | 1024bit-ECFP6 | RF | 0.923 | 0.901 | 0.873 | 0.965 | 0.932 | 0.846 | 0.910 | 800 |
| M0561 | 512 | exp12 | 1024bit-ECFP6 | RF | 0.896 | 0.885 | 0.856 | 0.929 | 0.907 | 0.790 | 0.917 | 800 |
| M0562 | 512 | exp13 | 1024bit-ECFP6 | RF | 0.888 | 0.878 | 0.847 | 0.922 | 0.900 | 0.774 | 0.918 | 900 |
| M0563 | 512 | exp14 | 1024bit-ECFP6 | RF | 0.907 | 0.877 | 0.839 | 0.965 | 0.919 | 0.816 | 0.923 | 600 |
| M0564 | 512 | exp15 | 1024bit-ECFP6 | RF | 0.896 | 0.885 | 0.856 | 0.929 | 0.907 | 0.790 | 0.905 | 500 |
| M0565 | 512 | exp16 | 1024bit-ECFP6 | RF | 0.903 | 0.877 | 0.839 | 0.957 | 0.915 | 0.808 | 0.909 | 900 |
| M0566 | 512 | exp17 | 1024bit-ECFP6 | RF | 0.900 | 0.897 | 0.873 | 0.922 | 0.909 | 0.797 | 0.912 | 500 |
| M0567 | 512 | exp18 | 1024bit-ECFP6 | RF | 0.911 | 0.921 | 0.907 | 0.915 | 0.918 | 0.821 | 0.903 | 1000 |
| M0568 | 512 | exp19 | 1024bit-ECFP6 | RF | 0.931 | 0.913 | 0.890 | 0.965 | 0.938 | 0.861 | 0.918 | 300 |
| M0569 | 512 | exp01 | 1024bit-ECFP6 | DNN2 | 0.884 | 0.894 | 0.873 | 0.894 | 0.894 | 0.766 | 0.905 | 400 60 0.4 |
| M0570 | 512 | exp02 | 1024bit-ECFP6 | DNN2 | 0.857 | 0.871 | 0.847 | 0.865 | 0.868 | 0.712 | 0.914 | 100 80 0.1 |
| M0571 | 512 | exp03 | 1024bit-ECFP6 | DNN2 | 0.865 | 0.858 | 0.822 | 0.901 | 0.879 | 0.727 | 0.930 | 200 80 0.3 |
| M0572 | 512 | exp01 | 1024bit-ECFP6 | DNN3 | 0.876 | 0.898 | 0.881 | 0.872 | 0.885 | 0.752 | 0.908 | 300 100 0.2 |
| M0573 | 512 | exp02 | 1024bit-ECFP6 | DNN3 | 0.876 | 0.887 | 0.864 | 0.887 | 0.887 | 0.751 | 0.915 | 300 100 0.5 |
| M0574 | 512 | exp03 | 1024bit-ECFP6 | DNN3 | 0.869 | 0.879 | 0.856 | 0.879 | 0.879 | 0.735 | 0.923 | 400 100 0.3 |
| M0575 | 512 | exp01 | 2048bit-ECFP6 | KNN | 0.869 | 0.854 | 0.814 | 0.915 | 0.884 | 0.736 | 0.925 | 3 distance |
| M0576 | 512 | exp02 | 2048bit-ECFP6 | KNN | 0.861 | 0.867 | 0.839 | 0.879 | 0.873 | 0.719 | 0.913 | 1 uniform |
| M0577 | 512 | exp03 | 2048bit-ECFP6 | KNN | 0.903 | 0.872 | 0.831 | 0.965 | 0.916 | 0.809 | 0.919 | 3 distance |
| M0578 | 512 | exp04 | 2048bit-ECFP6 | KNN | 0.911 | 0.883 | 0.847 | 0.965 | 0.922 | 0.824 | 0.909 | 1 uniform |
| M0579 | 512 | exp05 | 2048bit-ECFP6 | KNN | 0.927 | 0.901 | 0.873 | 0.972 | 0.935 | 0.854 | 0.915 | 3 distance |
| M0580 | 512 | exp06 | 2048bit-ECFP6 | KNN | 0.907 | 0.915 | 0.898 | 0.915 | 0.915 | 0.813 | 0.914 | 3 uniform |
| M0581 | 512 | exp07 | 2048bit-ECFP6 | KNN | 0.900 | 0.876 | 0.839 | 0.950 | 0.912 | 0.799 | 0.906 | 7 distance |
| M0582 | 512 | exp08 | 2048bit-ECFP6 | KNN | 0.931 | 0.918 | 0.898 | 0.957 | 0.938 | 0.860 | 0.910 | 3 distance |
| M0583 | 512 | exp09 | 2048bit-ECFP6 | KNN | 0.934 | 0.925 | 0.907 | 0.957 | 0.941 | 0.868 | 0.907 | 3 distance |
| M0584 | 512 | exp10 | 2048bit-ECFP6 | KNN | 0.915 | 0.889 | 0.856 | 0.965 | 0.925 | 0.831 | 0.911 | 3 distance |
| M0585 | 512 | exp11 | 2048bit-ECFP6 | KNN | 0.911 | 0.899 | 0.873 | 0.943 | 0.920 | 0.821 | 0.916 | 3 distance |
| M0586 | 512 | exp12 | 2048bit-ECFP6 | KNN | 0.903 | 0.892 | 0.864 | 0.936 | 0.913 | 0.806 | 0.920 | 3 uniform |
| M0587 | 512 | exp13 | 2048bit-ECFP6 | KNN | 0.873 | 0.860 | 0.822 | 0.915 | 0.887 | 0.743 | 0.922 | 3 uniform |
| M0588 | 512 | exp14 | 2048bit-ECFP6 | KNN | 0.907 | 0.893 | 0.864 | 0.943 | 0.917 | 0.814 | 0.911 | 5 uniform |
| M0589 | 512 | exp15 | 2048bit-ECFP6 | KNN | 0.911 | 0.904 | 0.881 | 0.936 | 0.920 | 0.821 | 0.909 | 3 distance |
| M0590 | 512 | exp16 | 2048bit-ECFP6 | KNN | 0.888 | 0.868 | 0.831 | 0.936 | 0.901 | 0.775 | 0.920 | 1 uniform |
| M0591 | 512 | exp17 | 2048bit-ECFP6 | KNN | 0.903 | 0.903 | 0.881 | 0.922 | 0.912 | 0.805 | 0.921 | 3 uniform |
| M0592 | 512 | exp18 | 2048bit-ECFP6 | KNN | 0.907 | 0.909 | 0.890 | 0.922 | 0.915 | 0.813 | 0.911 | 1 uniform |
| M0593 | 512 | exp19 | 2048bit-ECFP6 | KNN | 0.931 | 0.918 | 0.898 | 0.957 | 0.938 | 0.860 | 0.911 | 3 uniform |
| M0594 | 512 | exp01 | 2048bit-ECFP6 | SVM | 0.907 | 0.915 | 0.898 | 0.915 | 0.915 | 0.813 | 0.922 | 50000.0 0.05 |
| M0595 | 512 | exp02 | 2048bit-ECFP6 | SVM | 0.884 | 0.868 | 0.831 | 0.929 | 0.897 | 0.767 | 0.921 | 5 0.05 |
| M0596 | 512 | exp03 | 2048bit-ECFP6 | SVM | 0.907 | 0.893 | 0.864 | 0.943 | 0.917 | 0.814 | 0.929 | 1 0.05 |
| M0597 | 512 | exp04 | 2048bit-ECFP6 | SVM | 0.923 | 0.906 | 0.881 | 0.957 | 0.931 | 0.845 | 0.915 | 100 0.1 |
| M0598 | 512 | exp05 | 2048bit-ECFP6 | SVM | 0.896 | 0.880 | 0.847 | 0.936 | 0.907 | 0.790 | 0.919 | 10 0.05 |
| M0599 | 512 | exp06 | 2048bit-ECFP6 | SVM | 0.919 | 0.941 | 0.932 | 0.908 | 0.924 | 0.838 | 0.918 | 100000.0 0.05 |
| M0600 | 512 | exp07 | 2048bit-ECFP6 | SVM | 0.919 | 0.900 | 0.873 | 0.957 | 0.928 | 0.838 | 0.914 | 5 0.05 |
| M0601 | 512 | exp08 | 2048bit-ECFP6 | SVM | 0.938 | 0.937 | 0.924 | 0.950 | 0.944 | 0.875 | 0.919 | 1000.0 0.05 |
| M0602 | 512 | exp09 | 2048bit-ECFP6 | SVM | 0.931 | 0.930 | 0.915 | 0.943 | 0.937 | 0.860 | 0.909 | 1 0.1 |
| M0603 | 512 | exp10 | 2048bit-ECFP6 | SVM | 0.919 | 0.885 | 0.847 | 0.979 | 0.929 | 0.841 | 0.919 | 100000.0 0.1 |
| M0604 | 512 | exp11 | 2048bit-ECFP6 | SVM | 0.915 | 0.905 | 0.881 | 0.943 | 0.924 | 0.829 | 0.917 | 5000.0 0.05 |
| M0605 | 512 | exp12 | 2048bit-ECFP6 | SVM | 0.884 | 0.911 | 0.898 | 0.872 | 0.891 | 0.768 | 0.925 | 5 0.05 |
| M0606 | 512 | exp13 | 2048bit-ECFP6 | SVM | 0.903 | 0.887 | 0.856 | 0.943 | 0.914 | 0.806 | 0.925 | 1 0.1 |
| M0607 | 512 | exp14 | 2048bit-ECFP6 | SVM | 0.900 | 0.881 | 0.847 | 0.943 | 0.911 | 0.799 | 0.929 | 100 0.05 |
| M0608 | 512 | exp15 | 2048bit-ECFP6 | SVM | 0.911 | 0.899 | 0.873 | 0.943 | 0.920 | 0.821 | 0.911 | 1 0.1 |
| M0609 | 512 | exp16 | 2048bit-ECFP6 | SVM | 0.931 | 0.918 | 0.898 | 0.957 | 0.938 | 0.860 | 0.922 | 5 0.05 |
| M0610 | 512 | exp17 | 2048bit-ECFP6 | SVM | 0.911 | 0.928 | 0.915 | 0.908 | 0.918 | 0.822 | 0.927 | 5 0.05 |
| M0611 | 512 | exp18 | 2048bit-ECFP6 | SVM | 0.915 | 0.916 | 0.898 | 0.929 | 0.923 | 0.829 | 0.917 | 10 0.05 |
| M0612 | 512 | exp19 | 2048bit-ECFP6 | SVM | 0.927 | 0.912 | 0.890 | 0.957 | 0.934 | 0.853 | 0.920 | 100000.0 0.1 |
| M0613 | 512 | exp01 | 2048bit-ECFP6 | GBM | 0.884 | 0.868 | 0.831 | 0.929 | 0.897 | 0.767 | 0.905 | 200 0.6 |
| M0614 | 512 | exp02 | 2048bit-ECFP6 | GBM | 0.869 | 0.879 | 0.856 | 0.879 | 0.879 | 0.735 | 0.912 | 200 0.7 |
| M0615 | 512 | exp03 | 2048bit-ECFP6 | GBM | 0.892 | 0.874 | 0.839 | 0.936 | 0.904 | 0.783 | 0.903 | 400 0.6 |
| M0616 | 512 | exp04 | 2048bit-ECFP6 | GBM | 0.892 | 0.869 | 0.831 | 0.943 | 0.905 | 0.784 | 0.902 | 400 0.5 |
| M0617 | 512 | exp05 | 2048bit-ECFP6 | GBM | 0.896 | 0.875 | 0.839 | 0.943 | 0.908 | 0.791 | 0.906 | 300 0.4 |
| M0618 | 512 | exp06 | 2048bit-ECFP6 | GBM | 0.888 | 0.894 | 0.873 | 0.901 | 0.898 | 0.774 | 0.905 | 300 0.2 |
| M0619 | 512 | exp07 | 2048bit-ECFP6 | GBM | 0.903 | 0.892 | 0.864 | 0.936 | 0.913 | 0.806 | 0.905 | 300 0.5 |
| M0620 | 512 | exp08 | 2048bit-ECFP6 | GBM | 0.892 | 0.865 | 0.822 | 0.950 | 0.905 | 0.785 | 0.896 | 700 0.2 |
| M0621 | 512 | exp09 | 2048bit-ECFP6 | GBM | 0.931 | 0.924 | 0.907 | 0.950 | 0.937 | 0.860 | 0.889 | 200 0.2 |
| M0622 | 512 | exp10 | 2048bit-ECFP6 | GBM | 0.888 | 0.873 | 0.839 | 0.929 | 0.900 | 0.775 | 0.904 | 300 0.3 |
| M0623 | 512 | exp11 | 2048bit-ECFP6 | GBM | 0.900 | 0.897 | 0.873 | 0.922 | 0.909 | 0.797 | 0.902 | 200 0.6 |
| M0624 | 512 | exp12 | 2048bit-ECFP6 | GBM | 0.861 | 0.862 | 0.831 | 0.887 | 0.874 | 0.719 | 0.901 | 500 0.4 |
| M0625 | 512 | exp13 | 2048bit-ECFP6 | GBM | 0.884 | 0.872 | 0.839 | 0.922 | 0.897 | 0.767 | 0.910 | 1000 0.2 |
| M0626 | 512 | exp14 | 2048bit-ECFP6 | GBM | 0.896 | 0.870 | 0.831 | 0.950 | 0.908 | 0.792 | 0.903 | 200 0.1 |
| M0627 | 512 | exp15 | 2048bit-ECFP6 | GBM | 0.873 | 0.851 | 0.805 | 0.929 | 0.888 | 0.745 | 0.896 | 50 0.4 |
| M0628 | 512 | exp16 | 2048bit-ECFP6 | GBM | 0.900 | 0.871 | 0.831 | 0.957 | 0.912 | 0.801 | 0.910 | 200 0.1 |
| M0629 | 512 | exp17 | 2048bit-ECFP6 | GBM | 0.896 | 0.907 | 0.890 | 0.901 | 0.904 | 0.790 | 0.902 | 200 0.2 |
| M0630 | 512 | exp18 | 2048bit-ECFP6 | GBM | 0.888 | 0.912 | 0.898 | 0.879 | 0.895 | 0.776 | 0.905 | 200 0.4 |
| M0631 | 512 | exp19 | 2048bit-ECFP6 | GBM | 0.919 | 0.905 | 0.881 | 0.950 | 0.927 | 0.837 | 0.893 | 600 0.4 |
| M0632 | 512 | exp01 | 2048bit-ECFP6 | RF | 0.896 | 0.875 | 0.839 | 0.943 | 0.908 | 0.791 | 0.923 | 50 |
| M0633 | 512 | exp02 | 2048bit-ECFP6 | RF | 0.884 | 0.872 | 0.839 | 0.922 | 0.897 | 0.767 | 0.918 | 700 |
| M0634 | 512 | exp03 | 2048bit-ECFP6 | RF | 0.900 | 0.866 | 0.822 | 0.965 | 0.913 | 0.802 | 0.924 | 500 |
| M0635 | 512 | exp04 | 2048bit-ECFP6 | RF | 0.923 | 0.895 | 0.864 | 0.972 | 0.932 | 0.847 | 0.913 | 400 |
| M0636 | 512 | exp05 | 2048bit-ECFP6 | RF | 0.903 | 0.867 | 0.822 | 0.972 | 0.916 | 0.810 | 0.920 | 1000 |
| M0637 | 512 | exp06 | 2048bit-ECFP6 | RF | 0.903 | 0.897 | 0.873 | 0.929 | 0.913 | 0.805 | 0.920 | 400 |
| M0638 | 512 | exp07 | 2048bit-ECFP6 | RF | 0.919 | 0.885 | 0.847 | 0.979 | 0.929 | 0.841 | 0.913 | 800 |
| M0639 | 512 | exp08 | 2048bit-ECFP6 | RF | 0.927 | 0.912 | 0.890 | 0.957 | 0.934 | 0.853 | 0.908 | 800 |
| M0640 | 512 | exp09 | 2048bit-ECFP6 | RF | 0.946 | 0.932 | 0.915 | 0.972 | 0.951 | 0.892 | 0.904 | 200 |
| M0641 | 512 | exp10 | 2048bit-ECFP6 | RF | 0.919 | 0.880 | 0.839 | 0.986 | 0.930 | 0.842 | 0.916 | 400 |
| M0642 | 512 | exp11 | 2048bit-ECFP6 | RF | 0.915 | 0.894 | 0.864 | 0.957 | 0.925 | 0.830 | 0.915 | 600 |
| M0643 | 512 | exp12 | 2048bit-ECFP6 | RF | 0.903 | 0.897 | 0.873 | 0.929 | 0.913 | 0.805 | 0.922 | 1000 |
| M0644 | 512 | exp13 | 2048bit-ECFP6 | RF | 0.888 | 0.868 | 0.831 | 0.936 | 0.901 | 0.775 | 0.918 | 500 |
| M0645 | 512 | exp14 | 2048bit-ECFP6 | RF | 0.900 | 0.866 | 0.822 | 0.965 | 0.913 | 0.802 | 0.928 | 300 |
| M0646 | 512 | exp15 | 2048bit-ECFP6 | RF | 0.903 | 0.882 | 0.847 | 0.950 | 0.915 | 0.807 | 0.906 | 100 |
| M0647 | 512 | exp16 | 2048bit-ECFP6 | RF | 0.911 | 0.888 | 0.856 | 0.957 | 0.922 | 0.823 | 0.913 | 700 |
| M0648 | 512 | exp17 | 2048bit-ECFP6 | RF | 0.892 | 0.890 | 0.864 | 0.915 | 0.902 | 0.782 | 0.915 | 400 |
| M0649 | 512 | exp18 | 2048bit-ECFP6 | RF | 0.911 | 0.915 | 0.898 | 0.922 | 0.919 | 0.821 | 0.909 | 200 |
| M0650 | 512 | exp19 | 2048bit-ECFP6 | RF | 0.931 | 0.902 | 0.873 | 0.979 | 0.939 | 0.863 | 0.919 | 400 |
| M0651 | 512 | exp01 | 2048bit-ECFP6 | DNN2 | 0.873 | 0.865 | 0.831 | 0.908 | 0.886 | 0.743 | 0.918 | 200 120 0.1 |
| M0652 | 512 | exp02 | 2048bit-ECFP6 | DNN2 | 0.880 | 0.877 | 0.847 | 0.908 | 0.892 | 0.758 | 0.922 | 300 120 0.2 |
| M0653 | 512 | exp03 | 2048bit-ECFP6 | DNN2 | 0.873 | 0.860 | 0.822 | 0.915 | 0.887 | 0.743 | 0.922 | 100 100 0.5 |
| M0654 | 512 | exp01 | 2048bit-ECFP6 | DNN3 | 0.884 | 0.883 | 0.856 | 0.908 | 0.895 | 0.766 | 0.915 | 400 140 0.2 |
| M0655 | 512 | exp02 | 2048bit-ECFP6 | DNN3 | 0.888 | 0.884 | 0.856 | 0.915 | 0.899 | 0.774 | 0.921 | 200 160 0.4 |
| M0656 | 512 | exp03 | 2048bit-ECFP6 | DNN3 | 0.888 | 0.868 | 0.831 | 0.936 | 0.901 | 0.775 | 0.922 | 200 160 0.1 |
| M0657 | 256 | exp01 | 1024bit-ECFP4 | KNN | 0.884 | 0.872 | 0.839 | 0.922 | 0.897 | 0.767 | 0.924 | 5 distance |
| M0658 | 256 | exp02 | 1024bit-ECFP4 | KNN | 0.869 | 0.879 | 0.856 | 0.879 | 0.879 | 0.735 | 0.913 | 5 uniform |
| M0659 | 256 | exp03 | 1024bit-ECFP4 | KNN | 0.923 | 0.901 | 0.873 | 0.965 | 0.932 | 0.846 | 0.920 | 3 distance |
| M0660 | 256 | exp04 | 1024bit-ECFP4 | KNN | 0.896 | 0.870 | 0.831 | 0.950 | 0.908 | 0.792 | 0.912 | 1 uniform |
| M0661 | 256 | exp05 | 1024bit-ECFP4 | KNN | 0.907 | 0.882 | 0.847 | 0.957 | 0.918 | 0.815 | 0.917 | 3 uniform |
| M0662 | 256 | exp06 | 1024bit-ECFP4 | KNN | 0.911 | 0.928 | 0.915 | 0.908 | 0.918 | 0.822 | 0.915 | 3 uniform |
| M0663 | 256 | exp07 | 1024bit-ECFP4 | KNN | 0.907 | 0.887 | 0.856 | 0.950 | 0.918 | 0.814 | 0.906 | 1 uniform |
| M0664 | 256 | exp08 | 1024bit-ECFP4 | KNN | 0.934 | 0.937 | 0.924 | 0.943 | 0.940 | 0.868 | 0.913 | 3 uniform |
| M0665 | 256 | exp09 | 1024bit-ECFP4 | KNN | 0.934 | 0.931 | 0.915 | 0.950 | 0.940 | 0.868 | 0.913 | 3 uniform |
| M0666 | 256 | exp10 | 1024bit-ECFP4 | KNN | 0.923 | 0.906 | 0.881 | 0.957 | 0.931 | 0.845 | 0.910 | 5 distance |
| M0667 | 256 | exp11 | 1024bit-ECFP4 | KNN | 0.919 | 0.917 | 0.898 | 0.936 | 0.926 | 0.836 | 0.910 | 3 uniform |
| M0668 | 256 | exp12 | 1024bit-ECFP4 | KNN | 0.907 | 0.903 | 0.881 | 0.929 | 0.916 | 0.813 | 0.921 | 5 distance |
| M0669 | 256 | exp13 | 1024bit-ECFP4 | KNN | 0.884 | 0.883 | 0.856 | 0.908 | 0.895 | 0.766 | 0.922 | 3 distance |
| M0670 | 256 | exp14 | 1024bit-ECFP4 | KNN | 0.919 | 0.900 | 0.873 | 0.957 | 0.928 | 0.838 | 0.911 | 3 uniform |
| M0671 | 256 | exp15 | 1024bit-ECFP4 | KNN | 0.903 | 0.897 | 0.873 | 0.929 | 0.913 | 0.805 | 0.905 | 5 distance |
| M0672 | 256 | exp16 | 1024bit-ECFP4 | KNN | 0.931 | 0.930 | 0.915 | 0.943 | 0.937 | 0.860 | 0.914 | 3 uniform |
| M0673 | 256 | exp17 | 1024bit-ECFP4 | KNN | 0.911 | 0.928 | 0.915 | 0.908 | 0.918 | 0.822 | 0.913 | 3 distance |
| M0674 | 256 | exp18 | 1024bit-ECFP4 | KNN | 0.919 | 0.941 | 0.932 | 0.908 | 0.924 | 0.838 | 0.906 | 3 uniform |
| M0675 | 256 | exp19 | 1024bit-ECFP4 | KNN | 0.931 | 0.936 | 0.924 | 0.936 | 0.936 | 0.860 | 0.912 | 5 distance |
| M0676 | 256 | exp01 | 1024bit-ECFP4 | SVM | 0.915 | 0.916 | 0.898 | 0.929 | 0.923 | 0.829 | 0.925 | 10000.0 0.1 |
| M0677 | 256 | exp02 | 1024bit-ECFP4 | SVM | 0.884 | 0.883 | 0.856 | 0.908 | 0.895 | 0.766 | 0.920 | 1 0.1 |
| M0678 | 256 | exp03 | 1024bit-ECFP4 | SVM | 0.907 | 0.898 | 0.873 | 0.936 | 0.917 | 0.813 | 0.925 | 5 0.1 |
| M0679 | 256 | exp04 | 1024bit-ECFP4 | SVM | 0.903 | 0.887 | 0.856 | 0.943 | 0.914 | 0.806 | 0.924 | 500 0.1 |
| M0680 | 256 | exp05 | 1024bit-ECFP4 | SVM | 0.888 | 0.864 | 0.822 | 0.943 | 0.902 | 0.776 | 0.923 | 1000.0 0.1 |
| M0681 | 256 | exp06 | 1024bit-ECFP4 | SVM | 0.934 | 0.956 | 0.949 | 0.922 | 0.939 | 0.869 | 0.922 | 1000.0 0.1 |
| M0682 | 256 | exp07 | 1024bit-ECFP4 | SVM | 0.923 | 0.906 | 0.881 | 0.957 | 0.931 | 0.845 | 0.912 | 50000.0 0.1 |
| M0683 | 256 | exp08 | 1024bit-ECFP4 | SVM | 0.927 | 0.924 | 0.907 | 0.943 | 0.933 | 0.852 | 0.914 | 1 0.1 |
| M0684 | 256 | exp09 | 1024bit-ECFP4 | SVM | 0.946 | 0.944 | 0.932 | 0.957 | 0.951 | 0.891 | 0.909 | 500 0.1 |
| M0685 | 256 | exp10 | 1024bit-ECFP4 | SVM | 0.911 | 0.878 | 0.839 | 0.972 | 0.923 | 0.825 | 0.917 | 1 0.1 |
| M0686 | 256 | exp11 | 1024bit-ECFP4 | SVM | 0.915 | 0.941 | 0.932 | 0.901 | 0.920 | 0.830 | 0.920 | 5 0.05 |
| M0687 | 256 | exp12 | 1024bit-ECFP4 | SVM | 0.907 | 0.915 | 0.898 | 0.915 | 0.915 | 0.813 | 0.926 | 1 0.1 |
| M0688 | 256 | exp13 | 1024bit-ECFP4 | SVM | 0.892 | 0.895 | 0.873 | 0.908 | 0.901 | 0.782 | 0.927 | 1 0.1 |
| M0689 | 256 | exp14 | 1024bit-ECFP4 | SVM | 0.903 | 0.882 | 0.847 | 0.950 | 0.915 | 0.807 | 0.926 | 5 0.1 |
| M0690 | 256 | exp15 | 1024bit-ECFP4 | SVM | 0.907 | 0.893 | 0.864 | 0.943 | 0.917 | 0.814 | 0.911 | 5 0.1 |
| M0691 | 256 | exp16 | 1024bit-ECFP4 | SVM | 0.915 | 0.894 | 0.864 | 0.957 | 0.925 | 0.830 | 0.917 | 100 0.1 |
| M0692 | 256 | exp17 | 1024bit-ECFP4 | SVM | 0.903 | 0.920 | 0.907 | 0.901 | 0.910 | 0.806 | 0.920 | 1000.0 0.1 |
| M0693 | 256 | exp18 | 1024bit-ECFP4 | SVM | 0.915 | 0.922 | 0.907 | 0.922 | 0.922 | 0.829 | 0.915 | 10 0.05 |
| M0694 | 256 | exp19 | 1024bit-ECFP4 | SVM | 0.942 | 0.944 | 0.932 | 0.950 | 0.947 | 0.883 | 0.923 | 5 0.05 |
| M0695 | 256 | exp01 | 1024bit-ECFP4 | GBM | 0.896 | 0.901 | 0.881 | 0.908 | 0.905 | 0.790 | 0.912 | 200 0.4 |
| M0696 | 256 | exp02 | 1024bit-ECFP4 | GBM | 0.888 | 0.873 | 0.839 | 0.929 | 0.900 | 0.775 | 0.906 | 200 0.6 |
| M0697 | 256 | exp03 | 1024bit-ECFP4 | GBM | 0.884 | 0.872 | 0.839 | 0.922 | 0.897 | 0.767 | 0.918 | 400 0.1 |
| M0698 | 256 | exp04 | 1024bit-ECFP4 | GBM | 0.915 | 0.910 | 0.890 | 0.936 | 0.923 | 0.829 | 0.906 | 500 0.4 |
| M0699 | 256 | exp05 | 1024bit-ECFP4 | GBM | 0.911 | 0.888 | 0.856 | 0.957 | 0.922 | 0.823 | 0.919 | 400 0.6 |
| M0700 | 256 | exp06 | 1024bit-ECFP4 | GBM | 0.931 | 0.924 | 0.907 | 0.950 | 0.937 | 0.860 | 0.901 | 100 0.3 |
| M0701 | 256 | exp07 | 1024bit-ECFP4 | GBM | 0.896 | 0.875 | 0.839 | 0.943 | 0.908 | 0.791 | 0.904 | 200 0.3 |
| M0702 | 256 | exp08 | 1024bit-ECFP4 | GBM | 0.892 | 0.890 | 0.864 | 0.915 | 0.902 | 0.782 | 0.904 | 300 0.6 |
| M0703 | 256 | exp09 | 1024bit-ECFP4 | GBM | 0.915 | 0.941 | 0.932 | 0.901 | 0.920 | 0.830 | 0.901 | 700 0.2 |
| M0704 | 256 | exp10 | 1024bit-ECFP4 | GBM | 0.888 | 0.884 | 0.856 | 0.915 | 0.899 | 0.774 | 0.904 | 50 0.6 |
| M0705 | 256 | exp11 | 1024bit-ECFP4 | GBM | 0.903 | 0.903 | 0.881 | 0.922 | 0.912 | 0.805 | 0.911 | 300 0.4 |
| M0706 | 256 | exp12 | 1024bit-ECFP4 | GBM | 0.869 | 0.879 | 0.856 | 0.879 | 0.879 | 0.735 | 0.908 | 200 0.7 |
| M0707 | 256 | exp13 | 1024bit-ECFP4 | GBM | 0.888 | 0.884 | 0.856 | 0.915 | 0.899 | 0.774 | 0.917 | 300 0.2 |
| M0708 | 256 | exp14 | 1024bit-ECFP4 | GBM | 0.880 | 0.867 | 0.831 | 0.922 | 0.893 | 0.759 | 0.918 | 100 0.4 |
| M0709 | 256 | exp15 | 1024bit-ECFP4 | GBM | 0.884 | 0.878 | 0.847 | 0.915 | 0.896 | 0.766 | 0.906 | 300 0.2 |
| M0710 | 256 | exp16 | 1024bit-ECFP4 | GBM | 0.900 | 0.891 | 0.864 | 0.929 | 0.910 | 0.798 | 0.907 | 500 0.1 |
| M0711 | 256 | exp17 | 1024bit-ECFP4 | GBM | 0.903 | 0.920 | 0.907 | 0.901 | 0.910 | 0.806 | 0.910 | 300 0.3 |
| M0712 | 256 | exp18 | 1024bit-ECFP4 | GBM | 0.892 | 0.919 | 0.907 | 0.879 | 0.899 | 0.784 | 0.905 | 400 0.5 |
| M0713 | 256 | exp19 | 1024bit-ECFP4 | GBM | 0.927 | 0.942 | 0.932 | 0.922 | 0.932 | 0.853 | 0.909 | 400 0.6 |
| M0714 | 256 | exp01 | 1024bit-ECFP4 | RF | 0.888 | 0.884 | 0.856 | 0.915 | 0.899 | 0.774 | 0.919 | 50 |
| M0715 | 256 | exp02 | 1024bit-ECFP4 | RF | 0.865 | 0.849 | 0.805 | 0.915 | 0.881 | 0.728 | 0.917 | 900 |
| M0716 | 256 | exp03 | 1024bit-ECFP4 | RF | 0.892 | 0.855 | 0.805 | 0.965 | 0.907 | 0.787 | 0.920 | 900 |
| M0717 | 256 | exp04 | 1024bit-ECFP4 | RF | 0.907 | 0.893 | 0.864 | 0.943 | 0.917 | 0.814 | 0.918 | 300 |
| M0718 | 256 | exp05 | 1024bit-ECFP4 | RF | 0.907 | 0.868 | 0.822 | 0.979 | 0.920 | 0.819 | 0.923 | 200 |
| M0719 | 256 | exp06 | 1024bit-ECFP4 | RF | 0.942 | 0.944 | 0.932 | 0.950 | 0.947 | 0.883 | 0.921 | 600 |
| M0720 | 256 | exp07 | 1024bit-ECFP4 | RF | 0.923 | 0.895 | 0.864 | 0.972 | 0.932 | 0.847 | 0.910 | 600 |
| M0721 | 256 | exp08 | 1024bit-ECFP4 | RF | 0.923 | 0.912 | 0.890 | 0.950 | 0.931 | 0.845 | 0.911 | 1000 |
| M0722 | 256 | exp09 | 1024bit-ECFP4 | RF | 0.954 | 0.939 | 0.924 | 0.979 | 0.958 | 0.907 | 0.910 | 500 |
| M0723 | 256 | exp10 | 1024bit-ECFP4 | RF | 0.900 | 0.876 | 0.839 | 0.950 | 0.912 | 0.799 | 0.916 | 300 |
| M0724 | 256 | exp11 | 1024bit-ECFP4 | RF | 0.927 | 0.924 | 0.907 | 0.943 | 0.933 | 0.852 | 0.909 | 1000 |
| M0725 | 256 | exp12 | 1024bit-ECFP4 | RF | 0.907 | 0.903 | 0.881 | 0.929 | 0.916 | 0.813 | 0.923 | 500 |
| M0726 | 256 | exp13 | 1024bit-ECFP4 | RF | 0.903 | 0.897 | 0.873 | 0.929 | 0.913 | 0.805 | 0.923 | 700 |
| M0727 | 256 | exp14 | 1024bit-ECFP4 | RF | 0.900 | 0.871 | 0.831 | 0.957 | 0.912 | 0.801 | 0.925 | 100 |
| M0728 | 256 | exp15 | 1024bit-ECFP4 | RF | 0.896 | 0.880 | 0.847 | 0.936 | 0.907 | 0.790 | 0.907 | 300 |
| M0729 | 256 | exp16 | 1024bit-ECFP4 | RF | 0.927 | 0.907 | 0.881 | 0.965 | 0.935 | 0.853 | 0.921 | 700 |
| M0730 | 256 | exp17 | 1024bit-ECFP4 | RF | 0.907 | 0.909 | 0.890 | 0.922 | 0.915 | 0.813 | 0.921 | 200 |
| M0731 | 256 | exp18 | 1024bit-ECFP4 | RF | 0.907 | 0.927 | 0.915 | 0.901 | 0.914 | 0.814 | 0.913 | 400 |
| M0732 | 256 | exp19 | 1024bit-ECFP4 | RF | 0.942 | 0.926 | 0.907 | 0.972 | 0.948 | 0.884 | 0.918 | 900 |
| M0733 | 256 | exp01 | 1024bit-ECFP4 | DNN2 | 0.873 | 0.891 | 0.873 | 0.872 | 0.882 | 0.744 | 0.921 | 200 120 0.3 |
| M0734 | 256 | exp02 | 1024bit-ECFP4 | DNN2 | 0.876 | 0.876 | 0.847 | 0.901 | 0.888 | 0.751 | 0.913 | 300 80 0.5 |
| M0735 | 256 | exp03 | 1024bit-ECFP4 | DNN2 | 0.880 | 0.882 | 0.856 | 0.901 | 0.891 | 0.758 | 0.918 | 200 160 0.1 |
| M0736 | 256 | exp01 | 1024bit-ECFP4 | DNN3 | 0.869 | 0.891 | 0.873 | 0.865 | 0.878 | 0.736 | 0.917 | 600 80 0.1 |
| M0737 | 256 | exp02 | 1024bit-ECFP4 | DNN3 | 0.876 | 0.876 | 0.847 | 0.901 | 0.888 | 0.751 | 0.915 | 500 80 0.3 |
| M0738 | 256 | exp03 | 1024bit-ECFP4 | DNN3 | 0.884 | 0.894 | 0.873 | 0.894 | 0.894 | 0.766 | 0.921 | 100 160 0.1 |
| M0739 | 256 | exp01 | 2048bit-ECFP4 | KNN | 0.896 | 0.885 | 0.856 | 0.929 | 0.907 | 0.790 | 0.925 | 5 distance |
| M0740 | 256 | exp02 | 2048bit-ECFP4 | KNN | 0.880 | 0.882 | 0.856 | 0.901 | 0.891 | 0.758 | 0.919 | 3 uniform |
| M0741 | 256 | exp03 | 2048bit-ECFP4 | KNN | 0.892 | 0.869 | 0.831 | 0.943 | 0.905 | 0.784 | 0.921 | 5 distance |
| M0742 | 256 | exp04 | 2048bit-ECFP4 | KNN | 0.907 | 0.887 | 0.856 | 0.950 | 0.918 | 0.814 | 0.912 | 3 uniform |
| M0743 | 256 | exp05 | 2048bit-ECFP4 | KNN | 0.931 | 0.913 | 0.890 | 0.965 | 0.938 | 0.861 | 0.921 | 3 distance |
| M0744 | 256 | exp06 | 2048bit-ECFP4 | KNN | 0.915 | 0.941 | 0.932 | 0.901 | 0.920 | 0.830 | 0.916 | 3 uniform |
| M0745 | 256 | exp07 | 2048bit-ECFP4 | KNN | 0.919 | 0.890 | 0.856 | 0.972 | 0.929 | 0.839 | 0.908 | 7 distance |
| M0746 | 256 | exp08 | 2048bit-ECFP4 | KNN | 0.931 | 0.930 | 0.915 | 0.943 | 0.937 | 0.860 | 0.912 | 3 distance |
| M0747 | 256 | exp09 | 2048bit-ECFP4 | KNN | 0.938 | 0.919 | 0.898 | 0.972 | 0.945 | 0.876 | 0.907 | 5 uniform |
| M0748 | 256 | exp10 | 2048bit-ECFP4 | KNN | 0.915 | 0.899 | 0.873 | 0.950 | 0.924 | 0.829 | 0.913 | 3 uniform |
| M0749 | 256 | exp11 | 2048bit-ECFP4 | KNN | 0.911 | 0.921 | 0.907 | 0.915 | 0.918 | 0.821 | 0.910 | 1 uniform |
| M0750 | 256 | exp12 | 2048bit-ECFP4 | KNN | 0.911 | 0.915 | 0.898 | 0.922 | 0.919 | 0.821 | 0.922 | 3 uniform |
| M0751 | 256 | exp13 | 2048bit-ECFP4 | KNN | 0.892 | 0.884 | 0.856 | 0.922 | 0.903 | 0.782 | 0.922 | 3 uniform |
| M0752 | 256 | exp14 | 2048bit-ECFP4 | KNN | 0.903 | 0.877 | 0.839 | 0.957 | 0.915 | 0.808 | 0.914 | 7 distance |
| M0753 | 256 | exp15 | 2048bit-ECFP4 | KNN | 0.907 | 0.882 | 0.847 | 0.957 | 0.918 | 0.815 | 0.912 | 1 uniform |
| M0754 | 256 | exp16 | 2048bit-ECFP4 | KNN | 0.927 | 0.924 | 0.907 | 0.943 | 0.933 | 0.852 | 0.917 | 3 distance |
| M0755 | 256 | exp17 | 2048bit-ECFP4 | KNN | 0.900 | 0.914 | 0.898 | 0.901 | 0.907 | 0.798 | 0.922 | 3 uniform |
| M0756 | 256 | exp18 | 2048bit-ECFP4 | KNN | 0.900 | 0.914 | 0.898 | 0.901 | 0.907 | 0.798 | 0.910 | 1 uniform |
| M0757 | 256 | exp19 | 2048bit-ECFP4 | KNN | 0.950 | 0.944 | 0.932 | 0.965 | 0.954 | 0.899 | 0.918 | 3 uniform |
| M0758 | 256 | exp01 | 2048bit-ECFP4 | SVM | 0.919 | 0.923 | 0.907 | 0.929 | 0.926 | 0.836 | 0.924 | 100000.0 0.05 |
| M0759 | 256 | exp02 | 2048bit-ECFP4 | SVM | 0.876 | 0.866 | 0.831 | 0.915 | 0.890 | 0.751 | 0.925 | 10 0.05 |
| M0760 | 256 | exp03 | 2048bit-ECFP4 | SVM | 0.900 | 0.886 | 0.856 | 0.936 | 0.910 | 0.798 | 0.925 | 1 0.1 |
| M0761 | 256 | exp04 | 2048bit-ECFP4 | SVM | 0.907 | 0.893 | 0.864 | 0.943 | 0.917 | 0.814 | 0.917 | 5 0.1 |
| M0762 | 256 | exp05 | 2048bit-ECFP4 | SVM | 0.900 | 0.871 | 0.831 | 0.957 | 0.912 | 0.801 | 0.927 | 10000.0 0.1 |
| M0763 | 256 | exp06 | 2048bit-ECFP4 | SVM | 0.923 | 0.942 | 0.932 | 0.915 | 0.928 | 0.845 | 0.918 | 100000.0 0.1 |
| M0764 | 256 | exp07 | 2048bit-ECFP4 | SVM | 0.911 | 0.893 | 0.864 | 0.950 | 0.921 | 0.822 | 0.910 | 5 0.05 |
| M0765 | 256 | exp08 | 2048bit-ECFP4 | SVM | 0.927 | 0.930 | 0.915 | 0.936 | 0.933 | 0.852 | 0.914 | 5 0.1 |
| M0766 | 256 | exp09 | 2048bit-ECFP4 | SVM | 0.946 | 0.950 | 0.941 | 0.950 | 0.950 | 0.891 | 0.907 | 5 0.1 |
| M0767 | 256 | exp10 | 2048bit-ECFP4 | SVM | 0.911 | 0.893 | 0.864 | 0.950 | 0.921 | 0.822 | 0.913 | 5000.0 0.05 |
| M0768 | 256 | exp11 | 2048bit-ECFP4 | SVM | 0.927 | 0.942 | 0.932 | 0.922 | 0.932 | 0.853 | 0.919 | 1000.0 0.05 |
| M0769 | 256 | exp12 | 2048bit-ECFP4 | SVM | 0.900 | 0.914 | 0.898 | 0.901 | 0.907 | 0.798 | 0.924 | 1 0.1 |
| M0770 | 256 | exp13 | 2048bit-ECFP4 | SVM | 0.896 | 0.901 | 0.881 | 0.908 | 0.905 | 0.790 | 0.926 | 500 0.05 |
| M0771 | 256 | exp14 | 2048bit-ECFP4 | SVM | 0.903 | 0.887 | 0.856 | 0.943 | 0.914 | 0.806 | 0.924 | 1 0.1 |
| M0772 | 256 | exp15 | 2048bit-ECFP4 | SVM | 0.911 | 0.893 | 0.864 | 0.950 | 0.921 | 0.822 | 0.909 | 100 0.1 |
| M0773 | 256 | exp16 | 2048bit-ECFP4 | SVM | 0.931 | 0.936 | 0.924 | 0.936 | 0.936 | 0.860 | 0.924 | 100000.0 0.1 |
| M0774 | 256 | exp17 | 2048bit-ECFP4 | SVM | 0.903 | 0.914 | 0.898 | 0.908 | 0.911 | 0.806 | 0.925 | 50000.0 0.1 |
| M0775 | 256 | exp18 | 2048bit-ECFP4 | SVM | 0.919 | 0.929 | 0.915 | 0.922 | 0.925 | 0.837 | 0.917 | 10000.0 0.1 |
| M0776 | 256 | exp19 | 2048bit-ECFP4 | SVM | 0.931 | 0.918 | 0.898 | 0.957 | 0.938 | 0.860 | 0.928 | 1 0.1 |
| M0777 | 256 | exp01 | 2048bit-ECFP4 | GBM | 0.911 | 0.904 | 0.881 | 0.936 | 0.920 | 0.821 | 0.914 | 500 0.2 |
| M0778 | 256 | exp02 | 2048bit-ECFP4 | GBM | 0.873 | 0.842 | 0.788 | 0.943 | 0.890 | 0.747 | 0.909 | 300 0.4 |
| M0779 | 256 | exp03 | 2048bit-ECFP4 | GBM | 0.876 | 0.876 | 0.847 | 0.901 | 0.888 | 0.751 | 0.919 | 400 0.3 |
| M0780 | 256 | exp04 | 2048bit-ECFP4 | GBM | 0.892 | 0.884 | 0.856 | 0.922 | 0.903 | 0.782 | 0.908 | 1000 0.1 |
| M0781 | 256 | exp05 | 2048bit-ECFP4 | GBM | 0.911 | 0.893 | 0.864 | 0.950 | 0.921 | 0.822 | 0.919 | 400 0.3 |
| M0782 | 256 | exp06 | 2048bit-ECFP4 | GBM | 0.919 | 0.935 | 0.924 | 0.915 | 0.925 | 0.837 | 0.909 | 100 0.5 |
| M0783 | 256 | exp07 | 2048bit-ECFP4 | GBM | 0.884 | 0.894 | 0.873 | 0.894 | 0.894 | 0.766 | 0.906 | 300 0.8 |
| M0784 | 256 | exp08 | 2048bit-ECFP4 | GBM | 0.907 | 0.893 | 0.864 | 0.943 | 0.917 | 0.814 | 0.906 | 300 0.3 |
| M0785 | 256 | exp09 | 2048bit-ECFP4 | GBM | 0.934 | 0.937 | 0.924 | 0.943 | 0.940 | 0.868 | 0.905 | 700 0.2 |
| M0786 | 256 | exp10 | 2048bit-ECFP4 | GBM | 0.873 | 0.860 | 0.822 | 0.915 | 0.887 | 0.743 | 0.905 | 200 0.7 |
| M0787 | 256 | exp11 | 2048bit-ECFP4 | GBM | 0.915 | 0.910 | 0.890 | 0.936 | 0.923 | 0.829 | 0.915 | 200 0.5 |
| M0788 | 256 | exp12 | 2048bit-ECFP4 | GBM | 0.884 | 0.878 | 0.847 | 0.915 | 0.896 | 0.766 | 0.921 | 400 0.1 |
| M0789 | 256 | exp13 | 2048bit-ECFP4 | GBM | 0.861 | 0.862 | 0.831 | 0.887 | 0.874 | 0.719 | 0.919 | 800 0.1 |
| M0790 | 256 | exp14 | 2048bit-ECFP4 | GBM | 0.876 | 0.852 | 0.805 | 0.936 | 0.892 | 0.753 | 0.915 | 50 0.7 |
| M0791 | 256 | exp15 | 2048bit-ECFP4 | GBM | 0.896 | 0.880 | 0.847 | 0.936 | 0.907 | 0.790 | 0.906 | 200 0.2 |
| M0792 | 256 | exp16 | 2048bit-ECFP4 | GBM | 0.911 | 0.915 | 0.898 | 0.922 | 0.919 | 0.821 | 0.914 | 50 0.6 |
| M0793 | 256 | exp17 | 2048bit-ECFP4 | GBM | 0.892 | 0.895 | 0.873 | 0.908 | 0.901 | 0.782 | 0.920 | 200 0.4 |
| M0794 | 256 | exp18 | 2048bit-ECFP4 | GBM | 0.880 | 0.887 | 0.864 | 0.894 | 0.890 | 0.759 | 0.916 | 1000 0.1 |
| M0795 | 256 | exp19 | 2048bit-ECFP4 | GBM | 0.934 | 0.925 | 0.907 | 0.957 | 0.941 | 0.868 | 0.907 | 400 0.2 |
| M0796 | 256 | exp01 | 2048bit-ECFP4 | RF | 0.900 | 0.886 | 0.856 | 0.936 | 0.910 | 0.798 | 0.923 | 800 |
| M0797 | 256 | exp02 | 2048bit-ECFP4 | RF | 0.865 | 0.840 | 0.788 | 0.929 | 0.882 | 0.730 | 0.916 | 200 |
| M0798 | 256 | exp03 | 2048bit-ECFP4 | RF | 0.892 | 0.855 | 0.805 | 0.965 | 0.907 | 0.787 | 0.925 | 50 |
| M0799 | 256 | exp04 | 2048bit-ECFP4 | RF | 0.911 | 0.888 | 0.856 | 0.957 | 0.922 | 0.823 | 0.917 | 400 |
| M0800 | 256 | exp05 | 2048bit-ECFP4 | RF | 0.903 | 0.877 | 0.839 | 0.957 | 0.915 | 0.808 | 0.923 | 100 |
| M0801 | 256 | exp06 | 2048bit-ECFP4 | RF | 0.923 | 0.929 | 0.915 | 0.929 | 0.929 | 0.844 | 0.919 | 500 |
| M0802 | 256 | exp07 | 2048bit-ECFP4 | RF | 0.907 | 0.887 | 0.856 | 0.950 | 0.918 | 0.814 | 0.906 | 700 |
| M0803 | 256 | exp08 | 2048bit-ECFP4 | RF | 0.923 | 0.906 | 0.881 | 0.957 | 0.931 | 0.845 | 0.915 | 100 |
| M0804 | 256 | exp09 | 2048bit-ECFP4 | RF | 0.942 | 0.926 | 0.907 | 0.972 | 0.948 | 0.884 | 0.910 | 600 |
| M0805 | 256 | exp10 | 2048bit-ECFP4 | RF | 0.911 | 0.878 | 0.839 | 0.972 | 0.923 | 0.825 | 0.914 | 300 |
| M0806 | 256 | exp11 | 2048bit-ECFP4 | RF | 0.923 | 0.917 | 0.898 | 0.943 | 0.930 | 0.844 | 0.914 | 600 |
| M0807 | 256 | exp12 | 2048bit-ECFP4 | RF | 0.903 | 0.903 | 0.881 | 0.922 | 0.912 | 0.805 | 0.922 | 900 |
| M0808 | 256 | exp13 | 2048bit-ECFP4 | RF | 0.892 | 0.874 | 0.839 | 0.936 | 0.904 | 0.783 | 0.926 | 100 |
| M0809 | 256 | exp14 | 2048bit-ECFP4 | RF | 0.888 | 0.859 | 0.814 | 0.950 | 0.902 | 0.777 | 0.928 | 900 |
| M0810 | 256 | exp15 | 2048bit-ECFP4 | RF | 0.888 | 0.854 | 0.805 | 0.957 | 0.903 | 0.779 | 0.913 | 10 |
| M0811 | 256 | exp16 | 2048bit-ECFP4 | RF | 0.919 | 0.917 | 0.898 | 0.936 | 0.926 | 0.836 | 0.917 | 200 |
| M0812 | 256 | exp17 | 2048bit-ECFP4 | RF | 0.900 | 0.914 | 0.898 | 0.901 | 0.907 | 0.798 | 0.923 | 900 |
| M0813 | 256 | exp18 | 2048bit-ECFP4 | RF | 0.923 | 0.935 | 0.924 | 0.922 | 0.929 | 0.845 | 0.921 | 600 |
| M0814 | 256 | exp19 | 2048bit-ECFP4 | RF | 0.934 | 0.913 | 0.890 | 0.972 | 0.942 | 0.869 | 0.923 | 500 |
| M0815 | 256 | exp01 | 2048bit-ECFP4 | DNN2 | 0.923 | 0.929 | 0.915 | 0.929 | 0.929 | 0.844 | 0.931 | 100 120 0.4 |
| M0816 | 256 | exp02 | 2048bit-ECFP4 | DNN2 | 0.880 | 0.877 | 0.847 | 0.908 | 0.892 | 0.758 | 0.922 | 100 80 0.5 |
| M0817 | 256 | exp03 | 2048bit-ECFP4 | DNN2 | 0.873 | 0.875 | 0.847 | 0.894 | 0.884 | 0.743 | 0.921 | 200 100 0.1 |
| M0818 | 256 | exp01 | 2048bit-ECFP4 | DNN3 | 0.915 | 0.922 | 0.907 | 0.922 | 0.922 | 0.829 | 0.931 | 300 160 0.5 |
| M0819 | 256 | exp02 | 2048bit-ECFP4 | DNN3 | 0.865 | 0.858 | 0.822 | 0.901 | 0.879 | 0.727 | 0.924 | 500 100 0.1 |
| M0820 | 256 | exp03 | 2048bit-ECFP4 | DNN3 | 0.884 | 0.883 | 0.856 | 0.908 | 0.895 | 0.766 | 0.925 | 100 100 0.4 |
| M0821 | 256 | exp01 | 1024bit-ECFP6 | KNN | 0.876 | 0.871 | 0.839 | 0.908 | 0.889 | 0.751 | 0.921 | 3 uniform |
| M0822 | 256 | exp02 | 1024bit-ECFP6 | KNN | 0.873 | 0.870 | 0.839 | 0.901 | 0.885 | 0.743 | 0.915 | 3 uniform |
| M0823 | 256 | exp03 | 1024bit-ECFP6 | KNN | 0.911 | 0.888 | 0.856 | 0.957 | 0.922 | 0.823 | 0.916 | 3 uniform |
| M0824 | 256 | exp04 | 1024bit-ECFP6 | KNN | 0.900 | 0.871 | 0.831 | 0.957 | 0.912 | 0.801 | 0.912 | 5 distance |
| M0825 | 256 | exp05 | 1024bit-ECFP6 | KNN | 0.915 | 0.894 | 0.864 | 0.957 | 0.925 | 0.830 | 0.916 | 3 distance |
| M0826 | 256 | exp06 | 1024bit-ECFP6 | KNN | 0.907 | 0.933 | 0.924 | 0.894 | 0.913 | 0.815 | 0.913 | 3 uniform |
| M0827 | 256 | exp07 | 1024bit-ECFP6 | KNN | 0.915 | 0.899 | 0.873 | 0.950 | 0.924 | 0.829 | 0.904 | 5 uniform |
| M0828 | 256 | exp08 | 1024bit-ECFP6 | KNN | 0.927 | 0.924 | 0.907 | 0.943 | 0.933 | 0.852 | 0.910 | 3 uniform |
| M0829 | 256 | exp09 | 1024bit-ECFP6 | KNN | 0.923 | 0.935 | 0.924 | 0.922 | 0.929 | 0.845 | 0.906 | 3 uniform |
| M0830 | 256 | exp10 | 1024bit-ECFP6 | KNN | 0.911 | 0.888 | 0.856 | 0.957 | 0.922 | 0.823 | 0.907 | 3 distance |
| M0831 | 256 | exp11 | 1024bit-ECFP6 | KNN | 0.911 | 0.910 | 0.890 | 0.929 | 0.919 | 0.821 | 0.912 | 3 distance |
| M0832 | 256 | exp12 | 1024bit-ECFP6 | KNN | 0.896 | 0.907 | 0.890 | 0.901 | 0.904 | 0.790 | 0.920 | 3 uniform |
| M0833 | 256 | exp13 | 1024bit-ECFP6 | KNN | 0.876 | 0.866 | 0.831 | 0.915 | 0.890 | 0.751 | 0.918 | 5 distance |
| M0834 | 256 | exp14 | 1024bit-ECFP6 | KNN | 0.903 | 0.882 | 0.847 | 0.950 | 0.915 | 0.807 | 0.909 | 5 uniform |
| M0835 | 256 | exp15 | 1024bit-ECFP6 | KNN | 0.888 | 0.878 | 0.847 | 0.922 | 0.900 | 0.774 | 0.908 | 5 distance |
| M0836 | 256 | exp16 | 1024bit-ECFP6 | KNN | 0.915 | 0.910 | 0.890 | 0.936 | 0.923 | 0.829 | 0.913 | 3 distance |
| M0837 | 256 | exp17 | 1024bit-ECFP6 | KNN | 0.927 | 0.918 | 0.898 | 0.950 | 0.934 | 0.852 | 0.916 | 3 distance |
| M0838 | 256 | exp18 | 1024bit-ECFP6 | KNN | 0.919 | 0.941 | 0.932 | 0.908 | 0.924 | 0.838 | 0.895 | 3 uniform |
| M0839 | 256 | exp19 | 1024bit-ECFP6 | KNN | 0.923 | 0.929 | 0.915 | 0.929 | 0.929 | 0.844 | 0.909 | 3 uniform |
| M0840 | 256 | exp01 | 1024bit-ECFP6 | SVM | 0.915 | 0.916 | 0.898 | 0.929 | 0.923 | 0.829 | 0.926 | 5 0.1 |
| M0841 | 256 | exp02 | 1024bit-ECFP6 | SVM | 0.880 | 0.872 | 0.839 | 0.915 | 0.893 | 0.759 | 0.917 | 100000.0 0.05 |
| M0842 | 256 | exp03 | 1024bit-ECFP6 | SVM | 0.900 | 0.886 | 0.856 | 0.936 | 0.910 | 0.798 | 0.923 | 50000.0 0.1 |
| M0843 | 256 | exp04 | 1024bit-ECFP6 | SVM | 0.915 | 0.899 | 0.873 | 0.950 | 0.924 | 0.829 | 0.918 | 50 0.1 |
| M0844 | 256 | exp05 | 1024bit-ECFP6 | SVM | 0.907 | 0.873 | 0.831 | 0.972 | 0.919 | 0.818 | 0.919 | 10 0.1 |
| M0845 | 256 | exp06 | 1024bit-ECFP6 | SVM | 0.931 | 0.949 | 0.941 | 0.922 | 0.935 | 0.861 | 0.919 | 5000.0 0.1 |
| M0846 | 256 | exp07 | 1024bit-ECFP6 | SVM | 0.931 | 0.913 | 0.890 | 0.965 | 0.938 | 0.861 | 0.912 | 1000.0 0.1 |
| M0847 | 256 | exp08 | 1024bit-ECFP6 | SVM | 0.934 | 0.937 | 0.924 | 0.943 | 0.940 | 0.868 | 0.910 | 5000.0 0.1 |
| M0848 | 256 | exp09 | 1024bit-ECFP6 | SVM | 0.934 | 0.931 | 0.915 | 0.950 | 0.940 | 0.868 | 0.908 | 1 0.1 |
| M0849 | 256 | exp10 | 1024bit-ECFP6 | SVM | 0.915 | 0.884 | 0.847 | 0.972 | 0.926 | 0.832 | 0.915 | 5 0.1 |
| M0850 | 256 | exp11 | 1024bit-ECFP6 | SVM | 0.915 | 0.894 | 0.864 | 0.957 | 0.925 | 0.830 | 0.912 | 10000.0 0.1 |
| M0851 | 256 | exp12 | 1024bit-ECFP6 | SVM | 0.896 | 0.907 | 0.890 | 0.901 | 0.904 | 0.790 | 0.922 | 5 0.05 |
| M0852 | 256 | exp13 | 1024bit-ECFP6 | SVM | 0.900 | 0.891 | 0.864 | 0.929 | 0.910 | 0.798 | 0.919 | 50000.0 0.1 |
| M0853 | 256 | exp14 | 1024bit-ECFP6 | SVM | 0.907 | 0.882 | 0.847 | 0.957 | 0.918 | 0.815 | 0.923 | 10000.0 0.1 |
| M0854 | 256 | exp15 | 1024bit-ECFP6 | SVM | 0.896 | 0.890 | 0.864 | 0.922 | 0.906 | 0.790 | 0.914 | 1 0.1 |
| M0855 | 256 | exp16 | 1024bit-ECFP6 | SVM | 0.931 | 0.913 | 0.890 | 0.965 | 0.938 | 0.861 | 0.919 | 500 0.1 |
| M0856 | 256 | exp17 | 1024bit-ECFP6 | SVM | 0.911 | 0.915 | 0.898 | 0.922 | 0.919 | 0.821 | 0.923 | 50000.0 0.1 |
| M0857 | 256 | exp18 | 1024bit-ECFP6 | SVM | 0.923 | 0.935 | 0.924 | 0.922 | 0.929 | 0.845 | 0.910 | 100000.0 0.1 |
| M0858 | 256 | exp19 | 1024bit-ECFP6 | SVM | 0.919 | 0.911 | 0.890 | 0.943 | 0.927 | 0.837 | 0.923 | 100 0.1 |
| M0859 | 256 | exp01 | 1024bit-ECFP6 | GBM | 0.873 | 0.880 | 0.856 | 0.887 | 0.883 | 0.743 | 0.908 | 300 0.5 |
| M0860 | 256 | exp02 | 1024bit-ECFP6 | GBM | 0.884 | 0.883 | 0.856 | 0.908 | 0.895 | 0.766 | 0.901 | 200 0.8 |
| M0861 | 256 | exp03 | 1024bit-ECFP6 | GBM | 0.880 | 0.872 | 0.839 | 0.915 | 0.893 | 0.759 | 0.915 | 700 0.2 |
| M0862 | 256 | exp04 | 1024bit-ECFP6 | GBM | 0.919 | 0.900 | 0.873 | 0.957 | 0.928 | 0.838 | 0.901 | 300 0.2 |
| M0863 | 256 | exp05 | 1024bit-ECFP6 | GBM | 0.888 | 0.868 | 0.831 | 0.936 | 0.901 | 0.775 | 0.907 | 300 0.8 |
| M0864 | 256 | exp06 | 1024bit-ECFP6 | GBM | 0.915 | 0.916 | 0.898 | 0.929 | 0.923 | 0.829 | 0.913 | 400 0.2 |
| M0865 | 256 | exp07 | 1024bit-ECFP6 | GBM | 0.923 | 0.895 | 0.864 | 0.972 | 0.932 | 0.847 | 0.897 | 400 0.1 |
| M0866 | 256 | exp08 | 1024bit-ECFP6 | GBM | 0.900 | 0.897 | 0.873 | 0.922 | 0.909 | 0.797 | 0.902 | 500 0.3 |
| M0867 | 256 | exp09 | 1024bit-ECFP6 | GBM | 0.907 | 0.921 | 0.907 | 0.908 | 0.914 | 0.814 | 0.897 | 600 0.3 |
| M0868 | 256 | exp10 | 1024bit-ECFP6 | GBM | 0.907 | 0.882 | 0.847 | 0.957 | 0.918 | 0.815 | 0.904 | 300 0.1 |
| M0869 | 256 | exp11 | 1024bit-ECFP6 | GBM | 0.900 | 0.891 | 0.864 | 0.929 | 0.910 | 0.798 | 0.899 | 300 0.4 |
| M0870 | 256 | exp12 | 1024bit-ECFP6 | GBM | 0.888 | 0.889 | 0.864 | 0.908 | 0.898 | 0.774 | 0.908 | 300 0.1 |
| M0871 | 256 | exp13 | 1024bit-ECFP6 | GBM | 0.876 | 0.881 | 0.856 | 0.894 | 0.887 | 0.751 | 0.913 | 300 0.8 |
| M0872 | 256 | exp14 | 1024bit-ECFP6 | GBM | 0.884 | 0.858 | 0.814 | 0.943 | 0.899 | 0.769 | 0.905 | 200 0.1 |
| M0873 | 256 | exp15 | 1024bit-ECFP6 | GBM | 0.880 | 0.867 | 0.831 | 0.922 | 0.893 | 0.759 | 0.899 | 400 0.3 |
| M0874 | 256 | exp16 | 1024bit-ECFP6 | GBM | 0.907 | 0.877 | 0.839 | 0.965 | 0.919 | 0.816 | 0.902 | 700 0.3 |
| M0875 | 256 | exp17 | 1024bit-ECFP6 | GBM | 0.896 | 0.901 | 0.881 | 0.908 | 0.905 | 0.790 | 0.911 | 600 0.1 |
| M0876 | 256 | exp18 | 1024bit-ECFP6 | GBM | 0.896 | 0.919 | 0.907 | 0.887 | 0.903 | 0.791 | 0.892 | 300 0.7 |
| M0877 | 256 | exp19 | 1024bit-ECFP6 | GBM | 0.927 | 0.918 | 0.898 | 0.950 | 0.934 | 0.852 | 0.904 | 300 0.1 |
| M0878 | 256 | exp01 | 1024bit-ECFP6 | RF | 0.892 | 0.890 | 0.864 | 0.915 | 0.902 | 0.782 | 0.920 | 1000 |
| M0879 | 256 | exp02 | 1024bit-ECFP6 | RF | 0.865 | 0.840 | 0.788 | 0.929 | 0.882 | 0.730 | 0.912 | 400 |
| M0880 | 256 | exp03 | 1024bit-ECFP6 | RF | 0.896 | 0.861 | 0.814 | 0.965 | 0.910 | 0.795 | 0.924 | 1000 |
| M0881 | 256 | exp04 | 1024bit-ECFP6 | RF | 0.911 | 0.878 | 0.839 | 0.972 | 0.923 | 0.825 | 0.913 | 200 |
| M0882 | 256 | exp05 | 1024bit-ECFP6 | RF | 0.900 | 0.866 | 0.822 | 0.965 | 0.913 | 0.802 | 0.913 | 1000 |
| M0883 | 256 | exp06 | 1024bit-ECFP6 | RF | 0.915 | 0.910 | 0.890 | 0.936 | 0.923 | 0.829 | 0.915 | 50 |
| M0884 | 256 | exp07 | 1024bit-ECFP6 | RF | 0.919 | 0.890 | 0.856 | 0.972 | 0.929 | 0.839 | 0.909 | 500 |
| M0885 | 256 | exp08 | 1024bit-ECFP6 | RF | 0.915 | 0.899 | 0.873 | 0.950 | 0.924 | 0.829 | 0.910 | 700 |
| M0886 | 256 | exp09 | 1024bit-ECFP6 | RF | 0.938 | 0.914 | 0.890 | 0.979 | 0.945 | 0.877 | 0.907 | 200 |
| M0887 | 256 | exp10 | 1024bit-ECFP6 | RF | 0.907 | 0.873 | 0.831 | 0.972 | 0.919 | 0.818 | 0.914 | 600 |
| M0888 | 256 | exp11 | 1024bit-ECFP6 | RF | 0.911 | 0.893 | 0.864 | 0.950 | 0.921 | 0.822 | 0.910 | 300 |
| M0889 | 256 | exp12 | 1024bit-ECFP6 | RF | 0.900 | 0.902 | 0.881 | 0.915 | 0.908 | 0.797 | 0.921 | 200 |
| M0890 | 256 | exp13 | 1024bit-ECFP6 | RF | 0.892 | 0.879 | 0.847 | 0.929 | 0.903 | 0.782 | 0.913 | 100 |
| M0891 | 256 | exp14 | 1024bit-ECFP6 | RF | 0.903 | 0.867 | 0.822 | 0.972 | 0.916 | 0.810 | 0.923 | 600 |
| M0892 | 256 | exp15 | 1024bit-ECFP6 | RF | 0.896 | 0.875 | 0.839 | 0.943 | 0.908 | 0.791 | 0.910 | 200 |
| M0893 | 256 | exp16 | 1024bit-ECFP6 | RF | 0.923 | 0.906 | 0.881 | 0.957 | 0.931 | 0.845 | 0.912 | 300 |
| M0894 | 256 | exp17 | 1024bit-ECFP6 | RF | 0.900 | 0.897 | 0.873 | 0.922 | 0.909 | 0.797 | 0.916 | 1000 |
| M0895 | 256 | exp18 | 1024bit-ECFP6 | RF | 0.919 | 0.929 | 0.915 | 0.922 | 0.925 | 0.837 | 0.904 | 100 |
| M0896 | 256 | exp19 | 1024bit-ECFP6 | RF | 0.931 | 0.907 | 0.881 | 0.972 | 0.938 | 0.862 | 0.919 | 800 |
| M0897 | 256 | exp01 | 1024bit-ECFP6 | DNN2 | 0.869 | 0.879 | 0.856 | 0.879 | 0.879 | 0.735 | 0.916 | 100 80 0.1 |
| M0898 | 256 | exp02 | 1024bit-ECFP6 | DNN2 | 0.876 | 0.881 | 0.856 | 0.894 | 0.887 | 0.751 | 0.912 | 500 140 0.3 |
| M0899 | 256 | exp03 | 1024bit-ECFP6 | DNN2 | 0.861 | 0.867 | 0.839 | 0.879 | 0.873 | 0.719 | 0.912 | 300 100 0.5 |
| M0900 | 256 | exp01 | 1024bit-ECFP6 | DNN3 | 0.869 | 0.885 | 0.864 | 0.872 | 0.879 | 0.736 | 0.915 | 200 160 0.2 |
| M0901 | 256 | exp02 | 1024bit-ECFP6 | DNN3 | 0.876 | 0.898 | 0.881 | 0.872 | 0.885 | 0.752 | 0.910 | 600 120 0.5 |
| M0902 | 256 | exp03 | 1024bit-ECFP6 | DNN3 | 0.884 | 0.888 | 0.864 | 0.901 | 0.894 | 0.766 | 0.919 | 300 60 0.4 |
| M0903 | 256 | exp01 | 2048bit-ECFP6 | KNN | 0.900 | 0.891 | 0.864 | 0.929 | 0.910 | 0.798 | 0.926 | 3 uniform |
| M0904 | 256 | exp02 | 2048bit-ECFP6 | KNN | 0.880 | 0.872 | 0.839 | 0.915 | 0.893 | 0.759 | 0.919 | 3 uniform |
| M0905 | 256 | exp03 | 2048bit-ECFP6 | KNN | 0.911 | 0.873 | 0.831 | 0.979 | 0.923 | 0.826 | 0.920 | 3 distance |
| M0906 | 256 | exp04 | 2048bit-ECFP6 | KNN | 0.903 | 0.867 | 0.822 | 0.972 | 0.916 | 0.810 | 0.913 | 5 distance |
| M0907 | 256 | exp05 | 2048bit-ECFP6 | KNN | 0.911 | 0.883 | 0.847 | 0.965 | 0.922 | 0.824 | 0.915 | 3 distance |
| M0908 | 256 | exp06 | 2048bit-ECFP6 | KNN | 0.907 | 0.927 | 0.915 | 0.901 | 0.914 | 0.814 | 0.913 | 3 uniform |
| M0909 | 256 | exp07 | 2048bit-ECFP6 | KNN | 0.927 | 0.901 | 0.873 | 0.972 | 0.935 | 0.854 | 0.908 | 5 uniform |
| M0910 | 256 | exp08 | 2048bit-ECFP6 | KNN | 0.919 | 0.905 | 0.881 | 0.950 | 0.927 | 0.837 | 0.911 | 3 uniform |
| M0911 | 256 | exp09 | 2048bit-ECFP6 | KNN | 0.911 | 0.888 | 0.856 | 0.957 | 0.922 | 0.823 | 0.909 | 5 distance |
| M0912 | 256 | exp10 | 2048bit-ECFP6 | KNN | 0.915 | 0.889 | 0.856 | 0.965 | 0.925 | 0.831 | 0.912 | 5 uniform |
| M0913 | 256 | exp11 | 2048bit-ECFP6 | KNN | 0.903 | 0.892 | 0.864 | 0.936 | 0.913 | 0.806 | 0.912 | 3 uniform |
| M0914 | 256 | exp12 | 2048bit-ECFP6 | KNN | 0.896 | 0.896 | 0.873 | 0.915 | 0.905 | 0.790 | 0.916 | 3 uniform |
| M0915 | 256 | exp13 | 2048bit-ECFP6 | KNN | 0.876 | 0.871 | 0.839 | 0.908 | 0.889 | 0.751 | 0.920 | 3 uniform |
| M0916 | 256 | exp14 | 2048bit-ECFP6 | KNN | 0.907 | 0.882 | 0.847 | 0.957 | 0.918 | 0.815 | 0.918 | 5 distance |
| M0917 | 256 | exp15 | 2048bit-ECFP6 | KNN | 0.888 | 0.873 | 0.839 | 0.929 | 0.900 | 0.775 | 0.908 | 5 distance |
| M0918 | 256 | exp16 | 2048bit-ECFP6 | KNN | 0.903 | 0.903 | 0.881 | 0.922 | 0.912 | 0.805 | 0.917 | 3 distance |
| M0919 | 256 | exp17 | 2048bit-ECFP6 | KNN | 0.903 | 0.914 | 0.898 | 0.908 | 0.911 | 0.806 | 0.921 | 3 uniform |
| M0920 | 256 | exp18 | 2048bit-ECFP6 | KNN | 0.931 | 0.942 | 0.932 | 0.929 | 0.936 | 0.860 | 0.912 | 3 distance |
| M0921 | 256 | exp19 | 2048bit-ECFP6 | KNN | 0.950 | 0.932 | 0.915 | 0.979 | 0.955 | 0.900 | 0.910 | 5 distance |
| M0922 | 256 | exp01 | 2048bit-ECFP6 | SVM | 0.911 | 0.921 | 0.907 | 0.915 | 0.918 | 0.821 | 0.922 | 5 0.1 |
| M0923 | 256 | exp02 | 2048bit-ECFP6 | SVM | 0.876 | 0.887 | 0.864 | 0.887 | 0.887 | 0.751 | 0.919 | 1000.0 0.05 |
| M0924 | 256 | exp03 | 2048bit-ECFP6 | SVM | 0.896 | 0.890 | 0.864 | 0.922 | 0.906 | 0.790 | 0.923 | 100 0.1 |
| M0925 | 256 | exp04 | 2048bit-ECFP6 | SVM | 0.907 | 0.893 | 0.864 | 0.943 | 0.917 | 0.814 | 0.910 | 100000.0 0.1 |
| M0926 | 256 | exp05 | 2048bit-ECFP6 | SVM | 0.888 | 0.868 | 0.831 | 0.936 | 0.901 | 0.775 | 0.918 | 500 0.1 |
| M0927 | 256 | exp06 | 2048bit-ECFP6 | SVM | 0.915 | 0.941 | 0.932 | 0.901 | 0.920 | 0.830 | 0.922 | 5 0.1 |
| M0928 | 256 | exp07 | 2048bit-ECFP6 | SVM | 0.892 | 0.879 | 0.847 | 0.929 | 0.903 | 0.782 | 0.910 | 5 0.01 |
| M0929 | 256 | exp08 | 2048bit-ECFP6 | SVM | 0.919 | 0.917 | 0.898 | 0.936 | 0.926 | 0.836 | 0.912 | 1 0.1 |
| M0930 | 256 | exp09 | 2048bit-ECFP6 | SVM | 0.927 | 0.936 | 0.924 | 0.929 | 0.932 | 0.852 | 0.911 | 5000.0 0.1 |
| M0931 | 256 | exp10 | 2048bit-ECFP6 | SVM | 0.900 | 0.881 | 0.847 | 0.943 | 0.911 | 0.799 | 0.918 | 5 0.1 |
| M0932 | 256 | exp11 | 2048bit-ECFP6 | SVM | 0.919 | 0.917 | 0.898 | 0.936 | 0.926 | 0.836 | 0.921 | 5 0.1 |
| M0933 | 256 | exp12 | 2048bit-ECFP6 | SVM | 0.892 | 0.901 | 0.881 | 0.901 | 0.901 | 0.782 | 0.924 | 1 0.1 |
| M0934 | 256 | exp13 | 2048bit-ECFP6 | SVM | 0.888 | 0.878 | 0.847 | 0.922 | 0.900 | 0.774 | 0.921 | 1 0.1 |
| M0935 | 256 | exp14 | 2048bit-ECFP6 | SVM | 0.907 | 0.893 | 0.864 | 0.943 | 0.917 | 0.814 | 0.927 | 1 0.1 |
| M0936 | 256 | exp15 | 2048bit-ECFP6 | SVM | 0.915 | 0.905 | 0.881 | 0.943 | 0.924 | 0.829 | 0.912 | 1 0.1 |
| M0937 | 256 | exp16 | 2048bit-ECFP6 | SVM | 0.927 | 0.918 | 0.898 | 0.950 | 0.934 | 0.852 | 0.922 | 5 0.1 |
| M0938 | 256 | exp17 | 2048bit-ECFP6 | SVM | 0.923 | 0.935 | 0.924 | 0.922 | 0.929 | 0.845 | 0.922 | 5 0.05 |
| M0939 | 256 | exp18 | 2048bit-ECFP6 | SVM | 0.927 | 0.955 | 0.949 | 0.908 | 0.931 | 0.854 | 0.917 | 5 0.1 |
| M0940 | 256 | exp19 | 2048bit-ECFP6 | SVM | 0.931 | 0.918 | 0.898 | 0.957 | 0.938 | 0.860 | 0.929 | 1 0.1 |
| M0941 | 256 | exp01 | 2048bit-ECFP6 | GBM | 0.915 | 0.905 | 0.881 | 0.943 | 0.924 | 0.829 | 0.916 | 900 0.2 |
| M0942 | 256 | exp02 | 2048bit-ECFP6 | GBM | 0.861 | 0.848 | 0.805 | 0.908 | 0.877 | 0.720 | 0.920 | 100 0.7 |
| M0943 | 256 | exp03 | 2048bit-ECFP6 | GBM | 0.888 | 0.873 | 0.839 | 0.929 | 0.900 | 0.775 | 0.919 | 200 0.6 |
| M0944 | 256 | exp04 | 2048bit-ECFP6 | GBM | 0.884 | 0.863 | 0.822 | 0.936 | 0.898 | 0.768 | 0.900 | 200 0.2 |
| M0945 | 256 | exp05 | 2048bit-ECFP6 | GBM | 0.907 | 0.898 | 0.873 | 0.936 | 0.917 | 0.813 | 0.915 | 400 0.3 |
| M0946 | 256 | exp06 | 2048bit-ECFP6 | GBM | 0.892 | 0.901 | 0.881 | 0.901 | 0.901 | 0.782 | 0.910 | 200 0.2 |
| M0947 | 256 | exp07 | 2048bit-ECFP6 | GBM | 0.911 | 0.893 | 0.864 | 0.950 | 0.921 | 0.822 | 0.907 | 300 0.2 |
| M0948 | 256 | exp08 | 2048bit-ECFP6 | GBM | 0.896 | 0.880 | 0.847 | 0.936 | 0.907 | 0.790 | 0.904 | 200 0.2 |
| M0949 | 256 | exp09 | 2048bit-ECFP6 | GBM | 0.931 | 0.936 | 0.924 | 0.936 | 0.936 | 0.860 | 0.899 | 400 0.2 |
| M0950 | 256 | exp10 | 2048bit-ECFP6 | GBM | 0.896 | 0.890 | 0.864 | 0.922 | 0.906 | 0.790 | 0.907 | 400 0.5 |
| M0951 | 256 | exp11 | 2048bit-ECFP6 | GBM | 0.896 | 0.890 | 0.864 | 0.922 | 0.906 | 0.790 | 0.908 | 300 0.8 |
| M0952 | 256 | exp12 | 2048bit-ECFP6 | GBM | 0.884 | 0.878 | 0.847 | 0.915 | 0.896 | 0.766 | 0.909 | 1000 0.1 |
| M0953 | 256 | exp13 | 2048bit-ECFP6 | GBM | 0.861 | 0.857 | 0.822 | 0.894 | 0.875 | 0.719 | 0.908 | 200 0.3 |
| M0954 | 256 | exp14 | 2048bit-ECFP6 | GBM | 0.888 | 0.864 | 0.822 | 0.943 | 0.902 | 0.776 | 0.909 | 500 0.1 |
| M0955 | 256 | exp15 | 2048bit-ECFP6 | GBM | 0.911 | 0.899 | 0.873 | 0.943 | 0.920 | 0.821 | 0.905 | 500 0.1 |
| M0956 | 256 | exp16 | 2048bit-ECFP6 | GBM | 0.892 | 0.874 | 0.839 | 0.936 | 0.904 | 0.783 | 0.917 | 100 0.2 |
| M0957 | 256 | exp17 | 2048bit-ECFP6 | GBM | 0.903 | 0.903 | 0.881 | 0.922 | 0.912 | 0.805 | 0.912 | 700 0.1 |
| M0958 | 256 | exp18 | 2048bit-ECFP6 | GBM | 0.915 | 0.922 | 0.907 | 0.922 | 0.922 | 0.829 | 0.910 | 100 0.6 |
| M0959 | 256 | exp19 | 2048bit-ECFP6 | GBM | 0.923 | 0.917 | 0.898 | 0.943 | 0.930 | 0.844 | 0.903 | 200 0.6 |
| M0960 | 256 | exp01 | 2048bit-ECFP6 | RF | 0.896 | 0.880 | 0.847 | 0.936 | 0.907 | 0.790 | 0.924 | 800 |
| M0961 | 256 | exp02 | 2048bit-ECFP6 | RF | 0.857 | 0.833 | 0.780 | 0.922 | 0.875 | 0.714 | 0.919 | 200 |
| M0962 | 256 | exp03 | 2048bit-ECFP6 | RF | 0.892 | 0.855 | 0.805 | 0.965 | 0.907 | 0.787 | 0.929 | 200 |
| M0963 | 256 | exp04 | 2048bit-ECFP6 | RF | 0.911 | 0.888 | 0.856 | 0.957 | 0.922 | 0.823 | 0.918 | 400 |
| M0964 | 256 | exp05 | 2048bit-ECFP6 | RF | 0.907 | 0.887 | 0.856 | 0.950 | 0.918 | 0.814 | 0.919 | 100 |
| M0965 | 256 | exp06 | 2048bit-ECFP6 | RF | 0.915 | 0.922 | 0.907 | 0.922 | 0.922 | 0.829 | 0.923 | 700 |
| M0966 | 256 | exp07 | 2048bit-ECFP6 | RF | 0.923 | 0.901 | 0.873 | 0.965 | 0.932 | 0.846 | 0.910 | 700 |
| M0967 | 256 | exp08 | 2048bit-ECFP6 | RF | 0.923 | 0.917 | 0.898 | 0.943 | 0.930 | 0.844 | 0.907 | 900 |
| M0968 | 256 | exp09 | 2048bit-ECFP6 | RF | 0.950 | 0.938 | 0.924 | 0.972 | 0.955 | 0.899 | 0.909 | 1000 |
| M0969 | 256 | exp10 | 2048bit-ECFP6 | RF | 0.900 | 0.871 | 0.831 | 0.957 | 0.912 | 0.801 | 0.917 | 500 |
| M0970 | 256 | exp11 | 2048bit-ECFP6 | RF | 0.923 | 0.912 | 0.890 | 0.950 | 0.931 | 0.845 | 0.917 | 500 |
| M0971 | 256 | exp12 | 2048bit-ECFP6 | RF | 0.915 | 0.905 | 0.881 | 0.943 | 0.924 | 0.829 | 0.928 | 100 |
| M0972 | 256 | exp13 | 2048bit-ECFP6 | RF | 0.880 | 0.857 | 0.814 | 0.936 | 0.895 | 0.761 | 0.920 | 50 |
| M0973 | 256 | exp14 | 2048bit-ECFP6 | RF | 0.896 | 0.865 | 0.822 | 0.957 | 0.909 | 0.793 | 0.928 | 200 |
| M0974 | 256 | exp15 | 2048bit-ECFP6 | RF | 0.903 | 0.882 | 0.847 | 0.950 | 0.915 | 0.807 | 0.912 | 50 |
| M0975 | 256 | exp16 | 2048bit-ECFP6 | RF | 0.911 | 0.899 | 0.873 | 0.943 | 0.920 | 0.821 | 0.915 | 800 |
| M0976 | 256 | exp17 | 2048bit-ECFP6 | RF | 0.896 | 0.896 | 0.873 | 0.915 | 0.905 | 0.790 | 0.924 | 500 |
| M0977 | 256 | exp18 | 2048bit-ECFP6 | RF | 0.923 | 0.935 | 0.924 | 0.922 | 0.929 | 0.845 | 0.911 | 600 |
| M0978 | 256 | exp19 | 2048bit-ECFP6 | RF | 0.938 | 0.919 | 0.898 | 0.972 | 0.945 | 0.876 | 0.921 | 700 |
| M0979 | 256 | exp01 | 2048bit-ECFP6 | DNN2 | 0.888 | 0.894 | 0.873 | 0.901 | 0.898 | 0.774 | 0.921 | 100 100 0.1 |
| M0980 | 256 | exp02 | 2048bit-ECFP6 | DNN2 | 0.869 | 0.891 | 0.873 | 0.865 | 0.878 | 0.736 | 0.916 | 600 100 0.2 |
| M0981 | 256 | exp03 | 2048bit-ECFP6 | DNN2 | 0.892 | 0.901 | 0.881 | 0.901 | 0.901 | 0.782 | 0.915 | 500 140 0.4 |
| M0982 | 256 | exp01 | 2048bit-ECFP6 | DNN3 | 0.880 | 0.899 | 0.881 | 0.879 | 0.889 | 0.759 | 0.924 | 600 120 0.1 |
| M0983 | 256 | exp02 | 2048bit-ECFP6 | DNN3 | 0.861 | 0.867 | 0.839 | 0.879 | 0.873 | 0.719 | 0.920 | 300 60 0.3 |
| M0984 | 256 | exp03 | 2048bit-ECFP6 | DNN3 | 0.865 | 0.858 | 0.822 | 0.901 | 0.879 | 0.727 | 0.917 | 500 100 0.1 |
| M0985 | 128 | exp01 | 1024bit-ECFP4 | KNN | 0.892 | 0.874 | 0.839 | 0.936 | 0.904 | 0.783 | 0.926 | 3 uniform |
| M0986 | 128 | exp02 | 1024bit-ECFP4 | KNN | 0.880 | 0.877 | 0.847 | 0.908 | 0.892 | 0.758 | 0.920 | 3 uniform |
| M0987 | 128 | exp03 | 1024bit-ECFP4 | KNN | 0.876 | 0.852 | 0.805 | 0.936 | 0.892 | 0.753 | 0.917 | 3 distance |
| M0988 | 128 | exp04 | 1024bit-ECFP4 | KNN | 0.876 | 0.856 | 0.814 | 0.929 | 0.891 | 0.752 | 0.910 | 7 distance |
| M0989 | 128 | exp05 | 1024bit-ECFP4 | KNN | 0.903 | 0.882 | 0.847 | 0.950 | 0.915 | 0.807 | 0.918 | 3 uniform |
| M0990 | 128 | exp06 | 1024bit-ECFP4 | KNN | 0.903 | 0.933 | 0.924 | 0.887 | 0.909 | 0.808 | 0.911 | 3 uniform |
| M0991 | 128 | exp07 | 1024bit-ECFP4 | KNN | 0.907 | 0.898 | 0.873 | 0.936 | 0.917 | 0.813 | 0.904 | 3 uniform |
| M0992 | 128 | exp08 | 1024bit-ECFP4 | KNN | 0.911 | 0.904 | 0.881 | 0.936 | 0.920 | 0.821 | 0.902 | 3 uniform |
| M0993 | 128 | exp09 | 1024bit-ECFP4 | KNN | 0.931 | 0.918 | 0.898 | 0.957 | 0.938 | 0.860 | 0.906 | 3 uniform |
| M0994 | 128 | exp10 | 1024bit-ECFP4 | KNN | 0.880 | 0.853 | 0.805 | 0.943 | 0.896 | 0.762 | 0.910 | 3 uniform |
| M0995 | 128 | exp11 | 1024bit-ECFP4 | KNN | 0.915 | 0.916 | 0.898 | 0.929 | 0.923 | 0.829 | 0.909 | 5 distance |
| M0996 | 128 | exp12 | 1024bit-ECFP4 | KNN | 0.911 | 0.904 | 0.881 | 0.936 | 0.920 | 0.821 | 0.918 | 3 distance |
| M0997 | 128 | exp13 | 1024bit-ECFP4 | KNN | 0.888 | 0.878 | 0.847 | 0.922 | 0.900 | 0.774 | 0.920 | 3 uniform |
| M0998 | 128 | exp14 | 1024bit-ECFP4 | KNN | 0.892 | 0.860 | 0.814 | 0.957 | 0.906 | 0.786 | 0.913 | 3 distance |
| M0999 | 128 | exp15 | 1024bit-ECFP4 | KNN | 0.884 | 0.863 | 0.822 | 0.936 | 0.898 | 0.768 | 0.908 | 7 distance |
| M1000 | 128 | exp16 | 1024bit-ECFP4 | KNN | 0.900 | 0.891 | 0.864 | 0.929 | 0.910 | 0.798 | 0.912 | 1 uniform |
| M1001 | 128 | exp17 | 1024bit-ECFP4 | KNN | 0.896 | 0.913 | 0.898 | 0.894 | 0.903 | 0.790 | 0.920 | 3 uniform |
| M1002 | 128 | exp18 | 1024bit-ECFP4 | KNN | 0.919 | 0.929 | 0.915 | 0.922 | 0.925 | 0.837 | 0.904 | 3 uniform |
| M1003 | 128 | exp19 | 1024bit-ECFP4 | KNN | 0.919 | 0.911 | 0.890 | 0.943 | 0.927 | 0.837 | 0.907 | 1 uniform |
| M1004 | 128 | exp01 | 1024bit-ECFP4 | SVM | 0.896 | 0.901 | 0.881 | 0.908 | 0.905 | 0.790 | 0.917 | 10000.0 0.1 |
| M1005 | 128 | exp02 | 1024bit-ECFP4 | SVM | 0.884 | 0.872 | 0.839 | 0.922 | 0.897 | 0.767 | 0.920 | 10 0.1 |
| M1006 | 128 | exp03 | 1024bit-ECFP4 | SVM | 0.888 | 0.889 | 0.864 | 0.908 | 0.898 | 0.774 | 0.917 | 5 0.1 |
| M1007 | 128 | exp04 | 1024bit-ECFP4 | SVM | 0.892 | 0.890 | 0.864 | 0.915 | 0.902 | 0.782 | 0.917 | 50000.0 0.1 |
| M1008 | 128 | exp05 | 1024bit-ECFP4 | SVM | 0.876 | 0.861 | 0.822 | 0.922 | 0.890 | 0.752 | 0.912 | 50000.0 0.1 |
| M1009 | 128 | exp06 | 1024bit-ECFP4 | SVM | 0.931 | 0.949 | 0.941 | 0.922 | 0.935 | 0.861 | 0.917 | 10 0.1 |
| M1010 | 128 | exp07 | 1024bit-ECFP4 | SVM | 0.915 | 0.916 | 0.898 | 0.929 | 0.923 | 0.829 | 0.910 | 5 0.05 |
| M1011 | 128 | exp08 | 1024bit-ECFP4 | SVM | 0.919 | 0.917 | 0.898 | 0.936 | 0.926 | 0.836 | 0.909 | 1 0.1 |
| M1012 | 128 | exp09 | 1024bit-ECFP4 | SVM | 0.931 | 0.949 | 0.941 | 0.922 | 0.935 | 0.861 | 0.902 | 5 0.1 |
| M1013 | 128 | exp10 | 1024bit-ECFP4 | SVM | 0.915 | 0.899 | 0.873 | 0.950 | 0.924 | 0.829 | 0.912 | 5 0.1 |
| M1014 | 128 | exp11 | 1024bit-ECFP4 | SVM | 0.892 | 0.906 | 0.890 | 0.894 | 0.900 | 0.782 | 0.912 | 5 0.05 |
| M1015 | 128 | exp12 | 1024bit-ECFP4 | SVM | 0.892 | 0.912 | 0.898 | 0.887 | 0.899 | 0.783 | 0.918 | 1 0.1 |
| M1016 | 128 | exp13 | 1024bit-ECFP4 | SVM | 0.900 | 0.897 | 0.873 | 0.922 | 0.909 | 0.797 | 0.927 | 5 0.1 |
| M1017 | 128 | exp14 | 1024bit-ECFP4 | SVM | 0.900 | 0.876 | 0.839 | 0.950 | 0.912 | 0.799 | 0.917 | 500 0.1 |
| M1018 | 128 | exp15 | 1024bit-ECFP4 | SVM | 0.892 | 0.874 | 0.839 | 0.936 | 0.904 | 0.783 | 0.909 | 1 0.1 |
| M1019 | 128 | exp16 | 1024bit-ECFP4 | SVM | 0.923 | 0.929 | 0.915 | 0.929 | 0.929 | 0.844 | 0.916 | 5 0.1 |
| M1020 | 128 | exp17 | 1024bit-ECFP4 | SVM | 0.903 | 0.920 | 0.907 | 0.901 | 0.910 | 0.806 | 0.917 | 5 0.05 |
| M1021 | 128 | exp18 | 1024bit-ECFP4 | SVM | 0.919 | 0.935 | 0.924 | 0.915 | 0.925 | 0.837 | 0.912 | 500 0.1 |
| M1022 | 128 | exp19 | 1024bit-ECFP4 | SVM | 0.934 | 0.937 | 0.924 | 0.943 | 0.940 | 0.868 | 0.913 | 10 0.1 |
| M1023 | 128 | exp01 | 1024bit-ECFP4 | GBM | 0.869 | 0.885 | 0.864 | 0.872 | 0.879 | 0.736 | 0.902 | 200 0.3 |
| M1024 | 128 | exp02 | 1024bit-ECFP4 | GBM | 0.876 | 0.866 | 0.831 | 0.915 | 0.890 | 0.751 | 0.913 | 300 0.3 |
| M1025 | 128 | exp03 | 1024bit-ECFP4 | GBM | 0.884 | 0.872 | 0.839 | 0.922 | 0.897 | 0.767 | 0.904 | 100 0.2 |
| M1026 | 128 | exp04 | 1024bit-ECFP4 | GBM | 0.907 | 0.893 | 0.864 | 0.943 | 0.917 | 0.814 | 0.898 | 300 0.2 |
| M1027 | 128 | exp05 | 1024bit-ECFP4 | GBM | 0.892 | 0.879 | 0.847 | 0.929 | 0.903 | 0.782 | 0.906 | 200 0.2 |
| M1028 | 128 | exp06 | 1024bit-ECFP4 | GBM | 0.927 | 0.936 | 0.924 | 0.929 | 0.932 | 0.852 | 0.906 | 300 0.1 |
| M1029 | 128 | exp07 | 1024bit-ECFP4 | GBM | 0.888 | 0.878 | 0.847 | 0.922 | 0.900 | 0.774 | 0.904 | 500 0.7 |
| M1030 | 128 | exp08 | 1024bit-ECFP4 | GBM | 0.907 | 0.893 | 0.864 | 0.943 | 0.917 | 0.814 | 0.903 | 700 0.1 |
| M1031 | 128 | exp09 | 1024bit-ECFP4 | GBM | 0.903 | 0.903 | 0.881 | 0.922 | 0.912 | 0.805 | 0.897 | 50 0.4 |
| M1032 | 128 | exp10 | 1024bit-ECFP4 | GBM | 0.896 | 0.870 | 0.831 | 0.950 | 0.908 | 0.792 | 0.906 | 100 0.6 |
| M1033 | 128 | exp11 | 1024bit-ECFP4 | GBM | 0.907 | 0.921 | 0.907 | 0.908 | 0.914 | 0.814 | 0.903 | 100 0.3 |
| M1034 | 128 | exp12 | 1024bit-ECFP4 | GBM | 0.880 | 0.887 | 0.864 | 0.894 | 0.890 | 0.759 | 0.899 | 100 0.9 |
| M1035 | 128 | exp13 | 1024bit-ECFP4 | GBM | 0.892 | 0.879 | 0.847 | 0.929 | 0.903 | 0.782 | 0.910 | 300 0.6 |
| M1036 | 128 | exp14 | 1024bit-ECFP4 | GBM | 0.892 | 0.874 | 0.839 | 0.936 | 0.904 | 0.783 | 0.911 | 300 0.7 |
| M1037 | 128 | exp15 | 1024bit-ECFP4 | GBM | 0.873 | 0.865 | 0.831 | 0.908 | 0.886 | 0.743 | 0.905 | 50 0.4 |
| M1038 | 128 | exp16 | 1024bit-ECFP4 | GBM | 0.903 | 0.887 | 0.856 | 0.943 | 0.914 | 0.806 | 0.914 | 800 0.1 |
| M1039 | 128 | exp17 | 1024bit-ECFP4 | GBM | 0.915 | 0.910 | 0.890 | 0.936 | 0.923 | 0.829 | 0.903 | 1000 0.1 |
| M1040 | 128 | exp18 | 1024bit-ECFP4 | GBM | 0.900 | 0.926 | 0.915 | 0.887 | 0.906 | 0.799 | 0.899 | 50 0.6 |
| M1041 | 128 | exp19 | 1024bit-ECFP4 | GBM | 0.919 | 0.935 | 0.924 | 0.915 | 0.925 | 0.837 | 0.903 | 300 0.2 |
| M1042 | 128 | exp01 | 1024bit-ECFP4 | RF | 0.888 | 0.878 | 0.847 | 0.922 | 0.900 | 0.774 | 0.921 | 900 |
| M1043 | 128 | exp02 | 1024bit-ECFP4 | RF | 0.873 | 0.846 | 0.797 | 0.936 | 0.889 | 0.746 | 0.919 | 900 |
| M1044 | 128 | exp03 | 1024bit-ECFP4 | RF | 0.907 | 0.873 | 0.831 | 0.972 | 0.919 | 0.818 | 0.918 | 700 |
| M1045 | 128 | exp04 | 1024bit-ECFP4 | RF | 0.900 | 0.891 | 0.864 | 0.929 | 0.910 | 0.798 | 0.920 | 800 |
| M1046 | 128 | exp05 | 1024bit-ECFP4 | RF | 0.892 | 0.865 | 0.822 | 0.950 | 0.905 | 0.785 | 0.916 | 1000 |
| M1047 | 128 | exp06 | 1024bit-ECFP4 | RF | 0.927 | 0.942 | 0.932 | 0.922 | 0.932 | 0.853 | 0.916 | 600 |
| M1048 | 128 | exp07 | 1024bit-ECFP4 | RF | 0.915 | 0.889 | 0.856 | 0.965 | 0.925 | 0.831 | 0.916 | 200 |
| M1049 | 128 | exp08 | 1024bit-ECFP4 | RF | 0.923 | 0.912 | 0.890 | 0.950 | 0.931 | 0.845 | 0.910 | 1000 |
| M1050 | 128 | exp09 | 1024bit-ECFP4 | RF | 0.950 | 0.938 | 0.924 | 0.972 | 0.955 | 0.899 | 0.907 | 200 |
| M1051 | 128 | exp10 | 1024bit-ECFP4 | RF | 0.907 | 0.882 | 0.847 | 0.957 | 0.918 | 0.815 | 0.913 | 100 |
| M1052 | 128 | exp11 | 1024bit-ECFP4 | RF | 0.927 | 0.907 | 0.881 | 0.965 | 0.935 | 0.853 | 0.910 | 200 |
| M1053 | 128 | exp12 | 1024bit-ECFP4 | RF | 0.907 | 0.898 | 0.873 | 0.936 | 0.917 | 0.813 | 0.919 | 900 |
| M1054 | 128 | exp13 | 1024bit-ECFP4 | RF | 0.892 | 0.879 | 0.847 | 0.929 | 0.903 | 0.782 | 0.921 | 100 |
| M1055 | 128 | exp14 | 1024bit-ECFP4 | RF | 0.892 | 0.855 | 0.805 | 0.965 | 0.907 | 0.787 | 0.925 | 500 |
| M1056 | 128 | exp15 | 1024bit-ECFP4 | RF | 0.884 | 0.858 | 0.814 | 0.943 | 0.899 | 0.769 | 0.909 | 500 |
| M1057 | 128 | exp16 | 1024bit-ECFP4 | RF | 0.923 | 0.912 | 0.890 | 0.950 | 0.931 | 0.845 | 0.916 | 800 |
| M1058 | 128 | exp17 | 1024bit-ECFP4 | RF | 0.903 | 0.908 | 0.890 | 0.915 | 0.912 | 0.805 | 0.922 | 300 |
| M1059 | 128 | exp18 | 1024bit-ECFP4 | RF | 0.911 | 0.921 | 0.907 | 0.915 | 0.918 | 0.821 | 0.916 | 1000 |
| M1060 | 128 | exp19 | 1024bit-ECFP4 | RF | 0.923 | 0.901 | 0.873 | 0.965 | 0.932 | 0.846 | 0.917 | 500 |
| M1061 | 128 | exp01 | 1024bit-ECFP4 | DNN2 | 0.869 | 0.891 | 0.873 | 0.865 | 0.878 | 0.736 | 0.913 | 600 80 0.4 |
| M1062 | 128 | exp02 | 1024bit-ECFP4 | DNN2 | 0.853 | 0.860 | 0.831 | 0.872 | 0.866 | 0.704 | 0.907 | 400 100 0.2 |
| M1063 | 128 | exp03 | 1024bit-ECFP4 | DNN2 | 0.873 | 0.886 | 0.864 | 0.879 | 0.883 | 0.743 | 0.912 | 500 120 0.2 |
| M1064 | 128 | exp01 | 1024bit-ECFP4 | DNN3 | 0.869 | 0.879 | 0.856 | 0.879 | 0.879 | 0.735 | 0.912 | 300 100 0.5 |
| M1065 | 128 | exp02 | 1024bit-ECFP4 | DNN3 | 0.865 | 0.879 | 0.856 | 0.872 | 0.875 | 0.728 | 0.905 | 400 80 0.3 |
| M1066 | 128 | exp03 | 1024bit-ECFP4 | DNN3 | 0.911 | 0.910 | 0.890 | 0.929 | 0.919 | 0.821 | 0.910 | 500 60 0.3 |
| M1067 | 128 | exp01 | 2048bit-ECFP4 | KNN | 0.884 | 0.863 | 0.822 | 0.936 | 0.898 | 0.768 | 0.924 | 5 uniform |
| M1068 | 128 | exp02 | 2048bit-ECFP4 | KNN | 0.842 | 0.829 | 0.780 | 0.894 | 0.860 | 0.681 | 0.921 | 5 uniform |
| M1069 | 128 | exp03 | 2048bit-ECFP4 | KNN | 0.880 | 0.853 | 0.805 | 0.943 | 0.896 | 0.762 | 0.917 | 7 distance |
| M1070 | 128 | exp04 | 2048bit-ECFP4 | KNN | 0.896 | 0.875 | 0.839 | 0.943 | 0.908 | 0.791 | 0.914 | 5 distance |
| M1071 | 128 | exp05 | 2048bit-ECFP4 | KNN | 0.907 | 0.873 | 0.831 | 0.972 | 0.919 | 0.818 | 0.907 | 3 uniform |
| M1072 | 128 | exp06 | 2048bit-ECFP4 | KNN | 0.896 | 0.919 | 0.907 | 0.887 | 0.903 | 0.791 | 0.913 | 3 distance |
| M1073 | 128 | exp07 | 2048bit-ECFP4 | KNN | 0.915 | 0.894 | 0.864 | 0.957 | 0.925 | 0.830 | 0.905 | 3 uniform |
| M1074 | 128 | exp08 | 2048bit-ECFP4 | KNN | 0.915 | 0.899 | 0.873 | 0.950 | 0.924 | 0.829 | 0.907 | 3 distance |
| M1075 | 128 | exp09 | 2048bit-ECFP4 | KNN | 0.931 | 0.907 | 0.881 | 0.972 | 0.938 | 0.862 | 0.908 | 11 distance |
| M1076 | 128 | exp10 | 2048bit-ECFP4 | KNN | 0.888 | 0.868 | 0.831 | 0.936 | 0.901 | 0.775 | 0.912 | 3 uniform |
| M1077 | 128 | exp11 | 2048bit-ECFP4 | KNN | 0.907 | 0.903 | 0.881 | 0.929 | 0.916 | 0.813 | 0.910 | 3 distance |
| M1078 | 128 | exp12 | 2048bit-ECFP4 | KNN | 0.900 | 0.886 | 0.856 | 0.936 | 0.910 | 0.798 | 0.913 | 7 distance |
| M1079 | 128 | exp13 | 2048bit-ECFP4 | KNN | 0.900 | 0.886 | 0.856 | 0.936 | 0.910 | 0.798 | 0.912 | 5 uniform |
| M1080 | 128 | exp14 | 2048bit-ECFP4 | KNN | 0.896 | 0.865 | 0.822 | 0.957 | 0.909 | 0.793 | 0.916 | 5 distance |
| M1081 | 128 | exp15 | 2048bit-ECFP4 | KNN | 0.880 | 0.867 | 0.831 | 0.922 | 0.893 | 0.759 | 0.914 | 3 uniform |
| M1082 | 128 | exp16 | 2048bit-ECFP4 | KNN | 0.900 | 0.886 | 0.856 | 0.936 | 0.910 | 0.798 | 0.914 | 3 distance |
| M1083 | 128 | exp17 | 2048bit-ECFP4 | KNN | 0.896 | 0.907 | 0.890 | 0.901 | 0.904 | 0.790 | 0.917 | 3 uniform |
| M1084 | 128 | exp18 | 2048bit-ECFP4 | KNN | 0.927 | 0.936 | 0.924 | 0.929 | 0.932 | 0.852 | 0.905 | 3 uniform |
| M1085 | 128 | exp19 | 2048bit-ECFP4 | KNN | 0.919 | 0.895 | 0.864 | 0.965 | 0.928 | 0.838 | 0.911 | 7 distance |
| M1086 | 128 | exp01 | 2048bit-ECFP4 | SVM | 0.884 | 0.883 | 0.856 | 0.908 | 0.895 | 0.766 | 0.918 | 5 0.05 |
| M1087 | 128 | exp02 | 2048bit-ECFP4 | SVM | 0.873 | 0.865 | 0.831 | 0.908 | 0.886 | 0.743 | 0.921 | 1 0.1 |
| M1088 | 128 | exp03 | 2048bit-ECFP4 | SVM | 0.888 | 0.873 | 0.839 | 0.929 | 0.900 | 0.775 | 0.912 | 5 0.05 |
| M1089 | 128 | exp04 | 2048bit-ECFP4 | SVM | 0.884 | 0.888 | 0.864 | 0.901 | 0.894 | 0.766 | 0.913 | 5 0.1 |
| M1090 | 128 | exp05 | 2048bit-ECFP4 | SVM | 0.903 | 0.887 | 0.856 | 0.943 | 0.914 | 0.806 | 0.915 | 1 0.1 |
| M1091 | 128 | exp06 | 2048bit-ECFP4 | SVM | 0.896 | 0.890 | 0.864 | 0.922 | 0.906 | 0.790 | 0.910 | 1 0.05 |
| M1092 | 128 | exp07 | 2048bit-ECFP4 | SVM | 0.892 | 0.874 | 0.839 | 0.936 | 0.904 | 0.783 | 0.909 | 10 0.01 |
| M1093 | 128 | exp08 | 2048bit-ECFP4 | SVM | 0.919 | 0.917 | 0.898 | 0.936 | 0.926 | 0.836 | 0.909 | 1 0.1 |
| M1094 | 128 | exp09 | 2048bit-ECFP4 | SVM | 0.903 | 0.920 | 0.907 | 0.901 | 0.910 | 0.806 | 0.906 | 5 0.05 |
| M1095 | 128 | exp10 | 2048bit-ECFP4 | SVM | 0.900 | 0.886 | 0.856 | 0.936 | 0.910 | 0.798 | 0.910 | 5 0.1 |
| M1096 | 128 | exp11 | 2048bit-ECFP4 | SVM | 0.907 | 0.927 | 0.915 | 0.901 | 0.914 | 0.814 | 0.910 | 5 0.05 |
| M1097 | 128 | exp12 | 2048bit-ECFP4 | SVM | 0.900 | 0.908 | 0.890 | 0.908 | 0.908 | 0.798 | 0.920 | 10 0.05 |
| M1098 | 128 | exp13 | 2048bit-ECFP4 | SVM | 0.903 | 0.897 | 0.873 | 0.929 | 0.913 | 0.805 | 0.922 | 5 0.05 |
| M1099 | 128 | exp14 | 2048bit-ECFP4 | SVM | 0.911 | 0.883 | 0.847 | 0.965 | 0.922 | 0.824 | 0.919 | 1 0.1 |
| M1100 | 128 | exp15 | 2048bit-ECFP4 | SVM | 0.896 | 0.880 | 0.847 | 0.936 | 0.907 | 0.790 | 0.909 | 5 0.05 |
| M1101 | 128 | exp16 | 2048bit-ECFP4 | SVM | 0.915 | 0.905 | 0.881 | 0.943 | 0.924 | 0.829 | 0.923 | 5 0.05 |
| M1102 | 128 | exp17 | 2048bit-ECFP4 | SVM | 0.911 | 0.928 | 0.915 | 0.908 | 0.918 | 0.822 | 0.918 | 5 0.05 |
| M1103 | 128 | exp18 | 2048bit-ECFP4 | SVM | 0.911 | 0.934 | 0.924 | 0.901 | 0.917 | 0.822 | 0.910 | 1 0.1 |
| M1104 | 128 | exp19 | 2048bit-ECFP4 | SVM | 0.946 | 0.944 | 0.932 | 0.957 | 0.951 | 0.891 | 0.913 | 5 0.05 |
| M1105 | 128 | exp01 | 2048bit-ECFP4 | GBM | 0.873 | 0.875 | 0.847 | 0.894 | 0.884 | 0.743 | 0.902 | 200 0.7 |
| M1106 | 128 | exp02 | 2048bit-ECFP4 | GBM | 0.857 | 0.842 | 0.797 | 0.908 | 0.874 | 0.712 | 0.910 | 400 0.2 |
| M1107 | 128 | exp03 | 2048bit-ECFP4 | GBM | 0.892 | 0.879 | 0.847 | 0.929 | 0.903 | 0.782 | 0.918 | 300 0.1 |
| M1108 | 128 | exp04 | 2048bit-ECFP4 | GBM | 0.903 | 0.903 | 0.881 | 0.922 | 0.912 | 0.805 | 0.905 | 600 0.1 |
| M1109 | 128 | exp05 | 2048bit-ECFP4 | GBM | 0.869 | 0.850 | 0.805 | 0.922 | 0.884 | 0.736 | 0.911 | 200 0.5 |
| M1110 | 128 | exp06 | 2048bit-ECFP4 | GBM | 0.903 | 0.908 | 0.890 | 0.915 | 0.912 | 0.805 | 0.902 | 100 0.2 |
| M1111 | 128 | exp07 | 2048bit-ECFP4 | GBM | 0.892 | 0.879 | 0.847 | 0.929 | 0.903 | 0.782 | 0.905 | 100 0.2 |
| M1112 | 128 | exp08 | 2048bit-ECFP4 | GBM | 0.915 | 0.899 | 0.873 | 0.950 | 0.924 | 0.829 | 0.900 | 50 0.4 |
| M1113 | 128 | exp09 | 2048bit-ECFP4 | GBM | 0.915 | 0.922 | 0.907 | 0.922 | 0.922 | 0.829 | 0.895 | 200 0.1 |
| M1114 | 128 | exp10 | 2048bit-ECFP4 | GBM | 0.892 | 0.874 | 0.839 | 0.936 | 0.904 | 0.783 | 0.905 | 500 0.3 |
| M1115 | 128 | exp11 | 2048bit-ECFP4 | GBM | 0.896 | 0.896 | 0.873 | 0.915 | 0.905 | 0.790 | 0.910 | 600 0.1 |
| M1116 | 128 | exp12 | 2048bit-ECFP4 | GBM | 0.903 | 0.908 | 0.890 | 0.915 | 0.912 | 0.805 | 0.907 | 400 0.4 |
| M1117 | 128 | exp13 | 2048bit-ECFP4 | GBM | 0.869 | 0.841 | 0.788 | 0.936 | 0.886 | 0.738 | 0.911 | 100 0.2 |
| M1118 | 128 | exp14 | 2048bit-ECFP4 | GBM | 0.892 | 0.884 | 0.856 | 0.922 | 0.903 | 0.782 | 0.903 | 300 0.4 |
| M1119 | 128 | exp15 | 2048bit-ECFP4 | GBM | 0.896 | 0.865 | 0.822 | 0.957 | 0.909 | 0.793 | 0.911 | 200 0.2 |
| M1120 | 128 | exp16 | 2048bit-ECFP4 | GBM | 0.900 | 0.897 | 0.873 | 0.922 | 0.909 | 0.797 | 0.917 | 50 0.6 |
| M1121 | 128 | exp17 | 2048bit-ECFP4 | GBM | 0.896 | 0.901 | 0.881 | 0.908 | 0.905 | 0.790 | 0.909 | 200 0.7 |
| M1122 | 128 | exp18 | 2048bit-ECFP4 | GBM | 0.931 | 0.949 | 0.941 | 0.922 | 0.935 | 0.861 | 0.902 | 100 0.4 |
| M1123 | 128 | exp19 | 2048bit-ECFP4 | GBM | 0.927 | 0.907 | 0.881 | 0.965 | 0.935 | 0.853 | 0.900 | 100 0.4 |
| M1124 | 128 | exp01 | 2048bit-ECFP4 | RF | 0.876 | 0.861 | 0.822 | 0.922 | 0.890 | 0.752 | 0.924 | 200 |
| M1125 | 128 | exp02 | 2048bit-ECFP4 | RF | 0.861 | 0.834 | 0.780 | 0.929 | 0.879 | 0.722 | 0.915 | 900 |
| M1126 | 128 | exp03 | 2048bit-ECFP4 | RF | 0.896 | 0.865 | 0.822 | 0.957 | 0.909 | 0.793 | 0.924 | 200 |
| M1127 | 128 | exp04 | 2048bit-ECFP4 | RF | 0.896 | 0.875 | 0.839 | 0.943 | 0.908 | 0.791 | 0.913 | 800 |
| M1128 | 128 | exp05 | 2048bit-ECFP4 | RF | 0.884 | 0.863 | 0.822 | 0.936 | 0.898 | 0.768 | 0.910 | 700 |
| M1129 | 128 | exp06 | 2048bit-ECFP4 | RF | 0.919 | 0.941 | 0.932 | 0.908 | 0.924 | 0.838 | 0.918 | 300 |
| M1130 | 128 | exp07 | 2048bit-ECFP4 | RF | 0.907 | 0.887 | 0.856 | 0.950 | 0.918 | 0.814 | 0.909 | 200 |
| M1131 | 128 | exp08 | 2048bit-ECFP4 | RF | 0.915 | 0.899 | 0.873 | 0.950 | 0.924 | 0.829 | 0.908 | 400 |
| M1132 | 128 | exp09 | 2048bit-ECFP4 | RF | 0.938 | 0.931 | 0.915 | 0.957 | 0.944 | 0.876 | 0.903 | 200 |
| M1133 | 128 | exp10 | 2048bit-ECFP4 | RF | 0.896 | 0.865 | 0.822 | 0.957 | 0.909 | 0.793 | 0.913 | 100 |
| M1134 | 128 | exp11 | 2048bit-ECFP4 | RF | 0.923 | 0.912 | 0.890 | 0.950 | 0.931 | 0.845 | 0.909 | 100 |
| M1135 | 128 | exp12 | 2048bit-ECFP4 | RF | 0.900 | 0.881 | 0.847 | 0.943 | 0.911 | 0.799 | 0.918 | 200 |
| M1136 | 128 | exp13 | 2048bit-ECFP4 | RF | 0.903 | 0.887 | 0.856 | 0.943 | 0.914 | 0.806 | 0.917 | 50 |
| M1137 | 128 | exp14 | 2048bit-ECFP4 | RF | 0.892 | 0.855 | 0.805 | 0.965 | 0.907 | 0.787 | 0.923 | 50 |
| M1138 | 128 | exp15 | 2048bit-ECFP4 | RF | 0.900 | 0.876 | 0.839 | 0.950 | 0.912 | 0.799 | 0.908 | 500 |
| M1139 | 128 | exp16 | 2048bit-ECFP4 | RF | 0.911 | 0.899 | 0.873 | 0.943 | 0.920 | 0.821 | 0.920 | 700 |
| M1140 | 128 | exp17 | 2048bit-ECFP4 | RF | 0.907 | 0.915 | 0.898 | 0.915 | 0.915 | 0.813 | 0.922 | 900 |
| M1141 | 128 | exp18 | 2048bit-ECFP4 | RF | 0.919 | 0.941 | 0.932 | 0.908 | 0.924 | 0.838 | 0.911 | 300 |
| M1142 | 128 | exp19 | 2048bit-ECFP4 | RF | 0.915 | 0.889 | 0.856 | 0.965 | 0.925 | 0.831 | 0.917 | 400 |
| M1143 | 128 | exp01 | 2048bit-ECFP4 | DNN2 | 0.892 | 0.884 | 0.856 | 0.922 | 0.903 | 0.782 | 0.915 | 600 80 0.3 |
| M1144 | 128 | exp02 | 2048bit-ECFP4 | DNN2 | 0.853 | 0.860 | 0.831 | 0.872 | 0.866 | 0.704 | 0.912 | 500 80 0.1 |
| M1145 | 128 | exp03 | 2048bit-ECFP4 | DNN2 | 0.896 | 0.890 | 0.864 | 0.922 | 0.906 | 0.790 | 0.918 | 600 80 0.3 |
| M1146 | 128 | exp01 | 2048bit-ECFP4 | DNN3 | 0.892 | 0.890 | 0.864 | 0.915 | 0.902 | 0.782 | 0.914 | 500 100 0.2 |
| M1147 | 128 | exp02 | 2048bit-ECFP4 | DNN3 | 0.846 | 0.853 | 0.822 | 0.865 | 0.859 | 0.688 | 0.912 | 600 140 0.3 |
| M1148 | 128 | exp03 | 2048bit-ECFP4 | DNN3 | 0.884 | 0.878 | 0.847 | 0.915 | 0.896 | 0.766 | 0.923 | 100 120 0.5 |
| M1149 | 128 | exp01 | 1024bit-ECFP6 | KNN | 0.880 | 0.867 | 0.831 | 0.922 | 0.893 | 0.759 | 0.913 | 5 distance |
| M1150 | 128 | exp02 | 1024bit-ECFP6 | KNN | 0.865 | 0.863 | 0.831 | 0.894 | 0.878 | 0.727 | 0.914 | 3 uniform |
| M1151 | 128 | exp03 | 1024bit-ECFP6 | KNN | 0.884 | 0.868 | 0.831 | 0.929 | 0.897 | 0.767 | 0.911 | 5 distance |
| M1152 | 128 | exp04 | 1024bit-ECFP6 | KNN | 0.873 | 0.855 | 0.814 | 0.922 | 0.887 | 0.744 | 0.905 | 9 distance |
| M1153 | 128 | exp05 | 1024bit-ECFP6 | KNN | 0.907 | 0.887 | 0.856 | 0.950 | 0.918 | 0.814 | 0.906 | 3 uniform |
| M1154 | 128 | exp06 | 1024bit-ECFP6 | KNN | 0.903 | 0.926 | 0.915 | 0.894 | 0.910 | 0.807 | 0.907 | 5 uniform |
| M1155 | 128 | exp07 | 1024bit-ECFP6 | KNN | 0.915 | 0.894 | 0.864 | 0.957 | 0.925 | 0.830 | 0.897 | 7 uniform |
| M1156 | 128 | exp08 | 1024bit-ECFP6 | KNN | 0.900 | 0.891 | 0.864 | 0.929 | 0.910 | 0.798 | 0.905 | 7 distance |
| M1157 | 128 | exp09 | 1024bit-ECFP6 | KNN | 0.911 | 0.888 | 0.856 | 0.957 | 0.922 | 0.823 | 0.903 | 7 uniform |
| M1158 | 128 | exp10 | 1024bit-ECFP6 | KNN | 0.880 | 0.857 | 0.814 | 0.936 | 0.895 | 0.761 | 0.899 | 3 uniform |
| M1159 | 128 | exp11 | 1024bit-ECFP6 | KNN | 0.892 | 0.912 | 0.898 | 0.887 | 0.899 | 0.783 | 0.905 | 1 uniform |
| M1160 | 128 | exp12 | 1024bit-ECFP6 | KNN | 0.900 | 0.902 | 0.881 | 0.915 | 0.908 | 0.797 | 0.907 | 5 distance |
| M1161 | 128 | exp13 | 1024bit-ECFP6 | KNN | 0.896 | 0.885 | 0.856 | 0.929 | 0.907 | 0.790 | 0.912 | 5 distance |
| M1162 | 128 | exp14 | 1024bit-ECFP6 | KNN | 0.903 | 0.877 | 0.839 | 0.957 | 0.915 | 0.808 | 0.912 | 3 uniform |
| M1163 | 128 | exp15 | 1024bit-ECFP6 | KNN | 0.880 | 0.862 | 0.822 | 0.929 | 0.894 | 0.760 | 0.905 | 5 distance |
| M1164 | 128 | exp16 | 1024bit-ECFP6 | KNN | 0.911 | 0.915 | 0.898 | 0.922 | 0.919 | 0.821 | 0.908 | 3 uniform |
| M1165 | 128 | exp17 | 1024bit-ECFP6 | KNN | 0.911 | 0.915 | 0.898 | 0.922 | 0.919 | 0.821 | 0.909 | 3 distance |
| M1166 | 128 | exp18 | 1024bit-ECFP6 | KNN | 0.911 | 0.910 | 0.890 | 0.929 | 0.919 | 0.821 | 0.895 | 7 distance |
| M1167 | 128 | exp19 | 1024bit-ECFP6 | KNN | 0.911 | 0.910 | 0.890 | 0.929 | 0.919 | 0.821 | 0.905 | 1 uniform |
| M1168 | 128 | exp01 | 1024bit-ECFP6 | SVM | 0.873 | 0.860 | 0.822 | 0.915 | 0.887 | 0.743 | 0.916 | 1 0.1 |
| M1169 | 128 | exp02 | 1024bit-ECFP6 | SVM | 0.865 | 0.844 | 0.797 | 0.922 | 0.881 | 0.729 | 0.914 | 1 0.1 |
| M1170 | 128 | exp03 | 1024bit-ECFP6 | SVM | 0.888 | 0.878 | 0.847 | 0.922 | 0.900 | 0.774 | 0.923 | 50000.0 0.1 |
| M1171 | 128 | exp04 | 1024bit-ECFP6 | SVM | 0.903 | 0.882 | 0.847 | 0.950 | 0.915 | 0.807 | 0.909 | 5 0.1 |
| M1172 | 128 | exp05 | 1024bit-ECFP6 | SVM | 0.896 | 0.875 | 0.839 | 0.943 | 0.908 | 0.791 | 0.908 | 500 0.1 |
| M1173 | 128 | exp06 | 1024bit-ECFP6 | SVM | 0.934 | 0.949 | 0.941 | 0.929 | 0.939 | 0.868 | 0.910 | 5 0.1 |
| M1174 | 128 | exp07 | 1024bit-ECFP6 | SVM | 0.915 | 0.894 | 0.864 | 0.957 | 0.925 | 0.830 | 0.904 | 1 0.1 |
| M1175 | 128 | exp08 | 1024bit-ECFP6 | SVM | 0.915 | 0.916 | 0.898 | 0.929 | 0.923 | 0.829 | 0.908 | 5 0.1 |
| M1176 | 128 | exp09 | 1024bit-ECFP6 | SVM | 0.938 | 0.943 | 0.932 | 0.943 | 0.943 | 0.875 | 0.900 | 5 0.1 |
| M1177 | 128 | exp10 | 1024bit-ECFP6 | SVM | 0.911 | 0.888 | 0.856 | 0.957 | 0.922 | 0.823 | 0.914 | 5 0.1 |
| M1178 | 128 | exp11 | 1024bit-ECFP6 | SVM | 0.903 | 0.897 | 0.873 | 0.929 | 0.913 | 0.805 | 0.915 | 10 0.1 |
| M1179 | 128 | exp12 | 1024bit-ECFP6 | SVM | 0.892 | 0.912 | 0.898 | 0.887 | 0.899 | 0.783 | 0.918 | 5 0.1 |
| M1180 | 128 | exp13 | 1024bit-ECFP6 | SVM | 0.900 | 0.881 | 0.847 | 0.943 | 0.911 | 0.799 | 0.918 | 1 0.1 |
| M1181 | 128 | exp14 | 1024bit-ECFP6 | SVM | 0.892 | 0.869 | 0.831 | 0.943 | 0.905 | 0.784 | 0.915 | 5 0.1 |
| M1182 | 128 | exp15 | 1024bit-ECFP6 | SVM | 0.888 | 0.854 | 0.805 | 0.957 | 0.903 | 0.779 | 0.905 | 10 0.1 |
| M1183 | 128 | exp16 | 1024bit-ECFP6 | SVM | 0.923 | 0.912 | 0.890 | 0.950 | 0.931 | 0.845 | 0.916 | 5 0.1 |
| M1184 | 128 | exp17 | 1024bit-ECFP6 | SVM | 0.907 | 0.921 | 0.907 | 0.908 | 0.914 | 0.814 | 0.909 | 100 0.1 |
| M1185 | 128 | exp18 | 1024bit-ECFP6 | SVM | 0.923 | 0.935 | 0.924 | 0.922 | 0.929 | 0.845 | 0.901 | 5000.0 0.1 |
| M1186 | 128 | exp19 | 1024bit-ECFP6 | SVM | 0.915 | 0.905 | 0.881 | 0.943 | 0.924 | 0.829 | 0.913 | 1 0.1 |
| M1187 | 128 | exp01 | 1024bit-ECFP6 | GBM | 0.873 | 0.870 | 0.839 | 0.901 | 0.885 | 0.743 | 0.901 | 700 0.1 |
| M1188 | 128 | exp02 | 1024bit-ECFP6 | GBM | 0.830 | 0.839 | 0.805 | 0.851 | 0.845 | 0.657 | 0.899 | 800 0.4 |
| M1189 | 128 | exp03 | 1024bit-ECFP6 | GBM | 0.896 | 0.875 | 0.839 | 0.943 | 0.908 | 0.791 | 0.912 | 500 0.2 |
| M1190 | 128 | exp04 | 1024bit-ECFP6 | GBM | 0.880 | 0.853 | 0.805 | 0.943 | 0.896 | 0.762 | 0.895 | 400 0.2 |
| M1191 | 128 | exp05 | 1024bit-ECFP6 | GBM | 0.884 | 0.872 | 0.839 | 0.922 | 0.897 | 0.767 | 0.902 | 300 0.3 |
| M1192 | 128 | exp06 | 1024bit-ECFP6 | GBM | 0.884 | 0.894 | 0.873 | 0.894 | 0.894 | 0.766 | 0.896 | 100 0.2 |
| M1193 | 128 | exp07 | 1024bit-ECFP6 | GBM | 0.884 | 0.868 | 0.831 | 0.929 | 0.897 | 0.767 | 0.894 | 100 0.2 |
| M1194 | 128 | exp08 | 1024bit-ECFP6 | GBM | 0.896 | 0.907 | 0.890 | 0.901 | 0.904 | 0.790 | 0.904 | 300 0.3 |
| M1195 | 128 | exp09 | 1024bit-ECFP6 | GBM | 0.911 | 0.934 | 0.924 | 0.901 | 0.917 | 0.822 | 0.893 | 400 0.4 |
| M1196 | 128 | exp10 | 1024bit-ECFP6 | GBM | 0.911 | 0.888 | 0.856 | 0.957 | 0.922 | 0.823 | 0.902 | 100 0.4 |
| M1197 | 128 | exp11 | 1024bit-ECFP6 | GBM | 0.896 | 0.885 | 0.856 | 0.929 | 0.907 | 0.790 | 0.902 | 200 0.7 |
| M1198 | 128 | exp12 | 1024bit-ECFP6 | GBM | 0.880 | 0.882 | 0.856 | 0.901 | 0.891 | 0.758 | 0.908 | 200 0.1 |
| M1199 | 128 | exp13 | 1024bit-ECFP6 | GBM | 0.892 | 0.890 | 0.864 | 0.915 | 0.902 | 0.782 | 0.904 | 300 0.4 |
| M1200 | 128 | exp14 | 1024bit-ECFP6 | GBM | 0.888 | 0.873 | 0.839 | 0.929 | 0.900 | 0.775 | 0.900 | 200 0.2 |
| M1201 | 128 | exp15 | 1024bit-ECFP6 | GBM | 0.857 | 0.847 | 0.805 | 0.901 | 0.873 | 0.712 | 0.894 | 900 0.2 |
| M1202 | 128 | exp16 | 1024bit-ECFP6 | GBM | 0.896 | 0.890 | 0.864 | 0.922 | 0.906 | 0.790 | 0.901 | 300 0.2 |
| M1203 | 128 | exp17 | 1024bit-ECFP6 | GBM | 0.884 | 0.888 | 0.864 | 0.901 | 0.894 | 0.766 | 0.896 | 400 0.7 |
| M1204 | 128 | exp18 | 1024bit-ECFP6 | GBM | 0.900 | 0.914 | 0.898 | 0.901 | 0.907 | 0.798 | 0.891 | 100 0.4 |
| M1205 | 128 | exp19 | 1024bit-ECFP6 | GBM | 0.915 | 0.910 | 0.890 | 0.936 | 0.923 | 0.829 | 0.899 | 200 0.7 |
| M1206 | 128 | exp01 | 1024bit-ECFP6 | RF | 0.884 | 0.868 | 0.831 | 0.929 | 0.897 | 0.767 | 0.920 | 50 |
| M1207 | 128 | exp02 | 1024bit-ECFP6 | RF | 0.857 | 0.821 | 0.754 | 0.943 | 0.878 | 0.718 | 0.914 | 100 |
| M1208 | 128 | exp03 | 1024bit-ECFP6 | RF | 0.880 | 0.848 | 0.797 | 0.950 | 0.896 | 0.763 | 0.922 | 900 |
| M1209 | 128 | exp04 | 1024bit-ECFP6 | RF | 0.892 | 0.865 | 0.822 | 0.950 | 0.905 | 0.785 | 0.916 | 400 |
| M1210 | 128 | exp05 | 1024bit-ECFP6 | RF | 0.900 | 0.857 | 0.805 | 0.979 | 0.914 | 0.805 | 0.917 | 50 |
| M1211 | 128 | exp06 | 1024bit-ECFP6 | RF | 0.927 | 0.936 | 0.924 | 0.929 | 0.932 | 0.852 | 0.915 | 500 |
| M1212 | 128 | exp07 | 1024bit-ECFP6 | RF | 0.923 | 0.890 | 0.856 | 0.979 | 0.932 | 0.848 | 0.910 | 300 |
| M1213 | 128 | exp08 | 1024bit-ECFP6 | RF | 0.919 | 0.900 | 0.873 | 0.957 | 0.928 | 0.838 | 0.913 | 100 |
| M1214 | 128 | exp09 | 1024bit-ECFP6 | RF | 0.927 | 0.918 | 0.898 | 0.950 | 0.934 | 0.852 | 0.904 | 100 |
| M1215 | 128 | exp10 | 1024bit-ECFP6 | RF | 0.903 | 0.867 | 0.822 | 0.972 | 0.916 | 0.810 | 0.916 | 700 |
| M1216 | 128 | exp11 | 1024bit-ECFP6 | RF | 0.903 | 0.877 | 0.839 | 0.957 | 0.915 | 0.808 | 0.911 | 300 |
| M1217 | 128 | exp12 | 1024bit-ECFP6 | RF | 0.903 | 0.908 | 0.890 | 0.915 | 0.912 | 0.805 | 0.922 | 200 |
| M1218 | 128 | exp13 | 1024bit-ECFP6 | RF | 0.896 | 0.885 | 0.856 | 0.929 | 0.907 | 0.790 | 0.915 | 300 |
| M1219 | 128 | exp14 | 1024bit-ECFP6 | RF | 0.896 | 0.861 | 0.814 | 0.965 | 0.910 | 0.795 | 0.918 | 900 |
| M1220 | 128 | exp15 | 1024bit-ECFP6 | RF | 0.896 | 0.861 | 0.814 | 0.965 | 0.910 | 0.795 | 0.907 | 800 |
| M1221 | 128 | exp16 | 1024bit-ECFP6 | RF | 0.903 | 0.882 | 0.847 | 0.950 | 0.915 | 0.807 | 0.911 | 400 |
| M1222 | 128 | exp17 | 1024bit-ECFP6 | RF | 0.892 | 0.895 | 0.873 | 0.908 | 0.901 | 0.782 | 0.913 | 1000 |
| M1223 | 128 | exp18 | 1024bit-ECFP6 | RF | 0.934 | 0.943 | 0.932 | 0.936 | 0.940 | 0.868 | 0.901 | 900 |
| M1224 | 128 | exp19 | 1024bit-ECFP6 | RF | 0.931 | 0.902 | 0.873 | 0.979 | 0.939 | 0.863 | 0.917 | 700 |
| M1225 | 128 | exp01 | 1024bit-ECFP6 | DNN2 | 0.888 | 0.884 | 0.856 | 0.915 | 0.899 | 0.774 | 0.902 | 100 60 0.2 |
| M1226 | 128 | exp02 | 1024bit-ECFP6 | DNN2 | 0.853 | 0.855 | 0.822 | 0.879 | 0.867 | 0.704 | 0.896 | 600 100 0.3 |
| M1227 | 128 | exp03 | 1024bit-ECFP6 | DNN2 | 0.853 | 0.841 | 0.797 | 0.901 | 0.870 | 0.704 | 0.911 | 400 120 0.3 |
| M1228 | 128 | exp01 | 1024bit-ECFP6 | DNN3 | 0.876 | 0.887 | 0.864 | 0.887 | 0.887 | 0.751 | 0.896 | 500 140 0.4 |
| M1229 | 128 | exp02 | 1024bit-ECFP6 | DNN3 | 0.853 | 0.865 | 0.839 | 0.865 | 0.865 | 0.704 | 0.899 | 500 60 0.5 |
| M1230 | 128 | exp03 | 1024bit-ECFP6 | DNN3 | 0.853 | 0.850 | 0.814 | 0.887 | 0.868 | 0.704 | 0.913 | 400 140 0.4 |
| M1231 | 128 | exp01 | 2048bit-ECFP6 | KNN | 0.888 | 0.864 | 0.822 | 0.943 | 0.902 | 0.776 | 0.918 | 5 distance |
| M1232 | 128 | exp02 | 2048bit-ECFP6 | KNN | 0.822 | 0.803 | 0.737 | 0.894 | 0.846 | 0.643 | 0.919 | 5 uniform |
| M1233 | 128 | exp03 | 2048bit-ECFP6 | KNN | 0.884 | 0.849 | 0.797 | 0.957 | 0.900 | 0.771 | 0.913 | 3 distance |
| M1234 | 128 | exp04 | 2048bit-ECFP6 | KNN | 0.888 | 0.864 | 0.822 | 0.943 | 0.902 | 0.776 | 0.914 | 5 uniform |
| M1235 | 128 | exp05 | 2048bit-ECFP6 | KNN | 0.896 | 0.870 | 0.831 | 0.950 | 0.908 | 0.792 | 0.908 | 3 distance |
| M1236 | 128 | exp06 | 2048bit-ECFP6 | KNN | 0.907 | 0.921 | 0.907 | 0.908 | 0.914 | 0.814 | 0.907 | 3 uniform |
| M1237 | 128 | exp07 | 2048bit-ECFP6 | KNN | 0.915 | 0.894 | 0.864 | 0.957 | 0.925 | 0.830 | 0.898 | 3 uniform |
| M1238 | 128 | exp08 | 2048bit-ECFP6 | KNN | 0.903 | 0.892 | 0.864 | 0.936 | 0.913 | 0.806 | 0.903 | 3 uniform |
| M1239 | 128 | exp09 | 2048bit-ECFP6 | KNN | 0.907 | 0.898 | 0.873 | 0.936 | 0.917 | 0.813 | 0.900 | 3 distance |
| M1240 | 128 | exp10 | 2048bit-ECFP6 | KNN | 0.884 | 0.854 | 0.805 | 0.950 | 0.899 | 0.770 | 0.910 | 5 uniform |
| M1241 | 128 | exp11 | 2048bit-ECFP6 | KNN | 0.903 | 0.887 | 0.856 | 0.943 | 0.914 | 0.806 | 0.905 | 7 distance |
| M1242 | 128 | exp12 | 2048bit-ECFP6 | KNN | 0.907 | 0.898 | 0.873 | 0.936 | 0.917 | 0.813 | 0.912 | 5 distance |
| M1243 | 128 | exp13 | 2048bit-ECFP6 | KNN | 0.884 | 0.863 | 0.822 | 0.936 | 0.898 | 0.768 | 0.908 | 3 uniform |
| M1244 | 128 | exp14 | 2048bit-ECFP6 | KNN | 0.892 | 0.865 | 0.822 | 0.950 | 0.905 | 0.785 | 0.909 | 7 distance |
| M1245 | 128 | exp15 | 2048bit-ECFP6 | KNN | 0.880 | 0.857 | 0.814 | 0.936 | 0.895 | 0.761 | 0.909 | 3 uniform |
| M1246 | 128 | exp16 | 2048bit-ECFP6 | KNN | 0.903 | 0.892 | 0.864 | 0.936 | 0.913 | 0.806 | 0.911 | 3 distance |
| M1247 | 128 | exp17 | 2048bit-ECFP6 | KNN | 0.915 | 0.905 | 0.881 | 0.943 | 0.924 | 0.829 | 0.915 | 3 uniform |
| M1248 | 128 | exp18 | 2048bit-ECFP6 | KNN | 0.931 | 0.930 | 0.915 | 0.943 | 0.937 | 0.860 | 0.908 | 3 uniform |
| M1249 | 128 | exp19 | 2048bit-ECFP6 | KNN | 0.927 | 0.907 | 0.881 | 0.965 | 0.935 | 0.853 | 0.908 | 3 distance |
| M1250 | 128 | exp01 | 2048bit-ECFP6 | SVM | 0.873 | 0.865 | 0.831 | 0.908 | 0.886 | 0.743 | 0.919 | 1 0.1 |
| M1251 | 128 | exp02 | 2048bit-ECFP6 | SVM | 0.861 | 0.848 | 0.805 | 0.908 | 0.877 | 0.720 | 0.920 | 1 0.1 |
| M1252 | 128 | exp03 | 2048bit-ECFP6 | SVM | 0.873 | 0.865 | 0.831 | 0.908 | 0.886 | 0.743 | 0.928 | 5 0.1 |
| M1253 | 128 | exp04 | 2048bit-ECFP6 | SVM | 0.892 | 0.884 | 0.856 | 0.922 | 0.903 | 0.782 | 0.907 | 5 0.1 |
| M1254 | 128 | exp05 | 2048bit-ECFP6 | SVM | 0.884 | 0.872 | 0.839 | 0.922 | 0.897 | 0.767 | 0.909 | 10 0.1 |
| M1255 | 128 | exp06 | 2048bit-ECFP6 | SVM | 0.900 | 0.914 | 0.898 | 0.901 | 0.907 | 0.798 | 0.909 | 1 0.1 |
| M1256 | 128 | exp07 | 2048bit-ECFP6 | SVM | 0.876 | 0.871 | 0.839 | 0.908 | 0.889 | 0.751 | 0.903 | 10 0.01 |
| M1257 | 128 | exp08 | 2048bit-ECFP6 | SVM | 0.919 | 0.911 | 0.890 | 0.943 | 0.927 | 0.837 | 0.910 | 1 0.1 |
| M1258 | 128 | exp09 | 2048bit-ECFP6 | SVM | 0.919 | 0.929 | 0.915 | 0.922 | 0.925 | 0.837 | 0.908 | 5 0.05 |
| M1259 | 128 | exp10 | 2048bit-ECFP6 | SVM | 0.888 | 0.864 | 0.822 | 0.943 | 0.902 | 0.776 | 0.902 | 1 0.1 |
| M1260 | 128 | exp11 | 2048bit-ECFP6 | SVM | 0.903 | 0.908 | 0.890 | 0.915 | 0.912 | 0.805 | 0.913 | 5 0.05 |
| M1261 | 128 | exp12 | 2048bit-ECFP6 | SVM | 0.892 | 0.901 | 0.881 | 0.901 | 0.901 | 0.782 | 0.917 | 5 0.05 |
| M1262 | 128 | exp13 | 2048bit-ECFP6 | SVM | 0.896 | 0.890 | 0.864 | 0.922 | 0.906 | 0.790 | 0.918 | 5 0.1 |
| M1263 | 128 | exp14 | 2048bit-ECFP6 | SVM | 0.900 | 0.876 | 0.839 | 0.950 | 0.912 | 0.799 | 0.913 | 1 0.1 |
| M1264 | 128 | exp15 | 2048bit-ECFP6 | SVM | 0.888 | 0.864 | 0.822 | 0.943 | 0.902 | 0.776 | 0.908 | 10 0.05 |
| M1265 | 128 | exp16 | 2048bit-ECFP6 | SVM | 0.915 | 0.899 | 0.873 | 0.950 | 0.924 | 0.829 | 0.912 | 5 0.05 |
| M1266 | 128 | exp17 | 2048bit-ECFP6 | SVM | 0.884 | 0.899 | 0.881 | 0.887 | 0.893 | 0.767 | 0.911 | 10 0.1 |
| M1267 | 128 | exp18 | 2048bit-ECFP6 | SVM | 0.927 | 0.949 | 0.941 | 0.915 | 0.931 | 0.853 | 0.911 | 5 0.1 |
| M1268 | 128 | exp19 | 2048bit-ECFP6 | SVM | 0.915 | 0.899 | 0.873 | 0.950 | 0.924 | 0.829 | 0.913 | 1 0.1 |
| M1269 | 128 | exp01 | 2048bit-ECFP6 | GBM | 0.884 | 0.888 | 0.864 | 0.901 | 0.894 | 0.766 | 0.915 | 200 0.3 |
| M1270 | 128 | exp02 | 2048bit-ECFP6 | GBM | 0.853 | 0.846 | 0.805 | 0.894 | 0.869 | 0.704 | 0.907 | 300 0.7 |
| M1271 | 128 | exp03 | 2048bit-ECFP6 | GBM | 0.888 | 0.878 | 0.847 | 0.922 | 0.900 | 0.774 | 0.916 | 100 0.5 |
| M1272 | 128 | exp04 | 2048bit-ECFP6 | GBM | 0.892 | 0.884 | 0.856 | 0.922 | 0.903 | 0.782 | 0.902 | 100 0.4 |
| M1273 | 128 | exp05 | 2048bit-ECFP6 | GBM | 0.873 | 0.870 | 0.839 | 0.901 | 0.885 | 0.743 | 0.909 | 800 0.5 |
| M1274 | 128 | exp06 | 2048bit-ECFP6 | GBM | 0.915 | 0.922 | 0.907 | 0.922 | 0.922 | 0.829 | 0.908 | 50 0.4 |
| M1275 | 128 | exp07 | 2048bit-ECFP6 | GBM | 0.884 | 0.878 | 0.847 | 0.915 | 0.896 | 0.766 | 0.898 | 400 0.1 |
| M1276 | 128 | exp08 | 2048bit-ECFP6 | GBM | 0.876 | 0.876 | 0.847 | 0.901 | 0.888 | 0.751 | 0.899 | 200 0.5 |
| M1277 | 128 | exp09 | 2048bit-ECFP6 | GBM | 0.907 | 0.940 | 0.932 | 0.887 | 0.912 | 0.816 | 0.894 | 300 0.5 |
| M1278 | 128 | exp10 | 2048bit-ECFP6 | GBM | 0.892 | 0.855 | 0.805 | 0.965 | 0.907 | 0.787 | 0.907 | 100 0.2 |
| M1279 | 128 | exp11 | 2048bit-ECFP6 | GBM | 0.919 | 0.929 | 0.915 | 0.922 | 0.925 | 0.837 | 0.911 | 300 0.1 |
| M1280 | 128 | exp12 | 2048bit-ECFP6 | GBM | 0.896 | 0.901 | 0.881 | 0.908 | 0.905 | 0.790 | 0.903 | 50 0.4 |
| M1281 | 128 | exp13 | 2048bit-ECFP6 | GBM | 0.876 | 0.852 | 0.805 | 0.936 | 0.892 | 0.753 | 0.914 | 200 0.2 |
| M1282 | 128 | exp14 | 2048bit-ECFP6 | GBM | 0.907 | 0.877 | 0.839 | 0.965 | 0.919 | 0.816 | 0.905 | 100 0.2 |
| M1283 | 128 | exp15 | 2048bit-ECFP6 | GBM | 0.892 | 0.865 | 0.822 | 0.950 | 0.905 | 0.785 | 0.904 | 300 0.1 |
| M1284 | 128 | exp16 | 2048bit-ECFP6 | GBM | 0.896 | 0.880 | 0.847 | 0.936 | 0.907 | 0.790 | 0.901 | 100 0.5 |
| M1285 | 128 | exp17 | 2048bit-ECFP6 | GBM | 0.900 | 0.920 | 0.907 | 0.894 | 0.906 | 0.799 | 0.908 | 500 0.6 |
| M1286 | 128 | exp18 | 2048bit-ECFP6 | GBM | 0.915 | 0.947 | 0.941 | 0.894 | 0.920 | 0.831 | 0.899 | 600 0.2 |
| M1287 | 128 | exp19 | 2048bit-ECFP6 | GBM | 0.931 | 0.924 | 0.907 | 0.950 | 0.937 | 0.860 | 0.896 | 50 0.5 |
| M1288 | 128 | exp01 | 2048bit-ECFP6 | RF | 0.888 | 0.873 | 0.839 | 0.929 | 0.900 | 0.775 | 0.920 | 300 |
| M1289 | 128 | exp02 | 2048bit-ECFP6 | RF | 0.849 | 0.819 | 0.754 | 0.929 | 0.870 | 0.700 | 0.917 | 700 |
| M1290 | 128 | exp03 | 2048bit-ECFP6 | RF | 0.888 | 0.850 | 0.797 | 0.965 | 0.904 | 0.780 | 0.924 | 300 |
| M1291 | 128 | exp04 | 2048bit-ECFP6 | RF | 0.892 | 0.869 | 0.831 | 0.943 | 0.905 | 0.784 | 0.918 | 400 |
| M1292 | 128 | exp05 | 2048bit-ECFP6 | RF | 0.888 | 0.859 | 0.814 | 0.950 | 0.902 | 0.777 | 0.909 | 300 |
| M1293 | 128 | exp06 | 2048bit-ECFP6 | RF | 0.911 | 0.934 | 0.924 | 0.901 | 0.917 | 0.822 | 0.909 | 100 |
| M1294 | 128 | exp07 | 2048bit-ECFP6 | RF | 0.911 | 0.888 | 0.856 | 0.957 | 0.922 | 0.823 | 0.902 | 700 |
| M1295 | 128 | exp08 | 2048bit-ECFP6 | RF | 0.923 | 0.917 | 0.898 | 0.943 | 0.930 | 0.844 | 0.904 | 200 |
| M1296 | 128 | exp09 | 2048bit-ECFP6 | RF | 0.927 | 0.918 | 0.898 | 0.950 | 0.934 | 0.852 | 0.902 | 800 |
| M1297 | 128 | exp10 | 2048bit-ECFP6 | RF | 0.892 | 0.869 | 0.831 | 0.943 | 0.905 | 0.784 | 0.911 | 500 |
| M1298 | 128 | exp11 | 2048bit-ECFP6 | RF | 0.915 | 0.894 | 0.864 | 0.957 | 0.925 | 0.830 | 0.910 | 50 |
| M1299 | 128 | exp12 | 2048bit-ECFP6 | RF | 0.903 | 0.892 | 0.864 | 0.936 | 0.913 | 0.806 | 0.914 | 900 |
| M1300 | 128 | exp13 | 2048bit-ECFP6 | RF | 0.892 | 0.879 | 0.847 | 0.929 | 0.903 | 0.782 | 0.912 | 700 |
| M1301 | 128 | exp14 | 2048bit-ECFP6 | RF | 0.896 | 0.861 | 0.814 | 0.965 | 0.910 | 0.795 | 0.911 | 700 |
| M1302 | 128 | exp15 | 2048bit-ECFP6 | RF | 0.896 | 0.870 | 0.831 | 0.950 | 0.908 | 0.792 | 0.906 | 900 |
| M1303 | 128 | exp16 | 2048bit-ECFP6 | RF | 0.915 | 0.905 | 0.881 | 0.943 | 0.924 | 0.829 | 0.909 | 600 |
| M1304 | 128 | exp17 | 2048bit-ECFP6 | RF | 0.896 | 0.901 | 0.881 | 0.908 | 0.905 | 0.790 | 0.918 | 900 |
| M1305 | 128 | exp18 | 2048bit-ECFP6 | RF | 0.931 | 0.949 | 0.941 | 0.922 | 0.935 | 0.861 | 0.908 | 900 |
| M1306 | 128 | exp19 | 2048bit-ECFP6 | RF | 0.915 | 0.889 | 0.856 | 0.965 | 0.925 | 0.831 | 0.910 | 100 |
| M1307 | 128 | exp01 | 2048bit-ECFP6 | DNN2 | 0.876 | 0.871 | 0.839 | 0.908 | 0.889 | 0.751 | 0.910 | 400 100 0.1 |
| M1308 | 128 | exp02 | 2048bit-ECFP6 | DNN2 | 0.865 | 0.868 | 0.839 | 0.887 | 0.877 | 0.727 | 0.906 | 400 60 0.3 |
| M1309 | 128 | exp03 | 2048bit-ECFP6 | DNN2 | 0.873 | 0.860 | 0.822 | 0.915 | 0.887 | 0.743 | 0.912 | 600 100 0.5 |
| M1310 | 128 | exp01 | 2048bit-ECFP6 | DNN3 | 0.896 | 0.896 | 0.873 | 0.915 | 0.905 | 0.790 | 0.912 | 500 160 0.5 |
| M1311 | 128 | exp02 | 2048bit-ECFP6 | DNN3 | 0.853 | 0.865 | 0.839 | 0.865 | 0.865 | 0.704 | 0.909 | 400 100 0.3 |
| M1312 | 128 | exp03 | 2048bit-ECFP6 | DNN3 | 0.876 | 0.861 | 0.822 | 0.922 | 0.890 | 0.752 | 0.914 | 200 100 0.2 |

* Optimal parameters for each machine-learning methods as follows

(1) KNN: two parameters are the number of nearest neighbors (K) and weighting schemes (uniform weight or distance-dependent weight) respectively

(2) SVM: two parameters are the penalty parameter C and kernel parameter gamma respectively

(3) RF: one parameter is the number of decision trees

(4) GBM: two parameters are the number of decision trees and the learning rate

(5) DNN2 and DNN3: three parameters are number of epochs, the size of mini-batches, and the dropout rate

**Table S2.** All the average models for the bitterant/non-bitterant classification by averaging over different data-splitting schemes.

| **AM** | **FS** | **FP** | **method** | **Accuracy**  **(test)** | **Precision**  **(test)** | **Specificity**  **(test)** | **Sensitivity**  **(test)** | **F1-score**  **(test)** | **MCC**  **(test)** | **F1-score**  **(CV)** | $\boldsymbol{\Delta}$**F1-score** |
| --- | --- | --- | --- | --- | --- | --- | --- | --- | --- | --- | --- |
| AM01 | full | 1024bit-ECFP4 | KNN | 0.890(0.019) | 0.910(0.021) | 0.895(0.027) | 0.886(0.024) | 0.898(0.018) | 0.780(0.038) | 0.891(0.007) | 0.018(0.013) |
| AM02 | full | 1024bit-ECFP4 | SVM | 0.908(0.017) | 0.900(0.024) | 0.875(0.034) | 0.935(0.019) | 0.917(0.015) | 0.815(0.035) | 0.915(0.005) | 0.014(0.010) |
| AM03 | full | 1024bit-ECFP4 | GBM | 0.893(0.018) | 0.897(0.023) | 0.875(0.030) | 0.908(0.018) | 0.902(0.016) | 0.784(0.036) | 0.900(0.006) | 0.016(0.010) |
| AM04 | full | 1024bit-ECFP4 | RF | 0.909(0.018) | 0.899(0.023) | 0.872(0.032) | 0.939(0.020) | 0.918(0.016) | 0.816(0.036) | 0.914(0.005) | 0.015(0.010) |
| AM05 | full | 1024bit-ECFP4 | DNN2 | 0.896(0.006) | 0.903(0.008) | 0.884(0.010) | 0.906(0.003) | 0.905(0.005) | 0.790(0.013) | 0.918(0.005) | 0.013(0.010) |
| AM06 | full | 1024bit-ECFP4 | DNN3 | 0.907(0.003) | 0.909(0.008) | 0.890(0.012) | 0.922(0.006) | 0.915(0.002) | 0.813(0.007) | 0.917(0.001) | 0.002(0.002) |
| AM07 | full | 2048bit-ECFP4 | KNN | 0.893(0.015) | 0.915(0.016) | 0.901(0.020) | 0.887(0.022) | 0.900(0.014) | 0.786(0.031) | 0.894(0.006) | 0.013(0.012) |
| AM08 | full | 2048bit-ECFP4 | SVM | 0.911(0.018) | 0.903(0.024) | 0.879(0.034) | 0.938(0.022) | 0.920(0.016) | 0.821(0.036) | 0.919(0.005) | 0.016(0.010) |
| AM09 | full | 2048bit-ECFP4 | GBM | 0.894(0.018) | 0.894(0.021) | 0.869(0.029) | 0.916(0.020) | 0.904(0.016) | 0.787(0.035) | 0.896(0.007) | 0.017(0.014) |
| AM10 | full | 2048bit-ECFP4 | RF | 0.911(0.016) | 0.902(0.021) | 0.878(0.029) | 0.939(0.021) | 0.920(0.014) | 0.821(0.031) | 0.915(0.006) | 0.015(0.010) |
| AM11 | full | 2048bit-ECFP4 | DNN2 | 0.909(0.008) | 0.910(0.014) | 0.890(0.018) | 0.924(0.003) | 0.917(0.007) | 0.816(0.016) | 0.921(0.002) | 0.008(0.002) |
| AM12 | full | 2048bit-ECFP4 | DNN3 | 0.893(0.007) | 0.882(0.013) | 0.850(0.021) | 0.929(0.020) | 0.905(0.007) | 0.785(0.015) | 0.893(0.004) | 0.012(0.011) |
| AM13 | full | 1024bit-ECFP6 | KNN | 0.887(0.018) | 0.889(0.019) | 0.864(0.026) | 0.907(0.028) | 0.898(0.016) | 0.773(0.036) | 0.890(0.006) | 0.017(0.011) |
| AM14 | full | 1024bit-ECFP6 | SVM | 0.901(0.018) | 0.890(0.023) | 0.861(0.033) | 0.935(0.027) | 0.912(0.016) | 0.802(0.037) | 0.907(0.006) | 0.016(0.013) |
| AM15 | full | 1024bit-ECFP6 | GBM | 0.895(0.014) | 0.891(0.021) | 0.864(0.030) | 0.921(0.022) | 0.905(0.013) | 0.789(0.029) | 0.897(0.005) | 0.013(0.009) |
| AM16 | full | 1024bit-ECFP6 | RF | 0.905(0.017) | 0.888(0.021) | 0.856(0.030) | 0.946(0.020) | 0.916(0.015) | 0.810(0.034) | 0.910(0.005) | 0.015(0.011) |
| AM17 | full | 1024bit-ECFP6 | DNN2 | 0.889(0.020) | 0.887(0.019) | 0.862(0.024) | 0.913(0.020) | 0.900(0.018) | 0.777(0.041) | 0.909(0.006) | 0.019(0.017) |
| AM18 | full | 1024bit-ECFP6 | DNN3 | 0.891(0.013) | 0.890(0.014) | 0.864(0.018) | 0.913(0.009) | 0.901(0.011) | 0.779(0.026) | 0.906(0.005) | 0.013(0.011) |
| AM19 | full | 2048bit-ECFP6 | KNN | 0.888(0.018) | 0.891(0.018) | 0.867(0.026) | 0.905(0.029) | 0.898(0.017) | 0.774(0.036) | 0.889(0.005) | 0.018(0.011) |
| AM20 | full | 2048bit-ECFP6 | SVM | 0.908(0.020) | 0.902(0.026) | 0.877(0.036) | 0.933(0.014) | 0.917(0.017) | 0.814(0.039) | 0.914(0.005) | 0.015(0.011) |
| AM21 | full | 2048bit-ECFP6 | GBM | 0.891(0.018) | 0.885(0.022) | 0.857(0.030) | 0.921(0.022) | 0.902(0.016) | 0.782(0.036) | 0.895(0.006) | 0.015(0.014) |
| AM22 | full | 2048bit-ECFP6 | RF | 0.907(0.016) | 0.891(0.019) | 0.860(0.028) | 0.947(0.019) | 0.918(0.014) | 0.815(0.031) | 0.910(0.006) | 0.013(0.014) |
| AM23 | full | 2048bit-ECFP6 | DNN2 | 0.896(0.011) | 0.891(0.018) | 0.865(0.025) | 0.922(0.000) | 0.906(0.009) | 0.790(0.023) | 0.918(0.003) | 0.013(0.010) |
| AM24 | full | 2048bit-ECFP6 | DNN3 | 0.857(0.025) | 0.819(0.042) | 0.743(0.083) | 0.953(0.024) | 0.880(0.016) | 0.723(0.039) | 0.858(0.031) | 0.021(0.016) |
| AM25 | 512 | 1024bit-ECFP4 | KNN | 0.902(0.013) | 0.906(0.019) | 0.886(0.026) | 0.915(0.019) | 0.911(0.012) | 0.803(0.027) | 0.906(0.006) | 0.014(0.008) |
| AM26 | 512 | 1024bit-ECFP4 | SVM | 0.915(0.016) | 0.909(0.021) | 0.887(0.030) | 0.938(0.021) | 0.923(0.014) | 0.829(0.031) | 0.919(0.007) | 0.014(0.010) |
| AM27 | 512 | 1024bit-ECFP4 | GBM | 0.896(0.018) | 0.897(0.022) | 0.874(0.030) | 0.916(0.019) | 0.906(0.016) | 0.792(0.036) | 0.904(0.006) | 0.015(0.008) |
| AM28 | 512 | 1024bit-ECFP4 | RF | 0.913(0.019) | 0.901(0.023) | 0.875(0.031) | 0.945(0.019) | 0.922(0.017) | 0.826(0.038) | 0.915(0.006) | 0.016(0.013) |
| AM29 | 512 | 1024bit-ECFP4 | DNN2 | 0.896(0.006) | 0.907(0.009) | 0.890(0.012) | 0.901(0.006) | 0.904(0.005) | 0.790(0.013) | 0.920(0.004) | 0.016(0.009) |
| AM30 | 512 | 1024bit-ECFP4 | DNN3 | 0.893(0.007) | 0.911(0.003) | 0.895(0.004) | 0.891(0.012) | 0.901(0.007) | 0.786(0.014) | 0.919(0.003) | 0.018(0.009) |
| AM31 | 512 | 2048bit-ECFP4 | KNN | 0.913(0.017) | 0.911(0.023) | 0.891(0.031) | 0.931(0.021) | 0.921(0.015) | 0.824(0.033) | 0.914(0.006) | 0.014(0.012) |
| AM32 | 512 | 2048bit-ECFP4 | SVM | 0.917(0.011) | 0.911(0.020) | 0.889(0.029) | 0.939(0.017) | 0.925(0.010) | 0.833(0.022) | 0.922(0.007) | 0.009(0.010) |
| AM33 | 512 | 2048bit-ECFP4 | GBM | 0.898(0.014) | 0.892(0.020) | 0.865(0.029) | 0.926(0.020) | 0.908(0.012) | 0.796(0.028) | 0.906(0.005) | 0.012(0.011) |
| AM34 | 512 | 2048bit-ECFP4 | RF | 0.909(0.015) | 0.896(0.021) | 0.869(0.030) | 0.944(0.021) | 0.919(0.013) | 0.819(0.030) | 0.918(0.005) | 0.014(0.009) |
| AM35 | 512 | 2048bit-ECFP4 | DNN2 | 0.898(0.007) | 0.908(0.013) | 0.890(0.019) | 0.906(0.003) | 0.906(0.006) | 0.795(0.015) | 0.927(0.002) | 0.021(0.004) |
| AM36 | 512 | 2048bit-ECFP4 | DNN3 | 0.897(0.012) | 0.904(0.018) | 0.884(0.024) | 0.908(0.010) | 0.906(0.010) | 0.792(0.025) | 0.925(0.002) | 0.019(0.009) |
| AM37 | 512 | 1024bit-ECFP6 | KNN | 0.900(0.018) | 0.893(0.015) | 0.867(0.020) | 0.926(0.025) | 0.909(0.016) | 0.798(0.036) | 0.906(0.005) | 0.017(0.009) |
| AM38 | 512 | 1024bit-ECFP6 | SVM | 0.911(0.018) | 0.901(0.023) | 0.876(0.031) | 0.939(0.019) | 0.920(0.016) | 0.820(0.036) | 0.915(0.007) | 0.017(0.009) |
| AM39 | 512 | 1024bit-ECFP6 | GBM | 0.897(0.019) | 0.892(0.023) | 0.865(0.032) | 0.924(0.020) | 0.907(0.017) | 0.794(0.038) | 0.901(0.007) | 0.017(0.013) |
| AM40 | 512 | 1024bit-ECFP6 | RF | 0.908(0.018) | 0.888(0.021) | 0.856(0.030) | 0.952(0.023) | 0.919(0.016) | 0.817(0.037) | 0.913(0.006) | 0.016(0.013) |
| AM41 | 512 | 1024bit-ECFP6 | DNN2 | 0.869(0.011) | 0.874(0.015) | 0.847(0.021) | 0.887(0.016) | 0.880(0.011) | 0.735(0.023) | 0.916(0.010) | 0.036(0.018) |
| AM42 | 512 | 1024bit-ECFP6 | DNN3 | 0.874(0.003) | 0.888(0.008) | 0.867(0.010) | 0.879(0.006) | 0.884(0.003) | 0.746(0.008) | 0.915(0.006) | 0.032(0.009) |
| AM43 | 512 | 2048bit-ECFP6 | KNN | 0.905(0.020) | 0.892(0.021) | 0.863(0.028) | 0.939(0.023) | 0.915(0.018) | 0.809(0.040) | 0.914(0.005) | 0.017(0.013) |
| AM44 | 512 | 2048bit-ECFP6 | SVM | 0.913(0.015) | 0.906(0.020) | 0.883(0.028) | 0.937(0.023) | 0.921(0.013) | 0.825(0.029) | 0.920(0.005) | 0.015(0.008) |
| AM45 | 512 | 2048bit-ECFP6 | GBM | 0.892(0.016) | 0.882(0.019) | 0.852(0.028) | 0.926(0.024) | 0.903(0.014) | 0.783(0.031) | 0.902(0.006) | 0.012(0.013) |
| AM46 | 512 | 2048bit-ECFP6 | RF | 0.909(0.015) | 0.889(0.018) | 0.857(0.026) | 0.953(0.021) | 0.920(0.014) | 0.819(0.031) | 0.916(0.006) | 0.015(0.009) |
| AM47 | 512 | 2048bit-ECFP6 | DNN2 | 0.875(0.003) | 0.867(0.007) | 0.833(0.010) | 0.910(0.003) | 0.888(0.003) | 0.748(0.007) | 0.921(0.002) | 0.032(0.002) |
| AM48 | 512 | 2048bit-ECFP6 | DNN3 | 0.887(0.002) | 0.878(0.007) | 0.848(0.012) | 0.920(0.012) | 0.898(0.002) | 0.772(0.004) | 0.919(0.003) | 0.021(0.001) |
| AM49 | 256 | 1024bit-ECFP4 | KNN | 0.911(0.018) | 0.907(0.023) | 0.884(0.031) | 0.933(0.022) | 0.920(0.016) | 0.822(0.036) | 0.913(0.005) | 0.016(0.010) |
| AM50 | 256 | 1024bit-ECFP4 | SVM | 0.912(0.016) | 0.909(0.025) | 0.886(0.035) | 0.935(0.021) | 0.921(0.014) | 0.825(0.032) | 0.920(0.005) | 0.014(0.010) |
| AM51 | 256 | 1024bit-ECFP4 | GBM | 0.898(0.016) | 0.897(0.023) | 0.873(0.031) | 0.919(0.020) | 0.908(0.014) | 0.795(0.032) | 0.909(0.005) | 0.012(0.010) |
| AM52 | 256 | 1024bit-ECFP4 | RF | 0.911(0.021) | 0.898(0.027) | 0.870(0.037) | 0.946(0.023) | 0.921(0.018) | 0.823(0.042) | 0.917(0.005) | 0.016(0.013) |
| AM53 | 256 | 1024bit-ECFP4 | DNN2 | 0.876(0.003) | 0.883(0.006) | 0.859(0.011) | 0.891(0.014) | 0.887(0.004) | 0.751(0.006) | 0.917(0.003) | 0.030(0.006) |
| AM54 | 256 | 1024bit-ECFP4 | DNN3 | 0.876(0.006) | 0.887(0.008) | 0.864(0.012) | 0.887(0.016) | 0.887(0.007) | 0.751(0.012) | 0.917(0.002) | 0.031(0.006) |
| AM55 | 256 | 2048bit-ECFP4 | KNN | 0.912(0.017) | 0.905(0.022) | 0.881(0.030) | 0.937(0.025) | 0.920(0.015) | 0.823(0.035) | 0.916(0.005) | 0.015(0.010) |
| AM56 | 256 | 2048bit-ECFP4 | SVM | 0.913(0.016) | 0.910(0.024) | 0.888(0.033) | 0.933(0.017) | 0.921(0.014) | 0.824(0.031) | 0.920(0.007) | 0.015(0.010) |
| AM57 | 256 | 2048bit-ECFP4 | GBM | 0.896(0.021) | 0.891(0.026) | 0.863(0.036) | 0.924(0.020) | 0.907(0.018) | 0.791(0.042) | 0.912(0.006) | 0.018(0.012) |
| AM58 | 256 | 2048bit-ECFP4 | RF | 0.908(0.018) | 0.893(0.027) | 0.863(0.040) | 0.945(0.019) | 0.918(0.015) | 0.815(0.036) | 0.919(0.006) | 0.016(0.009) |
| AM59 | 256 | 2048bit-ECFP4 | DNN2 | 0.892(0.022) | 0.894(0.025) | 0.870(0.032) | 0.910(0.014) | 0.902(0.020) | 0.782(0.044) | 0.924(0.004) | 0.023(0.015) |
| AM60 | 256 | 2048bit-ECFP4 | DNN3 | 0.888(0.021) | 0.888(0.026) | 0.862(0.035) | 0.910(0.009) | 0.899(0.018) | 0.774(0.042) | 0.926(0.003) | 0.028(0.015) |
| AM61 | 256 | 1024bit-ECFP6 | KNN | 0.906(0.017) | 0.901(0.024) | 0.876(0.033) | 0.931(0.021) | 0.915(0.015) | 0.811(0.034) | 0.912(0.006) | 0.016(0.010) |
| AM62 | 256 | 1024bit-ECFP6 | SVM | 0.914(0.015) | 0.905(0.022) | 0.881(0.030) | 0.941(0.020) | 0.922(0.013) | 0.827(0.030) | 0.917(0.005) | 0.014(0.009) |
| AM63 | 256 | 1024bit-ECFP6 | GBM | 0.897(0.016) | 0.890(0.018) | 0.863(0.026) | 0.926(0.025) | 0.908(0.015) | 0.794(0.032) | 0.904(0.006) | 0.015(0.009) |
| AM64 | 256 | 1024bit-ECFP6 | RF | 0.907(0.016) | 0.888(0.021) | 0.856(0.031) | 0.949(0.022) | 0.917(0.014) | 0.814(0.032) | 0.914(0.005) | 0.014(0.009) |
| AM65 | 256 | 1024bit-ECFP6 | DNN2 | 0.869(0.006) | 0.876(0.006) | 0.850(0.008) | 0.884(0.007) | 0.880(0.006) | 0.735(0.013) | 0.913(0.002) | 0.034(0.006) |
| AM66 | 256 | 1024bit-ECFP6 | DNN3 | 0.876(0.006) | 0.890(0.006) | 0.870(0.008) | 0.882(0.014) | 0.886(0.006) | 0.751(0.012) | 0.915(0.004) | 0.029(0.005) |
| AM67 | 256 | 2048bit-ECFP6 | KNN | 0.907(0.017) | 0.895(0.021) | 0.867(0.030) | 0.941(0.026) | 0.917(0.015) | 0.815(0.034) | 0.915(0.005) | 0.014(0.012) |
| AM68 | 256 | 2048bit-ECFP6 | SVM | 0.908(0.016) | 0.907(0.024) | 0.885(0.032) | 0.928(0.018) | 0.917(0.014) | 0.816(0.032) | 0.919(0.006) | 0.013(0.007) |
| AM69 | 256 | 2048bit-ECFP6 | GBM | 0.898(0.018) | 0.889(0.022) | 0.861(0.031) | 0.928(0.015) | 0.908(0.016) | 0.794(0.036) | 0.909(0.006) | 0.014(0.012) |
| AM70 | 256 | 2048bit-ECFP6 | RF | 0.908(0.020) | 0.893(0.028) | 0.863(0.039) | 0.946(0.017) | 0.919(0.017) | 0.817(0.040) | 0.918(0.006) | 0.017(0.013) |
| AM71 | 256 | 2048bit-ECFP6 | DNN2 | 0.883(0.010) | 0.895(0.004) | 0.876(0.004) | 0.889(0.017) | 0.892(0.010) | 0.764(0.020) | 0.917(0.003) | 0.025(0.010) |
| AM72 | 256 | 2048bit-ECFP6 | DNN3 | 0.869(0.008) | 0.875(0.018) | 0.847(0.025) | 0.886(0.010) | 0.880(0.007) | 0.735(0.017) | 0.920(0.003) | 0.040(0.005) |
| AM73 | 128 | 1024bit-ECFP4 | KNN | 0.899(0.016) | 0.890(0.026) | 0.861(0.037) | 0.931(0.018) | 0.910(0.013) | 0.798(0.031) | 0.912(0.006) | 0.015(0.009) |
| AM74 | 128 | 1024bit-ECFP4 | SVM | 0.905(0.017) | 0.907(0.025) | 0.886(0.035) | 0.922(0.017) | 0.914(0.015) | 0.809(0.034) | 0.914(0.005) | 0.016(0.007) |
| AM75 | 128 | 1024bit-ECFP4 | GBM | 0.896(0.015) | 0.893(0.022) | 0.867(0.031) | 0.921(0.020) | 0.906(0.014) | 0.792(0.031) | 0.904(0.005) | 0.013(0.008) |
| AM76 | 128 | 1024bit-ECFP4 | RF | 0.908(0.018) | 0.892(0.026) | 0.863(0.038) | 0.945(0.019) | 0.918(0.016) | 0.815(0.036) | 0.916(0.005) | 0.015(0.011) |
| AM77 | 128 | 1024bit-ECFP4 | DNN2 | 0.865(0.009) | 0.879(0.014) | 0.856(0.018) | 0.872(0.006) | 0.876(0.007) | 0.728(0.017) | 0.911(0.002) | 0.035(0.005) |
| AM78 | 128 | 1024bit-ECFP4 | DNN3 | 0.882(0.021) | 0.889(0.015) | 0.867(0.016) | 0.893(0.025) | 0.891(0.020) | 0.761(0.042) | 0.909(0.003) | 0.024(0.011) |
| AM79 | 128 | 2048bit-ECFP4 | KNN | 0.899(0.019) | 0.885(0.024) | 0.853(0.035) | 0.937(0.023) | 0.910(0.017) | 0.797(0.039) | 0.913(0.005) | 0.016(0.013) |
| AM80 | 128 | 2048bit-ECFP4 | SVM | 0.902(0.015) | 0.899(0.022) | 0.875(0.031) | 0.925(0.020) | 0.912(0.014) | 0.803(0.031) | 0.914(0.005) | 0.010(0.011) |
| AM81 | 128 | 2048bit-ECFP4 | GBM | 0.896(0.019) | 0.888(0.027) | 0.860(0.038) | 0.926(0.017) | 0.906(0.016) | 0.790(0.038) | 0.906(0.006) | 0.015(0.012) |
| AM82 | 128 | 2048bit-ECFP4 | RF | 0.903(0.017) | 0.888(0.029) | 0.857(0.041) | 0.942(0.017) | 0.914(0.014) | 0.806(0.034) | 0.915(0.006) | 0.014(0.012) |
| AM83 | 128 | 2048bit-ECFP4 | DNN2 | 0.880(0.019) | 0.878(0.013) | 0.850(0.014) | 0.905(0.024) | 0.892(0.018) | 0.759(0.039) | 0.915(0.003) | 0.023(0.016) |
| AM84 | 128 | 2048bit-ECFP4 | DNN3 | 0.874(0.020) | 0.874(0.015) | 0.844(0.017) | 0.898(0.024) | 0.886(0.019) | 0.745(0.041) | 0.916(0.005) | 0.031(0.017) |
| AM85 | 128 | 1024bit-ECFP6 | KNN | 0.896(0.015) | 0.889(0.022) | 0.860(0.031) | 0.927(0.020) | 0.907(0.013) | 0.792(0.030) | 0.906(0.005) | 0.013(0.009) |
| AM86 | 128 | 1024bit-ECFP6 | SVM | 0.904(0.019) | 0.896(0.029) | 0.868(0.041) | 0.934(0.018) | 0.914(0.016) | 0.808(0.037) | 0.911(0.006) | 0.016(0.012) |
| AM87 | 128 | 1024bit-ECFP6 | GBM | 0.887(0.019) | 0.883(0.023) | 0.855(0.031) | 0.915(0.023) | 0.898(0.017) | 0.773(0.039) | 0.900(0.005) | 0.012(0.013) |
| AM88 | 128 | 1024bit-ECFP6 | RF | 0.903(0.019) | 0.883(0.030) | 0.848(0.044) | 0.950(0.021) | 0.915(0.016) | 0.808(0.037) | 0.914(0.005) | 0.015(0.011) |
| AM89 | 128 | 1024bit-ECFP6 | DNN2 | 0.865(0.016) | 0.860(0.018) | 0.825(0.024) | 0.898(0.015) | 0.879(0.014) | 0.727(0.033) | 0.903(0.006) | 0.025(0.016) |
| AM90 | 128 | 1024bit-ECFP6 | DNN3 | 0.861(0.011) | 0.867(0.015) | 0.839(0.020) | 0.880(0.010) | 0.873(0.010) | 0.720(0.022) | 0.902(0.007) | 0.029(0.015) |
| AM91 | 128 | 2048bit-ECFP6 | KNN | 0.897(0.023) | 0.880(0.029) | 0.845(0.042) | 0.940(0.016) | 0.909(0.019) | 0.793(0.045) | 0.909(0.005) | 0.016(0.016) |
| AM92 | 128 | 2048bit-ECFP6 | SVM | 0.895(0.018) | 0.890(0.025) | 0.863(0.035) | 0.922(0.019) | 0.905(0.015) | 0.789(0.036) | 0.912(0.006) | 0.015(0.012) |
| AM93 | 128 | 2048bit-ECFP6 | GBM | 0.895(0.018) | 0.891(0.030) | 0.864(0.042) | 0.920(0.024) | 0.905(0.016) | 0.788(0.037) | 0.905(0.006) | 0.014(0.012) |
| AM94 | 128 | 2048bit-ECFP6 | RF | 0.901(0.018) | 0.886(0.030) | 0.854(0.044) | 0.941(0.018) | 0.912(0.015) | 0.803(0.036) | 0.911(0.006) | 0.016(0.011) |
| AM95 | 128 | 2048bit-ECFP6 | DNN2 | 0.871(0.005) | 0.866(0.005) | 0.833(0.008) | 0.903(0.012) | 0.884(0.005) | 0.740(0.010) | 0.909(0.002) | 0.025(0.003) |
| AM96 | 128 | 2048bit-ECFP6 | DNN3 | 0.875(0.018) | 0.874(0.016) | 0.845(0.021) | 0.901(0.025) | 0.887(0.016) | 0.749(0.035) | 0.912(0.002) | 0.025(0.015) |

**Table S3.** Evaluation metrics for the **Dataset-CV** with the shuffling labels before the Y-randomization test.

| **Exp** | **TP** | **TN** | **FP** | **FN** | **Accuracy**  **(test)** | **Precision**  **(test)** | **Specificity**  **(test)** | **Sensitivity**  **(test)** | **F1-score (test)** | **MCC**  **(test)** |
| --- | --- | --- | --- | --- | --- | --- | --- | --- | --- | --- |
| exp01 | 315 | 223 | 251 | 251 | 0.517 | 0.557 | 0.470 | 0.557 | 0.557 | 0.027 |
| exp02 | 323 | 231 | 243 | 243 | 0.533 | 0.571 | 0.487 | 0.571 | 0.571 | 0.058 |
| exp03 | 309 | 217 | 257 | 257 | 0.506 | 0.546 | 0.458 | 0.546 | 0.546 | 0.004 |
| exp04 | 308 | 216 | 258 | 258 | 0.504 | 0.544 | 0.456 | 0.544 | 0.544 | -0.000 |
| exp05 | 308 | 216 | 258 | 258 | 0.504 | 0.544 | 0.456 | 0.544 | 0.544 | -0.000 |
| exp06 | 301 | 209 | 265 | 265 | 0.490 | 0.532 | 0.441 | 0.532 | 0.532 | -0.027 |
| exp07 | 305 | 213 | 261 | 261 | 0.498 | 0.539 | 0.449 | 0.539 | 0.539 | -0.012 |
| exp08 | 313 | 221 | 253 | 253 | 0.513 | 0.553 | 0.466 | 0.553 | 0.553 | 0.019 |
| exp09 | 308 | 216 | 258 | 258 | 0.504 | 0.544 | 0.456 | 0.544 | 0.544 | -0.000 |
| exp10 | 313 | 221 | 253 | 253 | 0.513 | 0.553 | 0.466 | 0.553 | 0.553 | 0.019 |
| exp11 | 310 | 218 | 256 | 256 | 0.508 | 0.548 | 0.460 | 0.548 | 0.548 | 0.008 |
| exp12 | 323 | 231 | 243 | 243 | 0.533 | 0.571 | 0.487 | 0.571 | 0.571 | 0.058 |
| exp13 | 309 | 217 | 257 | 257 | 0.506 | 0.546 | 0.458 | 0.546 | 0.546 | 0.004 |
| exp14 | 302 | 210 | 264 | 264 | 0.492 | 0.534 | 0.443 | 0.534 | 0.534 | -0.023 |
| exp15 | 313 | 221 | 253 | 253 | 0.513 | 0.553 | 0.466 | 0.553 | 0.553 | 0.019 |
| exp16 | 300 | 208 | 266 | 266 | 0.488 | 0.530 | 0.439 | 0.530 | 0.530 | -0.031 |
| exp17 | 293 | 201 | 273 | 273 | 0.475 | 0.518 | 0.424 | 0.518 | 0.518 | -0.058 |
| exp18 | 310 | 218 | 256 | 256 | 0.508 | 0.548 | 0.460 | 0.548 | 0.548 | 0.008 |
| exp19 | 308 | 216 | 258 | 258 | 0.504 | 0.544 | 0.456 | 0.544 | 0.544 | -0.000 |

Note: “Exp” refers to the random data-splitting scheme.

**Table S4.** The performance of 96 average models after the Y-randomization test.

| **AM** | **FS** | **FP** | **method** | **Accuracy**  **(test)** | **Precision**  **(test)** | **Specificity**  **(test)** | **Sensitivity**  **(test)** | **F1-score**  **(test)** | **MCC**  **(test)** | **F1-score**  **(CV)** | $\boldsymbol{\Delta}$**F1-score** |
| --- | --- | --- | --- | --- | --- | --- | --- | --- | --- | --- | --- |
| AM01 | full | 1024bit-ECFP4 | KNN | 0.553(0.058) | 0.572(0.041) | 0.372(0.110) | 0.704(0.132) | 0.627(0.069) | 0.089(0.125) | 0.602(0.036) | 0.046(0.035) |
| AM02 | full | 1024bit-ECFP4 | SVM | 0.544(0.004) | 0.544(0.003) | 0.006(0.023) | 0.994(0.013) | 0.704(0.002) | -0.009(0.035) | 0.687(0.039) | 0.018(0.039) |
| AM03 | full | 1024bit-ECFP4 | GBM | 0.528(0.021) | 0.538(0.011) | 0.039(0.028) | 0.938(0.027) | 0.684(0.015) | -0.054(0.079) | 0.676(0.009) | 0.014(0.013) |
| AM04 | full | 1024bit-ECFP4 | RF | 0.536(0.038) | 0.559(0.031) | 0.323(0.077) | 0.714(0.035) | 0.626(0.026) | 0.039(0.087) | 0.600(0.014) | 0.036(0.024) |
| AM05 | full | 1024bit-ECFP4 | DNN2 | 0.545(0.008) | 0.572(0.005) | 0.421(0.008) | 0.648(0.020) | 0.608(0.012) | 0.070(0.015) | 0.582(0.012) | 0.026(0.023) |
| AM06 | full | 1024bit-ECFP4 | DNN3 | 0.538(0.002) | 0.567(0.004) | 0.418(0.049) | 0.638(0.044) | 0.600(0.017) | 0.058(0.004) | 0.578(0.013) | 0.023(0.028) |
| AM07 | full | 2048bit-ECFP4 | KNN | 0.552(0.057) | 0.572(0.042) | 0.370(0.118) | 0.705(0.129) | 0.627(0.067) | 0.085(0.120) | 0.603(0.035) | 0.043(0.032) |
| AM08 | full | 2048bit-ECFP4 | SVM | 0.544(0.001) | 0.544(0.001) | 0.001(0.002) | 1.000(0.002) | 0.705(0.001) | 0.004(0.015) | 0.700(0.007) | 0.005(0.007) |
| AM09 | full | 2048bit-ECFP4 | GBM | 0.531(0.017) | 0.539(0.009) | 0.041(0.026) | 0.940(0.029) | 0.686(0.013) | -0.040(0.074) | 0.678(0.009) | 0.014(0.012) |
| AM10 | full | 2048bit-ECFP4 | RF | 0.534(0.036) | 0.557(0.026) | 0.327(0.061) | 0.707(0.049) | 0.622(0.031) | 0.036(0.078) | 0.606(0.016) | 0.031(0.029) |
| AM11 | full | 2048bit-ECFP4 | DNN2 | 0.555(0.034) | 0.577(0.023) | 0.407(0.012) | 0.678(0.057) | 0.623(0.037) | 0.090(0.069) | 0.588(0.003) | 0.035(0.040) |
| AM12 | full | 2048bit-ECFP4 | DNN3 | 0.538(0.009) | 0.545(0.002) | 0.093(0.132) | 0.910(0.127) | 0.679(0.037) | 0.004(0.005) | 0.657(0.027) | 0.022(0.014) |
| AM13 | full | 1024bit-ECFP6 | KNN | 0.559(0.066) | 0.571(0.046) | 0.345(0.121) | 0.737(0.163) | 0.638(0.085) | 0.103(0.143) | 0.605(0.046) | 0.054(0.027) |
| AM14 | full | 1024bit-ECFP6 | SVM | 0.545(0.002) | 0.545(0.001) | 0.003(0.008) | 0.999(0.003) | 0.705(0.001) | 0.011(0.024) | 0.693(0.032) | 0.013(0.032) |
| AM15 | full | 1024bit-ECFP6 | GBM | 0.538(0.019) | 0.544(0.011) | 0.061(0.040) | 0.936(0.023) | 0.688(0.012) | -0.012(0.080) | 0.671(0.008) | 0.021(0.011) |
| AM16 | full | 1024bit-ECFP6 | RF | 0.527(0.040) | 0.552(0.033) | 0.317(0.079) | 0.702(0.055) | 0.617(0.035) | 0.020(0.087) | 0.593(0.015) | 0.038(0.023) |
| AM17 | full | 1024bit-ECFP6 | DNN2 | 0.522(0.030) | 0.556(0.026) | 0.410(0.063) | 0.617(0.021) | 0.585(0.019) | 0.027(0.066) | 0.578(0.011) | 0.021(0.014) |
| AM18 | full | 1024bit-ECFP6 | DNN3 | 0.521(0.030) | 0.553(0.024) | 0.390(0.032) | 0.631(0.041) | 0.589(0.031) | 0.022(0.061) | 0.585(0.018) | 0.025(0.013) |
| AM19 | full | 2048bit-ECFP6 | KNN | 0.558(0.072) | 0.570(0.055) | 0.356(0.125) | 0.727(0.174) | 0.633(0.094) | 0.105(0.155) | 0.606(0.046) | 0.054(0.035) |
| AM20 | full | 2048bit-ECFP6 | SVM | 0.544(0.003) | 0.544(0.002) | 0.006(0.012) | 0.995(0.010) | 0.704(0.003) | 0.006(0.027) | 0.692(0.031) | 0.012(0.031) |
| AM21 | full | 2048bit-ECFP6 | GBM | 0.540(0.014) | 0.544(0.008) | 0.051(0.028) | 0.949(0.023) | 0.692(0.010) | -0.002(0.061) | 0.675(0.007) | 0.018(0.012) |
| AM22 | full | 2048bit-ECFP6 | RF | 0.528(0.039) | 0.553(0.030) | 0.317(0.069) | 0.706(0.042) | 0.620(0.030) | 0.024(0.086) | 0.599(0.013) | 0.036(0.019) |
| AM23 | full | 2048bit-ECFP6 | DNN2 | 0.557(0.023) | 0.580(0.021) | 0.410(0.052) | 0.681(0.015) | 0.626(0.014) | 0.093(0.052) | 0.583(0.022) | 0.044(0.009) |
| AM24 | full | 2048bit-ECFP6 | DNN3 | 0.544(0.000) | 0.544(0.000) | 0.000(0.000) | 1.000(0.000) | 0.705(0.000) | 0.000(0.000) | 0.671(0.018) | 0.034(0.018) |
| AM25 | 512 | 1024bit-ECFP4 | KNN | 0.567(0.058) | 0.577(0.043) | 0.316(0.112) | 0.778(0.085) | 0.661(0.049) | 0.106(0.130) | 0.624(0.026) | 0.044(0.035) |
| AM26 | 512 | 1024bit-ECFP4 | SVM | 0.542(0.010) | 0.544(0.004) | 0.007(0.014) | 0.991(0.029) | 0.702(0.011) | 0.002(0.038) | 0.700(0.006) | 0.006(0.008) |
| AM27 | 512 | 1024bit-ECFP4 | GBM | 0.532(0.023) | 0.541(0.013) | 0.050(0.041) | 0.936(0.027) | 0.685(0.015) | -0.033(0.089) | 0.677(0.009) | 0.017(0.012) |
| AM28 | 512 | 1024bit-ECFP4 | RF | 0.526(0.039) | 0.552(0.029) | 0.328(0.052) | 0.692(0.047) | 0.613(0.034) | 0.021(0.084) | 0.611(0.014) | 0.031(0.031) |
| AM29 | 512 | 1024bit-ECFP4 | DNN2 | 0.537(0.006) | 0.568(0.004) | 0.438(0.028) | 0.620(0.030) | 0.592(0.014) | 0.058(0.011) | 0.595(0.006) | 0.014(0.004) |
| AM30 | 512 | 1024bit-ECFP4 | DNN3 | 0.552(0.019) | 0.583(0.019) | 0.461(0.045) | 0.629(0.020) | 0.604(0.014) | 0.090(0.041) | 0.603(0.005) | 0.016(0.004) |
| AM31 | 512 | 2048bit-ECFP4 | KNN | 0.566(0.052) | 0.575(0.040) | 0.285(0.116) | 0.801(0.057) | 0.668(0.035) | 0.095(0.124) | 0.639(0.020) | 0.038(0.025) |
| AM32 | 512 | 2048bit-ECFP4 | SVM | 0.545(0.005) | 0.545(0.003) | 0.006(0.018) | 0.997(0.009) | 0.705(0.003) | 0.008(0.040) | 0.702(0.005) | 0.003(0.005) |
| AM33 | 512 | 2048bit-ECFP4 | GBM | 0.533(0.019) | 0.541(0.010) | 0.047(0.027) | 0.939(0.027) | 0.686(0.014) | -0.031(0.084) | 0.679(0.008) | 0.015(0.012) |
| AM34 | 512 | 2048bit-ECFP4 | RF | 0.538(0.043) | 0.561(0.033) | 0.339(0.066) | 0.705(0.062) | 0.624(0.040) | 0.048(0.092) | 0.621(0.018) | 0.035(0.029) |
| AM35 | 512 | 2048bit-ECFP4 | DNN2 | 0.538(0.008) | 0.569(0.005) | 0.435(0.038) | 0.624(0.041) | 0.595(0.020) | 0.060(0.013) | 0.613(0.014) | 0.028(0.017) |
| AM36 | 512 | 2048bit-ECFP4 | DNN3 | 0.533(0.033) | 0.564(0.028) | 0.424(0.055) | 0.624(0.051) | 0.592(0.034) | 0.049(0.067) | 0.620(0.001) | 0.035(0.026) |
| AM37 | 512 | 1024bit-ECFP6 | KNN | 0.582(0.068) | 0.582(0.045) | 0.304(0.102) | 0.814(0.122) | 0.676(0.066) | 0.150(0.158) | 0.635(0.030) | 0.053(0.037) |
| AM38 | 512 | 1024bit-ECFP6 | SVM | 0.544(0.000) | 0.544(0.000) | 0.002(0.008) | 0.999(0.006) | 0.705(0.001) | 0.001(0.004) | 0.685(0.046) | 0.019(0.046) |
| AM39 | 512 | 1024bit-ECFP6 | GBM | 0.540(0.016) | 0.545(0.010) | 0.061(0.040) | 0.940(0.022) | 0.690(0.010) | -0.004(0.065) | 0.674(0.007) | 0.019(0.010) |
| AM40 | 512 | 1024bit-ECFP6 | RF | 0.541(0.042) | 0.562(0.032) | 0.333(0.075) | 0.715(0.057) | 0.629(0.036) | 0.051(0.092) | 0.617(0.017) | 0.033(0.026) |
| AM41 | 512 | 1024bit-ECFP6 | DNN2 | 0.530(0.016) | 0.565(0.018) | 0.443(0.053) | 0.603(0.017) | 0.583(0.005) | 0.046(0.037) | 0.601(0.013) | 0.018(0.013) |
| AM42 | 512 | 1024bit-ECFP6 | DNN3 | 0.553(0.005) | 0.580(0.007) | 0.438(0.034) | 0.650(0.022) | 0.613(0.007) | 0.090(0.013) | 0.602(0.018) | 0.024(0.012) |
| AM43 | 512 | 2048bit-ECFP6 | KNN | 0.585(0.051) | 0.580(0.036) | 0.239(0.102) | 0.875(0.048) | 0.697(0.033) | 0.144(0.130) | 0.658(0.018) | 0.046(0.029) |
| AM44 | 512 | 2048bit-ECFP6 | SVM | 0.544(0.000) | 0.544(0.000) | 0.000(0.000) | 1.000(0.000) | 0.705(0.000) | 0.000(0.000) | 0.687(0.043) | 0.018(0.043) |
| AM45 | 512 | 2048bit-ECFP6 | GBM | 0.538(0.017) | 0.544(0.009) | 0.057(0.030) | 0.940(0.030) | 0.689(0.014) | -0.005(0.072) | 0.678(0.008) | 0.017(0.013) |
| AM46 | 512 | 2048bit-ECFP6 | RF | 0.528(0.041) | 0.554(0.032) | 0.329(0.080) | 0.695(0.049) | 0.616(0.033) | 0.024(0.090) | 0.635(0.014) | 0.031(0.024) |
| AM47 | 512 | 2048bit-ECFP6 | DNN2 | 0.538(0.016) | 0.569(0.015) | 0.429(0.045) | 0.629(0.039) | 0.596(0.020) | 0.059(0.033) | 0.628(0.018) | 0.032(0.005) |
| AM48 | 512 | 2048bit-ECFP6 | DNN3 | 0.547(0.008) | 0.570(0.014) | 0.364(0.098) | 0.700(0.067) | 0.626(0.018) | 0.067(0.029) | 0.635(0.006) | 0.019(0.014) |
| AM49 | 256 | 1024bit-ECFP4 | KNN | 0.558(0.056) | 0.572(0.044) | 0.307(0.113) | 0.767(0.062) | 0.654(0.042) | 0.080(0.128) | 0.625(0.025) | 0.043(0.033) |
| AM50 | 256 | 1024bit-ECFP4 | SVM | 0.544(0.001) | 0.544(0.000) | 0.001(0.004) | 0.999(0.004) | 0.705(0.001) | -0.002(0.013) | 0.694(0.030) | 0.011(0.030) |
| AM51 | 256 | 1024bit-ECFP4 | GBM | 0.529(0.018) | 0.539(0.010) | 0.047(0.034) | 0.931(0.018) | 0.683(0.011) | -0.052(0.076) | 0.679(0.007) | 0.012(0.008) |
| AM52 | 256 | 1024bit-ECFP4 | RF | 0.539(0.041) | 0.562(0.033) | 0.351(0.078) | 0.696(0.051) | 0.621(0.033) | 0.049(0.091) | 0.616(0.012) | 0.027(0.022) |
| AM53 | 256 | 1024bit-ECFP4 | DNN2 | 0.522(0.008) | 0.552(0.006) | 0.359(0.017) | 0.660(0.006) | 0.601(0.004) | 0.019(0.016) | 0.606(0.019) | 0.016(0.002) |
| AM54 | 256 | 1024bit-ECFP4 | DNN3 | 0.539(0.016) | 0.569(0.014) | 0.421(0.054) | 0.639(0.035) | 0.601(0.016) | 0.060(0.036) | 0.608(0.012) | 0.020(0.014) |
| AM55 | 256 | 2048bit-ECFP4 | KNN | 0.561(0.057) | 0.571(0.041) | 0.284(0.103) | 0.794(0.058) | 0.663(0.042) | 0.086(0.135) | 0.636(0.019) | 0.039(0.032) |
| AM56 | 256 | 2048bit-ECFP4 | SVM | 0.544(0.003) | 0.545(0.003) | 0.011(0.036) | 0.991(0.027) | 0.703(0.006) | 0.005(0.022) | 0.694(0.032) | 0.013(0.032) |
| AM57 | 256 | 2048bit-ECFP4 | GBM | 0.544(0.021) | 0.547(0.012) | 0.063(0.040) | 0.947(0.026) | 0.693(0.014) | 0.016(0.092) | 0.683(0.008) | 0.014(0.014) |
| AM58 | 256 | 2048bit-ECFP4 | RF | 0.537(0.044) | 0.561(0.035) | 0.346(0.082) | 0.697(0.058) | 0.621(0.037) | 0.045(0.095) | 0.623(0.014) | 0.030(0.025) |
| AM59 | 256 | 2048bit-ECFP4 | DNN2 | 0.505(0.020) | 0.543(0.018) | 0.432(0.025) | 0.565(0.029) | 0.554(0.022) | -0.003(0.040) | 0.609(0.018) | 0.055(0.023) |
| AM60 | 256 | 2048bit-ECFP4 | DNN3 | 0.516(0.010) | 0.553(0.010) | 0.447(0.029) | 0.574(0.012) | 0.564(0.006) | 0.021(0.022) | 0.613(0.020) | 0.049(0.019) |
| AM61 | 256 | 1024bit-ECFP6 | KNN | 0.565(0.053) | 0.572(0.035) | 0.290(0.087) | 0.795(0.092) | 0.664(0.051) | 0.103(0.124) | 0.634(0.024) | 0.042(0.032) |
| AM62 | 256 | 1024bit-ECFP6 | SVM | 0.542(0.006) | 0.543(0.002) | 0.002(0.008) | 0.995(0.018) | 0.703(0.007) | -0.006(0.022) | 0.657(0.064) | 0.048(0.064) |
| AM63 | 256 | 1024bit-ECFP6 | GBM | 0.539(0.021) | 0.545(0.013) | 0.070(0.047) | 0.932(0.027) | 0.688(0.013) | -0.001(0.077) | 0.677(0.008) | 0.016(0.011) |
| AM64 | 256 | 1024bit-ECFP6 | RF | 0.536(0.042) | 0.561(0.036) | 0.341(0.096) | 0.700(0.056) | 0.622(0.032) | 0.042(0.094) | 0.626(0.016) | 0.027(0.025) |
| AM65 | 256 | 1024bit-ECFP6 | DNN2 | 0.540(0.011) | 0.577(0.017) | 0.474(0.073) | 0.595(0.040) | 0.585(0.010) | 0.070(0.032) | 0.608(0.016) | 0.032(0.014) |
| AM66 | 256 | 1024bit-ECFP6 | DNN3 | 0.528(0.007) | 0.561(0.002) | 0.426(0.032) | 0.613(0.039) | 0.584(0.018) | 0.040(0.008) | 0.615(0.014) | 0.040(0.019) |
| AM67 | 256 | 2048bit-ECFP6 | KNN | 0.583(0.054) | 0.581(0.039) | 0.261(0.110) | 0.851(0.053) | 0.690(0.036) | 0.136(0.136) | 0.654(0.021) | 0.043(0.035) |
| AM68 | 256 | 2048bit-ECFP6 | SVM | 0.542(0.014) | 0.543(0.007) | 0.015(0.045) | 0.983(0.059) | 0.700(0.022) | 0.002(0.032) | 0.697(0.032) | 0.015(0.039) |
| AM69 | 256 | 2048bit-ECFP6 | GBM | 0.541(0.020) | 0.545(0.011) | 0.067(0.036) | 0.937(0.023) | 0.689(0.013) | 0.003(0.085) | 0.683(0.009) | 0.013(0.013) |
| AM70 | 256 | 2048bit-ECFP6 | RF | 0.536(0.048) | 0.559(0.039) | 0.346(0.080) | 0.694(0.072) | 0.618(0.047) | 0.043(0.100) | 0.642(0.011) | 0.038(0.037) |
| AM71 | 256 | 2048bit-ECFP6 | DNN2 | 0.544(0.006) | 0.576(0.006) | 0.455(0.034) | 0.620(0.030) | 0.596(0.013) | 0.075(0.013) | 0.634(0.007) | 0.037(0.020) |
| AM72 | 256 | 2048bit-ECFP6 | DNN3 | 0.514(0.014) | 0.549(0.010) | 0.415(0.019) | 0.596(0.036) | 0.571(0.021) | 0.011(0.024) | 0.643(0.015) | 0.072(0.017) |
| AM73 | 128 | 1024bit-ECFP4 | KNN | 0.543(0.058) | 0.565(0.045) | 0.346(0.103) | 0.707(0.073) | 0.627(0.048) | 0.056(0.127) | 0.617(0.023) | 0.035(0.036) |
| AM74 | 128 | 1024bit-ECFP4 | SVM | 0.544(0.004) | 0.544(0.002) | 0.002(0.006) | 0.998(0.008) | 0.704(0.004) | 0.002(0.034) | 0.687(0.046) | 0.018(0.046) |
| AM75 | 128 | 1024bit-ECFP4 | GBM | 0.531(0.019) | 0.540(0.011) | 0.057(0.030) | 0.929(0.025) | 0.683(0.014) | -0.032(0.078) | 0.685(0.008) | 0.014(0.012) |
| AM76 | 128 | 1024bit-ECFP4 | RF | 0.548(0.032) | 0.569(0.026) | 0.359(0.063) | 0.706(0.043) | 0.630(0.027) | 0.069(0.070) | 0.616(0.018) | 0.026(0.019) |
| AM77 | 128 | 1024bit-ECFP4 | DNN2 | 0.514(0.003) | 0.549(0.004) | 0.410(0.040) | 0.600(0.033) | 0.573(0.014) | 0.010(0.009) | 0.594(0.013) | 0.027(0.014) |
| AM78 | 128 | 1024bit-ECFP4 | DNN3 | 0.495(0.021) | 0.535(0.019) | 0.407(0.064) | 0.570(0.032) | 0.551(0.016) | -0.024(0.049) | 0.594(0.020) | 0.043(0.019) |
| AM79 | 128 | 2048bit-ECFP4 | KNN | 0.542(0.056) | 0.561(0.041) | 0.309(0.090) | 0.738(0.069) | 0.637(0.047) | 0.051(0.124) | 0.626(0.023) | 0.035(0.034) |
| AM80 | 128 | 2048bit-ECFP4 | SVM | 0.544(0.004) | 0.544(0.002) | 0.010(0.029) | 0.991(0.029) | 0.703(0.008) | 0.003(0.028) | 0.685(0.046) | 0.021(0.046) |
| AM81 | 128 | 2048bit-ECFP4 | GBM | 0.539(0.028) | 0.545(0.015) | 0.081(0.055) | 0.921(0.059) | 0.684(0.025) | 0.007(0.094) | 0.685(0.008) | 0.019(0.023) |
| AM82 | 128 | 2048bit-ECFP4 | RF | 0.530(0.041) | 0.555(0.031) | 0.347(0.057) | 0.682(0.053) | 0.612(0.038) | 0.032(0.086) | 0.617(0.015) | 0.035(0.024) |
| AM83 | 128 | 2048bit-ECFP4 | DNN2 | 0.503(0.007) | 0.542(0.004) | 0.438(0.034) | 0.558(0.039) | 0.549(0.020) | -0.004(0.009) | 0.599(0.012) | 0.050(0.029) |
| AM84 | 128 | 2048bit-ECFP4 | DNN3 | 0.489(0.022) | 0.528(0.019) | 0.396(0.040) | 0.567(0.051) | 0.546(0.032) | -0.038(0.042) | 0.605(0.009) | 0.059(0.032) |
| AM85 | 128 | 1024bit-ECFP6 | KNN | 0.554(0.052) | 0.567(0.035) | 0.295(0.087) | 0.771(0.073) | 0.652(0.044) | 0.075(0.122) | 0.632(0.024) | 0.036(0.030) |
| AM86 | 128 | 1024bit-ECFP6 | SVM | 0.543(0.003) | 0.544(0.001) | 0.001(0.006) | 0.997(0.008) | 0.704(0.003) | -0.009(0.025) | 0.694(0.035) | 0.010(0.035) |
| AM87 | 128 | 1024bit-ECFP6 | GBM | 0.536(0.017) | 0.543(0.010) | 0.068(0.043) | 0.927(0.027) | 0.685(0.012) | -0.014(0.070) | 0.681(0.008) | 0.014(0.008) |
| AM88 | 128 | 1024bit-ECFP6 | RF | 0.542(0.032) | 0.563(0.025) | 0.344(0.065) | 0.707(0.049) | 0.626(0.028) | 0.054(0.069) | 0.626(0.015) | 0.025(0.015) |
| AM89 | 128 | 1024bit-ECFP6 | DNN2 | 0.517(0.027) | 0.553(0.023) | 0.421(0.038) | 0.598(0.018) | 0.574(0.021) | 0.019(0.056) | 0.616(0.015) | 0.042(0.016) |
| AM90 | 128 | 1024bit-ECFP6 | DNN3 | 0.516(0.012) | 0.552(0.011) | 0.424(0.021) | 0.594(0.009) | 0.572(0.009) | 0.017(0.025) | 0.618(0.017) | 0.046(0.020) |
| AM91 | 128 | 2048bit-ECFP6 | KNN | 0.560(0.047) | 0.569(0.033) | 0.259(0.116) | 0.811(0.082) | 0.666(0.039) | 0.083(0.120) | 0.648(0.023) | 0.029(0.027) |
| AM92 | 128 | 2048bit-ECFP6 | SVM | 0.543(0.002) | 0.545(0.002) | 0.017(0.070) | 0.985(0.063) | 0.701(0.018) | 0.002(0.007) | 0.695(0.031) | 0.013(0.034) |
| AM93 | 128 | 2048bit-ECFP6 | GBM | 0.535(0.026) | 0.543(0.014) | 0.084(0.063) | 0.912(0.065) | 0.680(0.027) | -0.008(0.100) | 0.688(0.009) | 0.024(0.023) |
| AM94 | 128 | 2048bit-ECFP6 | RF | 0.531(0.038) | 0.559(0.035) | 0.370(0.087) | 0.665(0.061) | 0.606(0.036) | 0.037(0.081) | 0.637(0.014) | 0.036(0.032) |
| AM95 | 128 | 2048bit-ECFP6 | DNN2 | 0.537(0.023) | 0.570(0.018) | 0.460(0.021) | 0.600(0.044) | 0.585(0.029) | 0.062(0.043) | 0.632(0.007) | 0.047(0.037) |
| AM96 | 128 | 2048bit-ECFP6 | DNN3 | 0.523(0.013) | 0.558(0.012) | 0.438(0.024) | 0.593(0.014) | 0.575(0.011) | 0.031(0.027) | 0.628(0.014) | 0.054(0.023) |

**Table S5.** Nineteen individual models used to construct the consensus model 1 (**CM01**).

| **Model** | **FS** | **EXP** | **FP** | **Method** | **Accuracy(test)** | **Precision (test)** | **Specificity(test)** | **Sensitivity(test)** | **F1-score**  **(test)** | **MCC**  **(test)** | **F1-score**  **(CV)** | **Parameters from CV** |
| --- | --- | --- | --- | --- | --- | --- | --- | --- | --- | --- | --- | --- |
| M0266 | full | exp01 | 2048bit-ECFP6 | SVM | 0.923 | 0.917 | 0.898 | 0.943 | 0.930 | 0.844 | 0.911 | 10 0.01 |
| M0081 | full | exp02 | 1024bit-ECFP4 | DNN3 | 0.911 | 0.921 | 0.907 | 0.915 | 0.918 | 0.821 | 0.919 | 400 60 0.1 |
| M0432 | 512 | exp03 | 2048bit-ECFP4 | SVM | 0.927 | 0.918 | 0.898 | 0.950 | 0.934 | 0.852 | 0.932 | 10000.0 0.1 |
| M0635 | 512 | exp04 | 2048bit-ECFP6 | RF | 0.923 | 0.895 | 0.864 | 0.972 | 0.932 | 0.847 | 0.913 | 400 |
| M0415 | 512 | exp05 | 2048bit-ECFP4 | KNN | 0.934 | 0.913 | 0.890 | 0.972 | 0.942 | 0.869 | 0.922 | 3 distance |
| M0719 | 256 | exp06 | 1024bit-ECFP4 | RF | 0.942 | 0.944 | 0.932 | 0.950 | 0.947 | 0.883 | 0.921 | 600 |
| M0846 | 256 | exp07 | 1024bit-ECFP6 | SVM | 0.931 | 0.913 | 0.890 | 0.965 | 0.938 | 0.861 | 0.912 | 1000.0 0.1 |
| M0601 | 512 | exp08 | 2048bit-ECFP6 | SVM | 0.938 | 0.937 | 0.924 | 0.950 | 0.944 | 0.875 | 0.919 | 1000.0 0.05 |
| M0394 | 512 | exp09 | 1024bit-ECFP4 | RF | 0.958 | 0.939 | 0.924 | 0.986 | 0.962 | 0.915 | 0.905 | 300 |
| M0666 | 256 | exp10 | 1024bit-ECFP4 | KNN | 0.923 | 0.906 | 0.881 | 0.957 | 0.931 | 0.845 | 0.910 | 5 distance |
| M1052 | 128 | exp11 | 1024bit-ECFP4 | RF | 0.927 | 0.907 | 0.881 | 0.965 | 0.935 | 0.853 | 0.910 | 200 |
| M0971 | 256 | exp12 | 2048bit-ECFP6 | RF | 0.915 | 0.905 | 0.881 | 0.943 | 0.924 | 0.829 | 0.928 | 100 |
| M0423 | 512 | exp13 | 2048bit-ECFP4 | KNN | 0.911 | 0.910 | 0.890 | 0.929 | 0.919 | 0.821 | 0.918 | 1 uniform |
| M0670 | 256 | exp14 | 1024bit-ECFP4 | KNN | 0.919 | 0.900 | 0.873 | 0.957 | 0.928 | 0.838 | 0.911 | 3 uniform |
| M0936 | 256 | exp15 | 2048bit-ECFP6 | SVM | 0.915 | 0.905 | 0.881 | 0.943 | 0.924 | 0.829 | 0.912 | 1 0.1 |
| M0363 | 512 | exp16 | 1024bit-ECFP4 | SVM | 0.934 | 0.925 | 0.907 | 0.957 | 0.941 | 0.868 | 0.917 | 500 0.05 |
| M0837 | 256 | exp17 | 1024bit-ECFP6 | KNN | 0.927 | 0.918 | 0.898 | 0.950 | 0.934 | 0.852 | 0.916 | 3 distance |
| M1223 | 128 | exp18 | 1024bit-ECFP6 | RF | 0.934 | 0.943 | 0.932 | 0.936 | 0.940 | 0.868 | 0.901 | 900 |
| M0921 | 256 | exp19 | 2048bit-ECFP6 | KNN | 0.950 | 0.932 | 0.915 | 0.979 | 0.955 | 0.900 | 0.910 | 5 distance |

**Table S6.** Five average models used to construct the consensus model 2 (**CM02**).

| **AM** | **FS** | **FP** | **method** | **Accuracy**  **(test)** | **Precision**  **(test)** | **Specificity**  **(test)** | **Sensitivity**  **(test)** | **F1-score (test)** | **MCC**  **(test)** | **F1-score**  **(CV)** | $\boldsymbol{\Delta}$**F1-score** |
| --- | --- | --- | --- | --- | --- | --- | --- | --- | --- | --- | --- |
| AM32 | 512 | 2048bit-ECFP4 | SVM | 0.917(0.011) | 0.911(0.020) | 0.889(0.029) | 0.939(0.017) | 0.925(0.010) | 0.833(0.022) | 0.922(0.007) | 0.009(0.010) |
| AM28 | 512 | 1024bit-ECFP4 | RF | 0.913(0.019) | 0.901(0.023) | 0.875(0.031) | 0.945(0.019) | 0.922(0.017) | 0.826(0.038) | 0.915(0.006) | 0.016(0.013) |
| AM31 | 512 | 2048bit-ECFP4 | KNN | 0.913(0.017) | 0.911(0.023) | 0.891(0.031) | 0.931(0.021) | 0.921(0.015) | 0.824(0.033) | 0.914(0.006) | 0.014(0.012) |
| AM11 | full | 2048bit-ECFP4 | DNN2 | 0.909(0.008) | 0.910(0.014) | 0.890(0.018) | 0.924(0.003) | 0.917(0.007) | 0.816(0.016) | 0.921(0.002) | 0.008(0.002) |
| AM69 | 256 | 2048bit-ECFP6 | GBM | 0.898(0.018) | 0.889(0.022) | 0.861(0.031) | 0.928(0.015) | 0.908(0.016) | 0.794(0.036) | 0.909(0.006) | 0.014(0.012) |

**Table S7.** Five average models used to construct the consensus model 3 (**CM03**).

| **AM** | **FS** | **FP** | **method** | **Accuracy**  **(test)** | **Precision**  **(test)** | **Specificity**  **(test)** | **Sensitivity**  **(test)** | **F1-score (test)** | **MCC**  **(test)** | **F1-score**  **(CV)** | $\boldsymbol{\Delta}$**F1-score** |
| --- | --- | --- | --- | --- | --- | --- | --- | --- | --- | --- | --- |
| AM32 | 512 | 2048bit-ECFP4 | SVM | 0.917(0.011) | 0.911(0.020) | 0.889(0.029) | 0.939(0.017) | 0.925(0.010) | 0.833(0.022) | 0.922(0.007) | 0.009(0.010) |
| AM26 | 512 | 1024bit-ECFP4 | SVM | 0.915(0.016) | 0.909(0.021) | 0.887(0.030) | 0.938(0.021) | 0.923(0.014) | 0.829(0.031) | 0.919(0.007) | 0.014(0.010) |
| AM28 | 512 | 1024bit-ECFP4 | RF | 0.913(0.019) | 0.901(0.023) | 0.875(0.031) | 0.945(0.019) | 0.922(0.017) | 0.826(0.038) | 0.915(0.006) | 0.016(0.013) |
| AM62 | 256 | 1024bit-ECFP6 | SVM | 0.914(0.015) | 0.905(0.022) | 0.881(0.030) | 0.941(0.020) | 0.922(0.013) | 0.827(0.030) | 0.917(0.005) | 0.014(0.009) |
| AM52 | 256 | 1024bit-ECFP4 | RF | 0.911(0.021) | 0.898(0.027) | 0.870(0.037) | 0.946(0.023) | 0.921(0.018) | 0.823(0.042) | 0.917(0.005) | 0.016(0.013) |

**Table S8.** Five average models used to construct the consensus model 4 (**CM04**).

| **AM** | **FS** | **FP** | **method** | **Accuracy**  **(test)** | **Precision**  **(test)** | **Specificity**  **(test)** | **Sensitivity**  **(test)** | **F1-score (test)** | **MCC**  **(test)** | **F1-score**  **(CV)** | $\boldsymbol{\Delta}$**F1-score** |
| --- | --- | --- | --- | --- | --- | --- | --- | --- | --- | --- | --- |
| AM31 | 512 | 2048bit-ECFP4 | KNN | 0.913(0.017) | 0.911(0.023) | 0.891(0.031) | 0.931(0.021) | 0.921(0.015) | 0.824(0.033) | 0.914(0.006) | 0.014(0.012) |
| AM49 | 256 | 1024bit-ECFP4 | KNN | 0.911(0.018) | 0.907(0.023) | 0.884(0.031) | 0.933(0.022) | 0.920(0.016) | 0.822(0.036) | 0.913(0.005) | 0.016(0.010) |
| AM55 | 256 | 2048bit-ECFP4 | KNN | 0.912(0.017) | 0.905(0.022) | 0.881(0.030) | 0.937(0.025) | 0.920(0.015) | 0.823(0.035) | 0.916(0.005) | 0.015(0.010) |
| AM67 | 256 | 2048bit-ECFP6 | KNN | 0.907(0.017) | 0.895(0.021) | 0.867(0.030) | 0.941(0.026) | 0.917(0.015) | 0.815(0.034) | 0.915(0.005) | 0.014(0.012) |
| AM43 | 512 | 2048bit-ECFP6 | KNN | 0.905(0.020) | 0.892(0.021) | 0.863(0.028) | 0.939(0.023) | 0.915(0.018) | 0.809(0.040) | 0.914(0.005) | 0.017(0.013) |

**Table S9.** Five average models used to construct the consensus model 5 (**CM05**).

| **AM** | **FS** | **FP** | **method** | **Accuracy**  **(test)** | **Precision**  **(test)** | **Specificity**  **(test)** | **Sensitivity**  **(test)** | **F1-score (test)** | **MCC**  **(test)** | **F1-score**  **(CV)** | $\boldsymbol{\Delta}$**F1-score** |
| --- | --- | --- | --- | --- | --- | --- | --- | --- | --- | --- | --- |
| AM32 | 512 | 2048bit-ECFP4 | SVM | 0.917(0.011) | 0.911(0.020) | 0.889(0.029) | 0.939(0.017) | 0.925(0.010) | 0.833(0.022) | 0.922(0.007) | 0.009(0.010) |
| AM26 | 512 | 1024bit-ECFP4 | SVM | 0.915(0.016) | 0.909(0.021) | 0.887(0.030) | 0.938(0.021) | 0.923(0.014) | 0.829(0.031) | 0.919(0.007) | 0.014(0.010) |
| AM62 | 256 | 1024bit-ECFP6 | SVM | 0.914(0.015) | 0.905(0.022) | 0.881(0.030) | 0.941(0.020) | 0.922(0.013) | 0.827(0.030) | 0.917(0.005) | 0.014(0.009) |
| AM50 | 256 | 1024bit-ECFP4 | SVM | 0.912(0.016) | 0.909(0.025) | 0.886(0.035) | 0.935(0.021) | 0.921(0.014) | 0.825(0.032) | 0.920(0.005) | 0.014(0.010) |
| AM56 | 256 | 2048bit-ECFP4 | SVM | 0.913(0.016) | 0.910(0.024) | 0.888(0.033) | 0.933(0.017) | 0.921(0.014) | 0.824(0.031) | 0.920(0.007) | 0.015(0.010) |

**Table S10.** Five average models used to construct the consensus model 6 (**CM06**).

| **AM** | **FS** | **FP** | **method** | **Accuracy**  **(test)** | **Precision**  **(test)** | **Specificity**  **(test)** | **Sensitivity**  **(test)** | **F1-score (test)** | **MCC**  **(test)** | **F1-score**  **(CV)** | $\boldsymbol{\Delta}$**F1-score** |
| --- | --- | --- | --- | --- | --- | --- | --- | --- | --- | --- | --- |
| AM69 | 256 | 2048bit-ECFP6 | GBM | 0.898(0.018) | 0.889(0.022) | 0.861(0.031) | 0.928(0.015) | 0.908(0.016) | 0.794(0.036) | 0.909(0.006) | 0.014(0.012) |
| AM63 | 256 | 1024bit-ECFP6 | GBM | 0.897(0.016) | 0.890(0.018) | 0.863(0.026) | 0.926(0.025) | 0.908(0.015) | 0.794(0.032) | 0.904(0.006) | 0.015(0.009) |
| AM51 | 256 | 1024bit-ECFP4 | GBM | 0.898(0.016) | 0.897(0.023) | 0.873(0.031) | 0.919(0.020) | 0.908(0.014) | 0.795(0.032) | 0.909(0.005) | 0.012(0.010) |
| AM33 | 512 | 2048bit-ECFP4 | GBM | 0.898(0.014) | 0.892(0.020) | 0.865(0.029) | 0.926(0.020) | 0.908(0.012) | 0.796(0.028) | 0.906(0.005) | 0.012(0.011) |
| AM57 | 256 | 2048bit-ECFP4 | GBM | 0.896(0.021) | 0.891(0.026) | 0.863(0.036) | 0.924(0.020) | 0.907(0.018) | 0.791(0.042) | 0.912(0.006) | 0.018(0.012) |

**Table S11.** Five average models used to construct the consensus model 7 (**CM07**).

| **AM** | **FS** | **FP** | **method** | **Accuracy**  **(test)** | **Precision**  **(test)** | **Specificity**  **(test)** | **Sensitivity**  **(test)** | **F1-score (test)** | **MCC**  **(test)** | **F1-score**  **(CV)** | $\boldsymbol{\Delta}$**F1-score** |
| --- | --- | --- | --- | --- | --- | --- | --- | --- | --- | --- | --- |
| AM28 | 512 | 1024bit-ECFP4 | RF | 0.913(0.019) | 0.901(0.023) | 0.875(0.031) | 0.945(0.019) | 0.922(0.017) | 0.826(0.038) | 0.915(0.006) | 0.016(0.013) |
| AM52 | 256 | 1024bit-ECFP4 | RF | 0.911(0.021) | 0.898(0.027) | 0.870(0.037) | 0.946(0.023) | 0.921(0.018) | 0.823(0.042) | 0.917(0.005) | 0.016(0.013) |
| AM10 | full | 2048bit-ECFP4 | RF | 0.911(0.016) | 0.902(0.021) | 0.878(0.029) | 0.939(0.021) | 0.920(0.014) | 0.821(0.031) | 0.915(0.006) | 0.015(0.010) |
| AM46 | 512 | 2048bit-ECFP6 | RF | 0.909(0.015) | 0.889(0.018) | 0.857(0.026) | 0.953(0.021) | 0.920(0.014) | 0.819(0.031) | 0.916(0.006) | 0.015(0.009) |
| AM70 | 256 | 2048bit-ECFP6 | RF | 0.908(0.020) | 0.893(0.028) | 0.863(0.039) | 0.946(0.017) | 0.919(0.017) | 0.817(0.040) | 0.918(0.006) | 0.017(0.013) |

**Table S12.** Five average models used to construct the consensus model 8 (**CM08**).

| **AM** | **FS** | **FP** | **method** | **Accuracy**  **(test)** | **Precision**  **(test)** | **Specificity**  **(test)** | **Sensitivity**  **(test)** | **F1-score (test)** | **MCC**  **(test)** | **F1-score**  **(CV)** | $\boldsymbol{\Delta}$**F1-score** |
| --- | --- | --- | --- | --- | --- | --- | --- | --- | --- | --- | --- |
| AM11 | full | 2048bit-ECFP4 | DNN2 | 0.909(0.008) | 0.910(0.014) | 0.890(0.018) | 0.924(0.003) | 0.917(0.007) | 0.816(0.016) | 0.921(0.002) | 0.008(0.002) |
| AM23 | full | 2048bit-ECFP6 | DNN2 | 0.896(0.011) | 0.891(0.018) | 0.865(0.025) | 0.922(0.000) | 0.906(0.009) | 0.790(0.023) | 0.918(0.003) | 0.013(0.010) |
| AM35 | 512 | 2048bit-ECFP4 | DNN2 | 0.898(0.007) | 0.908(0.013) | 0.890(0.019) | 0.906(0.003) | 0.906(0.006) | 0.795(0.015) | 0.927(0.002) | 0.021(0.004) |
| AM05 | full | 1024bit-ECFP4 | DNN2 | 0.896(0.006) | 0.903(0.008) | 0.884(0.010) | 0.906(0.003) | 0.905(0.005) | 0.790(0.013) | 0.918(0.005) | 0.013(0.010) |
| AM29 | 512 | 1024bit-ECFP4 | DNN2 | 0.896(0.006) | 0.907(0.009) | 0.890(0.012) | 0.901(0.006) | 0.904(0.005) | 0.790(0.013) | 0.920(0.004) | 0.016(0.009) |

**Table S13.** Five average models used to construct the consensus model 9 (**CM09**).

| **AM** | **FS** | **FP** | **method** | **Accuracy**  **(test)** | **Precision**  **(test)** | **Specificity**  **(test)** | **Sensitivity**  **(test)** | **F1-score (test)** | **MCC**  **(test)** | **F1-score**  **(CV)** | $\boldsymbol{\Delta}$**F1-score** |
| --- | --- | --- | --- | --- | --- | --- | --- | --- | --- | --- | --- |
| AM06 | full | 1024bit-ECFP4 | DNN3 | 0.907(0.003) | 0.909(0.008) | 0.890(0.012) | 0.922(0.006) | 0.915(0.002) | 0.813(0.007) | 0.917(0.001) | 0.002(0.002) |
| AM36 | 512 | 2048bit-ECFP4 | DNN3 | 0.897(0.012) | 0.904(0.018) | 0.884(0.024) | 0.908(0.010) | 0.906(0.010) | 0.792(0.025) | 0.925(0.002) | 0.019(0.009) |
| AM12 | full | 2048bit-ECFP4 | DNN3 | 0.893(0.007) | 0.882(0.013) | 0.850(0.021) | 0.929(0.020) | 0.905(0.007) | 0.785(0.015) | 0.893(0.004) | 0.012(0.011) |
| AM18 | full | 1024bit-ECFP6 | DNN3 | 0.891(0.013) | 0.890(0.014) | 0.864(0.018) | 0.913(0.009) | 0.901(0.011) | 0.779(0.026) | 0.906(0.005) | 0.013(0.011) |
| AM30 | 512 | 1024bit-ECFP4 | DNN3 | 0.893(0.007) | 0.911(0.003) | 0.895(0.004) | 0.891(0.012) | 0.901(0.007) | 0.786(0.014) | 0.919(0.003) | 0.018(0.009) |

**Table S14.** Comparisons of average models (AM) with the different ECFPs, but with the same SVM method and Full features.

| **Name of average model** | **1024bit-ECFP4**  **[AM02, F1=0.917(0.015)]** | **2048bit-ECFP4**  **[AM08, F1=0.920(0.016)]** | **1024bit-ECFP6**  **[AM14, F1=0.912(0.016)]** | **2048bit-ECFP6**  **[AM20, F1=0.917(0.017)]** |
| --- | --- | --- | --- | --- |
| **1024bit-ECFP4**  **[AM02, F1=0.917(0.015)]** | - | 3.0124544701e-01(N) | 1.2490190370e-02(N) | 9.8554982294e-01(N) |
| **2048bit-ECFP4**  **[AM08, F1=0.920(0.016)]** | 3.0124544701e-01(N) | - | 5.9149298541e-03(N) | 2.5087329151e-01(N) |
| **1024bit-ECFP6**  **[AM14, F1=0.912(0.016)]** | 1.2490190370e-02(N) | 5.9149298541e-03(N) | - | 3.9725073424e-02(N) |
| **2048bit-ECFP6**  **[AM20, F1=0.917(0.017)]** | 9.8554982294e-01(N) | 2.5087329151e-01(N) | 3.9725073424e-02(N) | - |

Note: (1) “N” means that there is no significant difference between two models according to the criterion (p-value<0.0001); (2) the number in the element of matrix is the p-value after two-sample T test for every two average models. (3) ‘-’ indicates that two-sample T test between the same average models will be ignored.

**Table S15.** Comparisons of average models (AM) with the different ECFPs, but with the same GBM method and Full features.

| **Name of average model** | **1024bit-ECFP4**  **[AM03, F1=0.902(0.016)]** | **2048bit-ECFP4**  **[AM09, F1=0.904(0.016)]** | **1024bit-ECFP6**  **[AM15, F1=0.905(0.013)]** | **2048bit-ECFP6**  **[AM21, F1=0.902(0.016)]** |
| --- | --- | --- | --- | --- |
| **1024bit-ECFP4**  **[AM03, F1=0.902(0.016)]** | - | 5.7990793252e-01(N) | 2.7786107431e-01(N) | 9.8846581632e-01(N) |
| **2048bit-ECFP4**  **[AM09, F1=0.904(0.016)]** | 5.7990793252e-01(N) | - | 7.3252367924e-01(N) | 5.5321994133e-01(N) |
| **1024bit-ECFP6**  **[AM15, F1=0.905(0.013)]** | 2.7786107431e-01(N) | 7.3252367924e-01(N) | - | 4.4262986135e-01(N) |
| **2048bit-ECFP6**  **[AM21, F1=0.902(0.016)]** | 9.8846581632e-01(N) | 5.5321994133e-01(N) | 4.4262986135e-01(N) | - |

Note: (1) “N” means that there is no significant difference between two models according to the criterion (p-value<0.0001); (2) the number in the element of matrix is the p-value after two-sample T test for every two average models. (3) ‘-’ indicates that two-sample T test between the same average models will be ignored.

**Table S16.** Comparisons of average models (AM) with the different ECFPs, but with the same RF method and Full features.

| **Name of average model** | **1024bit-ECFP4**  **[AM04, F1=0.918(0.016)]** | **2048bit-ECFP4**  **[AM10, F1=0.920(0.014)]** | **1024bit-ECFP6**  **[AM16, F1=0.916(0.015)]** | **2048bit-ECFP6**  **[AM22, F1=0.918(0.014)]** |
| --- | --- | --- | --- | --- |
| **1024bit-ECFP4**  **[AM04, F1=0.918(0.016)]** | - | 2.0383446066e-01(N) | 1.9976542251e-01(N) | 8.4948811189e-01(N) |
| **2048bit-ECFP4**  **[AM10, F1=0.920(0.014)]** | 2.0383446066e-01(N) | - | 1.6846181231e-02(N) | 7.6536900217e-02(N) |
| **1024bit-ECFP6**  **[AM16, F1=0.916(0.015)]** | 1.9976542251e-01(N) | 1.6846181231e-02(N) | - | 2.9942272532e-01(N) |
| **2048bit-ECFP6**  **[AM22, F1=0.918(0.014)]** | 8.4948811189e-01(N) | 7.6536900217e-02(N) | 2.9942272532e-01(N) | - |

Note: (1) “N” means that there is no significant difference between two models according to the criterion (p-value<0.0001); (2) the number in the element of matrix is the p-value after two-sample T test for every two average models. (3) ‘-’ indicates that two-sample T test between the same average models will be ignored.

**Table S17.** Comparisons of average models (AM) with the different feature numbers, but with the same KNN method and 2048bit-ECFP4.

| **Name of average model** | **Full features**  **[AM07, F1=0.900(0.014)]** | **512 features**  **[AM31, F1=0.921(0.015)]** | **256 features**  **[AM55, F1=0.920(0.015)]** | **128 features**  **[AM79, F1=0.910(0.017)]** |
| --- | --- | --- | --- | --- |
| **Full features**  **[AM07, F1=0.900(0.014)]** | - | 5.7470067780e-06(Y) | 4.0008256150e-05(Y) | 3.5618059840e-02(N) |
| **512 features**  **[AM31, F1=0.921(0.015)]** | 5.7470067780e-06(Y) | - | 9.1237131784e-01(N) | 6.2360712874e-04(N) |
| **256 features**  **[AM55, F1=0.920(0.015)]** | 4.0008256150e-05(Y) | 9.1237131784e-01(N) | - | 2.1500105648e-03(N) |
| **128 features**  **[AM79, F1=0.910(0.017)]** | 3.5618059840e-02(N) | 6.2360712874e-04(N) | 2.1500105648e-03(N) | - |

Note: (1) “N” means that there is no significant difference between two models according to the criterion (p-value<0.0001), while “Y” suggests that there is significant difference between two models according to the criterion (p-value <0.0001); (2) the number in the element of matrix is the p-value after two-sample T test for every two average models. (3) ‘-’ indicates that two-sample T test between the same average models will be ignored.

**Table S18.** Comparisons of average models (AM) with the different feature numbers, but with the same KNN method and 1024bit-ECFP6.

| **Name of average model** | **Full features**  **[AM13, F1=0.898(0.016)]** | **512 features**  **[AM37, F1=0.909(0.016)]** | **256 features**  **[AM61, F1=0.915(0.015)]** | **128 features**  **[AM85, F1=0.907(0.013)]** |
| --- | --- | --- | --- | --- |
| **Full features**  **[AM13, F1=0.898(0.016)]** | - | 2.6483868071e-04(N) | 4.3884514141e-04(N) | 4.3870763670e-02(N) |
| **512 features**  **[AM37, F1=0.909(0.016)]** | 2.6483868071e-04(N) | - | 7.5864681223e-02(N) | 5.5598093598e-01(N) |
| **256 features**  **[AM61, F1=0.915(0.015)]** | 4.3884514141e-04(N) | 7.5864681223e-02(N) | - | 6.2915163096e-03(N) |
| **128 features**  **[AM85, F1=0.907(0.013)]** | 4.3870763670e-02(N) | 5.5598093598e-01(N) | 6.2915163096e-03(N) | - |

Note: (1) “N” means that there is no significant difference between two models according to the criterion (p-value <0.0001), while “Y” suggests that there is significant difference between two models according to the criterion (p-value <0.0001); (2) the number in the element of matrix is the p-value after two-sample T test for every two average models. (3) ‘-’ indicates that two-sample T test between the same average models will be ignored.

**Table S19.** Comparisons of average models (AM) with the different feature numbers, but with the same KNN method and 2048bit-ECFP6.

| **Name of average model** | **Full features**  **[AM19, F1=0.898(0.017)]** | **512 features**  **[AM43, F1=0.915(0.018)]** | **256 features**  **[AM67, F1=0.917(0.015)]** | **128 features**  **[AM91, F1=0.909(0.019)]** |
| --- | --- | --- | --- | --- |
| **Full features**  **[AM19, F1=0.898(0.017)]** | - | 5.1145563979e-04(N) | 5.2562861067e-04(N) | 6.6506844560e-02(N) |
| **512 features**  **[AM43, F1=0.915(0.018)]** | 5.1145563979e-04(N) | - | 5.0545116212e-01(N) | 1.3103490305e-01(N) |
| **256 features**  **[AM67, F1=0.917(0.015)]** | 5.2562861067e-04(N) | 5.0545116212e-01(N) | - | 1.9780834859e-02(N) |
| **128 features**  **[AM91, F1=0.909(0.019)]** | 6.6506844560e-02(N) | 1.3103490305e-01(N) | 1.9780834859e-02(N) | - |

Note: (1) “N” means that there is no significant difference between two models according to the criterion (p-value <0.0001), while “Y” means that there is significant difference between two models according to the criterion (p-value <0.0001); (2) the number in the element of matrix is the p-value after two-sample T test for every two average models. (3) ‘-’ indicates that two-sample T test between the same average models will be ignored.

**Table S20.** Comparisons of average models (AM) with the different feature numbers, but with the same SVM method and 1024bit-ECFP4.

| **Name of average model** | **Full features**  **[AM02, F1=0.917(0.015)]** | **512 features**  **[AM26, F1=0.923(0.014)]** | **256 features**  **[AM50, F1=0.921(0.014)]** | **128 features**  **[AM74, F1=0.914(0.015)]** |
| --- | --- | --- | --- | --- |
| **Full features**  **[AM02, F1=0.917(0.015)]** | - | 2.5444554397e-02(N) | 2.4325519813e-01(N) | 3.9423945848e-01(N) |
| **512 features**  **[AM26, F1=0.923(0.014)]** | 2.5444554397e-02(N) | - | 4.5920688482e-01(N) | 8.1775685141e-03(N) |
| **256 features**  **[AM50, F1=0.921(0.014)]** | 2.4325519813e-01(N) | 4.5920688482e-01(N) | - | 1.9020776377e-03(N) |
| **128 features**  **[AM74, F1=0.914(0.015)]** | 3.9423945848e-01(N) | 8.1775685141e-03(N) | 1.9020776377e-03(N) | - |

Note: (1) “N” means that there is no significant difference between two models according to the criterion (p-value <0.0001), while “Y” suggests that there is significant difference between two models according to the criterion (p-value <0.0001); (2) the number in the element of matrix is the p-value after two-sample T test for every two average models. (3) ‘-’ indicates that two-sample T test between the same average models will be ignored.

**Table S21.** Comparisons of average models (AM) with the different feature numbers, but with the same SVM method and 2048bit-ECFP4.

| **Name of average model** | **Full features**  **[AM08, F1=0.920(0.016)]** | **512 features**  **[AM32, F1=0.925(0.010)]** | **256 features**  **[AM56, F1=0.921(0.014)]** | **128 features**  **[AM80, F1=0.912(0.014)]** |
| --- | --- | --- | --- | --- |
| **Full features**  [AM08, F1=0.920(0.016)] | - | 8.0249290246e-02(N) | 6.8646259435e-01(N) | 3.0607956561e-02(N) |
| **512 features**  [AM32, F1=0.925(0.010)] | 8.0249290246e-02(N) | - | 1.2947814230e-01(N) | 8.1964270390e-04(N) |
| **256 features**  [AM56, F1=0.921(0.014)] | 6.8646259435e-01(N) | 1.2947814230e-01(N) | - | 9.4725521402e-03(N) |
| **128 features**  [AM80, F1=0.912(0.014)] | 3.0607956561e-02(N) | 8.1964270390e-04(N) | 9.4725521402e-03(N) | - |

Note: (1) “N” means that there is no significant difference between two models according to the criterion (p-value <0.0001), while “Y” suggests that there is significant difference between two models according to the criterion (p-value <0.0001); (2) the number in the element of matrix is the p-value after two-sample T test for every two average models. (3) ‘-’ indicates that two-sample T test between the same average models will be ignored.

**Table S22.** Comparisons of average models (AM) with the different feature numbers, but with the same SVM method and 1024bit-ECFP6.

| **Name of average model** | **Full features**  [AM14, F1=0.912(0.016)] | **512 features**  [AM38, F1=0.920(0.016)] | **256 features**  [AM62, F1=0.922(0.013)] | **128 features**  [AM86, F1=0.914(0.016)] |
| --- | --- | --- | --- | --- |
| **Full features**  [AM14, F1=0.912(0.016)] | - | 3.6919582821e-02(N) | 2.8378856837e-03(N) | 4.4445624907e-01(N) |
| **512 features**  [AM38, F1=0.920(0.016)] | 3.6919582821e-02(N) | - | 3.5303582952e-01(N) | 4.6657842725e-02(N) |
| **256 features**  [AM62, F1=0.922(0.013)] | 2.8378856837e-03(N) | 3.5303582952e-01(N) | - | 1.1040236023e-03(N) |
| **128 features**  [AM86, F1=0.914(0.016)] | 4.4445624907e-01(N) | 4.6657842725e-02(N) | 1.1040236023e-03(N) | - |

Note: (1) “N” means that there is no significant difference between two models according to the criterion (p-value <0.0001), while “Y” suggests that there is significant difference between two models according to the criterion (p-value <0.0001); (2) the number in the element of matrix is the p-value after two-sample T test for every two average models. (3) ‘-’ indicates that two-sample T test between the same average models will be ignored.

**Table S23.** Comparisons of average models (AM) with the different feature numbers, but with the same SVM method and 2048bit-ECFP6.

| **Name of average model** | **Full features**  [AM20, F1=0.917(0.017)] | **512 features**  [AM44, F1=0.921(0.013)] | **256 features**  [AM68, F1=0.917(0.014)] | **128 features**  [AM92, F1=0.905(0.015)] |
| --- | --- | --- | --- | --- |
| **Full features**  [AM20, F1=0.917(0.017)] | - | 1.7050428550e-01(N) | 9.6561605998e-01(N) | 1.9428587406e-02(N) |
| **512 features**  [AM44, F1=0.921(0.013)] | 1.7050428550e-01(N) | - | 9.4861743498e-02(N) | 4.9381152076e-05(Y) |
| **256 features**  [AM68, F1=0.917(0.014)] | 9.6561605998e-01(N) | 9.4861743498e-02(N) | - | 2.1093608215e-04(N) |
| **128 features**  [AM92, F1=0.905(0.015)] | 1.9428587406e-02(N) | 4.9381152076e-05(Y) | 2.1093608215e-04(N) | - |

Note: (1) “N” means that there is no significant difference between two models according to the criterion (p-value <0.0001), while “Y” means that there is significant difference between two models according to the criterion (p-value <0.0001); (2) the number in the element of matrix is the p-value after two-sample T test for every two average models. (3) ‘-’ indicates that two-sample T test between the same average models will be ignored.

**Table S24.** Comparisons of average models (AM) with the different feature numbers, but with the same GBM method and 1024bit-ECFP4.

| **Name of average model** | **Full features**  **[AM03, F1=0.902(0.016)]** | **512 features**  **[AM27, F1=0.906(0.016)]** | **256 features**  **[AM51, F1=0.908(0.014)]** | **128 features**  **[AM75, F1=0.906(0.014)]** |
| --- | --- | --- | --- | --- |
| **Full features**  **[AM03, F1=0.902(0.016)]** | - | 1.6069432976e-01(N) | 1.3355377145e-01(N) | 1.5406839320e-01(N) |
| **512 features**  **[AM27, F1=0.906(0.016)]** | 1.6069432976e-01(N) | - | 5.9781885080e-01(N) | 9.2540011894e-01(N) |
| **256 features**  **[AM51, F1=0.908(0.014)]** | 1.3355377145e-01(N) | 5.9781885080e-01(N) | - | 6.4257137128e-01(N) |
| **128 features**  **[AM75, F1=0.906(0.014)]** | 1.5406839320e-01(N) | 9.2540011894e-01(N) | 6.4257137128e-01(N) | - |

Note: (1) “N” means that there is no significant difference between two models according to the criterion (p-value <0.0001), while “Y” suggests that there is significant difference between two models according to the criterion (p-value <0.0001); (2) the number in the element of matrix is the p-value after two-sample T test for every two average models. (3) ‘-’ indicates that two-sample T test between the same average models will be ignored.

**Table S25.** Comparisons of average models (AM) with the different feature numbers, but with the same GBM method and 2048bit-ECFP4.

| **Name of average model** | **Full features**  **[AM09, F1=0.904(0.016)]** | **512 features**  **[AM33, F1=0.908(0.012)]** | **256 features**  **[AM57, F1=0.907(0.018)]** | **128 features**  **[AM81, F1=0.906(0.016)]** |
| --- | --- | --- | --- | --- |
| **Full features**  **[AM09, F1=0.904(0.016)]** | - | 1.2236946004e-02(N) | 3.4128523699e-01(N) | 6.0728106373e-01(N) |
| **512 features**  **[AM33, F1=0.908(0.012)]** | 1.2236946004e-02(N) | - | 5.5680800301e-01(N) | 5.4981602893e-01(N) |
| **256 features**  **[AM57, F1=0.907(0.018)]** | 3.4128523699e-01(N) | 5.5680800301e-01(N) | - | 9.3672328200e-01(N) |
| **128 features**  **[AM81, F1=0.906(0.016)]** | 6.0728106373e-01(N) | 5.4981602893e-01(N) | 9.3672328200e-01(N) | - |

Note: (1) “N” means that there is no significant difference between two models according to the criterion (p-value <0.0001), while “Y” suggests that there is significant difference between two models according to the criterion (p-value <0.0001); (2) the number in the element of matrix is the p-value after two-sample T test for every two average models. (3) ‘-’ indicates that two-sample T test between the same average models will be ignored.

**Table S26.** Comparisons of average models (AM) with the different feature numbers, but with the same GBM method and 1024bit-ECFP6.

| **Name of average model** | **Full features**  **[AM15, F1=0.905(0.013)]** | **512 features**  **[AM39, F1=0.907(0.017)]** | **256 features**  **[AM63, F1=0.908(0.015)]** | **128 features**  **[AM87, F1=0.898(0.017)]** |
| --- | --- | --- | --- | --- |
| **Full features**  **[AM15, F1=0.905(0.013)]** | - | 4.3865007184e-01(N) | 4.6851025609e-01(N) | 9.1292878344e-02(N) |
| **512 features**  **[AM39, F1=0.907(0.017)]** | 4.3865007184e-01(N) | - | 9.5984089480e-01(N) | 3.1514396846e-02(N) |
| **256 features**  **[AM63, F1=0.908(0.015)]** | 4.6851025609e-01(N) | 9.5984089480e-01(N) | - | 3.1092961638e-02(N) |
| **128 features**  **[AM87, F1=0.898(0.017)]** | 9.1292878344e-02(N) | 3.1514396846e-02(N) | 3.1092961638e-02(N) | - |

Note: (1) “N” means that there is no significant difference between two models according to the criterion (p-value <0.0001), while “Y” suggests that there is significant difference between two models according to the criterion (p-value <0.0001); (2) the number in the element of matrix is the p-value after two-sample T test for every two average models. (3) ‘-’ indicates that two-sample T test between the same average models will be ignored.

**Table S27.** Comparisons of average models (AM) with the different feature numbers, but with the same GBM method and 2048bit-ECFP6.

| **Name of average model** | **Full features**  **[AM21, F1=0.902(0.016)]** | **512 features**  **[AM45, F1=0.903(0.014)]** | **256 features**  **[AM69, F1=0.908(0.016)]** | **128 features**  **[AM93, F1=0.905(0.016)]** |
| --- | --- | --- | --- | --- |
| **Full features**  **[AM21, F1=0.902(0.016)]** | - | 6.6056332626e-01(N) | 3.2057175725e-02(N) | 4.8474302720e-01(N) |
| **512 features**  **[AM45, F1=0.903(0.014)]** | 6.6056332626e-01(N) | - | 1.3569839352e-01(N) | 6.5964925354e-01(N) |
| **256 features**  **[AM69, F1=0.908(0.016)]** | 3.2057175725e-02(N) | 1.3569839352e-01(N) | - | 4.1177463558e-01(N) |
| **128 features**  **[AM93, F1=0.905(0.016)]** | 4.8474302720e-01(N) | 6.5964925354e-01(N) | 4.1177463558e-01(N) | - |

Note: (1) “N” means that there is no significant difference between two models according to the criterion (p-value <0.0001), while “Y” means that there is significant difference between two models according to the criterion (p-value <0.0001); (2) the number in the element of matrix is the p-value after two-sample T test for every two average models. (3) ‘-’ indicates that two-sample T test between the same average models will be ignored.

**Table S28.** Comparisons of average models (AM) with the different feature numbers, but with the same RF method and 1024bit-ECFP4.

| **Name of average model** | **Full features**  **[AM04, F1=0.918(0.016)]** | **512 features**  **[AM28, F1=0.922(0.017)]** | **256 features**  **[AM52, F1=0.921(0.018)]** | **128 features**  **[AM76, F1=0.918(0.016)]** |
| --- | --- | --- | --- | --- |
| **Full features**  **[AM04, F1=0.918(0.016)]** | - | 7.8084567617e-03(N) | 1.3313485380e-01(N) | 9.5743572906e-01(N) |
| **512 features**  **[AM28, F1=0.922(0.017)]** | 7.8084567617e-03(N) | - | 3.7416775230e-01(N) | 2.8960088093e-02(N) |
| **256 features**  **[AM52, F1=0.921(0.018)]** | 1.3313485380e-01(N) | 3.7416775230e-01(N) | - | 1.0187333402e-01(N) |
| **128 features**  **[AM76, F1=0.918(0.016)]** | 9.5743572906e-01(N) | 2.8960088093e-02(N) | 1.0187333402e-01(N) | - |

Note: (1) “N” means that there is no significant difference between two models according to the criterion (p-value <0.0001), while “Y” suggests that there is significant difference between two models according to the criterion (p-value <0.0001); (2) the number in the element of matrix is the p-value after two-sample T test for every two average models. (3) ‘-’ indicates that two-sample T test between the same average models will be ignored.

**Table S29.** Comparisons of average models (AM) with the different feature numbers, but with the same RF method and 2048bit-ECFP4.

| **Name of average model** | **Full features**  **[AM10, F1=0.920(0.014)]** | **512 features**  **[AM34, F1=0.919(0.013)]** | **256 features**  **[AM58, F1=0.918(0.015)]** | **128 features**  **[AM82, F1=0.914(0.014)]** |
| --- | --- | --- | --- | --- |
| **Full features**  **[AM10, F1=0.920(0.014)]** | - | 5.7095233733e-01(N) | 3.4963877145e-01(N) | 1.7091994213e-02(N) |
| **512 features**  **[AM34, F1=0.919(0.013)]** | 5.7095233733e-01(N) | - | 5.2057918555e-01(N) | 3.4714454430e-02(N) |
| **256 features**  **[AM58, F1=0.918(0.015)]** | 3.4963877145e-01(N) | 5.2057918555e-01(N) | - | 6.4472761388e-02(N) |
| **128 features**  **[AM82, F1=0.914(0.014)]** | 1.7091994213e-02(N) | 3.4714454430e-02(N) | 6.4472761388e-02(N) | - |

Note: (1) “N” means that there is no significant difference between two models according to the criterion (p-value <0.0001), while “Y” suggests that there is significant difference between two models according to the criterion (p-value <0.0001); (2) the number in the element of matrix is the p-value after two-sample T test for every two average models. (3) ‘-’ indicates that two-sample T test between the same average models will be ignored.

**Table S30.** Comparisons of average models (AM) with the different feature numbers, but with the same RF method and 1024bit-ECFP6.

| **Name of average model** | **Full features**  **[AM16, F1=0.916(0.015)]** | **512 features**  **[AM40, F1=0.919(0.016)]** | **256 features**  **[AM64, F1=0.917(0.014)]** | **128 features**  **[AM88, F1=0.915(0.016)]** |
| --- | --- | --- | --- | --- |
| **Full features**  **[AM16, F1=0.916(0.015)]** | - | 2.1762618646e-02(N) | 4.0691331125e-01(N) | 7.2715261770e-01(N) |
| **512 features**  **[AM40, F1=0.919(0.016)]** | 2.1762618646e-02(N) | - | 4.6941010255e-01(N) | 1.8175042308e-01(N) |
| **256 features**  **[AM64, F1=0.917(0.014)]** | 4.0691331125e-01(N) | 4.6941010255e-01(N) | - | 2.1654857239e-01(N) |
| **128 features**  **[AM88, F1=0.915(0.016)]** | 7.2715261770e-01(N) | 1.8175042308e-01(N) | 2.1654857239e-01(N) | - |

Note: (1) “N” means that there is no significant difference between two models according to the criterion (p-value <0.0001), while “Y” suggests that there is significant difference between two models according to the criterion (p-value <0.0001); (2) the number in the element of matrix is the p-value after two-sample T test for every two average models. (3) ‘-’ indicates that two-sample T test between the same average models will be ignored.

**Table S31.** Comparisons of average models (AM) with the different feature numbers, but with the same RF method and 2048bit-ECFP6.

| **Name of average model** | **Full features**  **[AM22, F1=0.918(0.014)]** | **512 features**  **[AM46, F1=0.920(0.014)]** | **256 features**  **[AM70, F1=0.919(0.017)]** | **128 features**  **[AM94, F1=0.912(0.015)]** |
| --- | --- | --- | --- | --- |
| **Full features**  **[AM22, F1=0.918(0.014)]** | - | 3.4696371030e-01(N) | 6.6449965949e-01(N) | 6.9356615672e-02(N) |
| **512 features**  **[AM46, F1=0.920(0.014)]** | 3.4696371030e-01(N) | - | 6.2832151048e-01(N) | 1.4534249284e-02(N) |
| **256 features**  **[AM70, F1=0.919(0.017)]** | 6.6449965949e-01(N) | 6.2832151048e-01(N) | - | 5.1450092333e-03(N) |
| **128 features**  **[AM94, F1=0.912(0.015)]** | 6.9356615672e-02(N) | 1.4534249284e-02(N) | 5.1450092333e-03(N) | - |

Note: (1) “N” means that there is no significant difference between two models according to the criterion (p-value <0.0001), while “Y” means that there is significant difference between two models according to the criterion (p-value <0.0001); (2) the number in the element of matrix is the p-value after two-sample T test for every two average models. (3) ‘-’ indicates that two-sample T test between the same average models will be ignored.
